# Depleting extracellular vesicles from fetal bovine serum alters proliferation and differentiation of skeletal muscle cells in vitro

**DOI:** 10.1186/s12896-016-0262-0

**Published:** 2016-04-02

**Authors:** Hala Aswad, Audrey Jalabert, Sophie Rome

**Affiliations:** CarMeN laboratory (INSERM 1060, INRA 1397, INSA), Faculté de Médecine Lyon-Sud, University of Lyon, Chemin du Grand Revoyet, Oullins, 69600 France

**Keywords:** Extracellular vesicles, Bovine serum, Proliferation, C2C12, L6, Human myoblasts, Muscle cell, Gene expression, miRNAs

## Abstract

**Background:**

Fetal bovine serum (FBS) contains a wide range of growth factors, hormones, vitamins, amino acids, fatty acids and trace elements required for cell growth. It was shown that animal sera contain also extracellular vesicles (EVs) with important biological properties; thus we wondered whether EVs present in FBS would influence muscle cell phenotype.

EVs were removed from sera by ultracentrifugation (18 h). C2C12, L6 and human primary myoblasts, were grown either in classical media (CM) or in EVs-depleted media. Differentiation was induced by replacing the culture medium either with CM or EV-depleted media. qRT-PCR of relevant genes and miRNA involved in proliferation, differentiation, energy metabolism and EVs formation and secretion were performed.

**Results:**

Growth of myoblasts in EV-free media during proliferation produces the most unfavorable situation for proper myotube formation, when considering C212 and human myoblasts. Removing EVs from serum committed myoblasts to differentiate precociously (induction of myogenin and decreased expression of myomiR involved in myogenesis). C2C12 and human myoblasts, grown constantly in EV-depleted media during proliferation and differentiation, formed less myotubes than in CM. They had a reduced level of myogenin and a strong increase in myostatin expression, a negative regulator of muscle cell differentiation that affects myotube size. This situation was not reversed when confluent myoblasts were switched to CM for differentiation. Like C2C12 and human cells, L6 formed less myotubes in EVs-depleted media. However, as they do not express myostatin, L6 myotubes were larger and expressed higher level of CKTM2 compared to myotubes grown in CM suggesting that they had reached a higher level of differentiation.

**Conclusions:**

Researchers studying the role of muscle EVs in culture conditions should consider that depleting EVs from serum alters the phenotype of muscle cells. Interestingly, the cross-talk between myoblasts and myotubes during myogenesis (Forterre 2014, PLoS One. 2014 Jan 2;9(1):e84153) can be recapitulate by using FBS-EVs as well. This implies that EVs can transfer specific signals to cells from unrelated species and that part of serum EV composition is evolutionarily conserved (e.g.; myomiR are detected in FBS-EVs). EVs in body fluids could have an unsuspected function during embryogenesis and in regulation of cellular processes such as hypertrophy and hyperplasia.

**Electronic supplementary material:**

The online version of this article (doi:10.1186/s12896-016-0262-0) contains supplementary material, which is available to authorized users.

## Background

Fetal bovine serum (FBS) obtained from the clotted blood of the bovine fetus, is a common supplement to in vitro culture media. It contains a wide range of growth factors, hormones, vitamins, amino acids, fatty acids and trace elements required for cell growth and, for this reason, was introduced early in cell biology research [1, 2]. It is well-known that animal sera exhibit very high levels of batch to batch variations which can affect cellular proliferation, differentiation, and function. For instance, considerable lots of FBS contain significant levels of lipopolysaccharides which can affect the production of Tumor Necrosis Factor by macrophages [3]. In the case of the C2C12 cell line, which is typically used as a ‘model systems’ for understanding muscle growth and development, it has been demonstrated that differences in hormonal content depending on serum origin (e.g.; USA *vs* EU) caused a shift in calcium handling, resulting in a dramatic change in muscle cell spontaneous contractility [4]. Recently, it was shown that animal sera contain extracellular vesicles (EVs) (e.g.; exosomes, microparticles and apoptotic bodies) which also have important biological properties ranging from intercellular communication and angiogenesis to cell survival. These vesicles can be distinguished by their size and lipid composition: microvesicles (50–300 nanometers) originate from blebbing of the plasma membrane, exosomes (30–150 nanometers) originate from fusion of late endosomes (multivesicular bodies) with the plasma membrane, and apoptotic bodies (100–5000 nanometers) originate from apoptotic cells. EVs, containing certain combinations of lipids, adhesion and intercellular signaling molecules as well as RNAs, participate in intercellular communication processes [5, 6]. Depending on their origin, EVs can modulate immune-regulatory processes, set up tumor escape mechanisms and mediate regenerative or degenerative processes, amongst others. Numerous studies have reported changes in EV composition induced by modifications of the culture conditions, which can mimic different extracellular environments or different physiological or differentiation states of the secreting cells. Thus EVs in biological fluids such as serum or plasma are now considered as biomarkers for diagnosis purposes [7]. Recently, 3 studies have suggested that EVs from bovine serum could substantially influence cultured cell behavior and phenotype [8–10] as vesicle-depleted FBS have reduced capacity to support cell growth. It was also described that FBS vesicles mediated anchorage-independent growth of breast carcinoma cells [11] and thus were necessary for cellular adhesion. As muscle cells are very dependent on FBS origin for proliferation and differentiation [4], we wondered whether EVs present in FBS also affected myoblasts proliferation and differentiation and thus if FBS-EVs could influence cultured muscle cell phenotype.

Therefore, in this study we have determined the specific effect of FBS-EVs on myoblast proliferation and differentiation using mouse and rat muscle cell lines or human primary myoblasts. Our results show that removal of EVS from FBS affected proliferation of myoblasts and committed cells to differentiate precociously. In addition, the expression of myomiRs important for the myogenic process and induced during myoblast proliferation (*i.e*; miR-1, miR-206, miR-133a) are significantly decreased in C2C12. Myoblasts constantly grown in EV-depleted media during proliferation and differentiation formed less myotubes than myoblasts grown in normal serum. However, this study shows a divergence in muscle cell differentiation, depending on the origins of the myoblasts, towards EV-depleted treatments. Thus, researchers studying the role of muscle EVs in culture conditions should consider that depleting EVs from FBS significantly alters the phenotype of muscle cells by affecting gene and miRNA expressions during proliferation.

## Results

Fetal serums may contain more than a thousand components, many related to factors that have been shown to have a large effect on the muscle cell development. The identities and concentrations of these substances in serum are not well characterized. Therefore the use of serum introduces a large number of confounding variables to any experiment. In this study we tested the role of EVs on myoblast proliferation and differentiation using the mouse cell line C2C12, the rat cell line L6 and human primary myoblasts isolated from muscle biopsies.

**Fig. 1:**
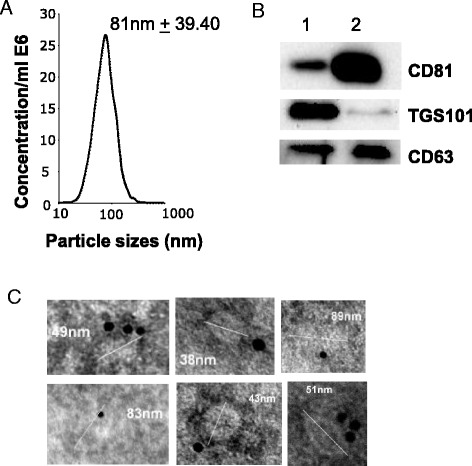
Fetal bovine serum (FBS) contain exosome-like vesicles. FBS-derived vesicles were pellet at 100,000 g for 18 h, resuspended in PBS and re-pelleted at 100,000 g for 70 min. **a** FBS-EV size distributions measured with Nanosight. **b** Immuno-blotting for enriched exosomal proteins in FBS (1); C2C12 released exosomes were used as positive controls (2). TSG101 = Tumor susceptibility gene 101, CD81 = Cluster of Differentiation 81 (tetraspanin). **c** TEM images of purified FBS-EVs. Nanovesicles are labeled with anti-CD81 gold particles to confirm their exosomal origin

EVs were removed from sera by ultracentrifugation for at least 18 h based on previous observations suggesting that shorter centrifugation time periods are not sufficient to get EV-free sera [9]. Proteins of the EV pellets were quantified using the Bradford Protein Assay (BPA). We estimated that 10 % FBS DMEM contained 10 μg of EV-derived proteins per ml of medium. The size of the particles and their concentrations were also analysed with Nanoparticle Tracking Analysis (NTA). NTA calculation of EV-derived proteins produced a concentration which was 4 times less than obtained when quantified with BPA indicating that EVs might be contaminated with other proteins, likely serum albumin and immunoglobulin [12]. The vesicle size distribution determined by NTA displayed a bell-shaped curve with a peak at 81 nm (+39 nm) in agreement with the reported size of EVs (Fig. 1a). As sera are usually filtered at 0.1 um by the supplier, it is likely that the EV pellet is strongly enriched in exosomal vesicles, which are smaller than microparticles and apoptotic bodies [13]. In agreement, classical exosomal markers were detected by WB and TEM on FBS-EVs (Fig. 1b-c, respectively).

### Removal EVs from culture medium affects myoblast proliferation

**Fig. 2:**
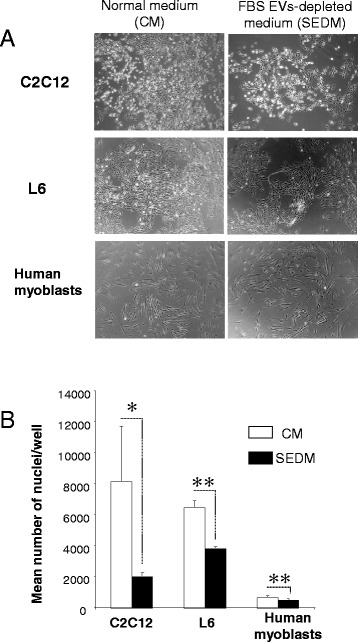
FBS-derived vesicles are involved in myoblast proliferation. **a** light-microscopy (x4) of representative wells from C2C12, L6 or human primary myoblasts grown in 6-well plates either in normal proliferative medium or EV-depleted proliferative medium. **b** Mean number of nuclei per well

Myoblasts, plated at the same density, were grown either in Control Media (CM) or in Serum EVs-Depleted Media (SEDM) (See Additional file 1: Table S1 for growth conditions). After 3 days, light microscopy analysis showed that SEDM affected myoblast proliferation (Fig. 2a). In agreement, the number of nuclei was higher for the cells grown in CM than in SEDM (Fig. 2b).

**Fig. 3:**
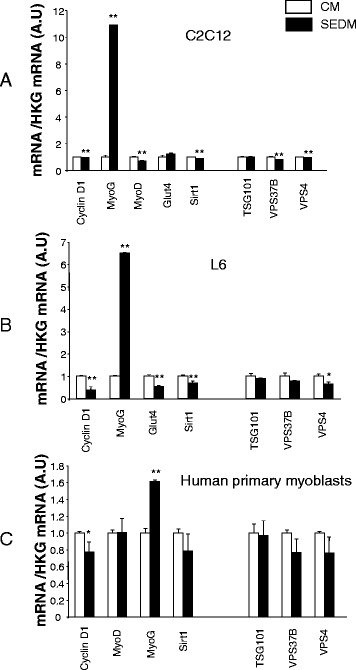
Removing FBS-derived vesicles from bovine serum affects gene expression. Quantitative RT-PCR of relevant genes for proliferation (Cyclin D1, Sirt1), differentiation (MyoD, MyoG), metabolism (Glut4) and vesicle trafficking and EV release (TSG101, VPS37B, VPS4). Data are expressed as fold compared to CM, normalized by house keeping gene (HKG). In white, cells grown in control media containing FBS-EVs (CM), in black, cells grown in CM depleted of FBS-EVs (SEDM). **a** C2C12 myoblasts; **b** L6 rat myoblasts; **c** human primary myoblasts

C2C12 and L6 grown in SEDM had reduced Cyclin D1 and Sirt1 mRNA levels, two important genes involved in cell proliferation [14, 15], compared to CM-treated cells (Fig. 3a-b). Cyclin D1 reduction level was also observed for human myoblasts grown in SEDM (Fig. 3c). It has to be noticed that human myoblasts are grown with only 2 % FBS conversely to L6 and C2C12 which are cultured with 10 % FBS, suggesting that even at low concentrations, FBS-EVs have an impact on cell proliferation. We also measured the expression of genes involved in multivesicular bodie formation (i.e.; VPS37B and VPS4A). Indeed, besides their newly identified roles in cell-cell communications, it should not be forgotten that EVs release is the major route for protein and lipid recycling during plasma membrane remodeling. Thus, EVs secretion is highly stimulated during cell proliferation. We found that VPS37B and VPS4A were decreased at the transcriptional level in C2C12 myoblasts grown in SEDM and VPS4A was reduced in L6 myoblasts, suggesting that reducing proliferation also reduced EV formation and vesicular trafficking. Although not significant, the same tendency was observed for human myoblasts grown without serum EVs (Fig. 3c)

**Fig. 4:**
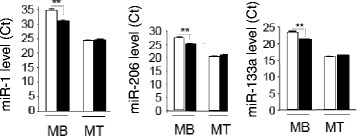
Removing FBS-derived vesicles from bovine serum affects myomiR expression in C2C12. Quantification of miR-1, miR-133a and miR-206 in C2C12, grown either in CM (*white*) or in EVs-depleted proliferation media (*black*). Data are expressed as Ct values

In the case of muscle cells, cell cycle exit and differentiation are coupled during myogenesis [16]. Thus, to determine whether reduced myoblast proliferation in SEDM was correlated with the entrance in the differentiation step, we measured the expression of myogenin, considered as one of the earliest molecular markers for myotube formation in vitro, and MyoD, which cooperate with CyclinD1 during proliferation [17]. As shown on Fig. 3a-b-c, myoblasts grown in SEDM exhibited higher level of myogenin mRNA than control cells, strongly suggesting that these growth conditions induced myoblast growth arrest and committed cells to differentiate precociously. In agreement, MyoD mRNA level was reduced in C2C12, concomitantly to Cyclin D1 (Fig. 3a). In addition, the expression of myomiRs important in the myogenic process, which are induced during myoblast proliferation (*i.e*; miR-1, miR-206, miR-133a [18]), were significantly decreased in C2C12 grown in SEDM, confirming exit from the proliferation step and entrance in the differentiation process (Fig. 4).

### Removing EVs from sera delays myoblasts differentiation

**Fig. 5:**
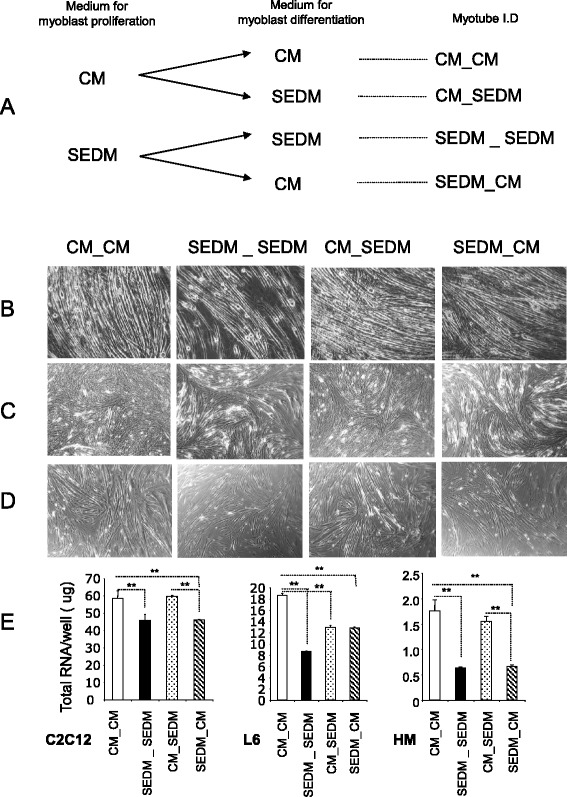
Removing EVs from culture medium sera affect myoblast differentiation. **a** Workflow showing myoblast growth conditions. CM = control media containing FBS-EVs, SEDM = media depleted of FBS-EVs. **b**-**c**-**d** Light-microscopy (x4) of representative wells showing myotubes after 8 days of differentiation; B = C2C12, C = L6 and D = human myotubes. **e** Mean quantities of total RNA/well (NanoDrop quantification) from the 3 types of myotubes after 8 days of differentiation

**Fig. 6:**
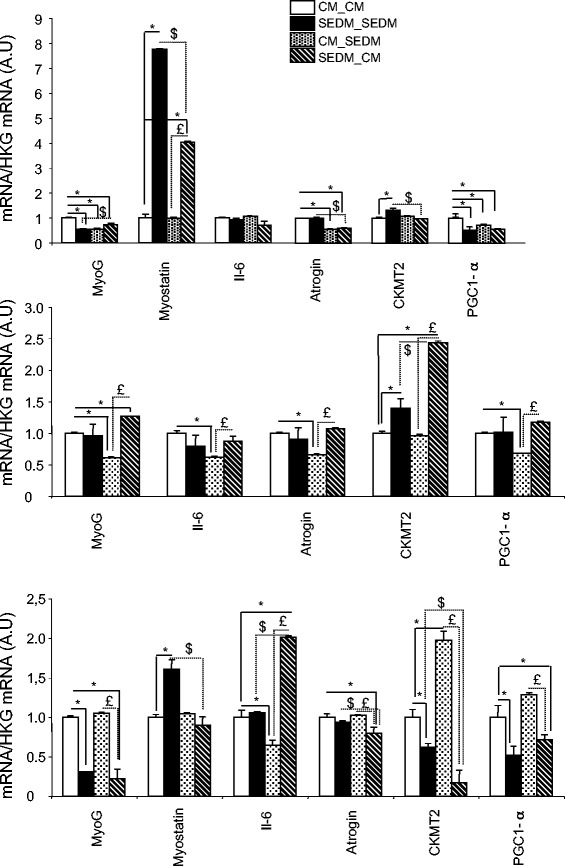
Removing FBS-derived vesicles from bovine serum affects gene expression in myotubes. Quantitative RT-PCR of relevant genes for differentiation (MyoG, CKMT2), myotube size (myostatin, atrogin), metabolism (PGC1-α) or encoding secreted proteins (Il-6). Data are expressed as fold compared to CM_CM, normalized by house keeping gene (HKG). * = p < 0.01 all conditions *vs* CM_CM, $ = p < 0.01 SEDM_SEDM *vs* SEDM_CM. £ = p < 0.01 CM_SEDM *vs* SEDM_CM

Although myoblasts grown in serum EVs-depleted media had reduced rate of proliferation, they were able to proliferate until confluence. After confluence (4 days in proliferation medium), myoblast differentiation was induced by replacing proliferation media with either control media containing serum EVs (CM) or with differentiation media without serum EVs (SEDM) (see Fig. 5a). After 8 days post-differentiation, cells were harvested for RNA extraction and gene expression analysis. As shown on Fig. 5b-c-d-e, cells constantly grown in EV-depleted media during proliferation and differentiation (SEDM_SEDM), formed less myotubes than cells grown in control media (CM_CM). In agreement C2C12 and human cells had a reduced level of myogenin mRNA (MyoG) and a strong increase in myostatin expression (a negative regulator of skeletal muscle size, which inhibits muscle cell differentiation [19]) (Fig. 6). C2C12 and human cells constantly grown in EV-depleted media also had altered mRNA levels of PGC1-α (involved in involved in energy metabolism) and of CKMT2 mRNA level (which has a central role in energy transduction in muscle cells) suggesting that these myotubes had altered substrate utilization compared to those cultivated in normal conditions. Of note, rat L6 myotubes do not express myostatin and conversely to the other cell types, had increased level of CKMT2 when grown in SEDM during proliferation and differentiation. For the 3 cell types, removal of serum EVs did not affect Atrogin-1 expression level, a muscle specific protein regulating muscle atrophy.

Myoblasts grown in SEDM during proliferation were switched to classical CM for differentiation (SEDM_SEDM *vs* SEDM_CM on Fig. 6). After 8 days in differentiation media, C2C12 and L6 myotubes grown in SEDM_CM had higher level of MyoG, and C2C12 and human myotubes had decreased level of myostatin when compared to cells constantly grown without EVs, suggesting that they had reached a higher level of differentiation. However, at the same time, C2C12 and human myotubes had reduced levels of Atrogin-1 suggesting that for these 2 cell types, SEDM_CM treatment increased myotube size without affecting the number myotubes (i.e.; the same quantity of RNA was extracted from myotubes grown in SEDM_CM *vs* SEDM_SEDM although Fig. 5b and d showed a reduced number of myotubes in SEDM_CM condition compared to CM_CM condition). Human myotubes expressed higher level of Il-6 when grown in SEDM_CM than in CM_CM suggesting a modification of their secretome.

L6 myotubes, which do not express myostatin, displayed a specific behavior. When grown in SEDM_SEDM or SEDM_CM compared to CM_CM or CM_SEDM conditions, they formed less myotubes (Fig. 5e), but these myotubes were larger (Fig. 5c) with an increase level of CKMT2 mRNA (Fig. 6). This result suggests that growing myoblast L6 in SEDM during proliferation is favorable for myotube size for this cell type, conversely to C2C12 and human myotubes.

We also tested the reverse experiment i.e.; myoblasts grown in a CM for proliferation were switched in EV-free medium for differentiation (CM_SEDM *vs* CM_CM on Fig. 6). After 8 days in differentiation medium, C2C12 and L6 myotubes grown in CM_SEDM had reduced levels of MyoG, PGC1-α and Atrogin-1 suggesting that these cells were less differentiated than when grown in normal medium. Compared to cells grown in normal conditions, CM_SEDM treated C2C12 and human myotubes formed roughly the same number of myotubes as indicated by the quantity of total RNA recovered in both treatments (Fig. 5d). However, this growth condition affected the number of L6 myotubes (Fig. 5c-d).

Taken altogether, we concluded that growth of myoblasts in EV-free media during proliferation produces the most unfavorable situation for proper myotube formation when considering C212 and human myoblasts. In EV-free media, myostatin is strongly increased and dramatically affects myotubes formation and likely the expression of myomiR involved in myogenesis [20], at least for C2C12. This situation is not reversed when the cells are switched from SEDM to CM for the step of differentiation. As a consequence, proliferation in SEDM affects the number of C2C12 and human myotubes. Surprisingly, growth in EV-free media during proliferation and then switching to CM for differentiation is favorable for rat L6 myotube size.

### Bovine EVs expressed miRNAs involved in muscle myogenesis

**Fig. 7:**
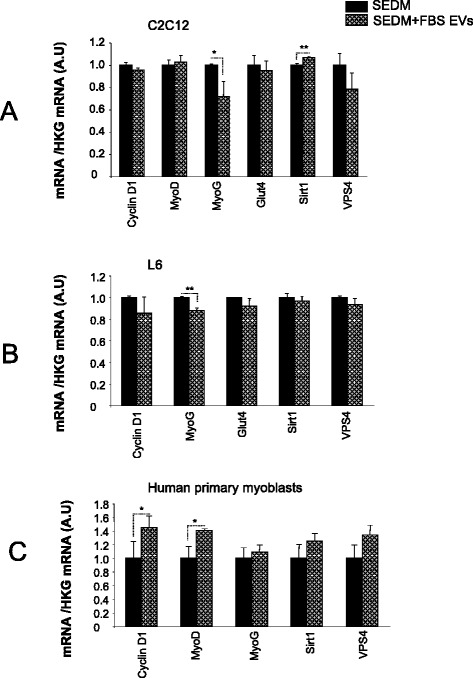
Effect of FBS-EVs on myoblasts gene expression quantified by qRT-PCR. Data are expressed as fold compared to EV-depleted condition, normalized by house keeping gene (HKG). In black, cells grown in serum depleted of its EVs (SEDM), in grey, cells grown in SEDM supplemented with FBS EVs. **a** C2C12 myoblasts; **b** L6 rat myoblasts; **c** human primary myoblasts

Myoblasts were grown either in SEDM or SEDM supplemented with FBS EV-derived proteins (10 μg per ml of medium for C2C12 and L6, or 2 μg for human myoblasts according EVs concentrations in each growth medium). The addition of FBS-EVs induced an increase in Sirt1 mRNA level in C2C12 myoblasts and a decrease in the expression of myogenin both in L6 and C2C12 myoblasts (Fig. 7). Conversely, FBS-EVs did not affect Cyclin D1 and MyoD1 expressions in these 2 cell types, suggesting FBS-EVs repress the induction of differentiation markers during proliferation. On the contrary, the addition of FBS-EVs induced an increase of CyclinD1 and MyoD1 in human primary myoblasts without affecting myogenin, suggesting that in this model, FBS-EVs participate in cell cycle regulation (Fig. 7).

Taken together, these data strongly suggested that FBS-EVs participated in the process of muscle cell myogenesis, and that their roles are additive to other important components of bovine serum.

**Fig. 8:**
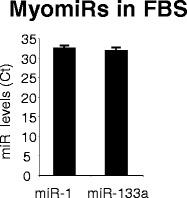
FBS-EVs contain miRNA involved in myogenesis (myomiRs). Data are expressed as Ct values

As it is known that EVs contain miRNAs, we wondered whether FBS would contain myomiR involved in muscle cell differentiation. As shown on Fig. 8, miR-133a and miR-1, which are important for myoblast proliferation [21] were detected by PCR in FBS-EVs. The presence of myomiR in EVs of bovine serum suggested that they might be transferred in recipient myoblasts during cell culture. However, as myomiRs display high evolutionary conservation in animal kingdom it was not possible to verify this hypothesis as murine, rat, human and bovine myomiR sequences are identical [22].

## Discussion

Until now, EVs have been widely studied for their important roles in cancer development and immune response. This is supported by a large number of articles within the literature describing their actions on cell proliferation and differentiation in various tissues and cell types. EVs carry numerous protein, lipid and nucleic acid components that can collectively affect multiple signaling pathways inside recipient cells. Because EVs are released by all tissues, mammals have significant quantities of EVs in circulating blood [23]. Locally, we have found that SkM-released EVs participate in muscle homeostasis [24] and we have demonstrated that myotube-released EVs are involved in the process of myogenesis [25], regulating myoblast proliferation. In order to isolate EVs from cell culture conditioned media, it is important to remove EVs from fetal bovine serum (FBS) to ensure that they are neither erroneously characterized nor competing with the experimental EVs [26]. Doing this, we have observed that C2C12 muscle cells constantly grown in EV-depleted FBS were unable to differentiate and stopped proliferating after 2-3 passages (data not shown). Thus, we suspected that EV-depleted FBS were important for myogenesis. In order to validate this hypothesis we have grown two myoblastic cell lines, mouse C2C12 and rat L6 and one human primary culture of myoblasts, either in normal growth conditions or in serum depleted of its EVs. Then we have quantified the expression of specific genes known for their important role in muscle cell proliferation (Cyclin D1, Sirt1), differentiation (Myogenin, MyoD), energy metabolism (Glut4, PGC1-α, CKMT2) and EVs secretion (TSG101, VPS37B, VPS4). The results of the present study confirm our hypothesis and demonstrate that FBS-EVs participate in the process of myoblast proliferation. Myoblasts grown in EV-depleted medium had affected proliferation and expressed early markers of differentiation. As a consequence, less myotubes were formed, an effect that could be partly reversed by treating cells with FBS-EVs alone. Surpringly, rat cell line L6 was positively affected and formed larger myotubes when grown in EV-depleted medium and then switch in differentiation medium containing EVs, compared to normal growth condition. In fact, EV-depleted proliferation medium induced the expression of myostatin in C2C12 and human myotubes, a negative regulator of skeletal muscle cell size. As L6 cells do not expressed myostatin, induction of precoce differentiation markers during proliferation by EV-depleted medium results in large hypertrophic myotubes. Thus study shows a divergence in muscle cells responses to FBS-EVs depending on their origins.

While preparing this manuscript, 3 other similar studies were published using other cell types, which confirmed that EVs in FBS support cell growth [8–10]. This result was reproduced whatever the commercial source of FBS used and with different serum batches [8].

The evidence that EVs can enter into cells and deliver their cargo is overwhelming. Most experimental evidence suggests that EVs are taken up into endosomal compartments via endocytosis [27] and it has clearly been demonstrated that internalized FBS-EVs interact with lysosomes in the recipient cells [8, 28]. As lysosomes are at the crossroads of several cellular processes such as secretion, plasma membrane repair, signaling and energy metabolism [29], it is likely that internalized EVs participate in various cellular pathways. However, the FBS-EV components that promote cell growth remain to be identified as EVs contain a variety of cargoes that include cell surface receptors, cytosolic and nuclear proteins, metabolic enzymes, RNA transcripts, miRNAs and even DNA, that collectively may participate in EV functions. EVs also contain cellular growth factors like TGFbeta [30–32], proteins of the Wnt [33] and Sonic Hedgehog [34] pathways and cytokine [35] required for proliferation. All these components could participate in FBS-EVs biological actions.

Numerous studies have also demonstrated the transfer of miRNAs from EVs to recipient cells. Interestingly, 2 miRNAs involved in myogenesis are detected in FBS-EVs (i.e.; miR-133a and miR-1). Recently, it was demonstrated that miRNAs exported in EVs can be transferred between unrelated species (e.g.; nematode parasite-derived miRNAs are transferred and regulate murine host genes [36]). It is thus tempting to speculate that bovine EV-miRNAs might participate in the regulation of genes involved in myogenesis in C2C12 recipient cells. Indeed, we have found 675 bovine miRNAs which are predicted to bind the murine myogenin 3’UTR region (Additional file 2: Table S2), including miR-1 and miR-133a. It is tempting to speculate that bovine EV-miRNAs might collectively participate in reducing the level of expression of this gene during myoblast proliferation.

It can be also envisaged that EV internalization might not always be necessary to elicit a phenotypic response by skeletal muscle cells. Indeed, because of their ability to bind to plates coated with fibronectin [37], EVs would aid in myoblast attachment as they express integrin receptors known to mediate cell adhesion [25]. It would therefore be interesting to further study whether FBS-EVs can be used on biomaterial surfaces as a possible strategy to improve performance of cell culture and tissue engineering.

The other important message is that removing FBS-EVs from culture medium affects gene and miRNA expressions and likely the proteome of the cells. Numerous studies, including ours, have found that the contents of EVs often contain distinguishing signatures that allow them to be traced back to their cell of origin. Based on this observation, circulating EVs are now considered as potential disease biomarkers. Therefore a lot of proteomic and genomic analyses have been performed on EVs collected from conditioned medium depleted of FBS-EVs in order to have specific cell-type and tissue EV signatures (http://www.microvesicles.org/). Given the results of the present study, it is conceivable and likely that part of the EV signatures already published might be affected by removal of FBS-EVs during EV collection.

Interestingly, our data showed that we can recapitulate the cross-talk between myoblast and myotubes previously demonstrated [25, 38] by using EVs from serum as well. Indeed FBS-EVs, as myotube-EVs, can affect proliferation of myoblasts and can induce their entrance in the differentiation process. This result implies that bovine EVs can transfer specific signals to cells from unrelated species (i.e.; to mice, rat and human) and thus that part of EV composition is evolutionarily conserved between these mammalian species. Generally speaking, these results suggest that EVs in body fluids could have an unsuspected function during embryogenesis and in the regulation of cellular adaptations that lead to hypertrophy, hyperplasia and metaplasia.

Recently, a number of laboratories have started to develop new strategies to use EVs as vehicles for DNA or siRNA delivery to treat various pathologies [39]. Given the strong impact of EVs on cell proliferation described previously and confirmed in this study, as well as the lack of knowledge on the EV cargoes involved in this biological action, we consider that it is important to keep in mind that EVs are capable of inducing changes in recipient cells.

## Conclusions

Researchers studying the role of muscle EVs in culture conditions should consider that depleting EVs from FBS significantly alters the phenotype of muscle cells by affecting gene and miRNA expressions during proliferation. Further studies are now necessary to elucidate the molecular mechanisms by which EVs in serum enhance cell proliferation in order to develop serum-free media that support rapid cell growth.

## Methods

### Removal and isolation of EVs from FBS and HS

We used FBS from PAA laboratories (PAA clone, # A-15-102, serum with low endotoxin content supplemented with BSA). According to the supplier, blood is removed from the foetus by aseptic cardiac puncture and is then centrifuged to separate blood cells from the serum. The serum is then passed through a cascade of triple 0.1 um filters.

### Preparation of EV-depeted DMEM (D-DMEM)

DMEM, supplemented with all nutrients (4.5 g/l glucose, 1000 IU/ml penicillin, 1000 IU/ml streptomycin, 2 mM L-Glutamine) and 20 % (v/v) heat-inactivated FBS, was ultracentifuged at 100,000 g overnight at 4 °C (Beckman-Coulter, Optima^tm^ L-80-XP ultracentrifuge, type 50-2Ti rotor) [9, 26]. The supernatant was filtered through a 0.22 um filter and diluted with serum-free DMEM to reach the final serum concentration (Additional file 1: Table S1). The pellets from a single sample were pooled, resuspended in 25 ml of PBS and again centrifuged at 100,000 g for 70 min. The EV pellet was finally resuspended in 100ul PBS. EV protein content was quantified using the Bradford protein assay. A NanoSight (Malvern Instruments, Orsay, France) was employed to measure the size of particles [40]. The number of particles and their movement were recorded for 1x60s and analyzed using the NS500 software.

### MicroRNA isolation from culture medium or EVs

Total RNA was extracted from either 100 μl of culture medium (CM or SEDM) or 100 μg of FBS-EVs by using TriPure Isolation Reagent (Roche Applied Science, France). RNA was quantified with a NanoDrop 2000c UV-Vis Spectrophotometer (Thermo Fisher Scientific).

### Origin of myoblasts and culture conditions

Experiments were performed either with immortalized murine C2C12 myoblasts (ATCC® CRL1772™) or rat L6 myoblasts (ATCC® CRL-1458™), or human primary myoblasts. Growth culture conditions are summarized in Additional file 1: Table S1. All myoblasts were grown at 37 °C under 5 % CO2 atmosphere. Differentiation was induced when myoblasts reached confluence and were grown in differentiation media for 8 days.

For the culture of human muscle cells, muscle biopsies (about 200 mg wet weight) were taken under local anaesthesia from the *vastus lateralis* muscle from a lean volonteer who gave his written consent after being informed of the nature, purpose and possible risks of the study. The protocol was approved by the ethics committee of the Hospices Civils de Lyon (#12/111). Differentiated myotubes were prepared according to the procedure previously described [41]. After selection of the myoblasts using a monoclonal antibody (5.1H11; Developmental Studies Hybridoma Bank, Iowa City, IA, USA) combined with magnetic beads, myoblasts were cultured in a Primaria flask (Falcon; Becton Dickinson, Bedford, MA, USA) in a growth medium containing Ham’s F10 supplemented with 2 % Ultroser G (BioSepra, Cergy-Saint-Christophe, France), 2 % FBS and 1 % antibiotics (Invitrogen). At confluence, differentiation into myotubes was induced by changing the medium to DMEM supplemented with 2 % FBS. Four days after initiation of differentiation, cells were polynucleated and expressed specific markers of human skeletal muscle [41].

It has to be noticed that mycoplasma bacteria are around 100 nm in size and can present a potential problem for accurate exosome measurements [42]. Thus, the cells used in this study were tested and found to be negative for mycoplasma.

### mRNA quantitative real-time PCR

Total RNA was extracted from muscle cells by using TriReagent® (Sigma) and quantify with NanoDrop 2000c UV-Vis Spectrophotometer (Thermo Fisher Scientific). RT-PCR was performed using ABsolute QPCR SYBR Green ROX Mix (Abgene, Courtaboeuf, France) with a Rotor-Gene 6000 system (Corbett Life Science, France). Results were normalized either with the gene encoding TBP (TATAbox binding protein) used as the reference for the quantification of gene expression in myotubes [43], or with the gene encoding CSNK2A2 (Casein Kinase 2, Alpha Prime Polypeptide), when gene expression was quantified in myoblast [44].

PCR primers were CCND1 (cyclin D1) S-CTTCCTCTCCAAAATGCCAG, and AS-TGGAGGGTGGGTTGGAAATG, MYOG (myogenin) S-CAACCCAGGAGATCATTTGC and AS-CATATCCTCCACCGTGATGC, TBP S-TTCACATCACAGCTCCCCAC and AS-TGGTGTGCACAGGAGCCAAG, IL-6 (interleukin-6) S-AGTTGCCTTCTTGGGACTGAT and AS-TCCACGATTTCCCAGAGAAC, CKMT2 (Creatine kinase S-type, mitochondrial) S-CTCATCGATGACCACTTTCTG and AS-ATTCCACATGAACTCCCAGC, MYOD1 (myogenic differentiation 1) S-TCCAGCCCGCGCTCCAACTGC and AS-TCGACACGGCCGCACTCTTCC, GLUT4 (SLC2A4) S-GGGTTTCCAGTATGTTGCGG and AS-CTGGGTTTCACCTCCTGCTC, Sirt1 (Sirtuin 1) S-TGAGAAAATGCTGGCCTAATA and AS-GATAAGACGTCATCTTCAGAG, Myostatin S-TGCTGTAACCTTCCCAGGACC and AS-GTGCTCATCGCAGTCAAGCCC, TSG101 S-GCCTCCTGTGACCACTGTTG and AS-CCATCTCTTCCAGTTTCTGGTG, VPS4a S-GCCAAGGAGAGCATTCGAG and AS-CCCTTCACTGTCACTGTCAC, PGC1 alpha S-TCCTCTGACCCCAGAGTCAC and AS-CTTGGTTGGCTTTATGAGGAGG.

Of note, L6 myotubes do not express myostatin; primers for MyoD amplification are not working for L6 RNA, and Glut4 primers are not working for human RNA.

### Western blotting

EV proteins were denatured in Laemmli Buffer (Tris-HCl 50 mM, Glycerol 12 %, SDS 1 %, beta-mercaptoethanol 4 %, Bromophenol blue 0.01 %, PH 6.8, (Sigma)) for 10 min at 100 °C and were migrated on 10 % SDS-PAGE gels (100 μg) and further transferred onto nitrocellulose PVDF membranes. As a positive control, we used muscle-released EVs from C2C12, previously well characterized by our group [25, 38]. Membranes were incubated with anti-Rabbit CD81, CD63, TSG101 [25]. Signals were revealed with Immuno detection kit ECL Luminata Classico (Millipore) and the imager Molecular Image® ChemiDoc™XRS+ (Bio-Rad). Proteins quantification was achieved using ImageLab 3.0 (Bio-Rad).

### Transmission electron microscopy (TEM)

EVs in PBS were adsorbed on 200 Mesh nickel grids coated with formar-C. Immunogold labelling was performed by flotation of grids on drops of reactive media. Non-specific sites were coated with 1 % BSA in 50 mM Tris–HCL, pH 7.4 for 10 min at RT. Antibody incubation was carried out for 4 h at 4 °C in a wet chamber with mouse monoclonal antibody raised against CD81 (sc-31234) antibodies (Santa Cruz Biotechnology) (dilution 1/50) in 1%BSA, 50 mM Tris–HCL, pH 7.4. Grids were successively washed in 50 mM Tris–HCL, pH 7.4 and pH 8.2 at RT. They were then preincubated with 1 % BSA in 50 mM Tris–HCL, pH 8.2 for 10 min at RT and labelled with a goat anti mouse IgG gold-conjugated 10 nm, (Tebu bio, France) diluted 1/80 in 1 % BSA-, 50 mM Tris–HCL, pH 8.2 in a wet chamber for 45 min. Grids were successively washed once in 50 mM Tris–HCL, pH 8.2 then pH 7.4 and in filtrated distilled water at RT. Grids with suspensions were colored with 2 % phosphotunstic acid for 2 min and examined using a JEM Jeol 1400 transmission electron microscope (Tokyo, Japan) equipped with a Orius 600 camera (USA).

### Statistical analysis

All results were expressed in mean +/- standard error of the mean (SEM). A parametric Student *t*-test was used for mean comparison. A *p* value < 0.05 was considered as significant.

### Availability of data and materials

The dataset supporting the conclusions of this article is included within the article (and its additional files).

## Additional files

Additional file 1: Table S1. Growth media used for myoblast proliferation and differentiation. (DOCX 11 kb) Additional file 2: Table S2. List of the 675 circulating bovin miRNAs predicted to bind the 3'-UTR region of the murine myogenin sequence. (PDF 542 kb)

## Abbreviations

CKMT2: Creatine kinase, mitochondrial 2
CM: Conditioned medium
DMEM: Dulbecco’s modified eagle's medium
EVs: Extracellular vesicles
FBS: Fetal bovine serum
Glut4: Solute carrier family 2 (Facilitated Glucose Transporter), member 4
Il-6: Interleukin 6
MyoD: Myogenic differentiation 1
PCG1-α: Peroxisome proliferator-activated receptor gamma, coactivator 1 alpha
SEDM: Serum EVs-depleted media
Sirt1: Sirtuin1
SkM: Skeletal muscle
TGF-beta: Transforming growth factor, beta 1
TSG101: Tumor susceptibility 101
VPS37B: Vacuolar protein sorting 37 homolog B
VPS4A: Vacuolar protein sorting 4 homolog A
