# Supplementary material for: Depleting extracellular vesicles from fetal bovine serum alters proliferation and differentiation of skeletal muscle cells in vitro
Source: BMC Biotechnol. 2016 Apr 2;16:32. doi: 10.1186/s12896-016-0262-0 (PMC4818850; doi:10.1186/s12896-016-0262-0)
Supplement: Additional file 1: Table S1. — Growth media used for myoblast proliferation and differentiation. (DOCX 11 kb) [file 12896_2016_262_MOESM1_ESM.docx]

| **Table S1**: Growth media used for myoblast proliferation and differentiation | | | |
| --- | --- | --- | --- |
|  |  |  |  |
| All media contain 1000 UI/ml penicillin, 1000 UI/ml streptomycin (Invitrogen) | | | |
|  |  |  |  |
| Myoblast origins | Number of cells for plating/cm2 | Proliferation media | Differentiation media |
| C2C12 | 2500 | DMEM (4.5g/l) +10%FBS | DMEM (4.5g/l) +2% HS |
| L6 | 4000 | DMEM (4.5g/l) +10%FBS | DMEM (4.5g/l) +1% FBS |
| Human myoblasts | 3500 | Ham’s F10 + 2% Ultroser G + 2% FBS | DMEM (1.0 g/l) +2% FBS |
|  |  |  |  |
| The number of cells for plating was calculated in order to reach confluence after 4 days of incubation in | | | |
| proliferation media. | |  |  |
| FBS = fetal bovine serum; HS = Horse Serum | | |  |
