# Supplementary material for: Depleting extracellular vesicles from fetal bovine serum alters proliferation and differentiation of skeletal muscle cells in vitro
Source: BMC Biotechnol. 2016 Apr 2;16:32. doi: 10.1186/s12896-016-0262-0 (PMC4818850; doi:10.1186/s12896-016-0262-0)
Supplement: Additional file 2: Table S2. — List of the 675 circulating bovin miRNAs predicted to bind the 3'-UTR region of the murine myogenin sequence. (PDF 542 kb) [file 12896_2016_262_MOESM2_ESM.pdf]

Supplementary Table 2: List of the 675 bovin miRNAs predicted to bind the 3'-UTR region of the murine myogenin sequence

The 3'UTR region of mouse myogenin was retrieved from Genome Browser (https://genome.ucsc.edu)
Then the sequence was scanned for bovine miRNA binding sites by using RNAhybrid (http://mamsap.it.deakin.edu.au/~amitkuma/mirna_targetsnew/sequence.html) which calculates the minimum free energy hybridization of a long and a short RNA, and thus effectively predict microRNA (miRNA) targets

Lower enery cutoff will be more specific and less senstive

Bovine miRNAs	Energy hybridization	Binding positions on mouse myogenin 3'UTR region
bta-miR-26a	-23.6	4
bta-miR-26a	-23.1	424
bta-miR-26a	-20.7	361
bta-miR-26a	-20.3	84
bta-miR-26a	-20.2	472
bta-miR-26a	-19.0	623
bta-miR-26a	-18.6	566
bta-miR-26a	-18.3	243
bta-miR-26a	-17.3	108
bta-miR-26a	-16.9	394
bta-miR-26a	-16.9	166
bta-miR-26a	-15.5	50
bta-miR-26a	-15.2	703
bta-miR-26a	-15.2	740
bta-miR-26a	-15.1	501
bta-miR-26a	-14.9	205
bta-miR-26a	-14.9	456
bta-miR-26a	-14.2	653
bta-miR-26a	-13.7	308
bta-miR-26a	-13.5	596
bta-miR-26a	-12.5	125
bta-miR-26a	-12.1	537
bta-miR-26a	-11.9	342
bta-miR-26a	-11.7	687
bta-miR-26a	-11.4	30
bta-miR-26a	-11.3	416
bta-miR-26a	-11.3	294
bta-miR-26a	-10.0	235
bta-miR-18b	-27.2	413
bta-miR-18b	-23.3	6
bta-miR-18b	-22.9	582
bta-miR-18b	-21.1	85
bta-miR-18b	-20.7	492
bta-miR-18b	-20.2	105
bta-miR-18b	-19.9	369
bta-miR-18b	-19.2	183
bta-miR-18b	-18.5	647
bta-miR-18b	-17.3	536
bta-miR-18b	-17.1	347
bta-miR-18b	-16.9	518
bta-miR-18b	-16.7	387
bta-miR-18b	-16.6	559
bta-miR-18b	-16.4	57
bta-miR-18b	-16.1	615
bta-miR-18b	-15.6	700

bta-miR-18b	-15.4	473
bta-miR-18b	-14.8	307
bta-miR-18b	-14.3	739
bta-miR-18b	-14.2	24
bta-miR-18b	-14.1	274
bta-miR-18b	-13.7	130
bta-miR-18b	-13.5	235
bta-miR-18b	-12.9	449
bta-miR-18b	-12.2	601
bta-miR-18b	-12.1	631
bta-miR-18b	-10.8	252
bta-miR-18b	-10.5	221
bta-miR-18b	-10.2	172
bta-miR-29a	-21.7	595
bta-miR-29a	-21.6	340
bta-miR-29a	-21.2	1
bta-miR-29a	-20.4	403
bta-miR-29a	-20.0	92
bta-miR-29a	-18.7	563
bta-miR-29a	-18.4	29
bta-miR-29a	-18.1	64
bta-miR-29a	-17.5	730
bta-miR-29a	-17.0	432
bta-miR-29a	-16.6	216
bta-miR-29a	-16.5	243
bta-miR-29a	-16.0	462
bta-miR-29a	-15.7	183
bta-miR-29a	-15.7	365
bta-miR-29a	-14.6	509
bta-miR-29a	-12.7	647
bta-miR-29a	-12.4	626
bta-miR-29a	-12.3	142
bta-miR-29a	-11.9	529
bta-miR-29a	-11.4	277
bta-miR-29a	-11.0	700
bta-miR-29a	-11.0	162
bta-miR-29a	-10.7	495
bta-miR-29a	-10.4	119
bta-let-7f	-18.9	456
bta-let-7f	-18.7	375
bta-let-7f	-17.6	85
bta-let-7f	-17.3	296
bta-let-7f	-15.7	648
bta-let-7f	-15.6	582
bta-let-7f	-15.6	559
bta-let-7f	-15.4	526
bta-let-7f	-15.3	171
bta-let-7f	-15.3	685
bta-let-7f	-15.0	487
bta-let-7f	-14.8	414
bta-let-7f	-14.6	738
bta-let-7f	-13.6	271
bta-let-7f	-13.1	244
bta-let-7f	-12.8	16
bta-let-7f	-12.7	122
bta-let-7f	-12.7	149
bta-let-7f	-12.7	359
bta-let-7f	-12.4	623
bta-let-7f	-12.3	199

bta-let-7f	-12.2	40
bta-let-7f	-11.4	708
bta-let-7f	-10.7	403
bta-let-7f	-10.6	61
bta-let-7f	-10.0	514
bta-miR-101	-18.6	9
bta-miR-101	-17.2	495
bta-miR-101	-16.5	50
bta-miR-101	-16.0	410
bta-miR-101	-15.6	376
bta-miR-101	-15.4	573
bta-miR-101	-15.0	545
bta-miR-101	-14.8	638
bta-miR-101	-14.4	277
bta-miR-101	-14.2	67
bta-miR-101	-14.0	100
bta-miR-101	-13.5	456
bta-miR-101	-13.3	607
bta-miR-101	-12.7	257
bta-miR-101	-12.3	140
bta-miR-101	-11.3	433
bta-miR-101	-11.3	527
bta-miR-101	-11.1	354
bta-miR-101	-10.5	200
bta-miR-101	-10.4	39
bta-miR-101	-10.4	165
bta-miR-101	-10.2	232
bta-miR-101	-10.2	597
bta-miR-103	-27.8	108
bta-miR-103	-23.9	354
bta-miR-103	-23.3	238
bta-miR-103	-21.6	469
bta-miR-103	-20.5	573
bta-miR-103	-20.4	407
bta-miR-103	-20.2	166
bta-miR-103	-20.0	50
bta-miR-103	-19.9	534
bta-miR-103	-19.3	497
bta-miR-103	-19.2	9
bta-miR-103	-18.6	429
bta-miR-103	-17.6	623
bta-miR-103	-17.4	653
bta-miR-103	-15.2	89
bta-miR-103	-15.1	277
bta-miR-103	-14.4	739
bta-miR-103	-13.9	200
bta-miR-103	-13.5	137
bta-miR-103	-13.5	683
bta-miR-103	-12.6	31
bta-miR-103	-12.1	125
bta-miR-103	-10.8	391
bta-miR-103	-10.6	328
bta-miR-148a	-21.3	66
bta-miR-148a	-20.7	349
bta-miR-148a	-19.5	469
bta-miR-148a	-19.5	535
bta-miR-148a	-19.5	573
bta-miR-148a	-19.1	383
bta-miR-148a	-18.5	14

bta-miR-148a	-17.4	606
bta-miR-148a	-16.2	517
bta-miR-148a	-15.9	421
bta-miR-148a	-15.6	199
bta-miR-148a	-15.2	552
bta-miR-148a	-15.0	172
bta-miR-148a	-14.3	641
bta-miR-148a	-14.3	263
bta-miR-148a	-13.3	733
bta-miR-148a	-13.1	369
bta-miR-148a	-12.4	106
bta-miR-148a	-12.2	36
bta-miR-148a	-12.2	660
bta-miR-148a	-10.9	445
bta-miR-148a	-10.9	683
bta-miR-148a	-10.8	632
bta-miR-148a	-10.5	59
bta-miR-148a	-10.2	290
bta-miR-148a	-10.1	5
bta-miR-151*	-25.0	415
bta-miR-151*	-24.3	13
bta-miR-151*	-24.3	615
bta-miR-151*	-22.1	185
bta-miR-151*	-21.2	369
bta-miR-151*	-20.8	269
bta-miR-151*	-20.1	112
bta-miR-151*	-19.3	449
bta-miR-151*	-19.1	71
bta-miR-151*	-18.9	498
bta-miR-151*	-18.8	351
bta-miR-151*	-18.3	737
bta-miR-151*	-18.2	228
bta-miR-151*	-17.0	154
bta-miR-151*	-17.0	536
bta-miR-151*	-16.9	649
bta-miR-151*	-16.5	560
bta-miR-151*	-15.7	702
bta-miR-151*	-15.5	633
bta-miR-151*	-14.7	309
bta-miR-151*	-14.3	57
bta-miR-151*	-13.8	586
bta-miR-151*	-13.0	477
bta-miR-151*	-12.5	252
bta-miR-151*	-11.2	1
bta-miR-151*	-10.9	520
bta-miR-151*	-10.3	139
bta-miR-151*	-10.1	213
bta-miR-151	-27.5	261
bta-miR-151	-23.7	67
bta-miR-151	-22.8	474
bta-miR-151	-22.2	452
bta-miR-151	-20.8	210
bta-miR-151	-20.0	169
bta-miR-151	-19.8	626
bta-miR-151	-18.5	402
bta-miR-151	-18.0	368
bta-miR-151	-15.6	192
bta-miR-151	-15.4	502
bta-miR-151	-15.0	559

bta-miR-151	-15.0	612
bta-miR-151	-14.9	9
bta-miR-151	-14.2	573
bta-miR-151	-13.0	45
bta-miR-151	-12.9	111
bta-miR-151	-12.6	703
bta-miR-151	-12.3	596
bta-miR-151	-12.2	437
bta-miR-151	-11.6	342
bta-miR-151	-11.6	309
bta-miR-151	-11.1	520
bta-miR-151	-10.6	650
bta-miR-151	-10.5	146
bta-miR-151	-10.4	86
bta-miR-151	-10.3	242
bta-miR-16b	-19.6	408
bta-miR-16b	-18.4	572
bta-miR-16b	-17.4	483
bta-miR-16b	-16.9	515
bta-miR-16b	-16.7	8
bta-miR-16b	-16.4	51
bta-miR-16b	-16.3	669
bta-miR-16b	-16.2	34
bta-miR-16b	-16.2	252
bta-miR-16b	-15.1	730
bta-miR-16b	-15.0	109
bta-miR-16b	-13.9	560
bta-miR-16b	-13.4	361
bta-miR-16b	-13.2	172
bta-miR-16b	-12.6	605
bta-miR-16b	-11.9	454
bta-miR-16b	-11.4	283
bta-miR-16b	-11.3	658
bta-miR-16b	-11.0	126
bta-miR-16b	-10.0	331
bta-miR-18a	-27.6	3
bta-miR-18a	-24.3	416
bta-miR-18a	-23.4	582
bta-miR-18a	-23.3	490
bta-miR-18a	-20.5	385
bta-miR-18a	-18.4	109
bta-miR-18a	-18.3	87
bta-miR-18a	-18.2	60
bta-miR-18a	-17.6	199
bta-miR-18a	-17.2	364
bta-miR-18a	-17.1	550
bta-miR-18a	-16.9	518
bta-miR-18a	-16.5	654
bta-miR-18a	-16.1	615
bta-miR-18a	-16.1	695
bta-miR-18a	-15.4	473
bta-miR-18a	-15.2	172
bta-miR-18a	-15.1	215
bta-miR-18a	-14.9	257
bta-miR-18a	-14.8	307
bta-miR-18a	-14.6	25
bta-miR-18a	-14.3	739
bta-miR-18a	-12.9	449
bta-miR-18a	-12.5	275

bta-miR-18a	-12.2	156
bta-miR-18a	-12.1	631
bta-miR-18a	-11.6	344
bta-miR-18a	-10.5	606
bta-miR-18a	-10.2	642
bta-miR-20a	-21.7	367
bta-miR-20a	-19.7	425
bta-miR-20a	-18.8	111
bta-miR-20a	-18.6	13
bta-miR-20a	-18.2	473
bta-miR-20a	-18.2	579
bta-miR-20a	-17.1	642
bta-miR-20a	-17.0	57
bta-miR-20a	-16.9	519
bta-miR-20a	-16.0	385
bta-miR-20a	-15.6	183
bta-miR-20a	-14.8	341
bta-miR-20a	-14.3	261
bta-miR-20a	-13.5	139
bta-miR-20a	-12.8	87
bta-miR-20a	-12.7	624
bta-miR-20a	-12.6	668
bta-miR-20a	-12.5	561
bta-miR-20a	-12.4	307
bta-miR-20a	-11.9	162
bta-miR-20a	-11.8	493
bta-miR-20a	-11.7	204
bta-miR-20a	-11.3	740
bta-miR-20a	-10.9	705
bta-miR-20a	-10.0	457
bta-miR-21	-21.9	44
bta-miR-21	-20.8	4
bta-miR-21	-20.3	201
bta-miR-21	-19.4	435
bta-miR-21	-19.2	379
bta-miR-21	-19.2	566
bta-miR-21	-17.9	645
bta-miR-21	-17.7	252
bta-miR-21	-16.8	601
bta-miR-21	-16.6	344
bta-miR-21	-16.0	539
bta-miR-21	-15.9	165
bta-miR-21	-15.6	730
bta-miR-21	-15.3	97
bta-miR-21	-14.7	464
bta-miR-21	-13.6	68
bta-miR-21	-12.6	229
bta-miR-21	-12.5	496
bta-miR-21	-11.9	147
bta-miR-21	-10.5	671
bta-miR-21*	-28.7	106
bta-miR-21*	-24.7	165
bta-miR-21*	-23.3	407
bta-miR-21*	-23.2	357
bta-miR-21*	-22.8	1
bta-miR-21*	-22.6	26
bta-miR-21*	-21.6	559
bta-miR-21*	-21.2	68
bta-miR-21*	-20.9	228

bta-miR-21*	-20.3	620
bta-miR-21*	-20.1	423
bta-miR-21*	-19.2	479
bta-miR-21*	-19.0	129
bta-miR-21*	-18.8	532
bta-miR-21*	-16.9	201
bta-miR-21*	-16.4	710
bta-miR-21*	-15.8	265
bta-miR-21*	-15.7	499
bta-miR-21*	-15.0	653
bta-miR-21*	-13.6	338
bta-miR-21*	-13.3	308
bta-miR-21*	-13.1	94
bta-miR-21*	-13.0	592
bta-miR-21*	-12.9	447
bta-miR-21*	-12.8	687
bta-miR-21*	-12.2	472
bta-miR-21*	-11.2	294
bta-miR-21*	-10.9	393
bta-miR-21*	-10.6	320
bta-miR-21*	-10.5	330
bta-miR-221	-29.9	166
bta-miR-221	-23.0	28
bta-miR-221	-22.3	361
bta-miR-221	-21.7	600
bta-miR-221	-21.3	405
bta-miR-221	-20.5	242
bta-miR-221	-20.5	481
bta-miR-221	-20.1	648
bta-miR-221	-19.9	67
bta-miR-221	-19.7	429
bta-miR-221	-18.9	188
bta-miR-221	-18.6	508
bta-miR-221	-18.2	5
bta-miR-221	-18.2	98
bta-miR-221	-17.0	119
bta-miR-221	-16.8	622
bta-miR-221	-16.7	460
bta-miR-221	-16.2	216
bta-miR-221	-16.0	273
bta-miR-221	-15.8	582
bta-miR-221	-15.4	142
bta-miR-221	-15.2	731
bta-miR-221	-14.3	339
bta-miR-221	-12.5	545
bta-miR-221	-11.0	564
bta-miR-221	-10.6	320
bta-miR-221	-10.3	391
bta-miR-221	-10.2	135
bta-miR-222	-27.3	409
bta-miR-222	-26.6	162
bta-miR-222	-24.2	30
bta-miR-222	-23.0	460
bta-miR-222	-22.4	5
bta-miR-222	-21.8	514
bta-miR-222	-21.1	262
bta-miR-222	-21.0	109
bta-miR-222	-20.8	596
bta-miR-222	-19.3	361

bta-miR-222	-18.8	67
bta-miR-222	-18.7	216
bta-miR-222	-18.0	650
bta-miR-222	-17.6	192
bta-miR-222	-17.4	236
bta-miR-222	-17.2	429
bta-miR-222	-16.6	580
bta-miR-222	-15.2	90
bta-miR-222	-15.1	542
bta-miR-222	-14.3	142
bta-miR-222	-14.0	740
bta-miR-222	-13.9	493
bta-miR-222	-13.1	247
bta-miR-222	-13.0	391
bta-miR-222	-12.8	324
bta-miR-222	-11.3	563
bta-miR-222	-10.9	669
bta-miR-222	-10.3	298
bta-miR-222	-10.2	135
bta-miR-222	-10.2	690
bta-miR-26b	-24.7	424
bta-miR-26b	-19.7	84
bta-miR-26b	-19.7	581
bta-miR-26b	-18.0	5
bta-miR-26b	-17.9	363
bta-miR-26b	-17.4	340
bta-miR-26b	-17.2	398
bta-miR-26b	-15.9	509
bta-miR-26b	-15.8	210
bta-miR-26b	-15.0	472
bta-miR-26b	-14.8	455
bta-miR-26b	-14.7	701
bta-miR-26b	-14.3	105
bta-miR-26b	-14.2	626
bta-miR-26b	-14.1	563
bta-miR-26b	-13.7	385
bta-miR-26b	-13.4	162
bta-miR-26b	-13.3	537
bta-miR-26b	-13.1	243
bta-miR-26b	-12.6	50
bta-miR-26b	-12.6	740
bta-miR-26b	-11.9	305
bta-miR-26b	-10.7	501
bta-miR-26b	-10.3	235
bta-miR-26b	-10.1	125
bta-miR-27a-5p	-30.7	408
bta-miR-27a-5p	-27.1	146
bta-miR-27a-5p	-25.8	3
bta-miR-27a-5p	-25.5	118
bta-miR-27a-5p	-25.4	73
bta-miR-27a-5p	-23.9	185
bta-miR-27a-5p	-23.2	585
bta-miR-27a-5p	-21.8	465
bta-miR-27a-5p	-21.3	214
bta-miR-27a-5p	-20.6	644
bta-miR-27a-5p	-19.9	344
bta-miR-27a-5p	-19.5	51
bta-miR-27a-5p	-19.2	550
bta-miR-27a-5p	-18.7	276

bta-miR-27a-5p	-17.6	489
bta-miR-27a-5p	-17.2	378
bta-miR-27a-5p	-17.2	238
bta-miR-27a-5p	-17.0	450
bta-miR-27a-5p	-16.1	100
bta-miR-27a-5p	-14.7	251
bta-miR-27a-5p	-14.0	519
bta-miR-27a-5p	-14.0	429
bta-miR-27a-5p	-13.3	739
bta-miR-27a-5p	-12.0	435
bta-miR-27a-5p	-11.9	697
bta-miR-27a-5p	-11.7	37
bta-miR-27a-5p	-11.1	170
bta-miR-27a-5p	-10.3	630
bta-miR-27a-5p	-10.2	575
bta-miR-27a-5p	-10.1	319
bta-miR-27a-3p	-22.0	177
bta-miR-27a-3p	-20.6	480
bta-miR-27a-3p	-19.5	70
bta-miR-27a-3p	-19.4	632
bta-miR-27a-3p	-19.4	418
bta-miR-27a-3p	-18.7	16
bta-miR-27a-3p	-17.5	114
bta-miR-27a-3p	-17.4	599
bta-miR-27a-3p	-17.1	507
bta-miR-27a-3p	-16.5	449
bta-miR-27a-3p	-16.2	407
bta-miR-27a-3p	-16.1	280
bta-miR-27a-3p	-15.6	41
bta-miR-27a-3p	-15.5	576
bta-miR-27a-3p	-15.4	559
bta-miR-27a-3p	-15.1	231
bta-miR-27a-3p	-14.3	350
bta-miR-27a-3p	-13.6	738
bta-miR-27a-3p	-13.3	130
bta-miR-27a-3p	-13.3	202
bta-miR-27a-3p	-12.6	155
bta-miR-27a-3p	-12.0	537
bta-miR-27a-3p	-11.8	313
bta-miR-27a-3p	-11.8	472
bta-miR-27a-3p	-11.4	381
bta-miR-27a-3p	-11.4	268
bta-miR-27a-3p	-11.0	665
bta-miR-27a-3p	-10.6	108
bta-miR-27a-3p	-10.5	393
bta-miR-27a-3p	-10.2	623
bta-miR-27a-3p	-10.1	693
bta-miR-30d	-27.2	456
bta-miR-30d	-24.6	72
bta-miR-30d	-24.1	586
bta-miR-30d	-22.1	166
bta-miR-30d	-20.2	512
bta-miR-30d	-20.0	419
bta-miR-30d	-19.7	5
bta-miR-30d	-18.2	282
bta-miR-30d	-17.8	29
bta-miR-30d	-17.5	263
bta-miR-30d	-17.1	205
bta-miR-30d	-15.9	98

bta-miR-30d	-15.8	626
bta-miR-30d	-15.6	390
bta-miR-30d	-15.2	358
bta-miR-30d	-14.8	559
bta-miR-30d	-14.6	539
bta-miR-30d	-14.3	653
bta-miR-30d	-13.6	403
bta-miR-30d	-13.5	499
bta-miR-30d	-13.1	236
bta-miR-30d	-11.5	184
bta-miR-30d	-11.3	58
bta-miR-30d	-11.1	740
bta-miR-30d	-10.9	698
bta-miR-30d	-10.5	342
bta-miR-30d	-10.4	316
bta-miR-30d	-10.4	127
bta-miR-30d	-10.3	572
bta-miR-320	-25.9	156
bta-miR-320	-24.1	96
bta-miR-320	-23.5	360
bta-miR-320	-22.6	257
bta-miR-320	-22.2	408
bta-miR-320	-21.1	474
bta-miR-320	-21.1	562
bta-miR-320	-20.9	118
bta-miR-320	-20.7	492
bta-miR-320	-20.2	51
bta-miR-320	-20.1	30
bta-miR-320	-18.8	2
bta-miR-320	-18.7	307
bta-miR-320	-18.7	624
bta-miR-320	-18.5	133
bta-miR-320	-18.0	235
bta-miR-320	-17.6	185
bta-miR-320	-17.2	388
bta-miR-320	-17.2	667
bta-miR-320	-17.0	206
bta-miR-320	-16.8	428
bta-miR-320	-14.4	644
bta-miR-320	-14.1	713
bta-miR-320	-13.9	326
bta-miR-320	-12.9	591
bta-miR-320	-12.8	88
bta-miR-320	-12.0	374
bta-miR-320	-11.2	530
bta-miR-484	-30.9	69
bta-miR-484	-29.9	412
bta-miR-484	-29.6	609
bta-miR-484	-25.8	8
bta-miR-484	-24.2	448
bta-miR-484	-21.0	263
bta-miR-484	-21.0	343
bta-miR-484	-21.0	476
bta-miR-484	-20.6	172
bta-miR-484	-20.0	220
bta-miR-484	-19.6	114
bta-miR-484	-16.3	566
bta-miR-484	-15.0	738
bta-miR-484	-14.8	631

bta-miR-484	-14.6	434
bta-miR-484	-14.6	41
bta-miR-484	-14.1	242
bta-miR-484	-13.9	369
bta-miR-484	-13.9	597
bta-miR-484	-13.5	656
bta-miR-484	-12.7	403
bta-miR-484	-12.1	536
bta-miR-484	-11.9	154
bta-miR-484	-11.5	308
bta-miR-484	-11.5	204
bta-miR-484	-11.3	710
bta-miR-484	-10.3	97
bta-miR-499	-19.9	53
bta-miR-499	-19.0	486
bta-miR-499	-18.7	383
bta-miR-499	-16.6	560
bta-miR-499	-16.5	648
bta-miR-499	-15.8	204
bta-miR-499	-15.8	600
bta-miR-499	-15.5	351
bta-miR-499	-14.7	418
bta-miR-499	-14.4	104
bta-miR-499	-14.3	84
bta-miR-499	-14.3	4
bta-miR-499	-14.0	520
bta-miR-499	-13.0	22
bta-miR-499	-12.8	178
bta-miR-499	-12.7	269
bta-miR-499	-12.6	541
bta-miR-499	-12.3	681
bta-miR-499	-12.0	297
bta-miR-499	-11.8	574
bta-miR-499	-11.5	159
bta-miR-499	-11.3	242
bta-miR-499	-11.1	471
bta-miR-499	-10.8	401
bta-miR-499	-10.3	147
bta-miR-499	-10.2	696
bta-miR-499	-10.1	233
bta-miR-99a	-27.8	615
bta-miR-99a	-23.5	494
bta-miR-99a	-18.0	573
bta-miR-99a	-17.9	443
bta-miR-99a	-17.3	1
bta-miR-99a	-17.3	66
bta-miR-99a	-15.5	415
bta-miR-99a	-14.7	476
bta-miR-99a	-14.3	736
bta-miR-99a	-13.8	35
bta-miR-99a	-13.4	106
bta-miR-99a	-13.3	202
bta-miR-99a	-12.7	654
bta-miR-99a	-12.7	702
bta-miR-99a	-12.4	271
bta-miR-99a	-12.3	373
bta-miR-99a	-12.2	602
bta-miR-99a	-11.6	172
bta-miR-99a*	-22.5	162

bta-miR-99a*	-22.4	60
bta-miR-99a*	-21.9	473
bta-miR-99a*	-20.8	361
bta-miR-99a*	-20.3	260
bta-miR-99a*	-20.0	429
bta-miR-99a*	-19.5	595
bta-miR-99a*	-19.3	408
bta-miR-99a*	-18.4	624
bta-miR-99a*	-16.7	98
bta-miR-99a*	-16.2	189
bta-miR-99a*	-15.6	13
bta-miR-99a*	-15.3	33
bta-miR-99a*	-15.1	208
bta-miR-99a*	-14.7	230
bta-miR-99a*	-14.3	330
bta-miR-99a*	-13.7	142
bta-miR-99a*	-13.5	79
bta-miR-99a*	-13.3	572
bta-miR-99a*	-13.1	514
bta-miR-99a*	-12.8	119
bta-miR-99a*	-12.0	489
bta-miR-99a*	-11.7	737
bta-miR-99a*	-11.4	552
bta-miR-99a*	-11.0	177
bta-miR-99a*	-10.9	649
bta-miR-99a*	-10.4	135
bta-miR-99a*	-10.4	324
bta-miR-125a	-31.1	606
bta-miR-125a	-22.2	66
bta-miR-125a	-21.3	447
bta-miR-125a	-21.1	264
bta-miR-125a	-20.0	494
bta-miR-125a	-19.5	216
bta-miR-125a	-18.0	410
bta-miR-125a	-17.3	10
bta-miR-125a	-15.2	576
bta-miR-125a	-14.6	148
bta-miR-125a	-14.4	106
bta-miR-125a	-13.4	382
bta-miR-125a	-12.7	35
bta-miR-125a	-12.3	654
bta-miR-125a	-11.7	739
bta-miR-125a	-11.4	199
bta-miR-125a	-10.8	556
bta-miR-125a	-10.6	337
bta-miR-125a	-10.6	93
bta-miR-125b	-27.2	606
bta-miR-125b	-21.8	55
bta-miR-125b	-21.5	263
bta-miR-125b	-19.2	476
bta-miR-125b	-18.4	33
bta-miR-125b	-18.1	215
bta-miR-125b	-17.8	408
bta-miR-125b	-16.6	5
bta-miR-125b	-16.2	434
bta-miR-125b	-16.1	567
bta-miR-125b	-15.9	147
bta-miR-125b	-15.6	382
bta-miR-125b	-14.2	121

bta-miR-125b	-13.9	354
bta-miR-125b	-13.7	654
bta-miR-125b	-11.3	299
bta-miR-125b	-11.2	373
bta-miR-125b	-10.8	453
bta-miR-125b	-10.6	114
bta-miR-125b	-10.5	97
bta-miR-126*	-16.1	565
bta-miR-126*	-16.0	425
bta-miR-126*	-15.8	377
bta-miR-126*	-15.6	18
bta-miR-126*	-14.8	618
bta-miR-126*	-14.6	62
bta-miR-126*	-14.5	116
bta-miR-126*	-14.4	479
bta-miR-126*	-14.3	546
bta-miR-126*	-13.4	30
bta-miR-126*	-13.0	659
bta-miR-126*	-12.5	271
bta-miR-126*	-12.4	518
bta-miR-126*	-12.2	594
bta-miR-126*	-12.2	167
bta-miR-126*	-12.1	96
bta-miR-126*	-11.8	359
bta-miR-126*	-11.7	463
bta-miR-126*	-11.5	235
bta-miR-126*	-11.3	206
bta-miR-126*	-11.2	126
bta-miR-126*	-10.9	408
bta-miR-126*	-10.8	257
bta-miR-126*	-10.6	294
bta-miR-126*	-10.6	341
bta-miR-126*	-10.1	45
bta-miR-126	-16.6	606
bta-miR-126	-15.9	271
bta-miR-126	-15.7	465
bta-miR-126	-15.4	546
bta-miR-126	-15.1	66
bta-miR-126	-15.1	654
bta-miR-126	-14.8	488
bta-miR-126	-14.1	343
bta-miR-126	-13.8	109
bta-miR-126	-13.7	1
bta-miR-126	-13.7	408
bta-miR-126	-13.5	22
bta-miR-126	-13.3	577
bta-miR-126	-12.4	244
bta-miR-126	-12.3	206
bta-miR-126	-11.8	698
bta-miR-126	-11.7	145
bta-miR-126	-11.5	527
bta-miR-126	-11.1	292
bta-miR-126	-10.8	512
bta-miR-126	-10.6	185
bta-miR-126	-10.5	367
bta-miR-126	-10.1	738
bta-miR-128	-23.7	452
bta-miR-128	-22.8	11
bta-miR-128	-21.1	611

bta-miR-128	-19.8	737
bta-miR-128	-19.8	570
bta-miR-128	-19.3	411
bta-miR-128	-18.9	68
bta-miR-128	-18.5	348
bta-miR-128	-16.7	366
bta-miR-128	-16.6	592
bta-miR-128	-16.1	220
bta-miR-128	-15.8	506
bta-miR-128	-15.6	174
bta-miR-128	-15.3	482
bta-miR-128	-15.2	539
bta-miR-128	-15.2	440
bta-miR-128	-14.6	652
bta-miR-128	-13.8	114
bta-miR-128	-13.6	268
bta-miR-128	-13.4	423
bta-miR-128	-13.2	201
bta-miR-128	-13.2	38
bta-miR-128	-13.0	124
bta-miR-128	-12.8	242
bta-miR-128	-12.4	557
bta-miR-128	-11.9	142
bta-miR-128	-11.1	1
bta-miR-128	-10.7	338
bta-miR-128	-10.6	154
bta-miR-128	-10.3	385
bta-miR-145	-28.4	620
bta-miR-145	-26.9	83
bta-miR-145	-23.9	449
bta-miR-145	-23.4	400
bta-miR-145	-22.0	214
bta-miR-145	-21.4	575
bta-miR-145	-20.4	259
bta-miR-145	-19.2	363
bta-miR-145	-19.0	173
bta-miR-145	-19.0	1
bta-miR-145	-18.6	502
bta-miR-145	-18.3	710
bta-miR-145	-15.6	59
bta-miR-145	-13.8	431
bta-miR-145	-13.4	191
bta-miR-145	-12.8	236
bta-miR-145	-11.6	613
bta-miR-145	-11.6	638
bta-miR-145	-11.5	548
bta-miR-145	-10.6	687
bta-miR-145	-10.5	342
bta-miR-145	-10.1	145
bta-miR-145	-10.0	315
bta-miR-181a	-21.3	1
bta-miR-181a	-20.9	155
bta-miR-181a	-20.1	594
bta-miR-181a	-19.3	520
bta-miR-181a	-18.7	364
bta-miR-181a	-17.7	397
bta-miR-181a	-17.3	256
bta-miR-181a	-17.2	482
bta-miR-181a	-17.0	623

bta-miR-181a	-16.5	22
bta-miR-181a	-16.5	59
bta-miR-181a	-16.2	188
bta-miR-181a	-15.8	425
bta-miR-181a	-15.0	335
bta-miR-181a	-14.9	95
bta-miR-181a	-14.7	559
bta-miR-181a	-14.2	274
bta-miR-181a	-13.4	456
bta-miR-181a	-13.4	130
bta-miR-181a	-13.3	734
bta-miR-181a	-13.1	116
bta-miR-181a	-13.0	233
bta-miR-181a	-12.5	695
bta-miR-181a	-11.8	299
bta-miR-181a	-11.6	211
bta-miR-181a	-10.8	145
bta-miR-181a	-10.7	441
bta-miR-181a	-10.2	666
bta-miR-181a	-10.1	319
bta-miR-181a	-10.1	326
bta-miR-199a-5p	-25.2	605
bta-miR-199a-5p	-22.9	407
bta-miR-199a-5p	-22.2	4
bta-miR-199a-5p	-22.0	575
bta-miR-199a-5p	-21.5	83
bta-miR-199a-5p	-18.3	352
bta-miR-199a-5p	-17.6	494
bta-miR-199a-5p	-17.6	60
bta-miR-199a-5p	-17.3	464
bta-miR-199a-5p	-16.5	258
bta-miR-199a-5p	-15.9	372
bta-miR-199a-5p	-15.6	198
bta-miR-199a-5p	-14.9	730
bta-miR-199a-5p	-14.8	170
bta-miR-199a-5p	-14.3	435
bta-miR-199a-5p	-14.1	548
bta-miR-199a-5p	-13.4	232
bta-miR-199a-5p	-13.4	566
bta-miR-199a-5p	-12.7	516
bta-miR-199a-5p	-12.7	653
bta-miR-199a-5p	-11.5	41
bta-miR-199a-5p	-11.3	393
bta-miR-199a-5p	-11.2	278
bta-miR-199a-5p	-11.1	344
bta-miR-199a-5p	-11.0	149
bta-miR-199a-5p	-10.4	695
bta-miR-199a-5p	-10.0	632
bta-miR-199a-3p	-25.2	407
bta-miR-199a-3p	-23.7	509
bta-miR-199a-3p	-22.4	569
bta-miR-199a-3p	-21.0	365
bta-miR-199a-3p	-20.6	50
bta-miR-199a-3p	-19.4	623
bta-miR-199a-3p	-18.8	647
bta-miR-199a-3p	-18.1	482
bta-miR-199a-3p	-17.5	92
bta-miR-199a-3p	-17.1	263
bta-miR-199a-3p	-17.0	119

bta-miR-199a-3p	-16.8	187
bta-miR-199a-3p	-16.6	1
bta-miR-199a-3p	-15.1	340
bta-miR-199a-3p	-15.0	544
bta-miR-199a-3p	-14.6	229
bta-miR-199a-3p	-14.5	723
bta-miR-199a-3p	-13.9	81
bta-miR-199a-3p	-13.7	143
bta-miR-199a-3p	-13.6	34
bta-miR-199a-3p	-13.4	456
bta-miR-199a-3p	-13.0	601
bta-miR-199a-3p	-11.7	166
bta-miR-199a-3p	-11.3	670
bta-miR-199a-3p	-10.8	688
bta-miR-199a-3p	-10.4	359
bta-miR-205	-27.5	603
bta-miR-205	-23.9	576
bta-miR-205	-23.1	472
bta-miR-205	-20.5	624
bta-miR-205	-20.2	414
bta-miR-205	-19.5	10
bta-miR-205	-18.6	71
bta-miR-205	-18.0	128
bta-miR-205	-17.9	262
bta-miR-205	-17.8	154
bta-miR-205	-17.7	200
bta-miR-205	-16.5	454
bta-miR-205	-16.3	351
bta-miR-205	-15.1	188
bta-miR-205	-13.3	513
bta-miR-205	-13.0	114
bta-miR-205	-12.9	737
bta-miR-205	-12.8	710
bta-miR-205	-12.0	654
bta-miR-205	-11.5	138
bta-miR-205	-11.5	393
bta-miR-205	-11.4	39
bta-miR-205	-10.7	57
bta-miR-205	-10.3	327
bta-miR-205	-10.3	560
bta-miR-205	-10.1	315
bta-miR-205	-10.0	376
bta-miR-27b	-22.3	415
bta-miR-27b	-20.2	100
bta-miR-27b	-20.2	177
bta-miR-27b	-19.4	632
bta-miR-27b	-19.0	66
bta-miR-27b	-18.8	480
bta-miR-27b	-18.7	13
bta-miR-27b	-17.9	114
bta-miR-27b	-17.7	199
bta-miR-27b	-16.8	555
bta-miR-27b	-16.0	349
bta-miR-27b	-15.8	238
bta-miR-27b	-15.6	599
bta-miR-27b	-15.4	516
bta-miR-27b	-15.1	128
bta-miR-27b	-15.0	575
bta-miR-27b	-14.8	41

bta-miR-27b	-14.7	449
bta-miR-27b	-14.0	271
bta-miR-27b	-13.9	393
bta-miR-27b	-13.6	737
bta-miR-27b	-12.2	313
bta-miR-27b	-11.8	472
bta-miR-27b	-11.7	156
bta-miR-27b	-11.4	381
bta-miR-27b	-11.0	665
bta-miR-27b	-10.9	687
bta-miR-27b	-10.2	623
bta-miR-27b	-10.1	258
bta-miR-30b-5p	-24.3	12
bta-miR-30b-5p	-21.0	605
bta-miR-30b-5p	-19.5	520
bta-miR-30b-5p	-18.3	351
bta-miR-30b-5p	-17.7	435
bta-miR-30b-5p	-17.5	65
bta-miR-30b-5p	-17.4	414
bta-miR-30b-5p	-16.8	653
bta-miR-30b-5p	-15.6	539
bta-miR-30b-5p	-15.6	586
bta-miR-30b-5p	-15.6	487
bta-miR-30b-5p	-15.0	216
bta-miR-30b-5p	-15.0	265
bta-miR-30b-5p	-13.9	37
bta-miR-30b-5p	-13.7	629
bta-miR-30b-5p	-13.2	166
bta-miR-30b-5p	-12.9	184
bta-miR-30b-5p	-12.3	92
bta-miR-30b-5p	-12.2	559
bta-miR-30b-5p	-12.0	233
bta-miR-30b-5p	-11.5	198
bta-miR-30b-5p	-10.8	456
bta-miR-30b-5p	-10.3	698
bta-miR-30b-3p	-25.3	201
bta-miR-30b-3p	-24.0	496
bta-miR-30b-3p	-23.6	313
bta-miR-30b-3p	-23.1	449
bta-miR-30b-3p	-22.5	16
bta-miR-30b-3p	-22.0	154
bta-miR-30b-3p	-21.7	129
bta-miR-30b-3p	-19.9	79
bta-miR-30b-3p	-19.7	375
bta-miR-30b-3p	-17.9	223
bta-miR-30b-3p	-17.9	411
bta-miR-30b-3p	-17.9	49
bta-miR-30b-3p	-17.2	555
bta-miR-30b-3p	-16.9	348
bta-miR-30b-3p	-16.8	252
bta-miR-30b-3p	-16.2	104
bta-miR-30b-3p	-15.6	174
bta-miR-30b-3p	-15.3	616
bta-miR-30b-3p	-15.3	327
bta-miR-30b-3p	-14.2	651
bta-miR-30b-3p	-13.6	588
bta-miR-30b-3p	-12.5	705
bta-miR-30b-3p	-11.8	5
bta-miR-30b-3p	-11.5	283

bta-miR-30b-3p	-11.2	294
bta-miR-30b-3p	-10.9	486
bta-miR-30b-3p	-10.7	687
bta-miR-30b-3p	-10.6	730
bta-miR-30b-3p	-10.6	145
bta-miR-30b-3p	-10.1	527
bta-miR-31	-25.2	12
bta-miR-31	-24.6	456
bta-miR-31	-24.2	408
bta-miR-31	-23.2	653
bta-miR-31	-20.9	559
bta-miR-31	-20.4	487
bta-miR-31	-20.2	155
bta-miR-31	-20.0	387
bta-miR-31	-20.0	85
bta-miR-31	-19.7	115
bta-miR-31	-18.7	38
bta-miR-31	-18.2	282
bta-miR-31	-18.2	614
bta-miR-31	-17.3	520
bta-miR-31	-16.1	344
bta-miR-31	-15.9	232
bta-miR-31	-15.7	198
bta-miR-31	-14.0	686
bta-miR-31	-13.5	256
bta-miR-31	-13.2	61
bta-miR-31	-12.9	377
bta-miR-31	-12.9	738
bta-miR-31	-12.6	630
bta-miR-31	-12.5	590
bta-miR-31	-12.0	545
bta-miR-31	-11.1	428
bta-miR-31	-10.9	2
bta-miR-34b	-22.6	107
bta-miR-34b	-22.4	8
bta-miR-34b	-22.3	410
bta-miR-34b	-20.5	544
bta-miR-34b	-19.2	67
bta-miR-34b	-19.0	452
bta-miR-34b	-18.6	150
bta-miR-34b	-17.2	170
bta-miR-34b	-16.7	645
bta-miR-34b	-16.1	37
bta-miR-34b	-16.1	576
bta-miR-34b	-16.0	351
bta-miR-34b	-15.8	282
bta-miR-34b	-15.0	516
bta-miR-34b	-14.9	376
bta-miR-34b	-14.9	487
bta-miR-34b	-14.3	692
bta-miR-34b	-13.9	198
bta-miR-34b	-13.9	238
bta-miR-34b	-13.3	737
bta-miR-34b	-13.2	128
bta-miR-34b	-12.9	662
bta-miR-34b	-12.4	263
bta-miR-34b	-12.2	623
bta-miR-34b	-10.9	54
bta-miR-34b	-10.4	311

bta-miR-34b	-10.4	392
bta-miR-106	-23.4	367
bta-miR-106	-20.8	425
bta-miR-106	-20.6	111
bta-miR-106	-20.5	13
bta-miR-106	-20.0	581
bta-miR-106	-19.2	51
bta-miR-106	-19.0	473
bta-miR-106	-18.5	649
bta-miR-106	-18.1	519
bta-miR-106	-16.3	261
bta-miR-106	-15.7	192
bta-miR-106	-15.6	385
bta-miR-106	-14.8	559
bta-miR-106	-14.4	615
bta-miR-106	-14.1	341
bta-miR-106	-13.8	85
bta-miR-106	-13.4	493
bta-miR-106	-13.4	275
bta-miR-106	-13.2	543
bta-miR-106	-12.4	167
bta-miR-106	-11.9	684
bta-miR-106	-11.9	739
bta-miR-106	-11.6	307
bta-miR-106	-11.2	210
bta-miR-106	-11.1	417
bta-miR-106	-11.0	143
bta-miR-106	-11.0	249
bta-miR-106	-10.7	2
bta-miR-106	-10.6	356
bta-miR-107	-27.8	108
bta-miR-107	-22.5	361
bta-miR-107	-22.4	243
bta-miR-107	-20.8	472
bta-miR-107	-20.4	407
bta-miR-107	-20.2	166
bta-miR-107	-20.0	50
bta-miR-107	-19.7	576
bta-miR-107	-18.8	29
bta-miR-107	-18.8	546
bta-miR-107	-18.7	497
bta-miR-107	-18.6	429
bta-miR-107	-17.7	1
bta-miR-107	-17.6	623
bta-miR-107	-17.4	653
bta-miR-107	-15.2	89
bta-miR-107	-15.1	711
bta-miR-107	-13.5	282
bta-miR-107	-13.5	683
bta-miR-107	-13.4	142
bta-miR-107	-13.2	205
bta-miR-107	-13.1	19
bta-miR-107	-12.1	125
bta-miR-107	-11.3	526
bta-miR-107	-10.7	386
bta-miR-107	-10.6	328
bta-miR-107	-10.3	191
bta-miR-107	-10.2	401
bta-miR-10a	-22.2	494

bta-miR-10a	-20.8	586
bta-miR-10a	-20.5	614
bta-miR-10a	-18.4	35
bta-miR-10a	-16.8	702
bta-miR-10a	-16.2	454
bta-miR-10a	-16.0	2
bta-miR-10a	-15.1	403
bta-miR-10a	-14.3	71
bta-miR-10a	-14.1	271
bta-miR-10a	-13.8	106
bta-miR-10a	-13.7	199
bta-miR-10a	-13.6	434
bta-miR-10a	-13.4	382
bta-miR-10a	-13.4	469
bta-miR-10a	-13.2	148
bta-miR-10a	-13.1	654
bta-miR-10a	-12.4	224
bta-miR-10a	-12.3	733
bta-miR-10a	-12.2	344
bta-miR-10a	-11.7	556
bta-miR-10a	-11.0	86
bta-miR-10a	-10.2	258
bta-miR-127	-31.6	482
bta-miR-127	-27.1	407
bta-miR-127	-25.9	65
bta-miR-127	-24.6	119
bta-miR-127	-23.8	429
bta-miR-127	-23.3	198
bta-miR-127	-22.5	166
bta-miR-127	-22.3	8
bta-miR-127	-22.3	243
bta-miR-127	-21.5	698
bta-miR-127	-21.1	603
bta-miR-127	-20.7	572
bta-miR-127	-18.2	622
bta-miR-127	-18.2	650
bta-miR-127	-15.6	29
bta-miR-127	-15.2	371
bta-miR-127	-14.3	50
bta-miR-127	-13.9	555
bta-miR-127	-13.6	331
bta-miR-127	-13.2	308
bta-miR-127	-13.0	515
bta-miR-127	-13.0	737
bta-miR-127	-12.9	97
bta-miR-127	-11.4	392
bta-miR-127	-11.3	472
bta-miR-127	-11.1	507
bta-miR-127	-10.8	154
bta-miR-127	-10.3	282
bta-miR-139	-27.1	346
bta-miR-139	-26.4	477
bta-miR-139	-23.1	628
bta-miR-139	-23.1	415
bta-miR-139	-23.0	13
bta-miR-139	-22.7	66
bta-miR-139	-19.7	504
bta-miR-139	-18.1	595
bta-miR-139	-17.1	267

bta-miR-139	-16.9	373
bta-miR-139	-16.6	737
bta-miR-139	-16.5	560
bta-miR-139	-16.4	220
bta-miR-139	-15.3	172
bta-miR-139	-15.2	112
bta-miR-139	-14.6	449
bta-miR-139	-14.5	39
bta-miR-139	-13.8	545
bta-miR-139	-11.8	527
bta-miR-139	-11.2	202
bta-miR-139	-11.0	155
bta-miR-139	-10.8	1
bta-miR-140	-24.9	416
bta-miR-140	-23.5	81
bta-miR-140	-23.5	660
bta-miR-140	-22.8	461
bta-miR-140	-22.0	618
bta-miR-140	-20.6	342
bta-miR-140	-20.6	497
bta-miR-140	-20.4	564
bta-miR-140	-19.3	247
bta-miR-140	-19.2	98
bta-miR-140	-18.8	14
bta-miR-140	-17.2	378
bta-miR-140	-14.9	541
bta-miR-140	-14.3	596
bta-miR-140	-14.0	311
bta-miR-140	-14.0	741
bta-miR-140	-13.5	209
bta-miR-140	-13.0	60
bta-miR-140	-13.0	167
bta-miR-140	-13.0	684
bta-miR-140	-12.7	276
bta-miR-140	-12.3	2
bta-miR-140	-12.0	133
bta-miR-140	-11.9	117
bta-miR-140	-10.8	327
bta-miR-140	-10.7	155
bta-miR-140	-10.6	408
bta-miR-142	-18.7	517
bta-miR-142	-14.9	280
bta-miR-142	-13.9	632
bta-miR-142	-13.8	412
bta-miR-142	-13.5	376
bta-miR-142	-13.4	584
bta-miR-142	-13.4	659
bta-miR-142	-13.1	544
bta-miR-142	-13.0	81
bta-miR-142	-12.9	11
bta-miR-142	-12.7	358
bta-miR-142	-12.6	63
bta-miR-142	-12.6	559
bta-miR-142	-11.8	241
bta-miR-142	-11.4	185
bta-miR-142	-11.0	40
bta-miR-142	-11.0	618
bta-miR-142	-10.7	741
bta-miR-142	-10.6	697

bta-miR-142	-10.4	266
bta-miR-142	-10.0	678
bta-miR-142*	-19.3	4
bta-miR-142*	-19.1	258
bta-miR-142*	-18.7	191
bta-miR-142*	-17.7	459
bta-miR-142*	-17.4	60
bta-miR-142*	-17.4	161
bta-miR-142*	-16.7	618
bta-miR-142*	-15.9	97
bta-miR-142*	-15.9	401
bta-miR-142*	-15.6	209
bta-miR-142*	-15.6	577
bta-miR-142*	-15.4	342
bta-miR-142*	-14.8	436
bta-miR-142*	-13.8	501
bta-miR-142*	-13.5	368
bta-miR-142*	-13.4	235
bta-miR-142*	-13.1	729
bta-miR-142*	-12.6	34
bta-miR-142*	-12.2	604
bta-miR-142*	-11.7	147
bta-miR-142*	-11.3	384
bta-miR-142*	-11.3	551
bta-miR-142*	-10.6	299
bta-miR-142*	-10.5	639
bta-miR-142*	-10.4	514
bta-miR-142*	-10.3	279
bta-miR-15b	-22.3	554
bta-miR-15b	-22.2	7
bta-miR-15b	-20.7	406
bta-miR-15b	-18.8	531
bta-miR-15b	-18.6	723
bta-miR-15b	-18.2	108
bta-miR-15b	-18.2	347
bta-miR-15b	-17.9	497
bta-miR-15b	-17.8	69
bta-miR-15b	-17.2	644
bta-miR-15b	-16.5	366
bta-miR-15b	-16.1	471
bta-miR-15b	-15.6	619
bta-miR-15b	-15.4	27
bta-miR-15b	-14.4	242
bta-miR-15b	-14.0	293
bta-miR-15b	-13.9	424
bta-miR-15b	-13.0	91
bta-miR-15b	-12.5	169
bta-miR-15b	-12.5	686
bta-miR-15b	-12.3	591
bta-miR-15b	-12.0	143
bta-miR-15b	-11.9	124
bta-miR-15b	-11.5	273
bta-miR-15b	-11.5	339
bta-miR-15b	-11.2	195
bta-miR-181b	-24.1	162
bta-miR-181b	-22.8	340
bta-miR-181b	-22.4	472
bta-miR-181b	-21.4	594
bta-miR-181b	-20.7	509

bta-miR-181b	-20.0	1
bta-miR-181b	-19.6	424
bta-miR-181b	-18.8	29
bta-miR-181b	-18.7	50
bta-miR-181b	-18.7	361
bta-miR-181b	-18.5	563
bta-miR-181b	-17.9	185
bta-miR-181b	-17.8	108
bta-miR-181b	-17.4	406
bta-miR-181b	-16.9	623
bta-miR-181b	-16.8	324
bta-miR-181b	-16.8	208
bta-miR-181b	-16.8	243
bta-miR-181b	-16.4	260
bta-miR-181b	-15.7	142
bta-miR-181b	-14.2	120
bta-miR-181b	-14.2	135
bta-miR-181b	-14.0	274
bta-miR-181b	-13.6	701
bta-miR-181b	-13.5	317
bta-miR-181b	-13.0	73
bta-miR-181b	-12.8	739
bta-miR-181b	-12.2	455
bta-miR-181b	-12.2	306
bta-miR-181b	-12.0	540
bta-miR-181b	-10.1	386
bta-miR-181b	-10.1	666
bta-miR-193a-5p	-23.5	546
bta-miR-193a-5p	-23.1	23
bta-miR-193a-5p	-22.7	395
bta-miR-193a-5p	-22.2	354
bta-miR-193a-5p	-21.8	167
bta-miR-193a-5p	-21.3	473
bta-miR-193a-5p	-20.1	299
bta-miR-193a-5p	-20.1	190
bta-miR-193a-5p	-20.0	118
bta-miR-193a-5p	-19.7	53
bta-miR-193a-5p	-19.5	2
bta-miR-193a-5p	-19.4	577
bta-miR-193a-5p	-19.3	137
bta-miR-193a-5p	-18.6	235
bta-miR-193a-5p	-18.3	256
bta-miR-193a-5p	-17.4	488
bta-miR-193a-5p	-17.3	428
bta-miR-193a-5p	-17.0	89
bta-miR-193a-5p	-16.8	639
bta-miR-193a-5p	-16.3	618
bta-miR-193a-5p	-15.9	664
bta-miR-193a-5p	-15.7	211
bta-miR-193a-5p	-15.3	565
bta-miR-193a-5p	-14.8	323
bta-miR-193a-5p	-14.7	694
bta-miR-193a-5p	-14.7	337
bta-miR-193a-5p	-13.9	377
bta-miR-193a-5p	-13.8	606
bta-miR-193a-5p	-13.5	734
bta-miR-193a-5p	-12.4	512
bta-miR-193a-5p	-12.2	461
bta-miR-193a-5p	-11.2	286

bta-miR-193a-5p	-10.3	744
bta-miR-193a-3p	-28.2	13
bta-miR-193a-3p	-26.5	66
bta-miR-193a-3p	-25.2	415
bta-miR-193a-3p	-23.2	112
bta-miR-193a-3p	-22.2	606
bta-miR-193a-3p	-19.9	477
bta-miR-193a-3p	-17.2	364
bta-miR-193a-3p	-17.1	267
bta-miR-193a-3p	-17.1	449
bta-miR-193a-3p	-16.3	216
bta-miR-193a-3p	-15.6	560
bta-miR-193a-3p	-15.1	649
bta-miR-193a-3p	-14.0	176
bta-miR-193a-3p	-13.9	41
bta-miR-193a-3p	-13.8	154
bta-miR-193a-3p	-13.7	520
bta-miR-193a-3p	-12.7	739
bta-miR-193a-3p	-12.1	595
bta-miR-193a-3p	-11.4	2
bta-miR-193a-3p	-11.3	96
bta-miR-193a-3p	-11.0	166
bta-miR-193a-3p	-10.9	683
bta-miR-193a-3p	-10.7	241
bta-miR-193a-3p	-10.1	706
bta-miR-20b	-26.3	367
bta-miR-20b	-23.0	13
bta-miR-20b	-23.0	425
bta-miR-20b	-22.4	537
bta-miR-20b	-21.8	581
bta-miR-20b	-21.4	649
bta-miR-20b	-20.3	490
bta-miR-20b	-19.6	111
bta-miR-20b	-19.1	261
bta-miR-20b	-18.3	602
bta-miR-20b	-18.2	51
bta-miR-20b	-17.9	473
bta-miR-20b	-16.7	402
bta-miR-20b	-16.5	192
bta-miR-20b	-15.7	341
bta-miR-20b	-15.3	85
bta-miR-20b	-14.6	275
bta-miR-20b	-14.1	167
bta-miR-20b	-13.9	559
bta-miR-20b	-13.3	385
bta-miR-20b	-13.3	526
bta-miR-20b	-13.1	210
bta-miR-20b	-12.5	126
bta-miR-20b	-12.4	449
bta-miR-20b	-11.9	684
bta-miR-20b	-11.9	739
bta-miR-20b	-11.7	235
bta-miR-20b	-11.6	307
bta-miR-20b	-10.8	2
bta-miR-20b	-10.6	631
bta-miR-215	-25.2	3
bta-miR-215	-18.2	251
bta-miR-215	-17.7	566
bta-miR-215	-15.5	60

bta-miR-215	-15.2	642
bta-miR-215	-14.6	491
bta-miR-215	-14.2	215
bta-miR-215	-14.0	25
bta-miR-215	-13.3	395
bta-miR-215	-13.3	543
bta-miR-215	-13.2	291
bta-miR-215	-13.1	82
bta-miR-215	-12.8	614
bta-miR-215	-12.8	476
bta-miR-215	-12.7	344
bta-miR-215	-11.6	583
bta-miR-215	-11.5	666
bta-miR-215	-11.2	386
bta-miR-215	-10.9	684
bta-miR-215	-10.7	436
bta-miR-215	-10.6	524
bta-miR-215	-10.1	695
bta-miR-218	-19.5	353
bta-miR-218	-19.0	587
bta-miR-218	-18.6	22
bta-miR-218	-18.1	459
bta-miR-218	-16.6	106
bta-miR-218	-16.5	8
bta-miR-218	-16.4	412
bta-miR-218	-16.2	654
bta-miR-218	-15.8	494
bta-miR-218	-15.3	66
bta-miR-218	-15.3	220
bta-miR-218	-14.9	172
bta-miR-218	-14.7	266
bta-miR-218	-13.3	201
bta-miR-218	-12.5	616
bta-miR-218	-12.3	435
bta-miR-218	-12.2	544
bta-miR-218	-11.7	739
bta-miR-218	-11.3	566
bta-miR-218	-10.8	148
bta-miR-218	-10.1	383
bta-miR-218	-10.0	702
bta-miR-30e-5p	-23.1	456
bta-miR-30e-5p	-21.2	166
bta-miR-30e-5p	-20.7	512
bta-miR-30e-5p	-20.1	5
bta-miR-30e-5p	-19.7	586
bta-miR-30e-5p	-19.2	72
bta-miR-30e-5p	-18.6	205
bta-miR-30e-5p	-18.2	282
bta-miR-30e-5p	-17.8	403
bta-miR-30e-5p	-16.4	358
bta-miR-30e-5p	-16.4	559
bta-miR-30e-5p	-16.0	29
bta-miR-30e-5p	-15.7	435
bta-miR-30e-5p	-15.3	390
bta-miR-30e-5p	-15.3	115
bta-miR-30e-5p	-14.6	539
bta-miR-30e-5p	-14.6	236
bta-miR-30e-5p	-14.3	653
bta-miR-30e-5p	-14.2	626

bta-miR-30e-5p	-13.7	263
bta-miR-30e-5p	-12.8	637
bta-miR-30e-5p	-12.5	98
bta-miR-30e-5p	-12.4	58
bta-miR-30e-5p	-12.3	499
bta-miR-30e-5p	-11.9	143
bta-miR-30e-5p	-11.8	184
bta-miR-30e-5p	-11.7	342
bta-miR-30e-5p	-11.6	698
bta-miR-30e-5p	-10.5	222
bta-miR-30e-5p	-10.4	316
bta-miR-30e-5p	-10.4	127
bta-miR-30e-5p	-10.1	713
bta-miR-345-5p	-26.5	407
bta-miR-345-5p	-25.5	494
bta-miR-345-5p	-24.7	605
bta-miR-345-5p	-23.5	108
bta-miR-345-5p	-22.7	65
bta-miR-345-5p	-22.2	472
bta-miR-345-5p	-21.2	369
bta-miR-345-5p	-21.1	4
bta-miR-345-5p	-19.5	205
bta-miR-345-5p	-19.0	622
bta-miR-345-5p	-18.9	435
bta-miR-345-5p	-17.6	270
bta-miR-345-5p	-17.0	653
bta-miR-345-5p	-16.9	166
bta-miR-345-5p	-16.1	584
bta-miR-345-5p	-14.6	739
bta-miR-345-5p	-13.9	37
bta-miR-345-5p	-13.3	527
bta-miR-345-5p	-13.2	559
bta-miR-345-5p	-12.2	384
bta-miR-345-5p	-12.0	350
bta-miR-345-5p	-11.5	698
bta-miR-345-5p	-10.6	150
bta-miR-345-5p	-10.4	263
bta-miR-345-3p	-26.9	236
bta-miR-345-3p	-23.6	98
bta-miR-345-3p	-22.9	461
bta-miR-345-3p	-21.3	127
bta-miR-345-3p	-21.3	421
bta-miR-345-3p	-21.2	514
bta-miR-345-3p	-19.8	164
bta-miR-345-3p	-19.6	356
bta-miR-345-3p	-19.6	579
bta-miR-345-3p	-19.6	52
bta-miR-345-3p	-19.4	186
bta-miR-345-3p	-18.7	317
bta-miR-345-3p	-18.6	27
bta-miR-345-3p	-18.4	622
bta-miR-345-3p	-17.9	136
bta-miR-345-3p	-17.2	401
bta-miR-345-3p	-16.6	71
bta-miR-345-3p	-16.5	9
bta-miR-345-3p	-16.0	209
bta-miR-345-3p	-14.6	635
bta-miR-345-3p	-14.3	707
bta-miR-345-3p	-13.8	493

bta-miR-345-3p	-13.5	327
bta-miR-345-3p	-13.4	567
bta-miR-345-3p	-12.9	737
bta-miR-345-3p	-12.7	283
bta-miR-345-3p	-12.6	391
bta-miR-345-3p	-11.9	331
bta-miR-345-3p	-11.8	85
bta-miR-345-3p	-11.7	305
bta-miR-345-3p	-11.4	650
bta-miR-345-3p	-11.0	542
bta-miR-345-3p	-10.3	154
bta-miR-345-3p	-10.2	444
bta-miR-345-3p	-10.2	115
bta-miR-345-3p	-10.1	41
bta-miR-345-3p	-10.1	607
bta-miR-369-5p	-18.3	404
bta-miR-369-5p	-16.0	13
bta-miR-369-5p	-15.9	730
bta-miR-369-5p	-15.7	62
bta-miR-369-5p	-14.5	301
bta-miR-369-5p	-14.4	614
bta-miR-369-5p	-14.3	450
bta-miR-369-5p	-14.1	356
bta-miR-369-5p	-14.0	584
bta-miR-369-5p	-13.6	569
bta-miR-369-5p	-12.8	81
bta-miR-369-5p	-12.7	497
bta-miR-369-5p	-12.5	600
bta-miR-369-5p	-11.9	158
bta-miR-369-5p	-11.8	335
bta-miR-369-5p	-11.5	266
bta-miR-369-5p	-10.9	220
bta-miR-369-5p	-10.8	376
bta-miR-369-5p	-10.8	653
bta-miR-369-5p	-10.7	550
bta-miR-369-5p	-10.5	176
bta-miR-369-5p	-10.2	477
bta-miR-369-5p	-10.1	102
bta-miR-369-5p	-10.0	199
bta-miR-369-3p	-18.0	499
bta-miR-369-3p	-15.2	574
bta-miR-369-3p	-15.2	16
bta-miR-369-3p	-13.9	645
bta-miR-369-3p	-13.4	542
bta-miR-369-3p	-13.2	101
bta-miR-369-3p	-13.2	226
bta-miR-369-3p	-13.0	44
bta-miR-369-3p	-12.8	449
bta-miR-369-3p	-12.1	663
bta-miR-369-3p	-12.1	407
bta-miR-369-3p	-11.8	423
bta-miR-369-3p	-11.7	349
bta-miR-369-3p	-11.6	175
bta-miR-369-3p	-11.5	239
bta-miR-369-3p	-11.3	479
bta-miR-369-3p	-11.2	598
bta-miR-369-3p	-11.1	201
bta-miR-369-3p	-10.9	371
bta-miR-369-3p	-10.7	165

bta-miR-369-3p	-10.6	125
bta-miR-369-3p	-10.6	340
bta-miR-369-3p	-10.5	557
bta-miR-369-3p	-10.4	114
bta-miR-380-5p	-23.4	654
bta-miR-380-5p	-23.1	379
bta-miR-380-5p	-22.5	20
bta-miR-380-5p	-19.8	525
bta-miR-380-5p	-18.7	493
bta-miR-380-5p	-17.7	415
bta-miR-380-5p	-17.5	252
bta-miR-380-5p	-16.7	457
bta-miR-380-5p	-16.6	631
bta-miR-380-5p	-16.3	66
bta-miR-380-5p	-16.3	206
bta-miR-380-5p	-15.6	109
bta-miR-380-5p	-14.8	606
bta-miR-380-5p	-13.8	567
bta-miR-380-5p	-13.2	359
bta-miR-380-5p	-12.8	584
bta-miR-380-5p	-12.8	167
bta-miR-380-5p	-12.4	283
bta-miR-380-5p	-12.1	3
bta-miR-380-5p	-11.7	741
bta-miR-380-5p	-11.3	697
bta-miR-380-5p	-10.7	336
bta-miR-380-5p	-10.3	88
bta-miR-380-3p	-20.1	366
bta-miR-380-3p	-19.3	478
bta-miR-380-3p	-18.5	418
bta-miR-380-3p	-18.1	11
bta-miR-380-3p	-17.4	108
bta-miR-380-3p	-17.1	80
bta-miR-380-3p	-16.9	617
bta-miR-380-3p	-16.4	497
bta-miR-380-3p	-16.0	129
bta-miR-380-3p	-15.7	266
bta-miR-380-3p	-15.2	737
bta-miR-380-3p	-15.1	660
bta-miR-380-3p	-14.7	228
bta-miR-380-3p	-14.1	563
bta-miR-380-3p	-13.9	448
bta-miR-380-3p	-13.9	25
bta-miR-380-3p	-13.5	589
bta-miR-380-3p	-12.8	351
bta-miR-380-3p	-12.6	172
bta-miR-380-3p	-11.6	66
bta-miR-380-3p	-11.5	293
bta-miR-380-3p	-10.7	709
bta-miR-380-3p	-10.5	518
bta-miR-380-3p	-10.2	644
bta-miR-380-3p	-10.1	313
bta-miR-380-3p	-10.0	51
bta-miR-380-3p	-10.0	405
bta-miR-487a	-25.1	420
bta-miR-487a	-24.7	13
bta-miR-487a	-21.7	568
bta-miR-487a	-20.9	250
bta-miR-487a	-19.6	82

bta-miR-487a	-19.3	616
bta-miR-487a	-17.9	356
bta-miR-487a	-17.6	708
bta-miR-487a	-17.3	39
bta-miR-487a	-16.8	231
bta-miR-487a	-16.3	498
bta-miR-487a	-15.6	644
bta-miR-487a	-14.8	454
bta-miR-487a	-14.7	477
bta-miR-487a	-14.5	387
bta-miR-487a	-14.4	112
bta-miR-487a	-13.9	739
bta-miR-487a	-13.9	542
bta-miR-487a	-13.1	185
bta-miR-487a	-12.0	330
bta-miR-487a	-11.8	1
bta-miR-487a	-11.7	280
bta-miR-487a	-11.6	199
bta-miR-487a	-11.3	97
bta-miR-487a	-10.6	140
bta-miR-487a	-10.6	155
bta-miR-487a	-10.6	240
bta-miR-487a	-10.0	167
bta-miR-545*	-19.5	97
bta-miR-545*	-17.3	484
bta-miR-545*	-16.5	409
bta-miR-545*	-16.0	572
bta-miR-545*	-15.7	343
bta-miR-545*	-15.7	459
bta-miR-545*	-15.4	715
bta-miR-545*	-15.1	514
bta-miR-545*	-14.7	169
bta-miR-545*	-14.3	235
bta-miR-545*	-14.2	200
bta-miR-545*	-13.6	4
bta-miR-545*	-13.0	596
bta-miR-545*	-12.6	645
bta-miR-545*	-12.5	681
bta-miR-545*	-12.2	143
bta-miR-545*	-11.8	53
bta-miR-545*	-11.7	368
bta-miR-545*	-11.4	258
bta-miR-545*	-11.2	384
bta-miR-545*	-11.0	76
bta-miR-545*	-10.9	495
bta-miR-545*	-10.6	20
bta-miR-545	-16.1	13
bta-miR-545	-14.9	603
bta-miR-545	-14.5	71
bta-miR-545	-13.0	489
bta-miR-545	-12.5	408
bta-miR-545	-12.2	550
bta-miR-545	-12.0	369
bta-miR-545	-11.9	516
bta-miR-545	-11.4	326
bta-miR-545	-11.1	654
bta-miR-545	-10.9	732
bta-miR-545	-10.9	148
bta-miR-545	-10.6	436

bta-miR-545	-10.5	571
bta-miR-545	-10.4	351
bta-miR-545	-10.0	624
bta-miR-92	-31.0	10
bta-miR-92	-28.9	602
bta-miR-92	-26.0	358
bta-miR-92	-24.8	481
bta-miR-92	-24.2	411
bta-miR-92	-22.0	66
bta-miR-92	-20.5	114
bta-miR-92	-19.6	499
bta-miR-92	-19.6	442
bta-miR-92	-18.2	264
bta-miR-92	-17.7	575
bta-miR-92	-17.2	623
bta-miR-92	-15.9	154
bta-miR-92	-14.8	35
bta-miR-92	-14.0	199
bta-miR-92	-13.4	176
bta-miR-92	-13.3	560
bta-miR-92	-13.3	596
bta-miR-92	-11.9	737
bta-miR-92	-11.7	462
bta-miR-92	-11.7	654
bta-miR-92	-11.1	241
bta-miR-92	-10.9	86
bta-miR-92	-10.7	106
bta-miR-92	-10.5	304
bta-miR-92	-10.2	341
bta-miR-98	-19.0	106
bta-miR-98	-18.5	171
bta-miR-98	-16.9	494
bta-miR-98	-16.8	372
bta-miR-98	-16.7	410
bta-miR-98	-16.3	264
bta-miR-98	-16.2	301
bta-miR-98	-15.6	65
bta-miR-98	-15.6	697
bta-miR-98	-14.9	729
bta-miR-98	-14.4	12
bta-miR-98	-14.4	149
bta-miR-98	-14.3	560
bta-miR-98	-14.1	122
bta-miR-98	-13.9	355
bta-miR-98	-13.4	199
bta-miR-98	-13.3	516
bta-miR-98	-13.0	37
bta-miR-98	-12.8	653
bta-miR-98	-12.6	463
bta-miR-98	-12.4	584
bta-miR-98	-12.2	623
bta-miR-98	-10.9	230
bta-let-7d	-21.8	414
bta-let-7d	-21.1	375
bta-let-7d	-20.4	107
bta-let-7d	-19.6	554
bta-let-7d	-19.2	456
bta-let-7d	-18.0	656
bta-let-7d	-18.0	42

bta-let-7d	-17.8	244
bta-let-7d	-17.8	266
bta-let-7d	-17.7	697
bta-let-7d	-17.6	582
bta-let-7d	-17.5	16
bta-let-7d	-17.4	359
bta-let-7d	-17.0	487
bta-let-7d	-16.9	293
bta-let-7d	-16.2	199
bta-let-7d	-16.1	62
bta-let-7d	-15.8	183
bta-let-7d	-15.1	81
bta-let-7d	-15.0	524
bta-let-7d	-14.8	618
bta-let-7d	-14.4	729
bta-let-7d	-13.5	125
bta-let-7d	-13.3	402
bta-let-7d	-12.8	155
bta-let-7d	-11.4	336
bta-miR-124a	-22.7	503
bta-miR-124a	-22.4	413
bta-miR-124a	-20.0	155
bta-miR-124a	-19.7	39
bta-miR-124a	-19.4	17
bta-miR-124a	-19.0	654
bta-miR-124a	-18.9	370
bta-miR-124a	-18.8	542
bta-miR-124a	-18.8	605
bta-miR-124a	-18.6	115
bta-miR-124a	-18.4	576
bta-miR-124a	-18.2	341
bta-miR-124a	-18.0	464
bta-miR-124a	-17.9	85
bta-miR-124a	-17.7	488
bta-miR-124a	-17.1	177
bta-miR-124a	-16.5	685
bta-miR-124a	-16.0	231
bta-miR-124a	-15.7	299
bta-miR-124a	-15.3	270
bta-miR-124a	-14.6	400
bta-miR-124a	-13.9	1
bta-miR-124a	-13.7	594
bta-miR-124a	-13.4	739
bta-miR-124a	-13.2	144
bta-miR-124a	-12.0	199
bta-miR-124a	-11.7	708
bta-miR-124a	-11.6	315
bta-miR-124a	-11.6	532
bta-miR-124a	-11.5	631
bta-miR-124a	-11.4	565
bta-miR-124a	-11.3	384
bta-miR-124a	-10.7	326
bta-miR-124a	-10.4	448
bta-miR-132	-25.8	408
bta-miR-132	-22.6	570
bta-miR-132	-20.3	9
bta-miR-132	-20.3	366
bta-miR-132	-20.1	72
bta-miR-132	-19.8	482

bta-miR-132	-19.7	109
bta-miR-132	-18.9	525
bta-miR-132	-18.5	605
bta-miR-132	-18.4	424
bta-miR-132	-16.9	51
bta-miR-132	-15.6	167
bta-miR-132	-15.5	341
bta-miR-132	-15.2	738
bta-miR-132	-14.6	204
bta-miR-132	-14.3	225
bta-miR-132	-13.9	635
bta-miR-132	-13.2	457
bta-miR-132	-12.5	143
bta-miR-132	-12.0	34
bta-miR-132	-11.9	261
bta-miR-132	-11.4	551
bta-miR-132	-11.2	661
bta-miR-132	-10.7	277
bta-miR-132	-10.3	1
bta-miR-132	-10.1	595
bta-miR-132	-10.0	515
bta-miR-138	-24.5	114
bta-miR-138	-24.5	155
bta-miR-138	-23.3	51
bta-miR-138	-23.1	576
bta-miR-138	-22.7	482
bta-miR-138	-22.0	85
bta-miR-138	-21.4	361
bta-miR-138	-21.3	12
bta-miR-138	-21.0	424
bta-miR-138	-19.1	402
bta-miR-138	-18.6	499
bta-miR-138	-18.5	623
bta-miR-138	-17.2	308
bta-miR-138	-16.6	344
bta-miR-138	-15.8	659
bta-miR-138	-15.7	177
bta-miR-138	-15.4	270
bta-miR-138	-15.2	242
bta-miR-138	-14.7	710
bta-miR-138	-14.4	551
bta-miR-138	-13.3	141
bta-miR-138	-13.2	382
bta-miR-138	-12.7	215
bta-miR-138	-12.2	605
bta-miR-138	-11.9	414
bta-miR-138	-11.7	291
bta-miR-138	-11.5	72
bta-miR-138	-11.0	467
bta-miR-138	-11.0	681
bta-miR-138	-11.0	527
bta-miR-148b	-22.6	66
bta-miR-148b	-19.7	469
bta-miR-148b	-19.6	573
bta-miR-148b	-19.5	535
bta-miR-148b	-18.5	415
bta-miR-148b	-17.1	606
bta-miR-148b	-17.0	5
bta-miR-148b	-17.0	353

bta-miR-148b	-15.6	263
bta-miR-148b	-15.4	172
bta-miR-148b	-14.9	517
bta-miR-148b	-14.6	654
bta-miR-148b	-14.0	733
bta-miR-148b	-13.4	384
bta-miR-148b	-13.2	552
bta-miR-148b	-12.6	106
bta-miR-148b	-11.9	36
bta-miR-148b	-11.8	226
bta-miR-148b	-11.4	280
bta-miR-148b	-11.3	206
bta-miR-148b	-11.0	435
bta-miR-148b	-10.9	633
bta-miR-148b	-10.6	59
bta-miR-148b	-10.3	691
bta-miR-17-5p	-23.4	367
bta-miR-17-5p	-22.9	13
bta-miR-17-5p	-21.9	420
bta-miR-17-5p	-20.7	579
bta-miR-17-5p	-20.7	488
bta-miR-17-5p	-20.6	111
bta-miR-17-5p	-20.4	649
bta-miR-17-5p	-19.5	537
bta-miR-17-5p	-18.3	189
bta-miR-17-5p	-18.0	51
bta-miR-17-5p	-17.6	457
bta-miR-17-5p	-17.4	86
bta-miR-17-5p	-16.7	560
bta-miR-17-5p	-16.3	261
bta-miR-17-5p	-15.9	385
bta-miR-17-5p	-14.5	618
bta-miR-17-5p	-14.3	341
bta-miR-17-5p	-13.4	739
bta-miR-17-5p	-13.2	138
bta-miR-17-5p	-13.2	526
bta-miR-17-5p	-12.8	275
bta-miR-17-5p	-12.4	307
bta-miR-17-5p	-12.4	167
bta-miR-17-5p	-12.4	668
bta-miR-17-5p	-11.2	210
bta-miR-17-5p	-11.0	249
bta-miR-17-5p	-10.7	2
bta-miR-17-5p	-10.1	705
bta-miR-17-3p	-22.6	35
bta-miR-17-3p	-21.8	480
bta-miR-17-3p	-21.4	573
bta-miR-17-3p	-20.5	355
bta-miR-17-3p	-19.3	408
bta-miR-17-3p	-18.7	517
bta-miR-17-3p	-17.6	106
bta-miR-17-3p	-17.1	172
bta-miR-17-3p	-17.0	631
bta-miR-17-3p	-16.8	70
bta-miR-17-3p	-16.7	544
bta-miR-17-3p	-16.2	425
bta-miR-17-3p	-15.9	151
bta-miR-17-3p	-15.9	558
bta-miR-17-3p	-15.7	257

bta-miR-17-3p	-15.5	3
bta-miR-17-3p	-15.0	654
bta-miR-17-3p	-14.8	394
bta-miR-17-3p	-14.3	278
bta-miR-17-3p	-14.0	596
bta-miR-17-3p	-13.4	86
bta-miR-17-3p	-13.2	668
bta-miR-17-3p	-12.6	240
bta-miR-17-3p	-12.5	202
bta-miR-17-3p	-11.9	122
bta-miR-17-3p	-11.7	531
bta-miR-17-3p	-11.6	453
bta-miR-17-3p	-11.1	473
bta-miR-17-3p	-11.1	506
bta-miR-17-3p	-10.9	22
bta-miR-17-3p	-10.7	693
bta-miR-17-3p	-10.6	739
bta-miR-181c	-25.2	1
bta-miR-181c	-21.9	594
bta-miR-181c	-19.3	71
bta-miR-181c	-18.6	406
bta-miR-181c	-17.1	558
bta-miR-181c	-17.1	734
bta-miR-181c	-16.1	361
bta-miR-181c	-15.5	622
bta-miR-181c	-15.4	255
bta-miR-181c	-15.3	30
bta-miR-181c	-15.2	154
bta-miR-181c	-15.1	425
bta-miR-181c	-14.9	481
bta-miR-181c	-14.6	520
bta-miR-181c	-14.0	129
bta-miR-181c	-14.0	188
bta-miR-181c	-14.0	334
bta-miR-181c	-13.9	648
bta-miR-181c	-13.4	443
bta-miR-181c	-13.0	273
bta-miR-181c	-12.9	211
bta-miR-181c	-12.9	455
bta-miR-181c	-12.5	59
bta-miR-181c	-12.3	114
bta-miR-181c	-12.3	695
bta-miR-181c	-11.8	313
bta-miR-181c	-10.9	352
bta-miR-181c	-10.8	49
bta-miR-181c	-10.8	145
bta-miR-181c	-10.3	229
bta-miR-181c	-10.1	326
bta-miR-181c	-10.0	666
bta-miR-186	-25.6	166
bta-miR-186	-24.3	623
bta-miR-186	-23.4	429
bta-miR-186	-19.5	605
bta-miR-186	-18.9	205
bta-miR-186	-18.8	472
bta-miR-186	-18.2	270
bta-miR-186	-17.2	29
bta-miR-186	-17.2	234
bta-miR-186	-16.7	108

bta-miR-186	-16.7	50
bta-miR-186	-16.2	361
bta-miR-186	-14.3	330
bta-miR-186	-14.3	97
bta-miR-186	-13.9	308
bta-miR-186	-13.9	4
bta-miR-186	-13.6	407
bta-miR-186	-13.2	566
bta-miR-186	-12.5	669
bta-miR-186	-12.3	344
bta-miR-186	-12.3	499
bta-miR-186	-11.7	192
bta-miR-186	-10.9	698
bta-miR-186	-10.6	596
bta-miR-186	-10.1	324
bta-miR-186	-10.0	78
bta-miR-191	-23.8	617
bta-miR-191	-23.1	64
bta-miR-191	-21.8	410
bta-miR-191	-21.6	495
bta-miR-191	-19.4	453
bta-miR-191	-18.5	165
bta-miR-191	-18.1	559
bta-miR-191	-17.9	115
bta-miR-191	-17.8	696
bta-miR-191	-17.2	278
bta-miR-191	-16.9	12
bta-miR-191	-16.5	472
bta-miR-191	-16.3	586
bta-miR-191	-16.2	653
bta-miR-191	-15.1	219
bta-miR-191	-14.7	742
bta-miR-191	-14.6	516
bta-miR-191	-13.6	682
bta-miR-191	-13.3	243
bta-miR-191	-12.7	603
bta-miR-191	-12.3	307
bta-miR-191	-12.3	384
bta-miR-191	-11.4	548
bta-miR-191	-10.8	342
bta-miR-191	-10.3	358
bta-miR-191	-10.3	448
bta-miR-191	-10.1	198
bta-miR-191	-10.0	1
bta-miR-192	-22.4	3
bta-miR-192	-19.5	85
bta-miR-192	-19.0	413
bta-miR-192	-18.3	582
bta-miR-192	-18.1	250
bta-miR-192	-18.0	40
bta-miR-192	-17.7	560
bta-miR-192	-17.5	605
bta-miR-192	-16.7	115
bta-miR-192	-16.0	477
bta-miR-192	-15.3	632
bta-miR-192	-14.9	155
bta-miR-192	-14.7	432
bta-miR-192	-14.6	369
bta-miR-192	-14.1	216

bta-miR-192	-14.1	740
bta-miR-192	-13.1	72
bta-miR-192	-12.4	524
bta-miR-192	-12.2	497
bta-miR-192	-11.9	292
bta-miR-192	-11.5	185
bta-miR-192	-11.3	455
bta-miR-192	-11.0	623
bta-miR-192	-10.4	667
bta-miR-192	-10.2	548
bta-miR-199b	-22.8	1
bta-miR-199b	-22.1	393
bta-miR-199b	-21.9	600
bta-miR-199b	-20.4	65
bta-miR-199b	-20.4	445
bta-miR-199b	-18.3	92
bta-miR-199b	-16.8	494
bta-miR-199b	-16.6	198
bta-miR-199b	-16.5	242
bta-miR-199b	-16.3	352
bta-miR-199b	-16.1	650
bta-miR-199b	-15.4	170
bta-miR-199b	-15.2	734
bta-miR-199b	-14.4	420
bta-miR-199b	-14.3	372
bta-miR-199b	-14.1	559
bta-miR-199b	-12.7	622
bta-miR-199b	-11.9	577
bta-miR-199b	-11.6	221
bta-miR-199b	-11.0	149
bta-miR-199b	-11.0	516
bta-miR-199b	-10.7	281
bta-miR-199b	-10.6	28
bta-miR-199b	-10.4	128
bta-miR-199b	-10.3	482
bta-miR-199b	-10.1	661
bta-miR-199b	-10.0	692
bta-miR-200a	-22.4	352
bta-miR-200a	-20.4	1
bta-miR-200a	-19.4	592
bta-miR-200a	-18.8	383
bta-miR-200a	-18.2	472
bta-miR-200a	-17.7	227
bta-miR-200a	-17.4	635
bta-miR-200a	-17.0	22
bta-miR-200a	-16.8	126
bta-miR-200a	-16.1	500
bta-miR-200a	-15.8	65
bta-miR-200a	-15.6	255
bta-miR-200a	-14.9	535
bta-miR-200a	-14.4	565
bta-miR-200a	-13.8	85
bta-miR-200a	-13.6	188
bta-miR-200a	-13.6	415
bta-miR-200a	-13.5	738
bta-miR-200a	-13.3	613
bta-miR-200a	-12.4	454
bta-miR-200a	-12.1	149
bta-miR-200a	-11.9	206

bta-miR-200a	-11.8	436
bta-miR-200a	-11.4	667
bta-miR-200a	-11.0	109
bta-miR-200a	-10.7	308
bta-miR-200a	-10.4	118
bta-miR-200c	-23.4	5
bta-miR-200c	-21.6	342
bta-miR-200c	-21.1	52
bta-miR-200c	-19.3	572
bta-miR-200c	-19.1	467
bta-miR-200c	-19.0	409
bta-miR-200c	-18.7	98
bta-miR-200c	-18.5	514
bta-miR-200c	-18.0	164
bta-miR-200c	-17.5	247
bta-miR-200c	-16.3	639
bta-miR-200c	-16.2	295
bta-miR-200c	-15.2	143
bta-miR-200c	-14.5	618
bta-miR-200c	-14.4	23
bta-miR-200c	-14.3	459
bta-miR-200c	-14.2	428
bta-miR-200c	-14.0	207
bta-miR-200c	-13.6	317
bta-miR-200c	-13.4	534
bta-miR-200c	-13.3	699
bta-miR-200c	-12.6	182
bta-miR-200c	-12.5	118
bta-miR-200c	-11.9	391
bta-miR-200c	-11.7	715
bta-miR-200c	-11.4	270
bta-miR-200c	-11.2	85
bta-miR-200c	-11.2	275
bta-miR-200c	-11.0	564
bta-miR-200c	-10.5	666
bta-miR-200c	-10.0	72
bta-miR-210	-30.9	107
bta-miR-210	-27.3	559
bta-miR-210	-25.7	472
bta-miR-210	-25.3	72
bta-miR-210	-24.9	410
bta-miR-210	-23.6	37
bta-miR-210	-22.0	165
bta-miR-210	-21.8	2
bta-miR-210	-20.9	243
bta-miR-210	-20.8	583
bta-miR-210	-20.2	361
bta-miR-210	-16.7	277
bta-miR-210	-16.4	637
bta-miR-210	-15.4	342
bta-miR-210	-14.2	137
bta-miR-210	-14.0	516
bta-miR-210	-14.0	742
bta-miR-210	-13.8	228
bta-miR-210	-13.6	207
bta-miR-210	-13.1	19
bta-miR-210	-13.1	543
bta-miR-210	-12.5	444
bta-miR-210	-12.4	617

bta-miR-210	-12.1	660
bta-miR-210	-11.8	393
bta-miR-210	-11.5	311
bta-miR-210	-11.4	692
bta-miR-210	-11.0	29
bta-miR-214	-30.6	560
bta-miR-214	-27.7	413
bta-miR-214	-27.6	1
bta-miR-214	-24.8	86
bta-miR-214	-24.0	364
bta-miR-214	-22.4	473
bta-miR-214	-22.1	529
bta-miR-214	-19.9	51
bta-miR-214	-18.2	656
bta-miR-214	-18.0	30
bta-miR-214	-17.5	244
bta-miR-214	-17.0	624
bta-miR-214	-16.5	511
bta-miR-214	-15.4	391
bta-miR-214	-15.2	283
bta-miR-214	-14.7	685
bta-miR-214	-14.6	167
bta-miR-214	-13.5	119
bta-miR-214	-13.2	739
bta-miR-214	-12.9	585
bta-miR-214	-12.2	206
bta-miR-214	-12.1	337
bta-miR-214	-11.8	457
bta-miR-214	-11.1	138
bta-miR-214	-10.7	431
bta-miR-214	-10.3	408
bta-miR-22-5p	-24.1	556
bta-miR-22-5p	-22.6	423
bta-miR-22-5p	-22.2	349
bta-miR-22-5p	-21.7	203
bta-miR-22-5p	-21.1	454
bta-miR-22-5p	-20.6	82
bta-miR-22-5p	-20.4	114
bta-miR-22-5p	-20.0	615
bta-miR-22-5p	-19.7	404
bta-miR-22-5p	-19.4	152
bta-miR-22-5p	-18.3	265
bta-miR-22-5p	-16.7	11
bta-miR-22-5p	-16.6	231
bta-miR-22-5p	-16.6	585
bta-miR-22-5p	-16.4	48
bta-miR-22-5p	-15.5	375
bta-miR-22-5p	-15.2	491
bta-miR-22-5p	-14.5	652
bta-miR-22-5p	-14.4	734
bta-miR-22-5p	-13.3	695
bta-miR-22-5p	-12.5	307
bta-miR-22-5p	-11.9	2
bta-miR-22-5p	-11.1	520
bta-miR-22-5p	-10.9	331
bta-miR-22-5p	-10.9	249
bta-miR-22-5p	-10.8	136
bta-miR-22-5p	-10.8	185
bta-miR-22-5p	-10.7	195

bta-miR-22-5p	-10.3	445
bta-miR-22-3p	-21.8	463
bta-miR-22-3p	-21.8	5
bta-miR-22-3p	-21.2	393
bta-miR-22-3p	-20.9	59
bta-miR-22-3p	-19.9	738
bta-miR-22-3p	-17.4	607
bta-miR-22-3p	-17.3	264
bta-miR-22-3p	-17.1	176
bta-miR-22-3p	-17.0	76
bta-miR-22-3p	-16.8	100
bta-miR-22-3p	-16.4	632
bta-miR-22-3p	-15.8	358
bta-miR-22-3p	-14.3	516
bta-miR-22-3p	-14.1	551
bta-miR-22-3p	-13.8	165
bta-miR-22-3p	-13.3	209
bta-miR-22-3p	-13.2	41
bta-miR-22-3p	-13.2	450
bta-miR-22-3p	-12.9	575
bta-miR-22-3p	-12.6	487
bta-miR-22-3p	-12.4	677
bta-miR-22-3p	-11.8	23
bta-miR-22-3p	-11.1	424
bta-miR-22-3p	-10.7	146
bta-miR-22-3p	-10.4	294
bta-miR-22-3p	-10.2	703
bta-miR-22-3p	-10.1	231
bta-miR-23a	-22.5	619
bta-miR-23a	-21.9	505
bta-miR-23a	-20.7	368
bta-miR-23a	-20.3	478
bta-miR-23a	-19.7	422
bta-miR-23a	-19.2	258
bta-miR-23a	-19.2	575
bta-miR-23a	-18.8	3
bta-miR-23a	-18.5	102
bta-miR-23a	-18.3	598
bta-miR-23a	-17.5	82
bta-miR-23a	-16.9	709
bta-miR-23a	-16.6	176
bta-miR-23a	-16.4	400
bta-miR-23a	-15.5	231
bta-miR-23a	-15.5	650
bta-miR-23a	-15.5	454
bta-miR-23a	-13.9	33
bta-miR-23a	-13.3	357
bta-miR-23a	-13.1	280
bta-miR-23a	-12.5	60
bta-miR-23a	-12.4	731
bta-miR-23a	-12.0	328
bta-miR-23a	-11.9	140
bta-miR-23a	-11.7	313
bta-miR-23a	-11.3	202
bta-miR-23a	-11.2	681
bta-miR-23a	-10.8	168
bta-miR-23a	-10.7	133
bta-miR-23a	-10.6	532
bta-miR-23a	-10.1	163

bta-miR-29b	-22.7	65
bta-miR-29b	-18.9	22
bta-miR-29b	-18.8	366
bta-miR-29b	-18.0	92
bta-miR-29b	-17.8	403
bta-miR-29b	-16.7	586
bta-miR-29b	-16.5	429
bta-miR-29b	-16.4	653
bta-miR-29b	-15.6	736
bta-miR-29b	-15.4	1
bta-miR-29b	-15.0	205
bta-miR-29b	-15.0	264
bta-miR-29b	-14.2	340
bta-miR-29b	-14.1	523
bta-miR-29b	-14.0	464
bta-miR-29b	-13.8	137
bta-miR-29b	-13.2	159
bta-miR-29b	-13.0	494
bta-miR-29b	-12.3	626
bta-miR-29b	-12.2	560
bta-miR-29b	-11.1	685
bta-miR-29b	-10.9	711
bta-miR-29c	-21.2	64
bta-miR-29c	-19.0	340
bta-miR-29c	-18.7	1
bta-miR-29c	-18.6	24
bta-miR-29c	-18.4	462
bta-miR-29c	-18.3	569
bta-miR-29c	-18.1	365
bta-miR-29c	-17.9	595
bta-miR-29c	-17.3	92
bta-miR-29c	-17.1	403
bta-miR-29c	-17.0	716
bta-miR-29c	-14.9	243
bta-miR-29c	-14.8	509
bta-miR-29c	-14.7	435
bta-miR-29c	-14.1	166
bta-miR-29c	-14.0	216
bta-miR-29c	-13.8	647
bta-miR-29c	-12.3	626
bta-miR-29c	-11.4	543
bta-miR-29c	-11.2	487
bta-miR-29c	-10.9	142
bta-miR-29c	-10.8	691
bta-miR-29c	-10.5	277
bta-miR-29c	-10.1	188
bta-miR-361	-23.8	473
bta-miR-361	-22.0	624
bta-miR-361	-20.7	189
bta-miR-361	-20.1	577
bta-miR-361	-19.9	428
bta-miR-361	-19.5	327
bta-miR-361	-19.4	359
bta-miR-361	-19.1	73
bta-miR-361	-18.7	108
bta-miR-361	-18.5	275
bta-miR-361	-18.0	408
bta-miR-361	-17.9	30
bta-miR-361	-16.3	167

bta-miR-361	-16.0	220
bta-miR-361	-15.8	258
bta-miR-361	-14.4	244
bta-miR-361	-14.1	649
bta-miR-361	-14.1	320
bta-miR-361	-13.8	492
bta-miR-361	-13.8	560
bta-miR-361	-13.4	379
bta-miR-361	-13.1	307
bta-miR-361	-13.0	594
bta-miR-361	-12.9	134
bta-miR-361	-12.9	702
bta-miR-361	-12.7	2
bta-miR-361	-12.7	606
bta-miR-361	-11.6	400
bta-miR-361	-11.3	537
bta-miR-361	-11.3	740
bta-miR-361	-11.3	459
bta-miR-361	-10.8	510
bta-miR-361	-10.6	682
bta-miR-361	-10.3	248
bta-miR-423-5p	-28.4	241
bta-miR-423-5p	-24.3	394
bta-miR-423-5p	-22.9	16
bta-miR-423-5p	-22.6	546
bta-miR-423-5p	-21.7	574
bta-miR-423-5p	-20.4	308
bta-miR-423-5p	-20.2	615
bta-miR-423-5p	-20.1	451
bta-miR-423-5p	-19.4	280
bta-miR-423-5p	-19.1	68
bta-miR-423-5p	-19.0	174
bta-miR-423-5p	-18.5	663
bta-miR-423-5p	-17.5	375
bta-miR-423-5p	-17.4	129
bta-miR-423-5p	-17.2	343
bta-miR-423-5p	-17.0	491
bta-miR-423-5p	-16.7	97
bta-miR-423-5p	-15.9	519
bta-miR-423-5p	-15.9	36
bta-miR-423-5p	-15.6	2
bta-miR-423-5p	-15.4	156
bta-miR-423-5p	-15.0	199
bta-miR-423-5p	-14.4	735
bta-miR-423-5p	-13.2	328
bta-miR-423-5p	-12.6	641
bta-miR-423-5p	-12.5	707
bta-miR-423-5p	-11.7	59
bta-miR-423-5p	-10.9	235
bta-miR-423-5p	-10.1	483
bta-miR-423-3p	-32.2	415
bta-miR-423-3p	-29.4	61
bta-miR-423-3p	-29.2	266
bta-miR-423-3p	-27.8	619
bta-miR-423-3p	-27.4	452
bta-miR-423-3p	-25.8	216
bta-miR-423-3p	-24.8	174
bta-miR-423-3p	-23.7	1
bta-miR-423-3p	-23.5	92

bta-miR-423-3p	-23.3	568
bta-miR-423-3p	-22.4	356
bta-miR-423-3p	-20.5	591
bta-miR-423-3p	-18.8	503
bta-miR-423-3p	-17.9	656
bta-miR-423-3p	-17.7	387
bta-miR-423-3p	-17.1	27
bta-miR-423-3p	-16.8	730
bta-miR-423-3p	-16.1	308
bta-miR-423-3p	-15.9	155
bta-miR-423-3p	-15.3	240
bta-miR-423-3p	-15.1	119
bta-miR-423-3p	-13.4	529
bta-miR-423-3p	-12.6	613
bta-miR-423-3p	-11.3	337
bta-miR-423-3p	-10.4	443
bta-miR-425-5p	-22.7	9
bta-miR-425-5p	-20.3	67
bta-miR-425-5p	-19.6	618
bta-miR-425-5p	-18.6	505
bta-miR-425-5p	-18.0	447
bta-miR-425-5p	-16.5	215
bta-miR-425-5p	-15.2	653
bta-miR-425-5p	-14.8	352
bta-miR-425-5p	-14.7	478
bta-miR-425-5p	-14.2	170
bta-miR-425-5p	-14.2	263
bta-miR-425-5p	-13.3	594
bta-miR-425-5p	-12.9	414
bta-miR-425-5p	-12.5	605
bta-miR-425-5p	-11.4	545
bta-miR-425-5p	-11.0	238
bta-miR-425-5p	-10.7	107
bta-miR-425-5p	-10.5	1
bta-miR-425-5p	-10.4	734
bta-miR-425-5p	-10.1	198
bta-miR-425-3p	-29.6	414
bta-miR-425-3p	-27.2	16
bta-miR-425-3p	-24.5	614
bta-miR-425-3p	-23.9	69
bta-miR-425-3p	-21.8	114
bta-miR-425-3p	-21.2	370
bta-miR-425-3p	-21.0	448
bta-miR-425-3p	-20.4	479
bta-miR-425-3p	-19.1	350
bta-miR-425-3p	-19.0	155
bta-miR-425-3p	-18.6	497
bta-miR-425-3p	-17.6	269
bta-miR-425-3p	-16.8	220
bta-miR-425-3p	-16.5	176
bta-miR-425-3p	-15.1	586
bta-miR-425-3p	-15.1	41
bta-miR-425-3p	-14.9	199
bta-miR-425-3p	-14.4	631
bta-miR-425-3p	-14.4	708
bta-miR-425-3p	-13.7	560
bta-miR-425-3p	-13.7	294
bta-miR-425-3p	-13.4	599
bta-miR-425-3p	-12.1	738

bta-miR-425-3p	-11.6	244
bta-miR-425-3p	-11.4	137
bta-miR-425-3p	-11.2	319
bta-miR-425-3p	-11.0	336
bta-miR-450	-20.1	478
bta-miR-450	-18.5	598
bta-miR-450	-17.3	504
bta-miR-450	-17.0	453
bta-miR-450	-16.8	628
bta-miR-450	-16.2	371
bta-miR-450	-16.0	154
bta-miR-450	-15.7	738
bta-miR-450	-15.7	15
bta-miR-450	-14.5	83
bta-miR-450	-14.4	279
bta-miR-450	-13.7	219
bta-miR-450	-13.5	113
bta-miR-450	-13.5	177
bta-miR-450	-13.2	38
bta-miR-450	-13.1	619
bta-miR-450	-12.8	654
bta-miR-450	-12.3	349
bta-miR-450	-12.1	422
bta-miR-450	-12.1	268
bta-miR-450	-10.7	559
bta-miR-450	-10.2	206
bta-miR-450	-10.1	705
bta-miR-455	-24.5	229
bta-miR-455	-19.7	251
bta-miR-455	-19.2	404
bta-miR-455	-17.9	62
bta-miR-455	-17.9	476
bta-miR-455	-17.8	3
bta-miR-455	-17.6	94
bta-miR-455	-17.1	589
bta-miR-455	-16.5	659
bta-miR-455	-16.4	617
bta-miR-455	-14.8	166
bta-miR-455	-14.8	497
bta-miR-455	-14.5	146
bta-miR-455	-14.5	370
bta-miR-455	-14.5	562
bta-miR-455	-14.3	200
bta-miR-455	-14.2	40
bta-miR-455	-13.9	464
bta-miR-455	-13.1	433
bta-miR-455	-12.6	80
bta-miR-455	-12.1	419
bta-miR-455	-11.8	344
bta-miR-455	-11.7	125
bta-miR-455	-11.2	514
bta-miR-455	-11.2	209
bta-miR-455	-11.0	393
bta-miR-455	-10.8	546
bta-miR-455	-10.5	734
bta-miR-455	-10.5	686
bta-miR-455	-10.4	525
bta-miR-455	-10.2	317
bta-miR-455*	-28.3	80

bta-miR-455*	-24.5	359
bta-miR-455*	-23.4	17
bta-miR-455*	-22.9	411
bta-miR-455*	-22.7	617
bta-miR-455*	-22.1	544
bta-miR-455*	-20.6	251
bta-miR-455*	-19.9	167
bta-miR-455*	-19.7	584
bta-miR-455*	-18.8	473
bta-miR-455*	-17.0	659
bta-miR-455*	-16.2	235
bta-miR-455*	-16.0	525
bta-miR-455*	-15.8	304
bta-miR-455*	-14.7	51
bta-miR-455*	-14.7	697
bta-miR-455*	-14.1	126
bta-miR-455*	-13.8	729
bta-miR-455*	-13.5	3
bta-miR-455*	-13.4	496
bta-miR-455*	-13.3	566
bta-miR-455*	-13.1	66
bta-miR-455*	-12.7	142
bta-miR-455*	-12.6	336
bta-miR-455*	-12.4	276
bta-miR-455*	-11.8	199
bta-miR-455*	-11.0	215
bta-miR-455*	-11.0	742
bta-miR-455*	-10.9	430
bta-miR-455*	-10.5	330
bta-miR-455*	-10.2	135
bta-miR-455*	-10.2	324
bta-miR-93	-23.1	490
bta-miR-93	-21.9	367
bta-miR-93	-20.2	581
bta-miR-93	-19.1	408
bta-miR-93	-18.8	91
bta-miR-93	-17.6	13
bta-miR-93	-17.5	60
bta-miR-93	-17.5	473
bta-miR-93	-17.0	167
bta-miR-93	-16.3	192
bta-miR-93	-16.3	307
bta-miR-93	-15.9	115
bta-miR-93	-15.7	642
bta-miR-93	-14.8	615
bta-miR-93	-14.5	261
bta-miR-93	-14.4	341
bta-miR-93	-14.3	30
bta-miR-93	-14.2	550
bta-miR-93	-14.0	448
bta-miR-93	-13.3	385
bta-miR-93	-13.0	215
bta-miR-93	-12.9	431
bta-miR-93	-11.9	684
bta-miR-93	-11.7	729
bta-miR-93	-11.1	235
bta-miR-93	-10.3	138
bta-miR-93	-10.0	630
bta-let-7g	-20.1	115

bta-let-7g	-19.8	582
bta-let-7g	-18.3	267
bta-let-7g	-17.9	375
bta-let-7g	-17.8	553
bta-let-7g	-17.2	184
bta-let-7g	-17.2	413
bta-let-7g	-16.8	61
bta-let-7g	-16.8	292
bta-let-7g	-15.7	685
bta-let-7g	-15.6	6
bta-let-7g	-15.5	618
bta-let-7g	-15.4	730
bta-let-7g	-15.1	453
bta-let-7g	-15.1	235
bta-let-7g	-15.1	518
bta-let-7g	-14.7	96
bta-let-7g	-14.6	477
bta-let-7g	-14.4	149
bta-let-7g	-14.4	359
bta-let-7g	-14.3	648
bta-let-7g	-13.5	251
bta-let-7g	-13.4	43
bta-let-7g	-11.7	497
bta-let-7g	-10.8	602
bta-let-7g	-10.2	403
bta-let-7g	-10.0	332
bta-miR-10b	-20.9	586
bta-miR-10b	-20.6	35
bta-miR-10b	-19.0	614
bta-miR-10b	-18.7	457
bta-miR-10b	-17.6	488
bta-miR-10b	-17.6	373
bta-miR-10b	-17.3	654
bta-miR-10b	-16.5	5
bta-miR-10b	-14.9	72
bta-miR-10b	-14.3	199
bta-miR-10b	-14.3	733
bta-miR-10b	-13.8	267
bta-miR-10b	-13.2	148
bta-miR-10b	-12.7	702
bta-miR-10b	-12.7	415
bta-miR-10b	-11.9	106
bta-miR-10b	-11.7	556
bta-miR-10b	-10.3	689
bta-miR-24-3p	-33.0	403
bta-miR-24-3p	-25.0	6
bta-miR-24-3p	-24.4	87
bta-miR-24-3p	-23.2	465
bta-miR-24-3p	-21.6	550
bta-miR-24-3p	-20.9	577
bta-miR-24-3p	-20.8	359
bta-miR-24-3p	-20.0	209
bta-miR-24-3p	-19.9	427
bta-miR-24-3p	-19.7	257
bta-miR-24-3p	-19.3	169
bta-miR-24-3p	-19.0	33
bta-miR-24-3p	-18.9	59
bta-miR-24-3p	-16.8	231
bta-miR-24-3p	-16.7	641

bta-miR-24-3p	-16.7	502
bta-miR-24-3p	-15.9	714
bta-miR-24-3p	-15.2	191
bta-miR-24-3p	-14.6	657
bta-miR-24-3p	-14.6	293
bta-miR-24-3p	-14.5	686
bta-miR-24-3p	-14.4	524
bta-miR-24-3p	-14.3	387
bta-miR-24-3p	-14.0	606
bta-miR-24-3p	-13.8	331
bta-miR-24-3p	-12.7	626
bta-miR-24-3p	-12.1	146
bta-miR-24-3p	-10.3	249
bta-miR-30a-5p	-24.2	456
bta-miR-30a-5p	-23.3	166
bta-miR-30a-5p	-21.6	72
bta-miR-30a-5p	-21.3	512
bta-miR-30a-5p	-21.1	586
bta-miR-30a-5p	-20.7	205
bta-miR-30a-5p	-20.4	419
bta-miR-30a-5p	-19.2	4
bta-miR-30a-5p	-18.2	282
bta-miR-30a-5p	-16.5	236
bta-miR-30a-5p	-16.2	342
bta-miR-30a-5p	-16.2	29
bta-miR-30a-5p	-15.6	390
bta-miR-30a-5p	-15.1	539
bta-miR-30a-5p	-15.0	559
bta-miR-30a-5p	-14.5	263
bta-miR-30a-5p	-14.3	653
bta-miR-30a-5p	-14.1	115
bta-miR-30a-5p	-13.5	626
bta-miR-30a-5p	-13.1	363
bta-miR-30a-5p	-12.8	403
bta-miR-30a-5p	-12.5	98
bta-miR-30a-5p	-12.2	444
bta-miR-30a-5p	-11.6	499
bta-miR-30a-5p	-11.6	698
bta-miR-30a-5p	-11.3	58
bta-miR-30a-5p	-11.3	184
bta-miR-30a-5p	-11.3	637
bta-miR-30a-5p	-10.9	740
bta-miR-30a-5p	-10.6	223
bta-miR-30a-5p	-10.4	316
bta-miR-200b	-19.4	4
bta-miR-200b	-18.9	597
bta-miR-200b	-17.8	53
bta-miR-200b	-17.4	410
bta-miR-200b	-17.1	367
bta-miR-200b	-17.0	565
bta-miR-200b	-16.7	501
bta-miR-200b	-15.5	476
bta-miR-200b	-15.1	618
bta-miR-200b	-14.8	263
bta-miR-200b	-14.4	144
bta-miR-200b	-14.3	645
bta-miR-200b	-14.0	109
bta-miR-200b	-13.2	23
bta-miR-200b	-12.8	82

bta-miR-200b	-12.5	428
bta-miR-200b	-12.4	126
bta-miR-200b	-12.3	297
bta-miR-200b	-11.8	172
bta-miR-200b	-11.8	275
bta-miR-200b	-11.5	667
bta-miR-200b	-11.4	534
bta-miR-200b	-11.3	384
bta-miR-200b	-11.2	191
bta-miR-200b	-10.8	207
bta-miR-200b	-10.7	459
bta-miR-200b	-10.7	741
bta-miR-200b	-10.4	118
bta-miR-200b	-10.0	237
bta-miR-200b	-10.0	354
bta-miR-7	-22.1	372
bta-miR-7	-21.5	487
bta-miR-7	-19.5	270
bta-miR-7	-17.7	198
bta-miR-7	-17.6	171
bta-miR-7	-17.4	729
bta-miR-7	-16.8	647
bta-miR-7	-16.6	407
bta-miR-7	-16.2	105
bta-miR-7	-15.6	64
bta-miR-7	-15.1	623
bta-miR-7	-14.9	447
bta-miR-7	-14.3	560
bta-miR-7	-13.8	218
bta-miR-7	-13.6	301
bta-miR-7	-13.4	700
bta-miR-7	-13.2	26
bta-miR-7	-12.9	576
bta-miR-7	-12.4	605
bta-miR-7	-12.0	54
bta-miR-7	-11.6	122
bta-miR-7	-11.6	240
bta-miR-7	-11.2	352
bta-miR-7	-11.0	1
bta-miR-7	-10.4	684
bta-let-7a	-24.3	85
bta-let-7a	-20.0	414
bta-let-7a	-18.1	375
bta-let-7a	-17.6	296
bta-let-7a	-17.6	456
bta-let-7a	-17.0	244
bta-let-7a	-16.7	183
bta-let-7a	-15.7	16
bta-let-7a	-15.6	582
bta-let-7a	-15.6	507
bta-let-7a	-15.5	359
bta-let-7a	-15.3	697
bta-let-7a	-15.1	155
bta-let-7a	-15.1	266
bta-let-7a	-14.7	122
bta-let-7a	-14.6	553
bta-let-7a	-14.6	648
bta-let-7a	-14.0	526
bta-let-7a	-13.9	50

bta-let-7a	-13.8	623
bta-let-7a	-13.7	733
bta-let-7a	-13.6	399
bta-let-7a	-13.4	199
bta-let-7a	-11.9	61
bta-let-7a	-11.8	480
bta-let-7a	-10.3	332
bta-miR-150	-27.1	593
bta-miR-150	-24.8	56
bta-miR-150	-23.2	415
bta-miR-150	-22.2	488
bta-miR-150	-18.3	367
bta-miR-150	-18.1	444
bta-miR-150	-17.7	264
bta-miR-150	-17.7	160
bta-miR-150	-17.3	13
bta-miR-150	-16.8	464
bta-miR-150	-15.9	199
bta-miR-150	-14.6	109
bta-miR-150	-13.9	739
bta-miR-150	-13.5	654
bta-miR-150	-13.2	624
bta-miR-150	-12.5	560
bta-miR-150	-11.2	232
bta-miR-150	-10.8	148
bta-miR-150	-10.7	346
bta-miR-150	-10.2	353
bta-miR-342	-25.7	610
bta-miR-342	-25.2	11
bta-miR-342	-21.2	418
bta-miR-342	-20.4	499
bta-miR-342	-19.1	69
bta-miR-342	-19.0	228
bta-miR-342	-17.7	444
bta-miR-342	-16.8	366
bta-miR-342	-16.0	659
bta-miR-342	-15.7	201
bta-miR-342	-15.3	37
bta-miR-342	-15.0	575
bta-miR-342	-14.4	113
bta-miR-342	-13.4	737
bta-miR-342	-12.7	280
bta-miR-342	-12.6	351
bta-miR-342	-12.5	172
bta-miR-342	-12.3	1
bta-miR-342	-12.1	562
bta-miR-342	-11.0	709
bta-miR-342	-10.6	86
bta-miR-487b	-23.1	616
bta-miR-487b	-22.4	357
bta-miR-487b	-22.3	411
bta-miR-487b	-21.4	11
bta-miR-487b	-21.4	79
bta-miR-487b	-20.4	496
bta-miR-487b	-20.4	576
bta-miR-487b	-16.8	252
bta-miR-487b	-16.6	109
bta-miR-487b	-16.5	174
bta-miR-487b	-16.2	446

bta-miR-487b	-15.4	478
bta-miR-487b	-15.0	36
bta-miR-487b	-14.8	709
bta-miR-487b	-14.6	227
bta-miR-487b	-14.2	661
bta-miR-487b	-12.6	566
bta-miR-487b	-12.5	69
bta-miR-487b	-12.3	155
bta-miR-487b	-11.9	327
bta-miR-487b	-11.7	544
bta-miR-487b	-11.6	634
bta-miR-487b	-11.4	167
bta-miR-487b	-11.4	401
bta-miR-487b	-11.1	130
bta-miR-487b	-10.8	1
bta-miR-487b	-10.8	306
bta-miR-487b	-10.6	280
bta-miR-487b	-10.5	28
bta-miR-487b	-10.4	592
bta-miR-487b	-10.4	204
bta-miR-487b	-10.4	738
bta-miR-487b	-10.2	320
bta-miR-532	-22.0	463
bta-miR-532	-21.4	576
bta-miR-532	-20.7	255
bta-miR-532	-20.5	617
bta-miR-532	-19.8	86
bta-miR-532	-19.1	140
bta-miR-532	-18.6	4
bta-miR-532	-18.5	422
bta-miR-532	-18.1	389
bta-miR-532	-17.7	40
bta-miR-532	-16.6	275
bta-miR-532	-16.2	191
bta-miR-532	-15.0	662
bta-miR-532	-14.9	164
bta-miR-532	-14.9	498
bta-miR-532	-14.3	368
bta-miR-532	-13.8	709
bta-miR-532	-13.8	209
bta-miR-532	-13.6	341
bta-miR-532	-13.5	638
bta-miR-532	-13.2	533
bta-miR-532	-13.1	117
bta-miR-532	-12.8	230
bta-miR-532	-12.2	307
bta-miR-532	-11.7	741
bta-miR-532	-11.7	484
bta-miR-532	-11.1	694
bta-miR-532	-10.9	454
bta-miR-532	-10.8	563
bta-miR-532	-10.1	22
bta-miR-122	-20.8	114
bta-miR-122	-20.3	159
bta-miR-122	-19.1	558
bta-miR-122	-19.0	28
bta-miR-122	-18.9	242
bta-miR-122	-18.6	371
bta-miR-122	-18.4	69

bta-miR-122	-17.3	651
bta-miR-122	-16.9	485
bta-miR-122	-16.6	1
bta-miR-122	-16.4	332
bta-miR-122	-16.0	137
bta-miR-122	-16.0	297
bta-miR-122	-15.9	455
bta-miR-122	-15.9	592
bta-miR-122	-14.3	410
bta-miR-122	-14.2	620
bta-miR-122	-13.9	204
bta-miR-122	-13.9	272
bta-miR-122	-13.7	520
bta-miR-122	-12.8	734
bta-miR-122	-11.9	395
bta-miR-122	-11.7	702
bta-miR-122	-11.6	682
bta-miR-122	-11.0	92
bta-miR-122	-10.3	232
bta-miR-122	-10.0	536
bta-miR-30c	-21.7	614
bta-miR-30c	-21.4	436
bta-miR-30c	-19.8	13
bta-miR-30c	-18.7	66
bta-miR-30c	-17.9	504
bta-miR-30c	-17.2	346
bta-miR-30c	-16.9	199
bta-miR-30c	-16.7	403
bta-miR-30c	-16.2	477
bta-miR-30c	-15.9	586
bta-miR-30c	-15.3	267
bta-miR-30c	-14.5	537
bta-miR-30c	-14.0	240
bta-miR-30c	-13.7	650
bta-miR-30c	-12.0	223
bta-miR-30c	-11.9	172
bta-miR-30c	-11.8	92
bta-miR-30c	-11.0	38
bta-miR-30c	-10.8	185
bta-miR-30c	-10.0	453
bta-let-7i	-22.1	105
bta-let-7i	-20.6	264
bta-let-7i	-19.7	170
bta-let-7i	-19.4	125
bta-let-7i	-18.7	12
bta-let-7i	-18.5	407
bta-let-7i	-18.4	447
bta-let-7i	-18.3	372
bta-let-7i	-17.9	653
bta-let-7i	-17.9	479
bta-let-7i	-17.8	198
bta-let-7i	-17.5	64
bta-let-7i	-17.3	516
bta-let-7i	-16.9	297
bta-let-7i	-16.7	234
bta-let-7i	-16.1	614
bta-let-7i	-16.0	148
bta-let-7i	-15.5	348
bta-let-7i	-15.5	555

bta-let-7i	-15.3	737
bta-let-7i	-15.1	575
bta-let-7i	-13.7	37
bta-let-7i	-13.5	495
bta-let-7i	-13.4	687
bta-let-7i	-12.3	597
bta-let-7i	-11.1	435
bta-let-7i	-10.0	332
bta-miR-23b-5p	-25.7	16
bta-miR-23b-5p	-24.7	410
bta-miR-23b-5p	-23.4	263
bta-miR-23b-5p	-22.8	487
bta-miR-23b-5p	-22.4	234
bta-miR-23b-5p	-21.7	113
bta-miR-23b-5p	-21.5	64
bta-miR-23b-5p	-21.5	653
bta-miR-23b-5p	-21.0	605
bta-miR-23b-5p	-20.9	298
bta-miR-23b-5p	-20.6	463
bta-miR-23b-5p	-20.5	165
bta-miR-23b-5p	-20.0	559
bta-miR-23b-5p	-19.1	583
bta-miR-23b-5p	-18.5	354
bta-miR-23b-5p	-18.1	37
bta-miR-23b-5p	-16.6	439
bta-miR-23b-5p	-16.5	205
bta-miR-23b-5p	-16.3	1
bta-miR-23b-5p	-15.4	375
bta-miR-23b-5p	-14.9	692
bta-miR-23b-5p	-14.2	89
bta-miR-23b-5p	-14.2	128
bta-miR-23b-5p	-13.4	740
bta-miR-23b-5p	-12.8	182
bta-miR-23b-5p	-11.7	147
bta-miR-23b-5p	-11.6	516
bta-miR-23b-5p	-11.1	325
bta-miR-23b-5p	-10.8	544
bta-miR-23b-5p	-10.4	629
bta-miR-23b-5p	-10.4	332
bta-miR-23b-3p	-21.8	359
bta-miR-23b-3p	-21.5	618
bta-miR-23b-3p	-20.3	422
bta-miR-23b-3p	-19.6	576
bta-miR-23b-3p	-19.3	464
bta-miR-23b-3p	-19.3	3
bta-miR-23b-3p	-19.2	252
bta-miR-23b-3p	-19.0	81
bta-miR-23b-3p	-19.0	505
bta-miR-23b-3p	-16.9	110
bta-miR-23b-3p	-16.4	400
bta-miR-23b-3p	-16.2	644
bta-miR-23b-3p	-15.9	598
bta-miR-23b-3p	-15.7	40
bta-miR-23b-3p	-15.5	155
bta-miR-23b-3p	-15.2	231
bta-miR-23b-3p	-13.8	709
bta-miR-23b-3p	-13.4	687
bta-miR-23b-3p	-13.3	191
bta-miR-23b-3p	-12.1	740

bta-miR-23b-3p	-12.0	328
bta-miR-23b-3p	-12.0	555
bta-miR-23b-3p	-11.8	527
bta-miR-23b-3p	-11.1	215
bta-miR-23b-3p	-11.1	314
bta-miR-23b-3p	-10.7	133
bta-miR-23b-3p	-10.7	140
bta-miR-23b-3p	-10.4	247
bta-miR-25	-28.0	364
bta-miR-25	-27.8	9
bta-miR-25	-24.6	481
bta-miR-25	-22.3	603
bta-miR-25	-21.7	567
bta-miR-25	-20.1	350
bta-miR-25	-19.4	499
bta-miR-25	-18.4	423
bta-miR-25	-18.3	71
bta-miR-25	-18.2	200
bta-miR-25	-17.8	114
bta-miR-25	-17.0	263
bta-miR-25	-16.8	165
bta-miR-25	-14.7	398
bta-miR-25	-14.4	595
bta-miR-25	-14.3	29
bta-miR-25	-14.3	737
bta-miR-25	-13.9	234
bta-miR-25	-13.6	649
bta-miR-25	-12.8	414
bta-miR-25	-11.6	623
bta-miR-25	-11.5	51
bta-miR-25	-11.0	544
bta-miR-25	-10.7	182
bta-miR-25	-10.5	154
bta-miR-25	-10.2	454
bta-miR-25	-10.1	100
bta-miR-34c	-25.7	107
bta-miR-34c	-23.5	410
bta-miR-34c	-22.1	8
bta-miR-34c	-21.0	639
bta-miR-34c	-20.7	182
bta-miR-34c	-20.4	150
bta-miR-34c	-20.2	544
bta-miR-34c	-19.3	67
bta-miR-34c	-18.1	351
bta-miR-34c	-17.5	452
bta-miR-34c	-17.5	277
bta-miR-34c	-16.7	692
bta-miR-34c	-16.6	500
bta-miR-34c	-16.4	576
bta-miR-34c	-15.7	125
bta-miR-34c	-15.2	37
bta-miR-34c	-14.9	376
bta-miR-34c	-13.9	476
bta-miR-34c	-12.8	663
bta-miR-34c	-12.8	263
bta-miR-34c	-12.7	738
bta-miR-34c	-12.5	623
bta-miR-34c	-12.4	238
bta-miR-34c	-11.6	524

bta-miR-34c	-10.9	54
bta-miR-34c	-10.9	170
bta-miR-34c	-10.4	432
bta-miR-34c	-10.4	311
bta-miR-363	-25.2	586
bta-miR-363	-22.4	494
bta-miR-363	-20.7	614
bta-miR-363	-20.3	8
bta-miR-363	-19.5	361
bta-miR-363	-19.5	473
bta-miR-363	-19.4	412
bta-miR-363	-19.1	66
bta-miR-363	-17.7	228
bta-miR-363	-17.3	654
bta-miR-363	-17.2	449
bta-miR-363	-15.8	100
bta-miR-363	-15.1	199
bta-miR-363	-14.4	532
bta-miR-363	-13.9	172
bta-miR-363	-13.7	737
bta-miR-363	-13.3	338
bta-miR-363	-13.1	264
bta-miR-363	-13.0	698
bta-miR-363	-12.6	555
bta-miR-363	-12.1	38
bta-miR-363	-11.8	238
bta-miR-363	-11.3	523
bta-miR-363	-10.1	155
bta-let-7a*	-18.5	610
bta-let-7a*	-18.2	405
bta-let-7a*	-16.9	479
bta-let-7a*	-15.7	516
bta-let-7a*	-15.2	348
bta-let-7a*	-14.7	225
bta-let-7a*	-14.5	735
bta-let-7a*	-14.1	653
bta-let-7a*	-13.9	69
bta-let-7a*	-13.9	441
bta-let-7a*	-12.4	8
bta-let-7a*	-12.2	196
bta-let-7a*	-10.6	102
bta-let-7a*	-10.5	570
bta-let-7a*	-10.4	539
bta-let-7a*	-10.2	1
bta-let-7b	-24.8	99
bta-let-7b	-23.8	414
bta-let-7b	-21.8	125
bta-let-7b	-19.8	503
bta-let-7b	-19.6	147
bta-let-7b	-19.6	237
bta-let-7b	-19.6	377
bta-let-7b	-19.1	300
bta-let-7b	-18.9	166
bta-let-7b	-18.0	50
bta-let-7b	-17.9	468
bta-let-7b	-17.9	570
bta-let-7b	-17.4	340
bta-let-7b	-15.5	197
bta-let-7b	-15.5	696

bta-let-7b	-15.2	648
bta-let-7b	-14.8	29
bta-let-7b	-14.3	560
bta-let-7b	-14.2	739
bta-let-7b	-13.8	623
bta-let-7b	-13.8	267
bta-let-7b	-13.7	318
bta-let-7b	-13.6	399
bta-let-7b	-12.4	595
bta-let-7b	-11.9	13
bta-let-7b	-11.7	525
bta-let-7b	-11.5	457
bta-let-7b	-10.4	485
bta-let-7c	-24.8	99
bta-let-7c	-20.9	407
bta-let-7c	-19.7	125
bta-let-7c	-19.1	237
bta-let-7c	-18.5	563
bta-let-7c	-18.3	482
bta-let-7c	-18.0	166
bta-let-7c	-17.9	298
bta-let-7c	-17.7	340
bta-let-7c	-17.3	456
bta-let-7c	-17.2	50
bta-let-7c	-16.8	375
bta-let-7c	-15.6	507
bta-let-7c	-15.4	12
bta-let-7c	-15.3	697
bta-let-7c	-15.1	266
bta-let-7c	-14.3	648
bta-let-7c	-14.2	150
bta-let-7c	-13.9	584
bta-let-7c	-13.8	623
bta-let-7c	-13.7	733
bta-let-7c	-13.6	198
bta-let-7c	-12.9	527
bta-let-7c	-10.4	318
bta-let-7e	-21.1	457
bta-let-7e	-20.5	86
bta-let-7e	-20.3	415
bta-let-7e	-19.2	702
bta-let-7e	-18.3	241
bta-let-7e	-18.0	378
bta-let-7e	-18.0	507
bta-let-7e	-17.6	296
bta-let-7e	-17.0	19
bta-let-7e	-16.9	185
bta-let-7e	-16.7	553
bta-let-7e	-16.7	618
bta-let-7e	-16.6	271
bta-let-7e	-15.9	353
bta-let-7e	-15.6	582
bta-let-7e	-15.6	399
bta-let-7e	-15.3	156
bta-let-7e	-15.3	685
bta-let-7e	-15.1	649
bta-let-7e	-15.1	122
bta-let-7e	-14.6	50
bta-let-7e	-14.4	320

bta-let-7e	-12.7	738
bta-let-7e	-11.9	61
bta-let-7e	-10.8	671
bta-let-7e	-10.7	6
bta-let-7e	-10.4	141
bta-let-7e	-10.3	213
bta-miR-15a	-21.2	10
bta-miR-15a	-20.7	406
bta-miR-15a	-20.1	649
bta-miR-15a	-19.1	542
bta-miR-15a	-19.0	570
bta-miR-15a	-18.6	66
bta-miR-15a	-17.6	516
bta-miR-15a	-17.6	106
bta-miR-15a	-16.6	361
bta-miR-15a	-16.1	737
bta-miR-15a	-15.4	271
bta-miR-15a	-15.3	602
bta-miR-15a	-15.2	122
bta-miR-15a	-14.5	481
bta-miR-15a	-13.9	554
bta-miR-15a	-13.5	28
bta-miR-15a	-13.3	165
bta-miR-15a	-13.3	242
bta-miR-15a	-12.9	339
bta-miR-15a	-12.6	423
bta-miR-15a	-11.9	454
bta-miR-15a	-11.0	696
bta-miR-15a	-10.6	298
bta-miR-15a	-10.0	508
bta-miR-195	-25.8	408
bta-miR-195	-23.0	569
bta-miR-195	-22.8	51
bta-miR-195	-22.6	668
bta-miR-195	-20.5	531
bta-miR-195	-19.8	109
bta-miR-195	-19.6	7
bta-miR-195	-19.5	251
bta-miR-195	-17.9	740
bta-miR-195	-17.1	473
bta-miR-195	-16.9	515
bta-miR-195	-15.8	360
bta-miR-195	-15.5	425
bta-miR-195	-14.8	76
bta-miR-195	-14.5	172
bta-miR-195	-14.5	553
bta-miR-195	-14.2	644
bta-miR-195	-13.8	30
bta-miR-195	-13.7	605
bta-miR-195	-13.6	283
bta-miR-195	-13.6	206
bta-miR-195	-13.1	126
bta-miR-195	-11.9	454
bta-miR-195	-10.7	493
bta-miR-19a	-19.5	653
bta-miR-19a	-19.4	5
bta-miR-19a	-18.9	539
bta-miR-19a	-17.3	358
bta-miR-19a	-16.5	491

bta-miR-19a	-16.4	516
bta-miR-19a	-16.2	597
bta-miR-19a	-16.1	410
bta-miR-19a	-15.7	263
bta-miR-19a	-14.5	165
bta-miR-19a	-14.2	463
bta-miR-19a	-14.2	199
bta-miR-19a	-14.0	683
bta-miR-19a	-13.6	67
bta-miR-19a	-13.5	100
bta-miR-19a	-11.2	575
bta-miR-19a	-10.2	37
bta-miR-19a	-10.2	307
bta-miR-19a	-10.2	435
bta-miR-19a	-10.1	628
bta-miR-19a	-10.1	737
bta-miR-19b	-21.8	653
bta-miR-19b	-21.7	5
bta-miR-19b	-20.7	539
bta-miR-19b	-19.6	358
bta-miR-19b	-18.8	491
bta-miR-19b	-18.4	410
bta-miR-19b	-18.1	597
bta-miR-19b	-17.6	263
bta-miR-19b	-16.5	463
bta-miR-19b	-16.4	165
bta-miR-19b	-15.9	67
bta-miR-19b	-15.7	694
bta-miR-19b	-14.9	100
bta-miR-19b	-14.2	516
bta-miR-19b	-12.5	199
bta-miR-19b	-11.8	576
bta-miR-19b	-11.2	230
bta-miR-19b	-10.9	741
bta-miR-19b	-10.4	435
bta-miR-19b	-10.2	37
bta-miR-19b	-10.2	307
bta-miR-19b	-10.1	628
bta-miR-19b	-10.0	683
bta-miR-204	-26.8	604
bta-miR-204	-23.9	418
bta-miR-204	-23.7	575
bta-miR-204	-20.7	203
bta-miR-204	-19.9	11
bta-miR-204	-18.8	154
bta-miR-204	-18.5	481
bta-miR-204	-18.1	66
bta-miR-204	-15.6	269
bta-miR-204	-12.7	654
bta-miR-204	-12.6	351
bta-miR-204	-12.4	109
bta-miR-204	-12.0	454
bta-miR-204	-11.1	86
bta-miR-204	-10.9	41
bta-miR-331	-30.9	615
bta-miR-331	-27.0	59
bta-miR-331	-26.8	416
bta-miR-331	-24.5	453
bta-miR-331	-23.5	214

bta-miR-331	-21.3	598
bta-miR-331	-21.2	267
bta-miR-331	-21.0	98
bta-miR-331	-20.7	154
bta-miR-331	-20.1	5
bta-miR-331	-18.3	502
bta-miR-331	-16.3	345
bta-miR-331	-16.0	567
bta-miR-331	-14.8	26
bta-miR-331	-13.8	390
bta-miR-331	-11.9	181
bta-miR-331	-11.7	707
bta-miR-331	-11.3	663
bta-miR-331	-10.8	304
bta-miR-331	-10.4	127
bta-miR-331	-10.3	330
bta-miR-331	-10.3	740
bta-miR-331	-10.1	142
bta-miR-331	-10.0	135
bta-miR-331	-10.0	324
bta-miR-331	-10.0	119
bta-miR-34a	-27.2	407
bta-miR-34a	-26.3	106
bta-miR-34a	-23.8	340
bta-miR-34a	-22.3	165
bta-miR-34a	-21.9	66
bta-miR-34a	-20.5	647
bta-miR-34a	-20.2	12
bta-miR-34a	-19.9	602
bta-miR-34a	-19.5	457
bta-miR-34a	-19.4	545
bta-miR-34a	-19.3	570
bta-miR-34a	-18.7	366
bta-miR-34a	-17.6	34
bta-miR-34a	-17.4	512
bta-miR-34a	-16.8	623
bta-miR-34a	-16.6	263
bta-miR-34a	-16.1	736
bta-miR-34a	-16.0	206
bta-miR-34a	-14.6	488
bta-miR-34a	-14.5	431
bta-miR-34a	-14.2	149
bta-miR-34a	-14.0	283
bta-miR-34a	-13.2	243
bta-miR-34a	-12.2	700
bta-miR-34a	-11.6	1
bta-miR-34a	-10.8	318
bta-miR-34a	-10.8	595
bta-miR-34a	-10.7	385
bta-miR-34a	-10.6	129
bta-miR-365-5p	-31.2	23
bta-miR-365-5p	-29.8	382
bta-miR-365-5p	-29.4	244
bta-miR-365-5p	-25.6	565
bta-miR-365-5p	-25.0	106
bta-miR-365-5p	-23.3	87
bta-miR-365-5p	-23.3	148
bta-miR-365-5p	-22.7	473
bta-miR-365-5p	-22.5	427

bta-miR-365-5p	-22.4	319
bta-miR-365-5p	-22.2	353
bta-miR-365-5p	-22.1	206
bta-miR-365-5p	-21.2	488
bta-miR-365-5p	-19.4	56
bta-miR-365-5p	-19.2	271
bta-miR-365-5p	-18.0	457
bta-miR-365-5p	-17.6	186
bta-miR-365-5p	-17.3	300
bta-miR-365-5p	-16.9	4
bta-miR-365-5p	-16.6	695
bta-miR-365-5p	-15.2	654
bta-miR-365-5p	-14.9	137
bta-miR-365-5p	-14.8	624
bta-miR-365-5p	-13.9	400
bta-miR-365-5p	-13.1	544
bta-miR-365-5p	-13.0	519
bta-miR-365-5p	-12.8	46
bta-miR-365-5p	-12.8	583
bta-miR-365-5p	-12.3	602
bta-miR-365-5p	-11.6	739
bta-miR-365-3p	-24.7	612
bta-miR-365-3p	-22.0	69
bta-miR-365-3p	-19.5	452
bta-miR-365-3p	-17.7	268
bta-miR-365-3p	-14.9	11
bta-miR-365-3p	-14.5	630
bta-miR-365-3p	-13.8	217
bta-miR-365-3p	-13.6	584
bta-miR-365-3p	-13.1	499
bta-miR-365-3p	-12.9	406
bta-miR-365-3p	-12.7	438
bta-miR-365-3p	-12.3	174
bta-miR-365-3p	-12.0	114
bta-miR-365-3p	-11.6	423
bta-miR-365-3p	-10.6	279
bta-miR-365-3p	-10.5	651
bta-miR-365-3p	-10.2	710
bta-miR-365-3p	-10.0	26
bta-miR-374a	-18.6	7
bta-miR-374a	-14.9	56
bta-miR-374a	-14.8	537
bta-miR-374a	-14.1	654
bta-miR-374a	-13.7	702
bta-miR-374a	-13.3	560
bta-miR-374a	-13.2	520
bta-miR-374a	-13.1	606
bta-miR-374a	-12.9	457
bta-miR-374a	-12.7	624
bta-miR-374a	-12.2	277
bta-miR-374a	-11.7	81
bta-miR-374a	-11.6	740
bta-miR-374a	-10.6	415
bta-miR-374a	-10.4	489
bta-miR-497	-23.7	10
bta-miR-497	-23.2	405
bta-miR-497	-22.1	360
bta-miR-497	-21.9	66
bta-miR-497	-21.9	562

bta-miR-497	-21.5	100
bta-miR-497	-20.4	505
bta-miR-497	-19.7	602
bta-miR-497	-19.6	271
bta-miR-497	-18.9	649
bta-miR-497	-18.5	544
bta-miR-497	-17.5	485
bta-miR-497	-17.3	160
bta-miR-497	-17.0	729
bta-miR-497	-15.9	28
bta-miR-497	-15.7	122
bta-miR-497	-15.0	425
bta-miR-497	-14.3	256
bta-miR-497	-14.2	199
bta-miR-497	-13.4	49
bta-miR-497	-12.2	233
bta-miR-497	-11.2	147
bta-miR-497	-11.1	460
bta-miR-497	-10.8	298
bta-miR-497	-10.6	339
bta-miR-497	-10.1	631
bta-miR-660	-23.1	472
bta-miR-660	-22.0	586
bta-miR-660	-21.2	85
bta-miR-660	-21.0	12
bta-miR-660	-20.1	165
bta-miR-660	-19.4	50
bta-miR-660	-19.4	243
bta-miR-660	-19.1	623
bta-miR-660	-17.9	410
bta-miR-660	-17.9	429
bta-miR-660	-16.9	205
bta-miR-660	-16.7	559
bta-miR-660	-16.3	513
bta-miR-660	-15.9	361
bta-miR-660	-15.1	456
bta-miR-660	-14.5	539
bta-miR-660	-13.7	282
bta-miR-660	-13.4	638
bta-miR-660	-13.0	128
bta-miR-660	-12.1	737
bta-miR-660	-12.0	183
bta-miR-660	-11.6	299
bta-miR-660	-11.3	500
bta-miR-660	-10.9	691
bta-miR-660	-10.6	116
bta-miR-99b	-33.0	606
bta-miR-99b	-23.4	66
bta-miR-99b	-22.4	450
bta-miR-99b	-21.3	494
bta-miR-99b	-21.2	573
bta-miR-99b	-20.6	654
bta-miR-99b	-19.6	415
bta-miR-99b	-18.2	8
bta-miR-99b	-16.2	172
bta-miR-99b	-16.0	381
bta-miR-99b	-14.3	206
bta-miR-99b	-13.8	35
bta-miR-99b	-13.7	252

bta-miR-99b	-12.9	736
bta-miR-99b	-12.8	527
bta-miR-99b	-12.4	109
bta-miR-99b	-12.3	271
bta-miR-99b	-10.2	560
bta-miR-99b	-10.0	85
bta-miR-1	-18.0	304
bta-miR-1	-17.2	493
bta-miR-1	-15.8	628
bta-miR-1	-15.0	206
bta-miR-1	-14.9	66
bta-miR-1	-14.7	283
bta-miR-1	-14.6	697
bta-miR-1	-14.1	106
bta-miR-1	-13.2	679
bta-miR-1	-13.1	544
bta-miR-1	-13.0	518
bta-miR-1	-12.9	10
bta-miR-1	-12.9	379
bta-miR-1	-12.8	149
bta-miR-1	-12.6	410
bta-miR-1	-12.3	53
bta-miR-1	-12.2	457
bta-miR-1	-11.1	584
bta-miR-1	-10.9	473
bta-miR-1	-10.3	353
bta-miR-1	-10.1	733
bta-miR-1	-10.1	661
bta-miR-1	-10.0	244
bta-miR-100	-26.8	606
bta-miR-100	-24.2	494
bta-miR-100	-18.1	66
bta-miR-100	-17.6	464
bta-miR-100	-16.3	415
bta-miR-100	-15.9	11
bta-miR-100	-15.2	434
bta-miR-100	-15.1	373
bta-miR-100	-14.7	35
bta-miR-100	-14.6	573
bta-miR-100	-14.2	654
bta-miR-100	-14.1	106
bta-miR-100	-13.9	702
bta-miR-100	-12.9	206
bta-miR-100	-12.5	150
bta-miR-100	-12.5	344
bta-miR-100	-12.1	450
bta-miR-100	-11.9	551
bta-miR-100	-11.8	271
bta-miR-100	-11.5	736
bta-miR-100	-11.1	389
bta-miR-100	-11.0	121
bta-miR-105b	-24.0	473
bta-miR-105b	-23.9	571
bta-miR-105b	-22.6	606
bta-miR-105b	-22.0	408
bta-miR-105b	-20.3	446
bta-miR-105b	-19.6	232
bta-miR-105b	-19.5	51
bta-miR-105b	-18.3	9

bta-miR-105b	-18.2	341
bta-miR-105b	-17.9	172
bta-miR-105b	-17.2	263
bta-miR-105b	-15.7	649
bta-miR-105b	-15.3	216
bta-miR-105b	-15.2	367
bta-miR-105b	-15.0	99
bta-miR-105b	-14.5	510
bta-miR-105b	-14.5	34
bta-miR-105b	-14.2	73
bta-miR-105b	-12.7	495
bta-miR-105b	-12.0	595
bta-miR-105b	-10.9	541
bta-miR-105b	-10.9	551
bta-miR-105b	-10.9	669
bta-miR-105a	-25.2	408
bta-miR-105a	-24.1	473
bta-miR-105a	-21.9	606
bta-miR-105a	-20.8	51
bta-miR-105a	-20.6	199
bta-miR-105a	-20.4	263
bta-miR-105a	-20.1	571
bta-miR-105a	-20.0	7
bta-miR-105a	-19.6	232
bta-miR-105a	-19.3	447
bta-miR-105a	-18.3	167
bta-miR-105a	-17.7	341
bta-miR-105a	-17.2	106
bta-miR-105a	-17.0	495
bta-miR-105a	-16.9	510
bta-miR-105a	-15.5	367
bta-miR-105a	-15.3	34
bta-miR-105a	-14.7	119
bta-miR-105a	-12.7	636
bta-miR-105a	-11.6	595
bta-miR-105a	-11.4	560
bta-miR-105a	-11.3	669
bta-miR-105a	-11.3	147
bta-miR-105a	-10.9	541
bta-miR-106b	-21.6	4
bta-miR-106b	-21.1	490
bta-miR-106b	-20.7	537
bta-miR-106b	-20.6	654
bta-miR-106b	-20.5	369
bta-miR-106b	-18.6	566
bta-miR-106b	-18.5	416
bta-miR-106b	-17.6	112
bta-miR-106b	-16.7	60
bta-miR-106b	-16.5	385
bta-miR-106b	-15.8	271
bta-miR-106b	-15.4	87
bta-miR-106b	-15.1	199
bta-miR-106b	-14.5	526
bta-miR-106b	-14.2	473
bta-miR-106b	-13.8	615
bta-miR-106b	-13.8	684
bta-miR-106b	-13.0	250
bta-miR-106b	-12.6	172
bta-miR-106b	-12.2	428

bta-miR-106b	-12.1	403
bta-miR-106b	-12.0	639
bta-miR-106b	-12.0	38
bta-miR-106b	-11.9	360
bta-miR-106b	-11.9	739
bta-miR-106b	-11.8	25
bta-miR-106b	-11.5	591
bta-miR-106b	-11.3	161
bta-miR-106b	-11.0	286
bta-miR-106b	-10.9	215
bta-miR-106b	-10.6	344
bta-miR-106b	-10.1	133
bta-miR-124b	-22.7	503
bta-miR-124b	-22.4	413
bta-miR-124b	-20.0	155
bta-miR-124b	-19.7	39
bta-miR-124b	-19.4	17
bta-miR-124b	-19.0	654
bta-miR-124b	-18.9	370
bta-miR-124b	-18.8	542
bta-miR-124b	-18.8	605
bta-miR-124b	-18.6	115
bta-miR-124b	-18.4	576
bta-miR-124b	-18.2	341
bta-miR-124b	-18.0	464
bta-miR-124b	-17.9	85
bta-miR-124b	-17.7	488
bta-miR-124b	-17.1	177
bta-miR-124b	-16.5	685
bta-miR-124b	-16.0	231
bta-miR-124b	-15.7	299
bta-miR-124b	-15.3	270
bta-miR-124b	-14.6	400
bta-miR-124b	-13.9	1
bta-miR-124b	-13.7	594
bta-miR-124b	-13.4	739
bta-miR-124b	-13.2	144
bta-miR-124b	-12.0	199
bta-miR-124b	-11.7	708
bta-miR-124b	-11.6	315
bta-miR-124b	-11.6	532
bta-miR-124b	-11.5	631
bta-miR-124b	-11.4	565
bta-miR-124b	-11.3	384
bta-miR-124b	-10.7	326
bta-miR-124b	-10.4	448
bta-miR-129	-26.1	8
bta-miR-129	-24.9	155
bta-miR-129	-24.6	424
bta-miR-129	-23.9	108
bta-miR-129	-23.2	233
bta-miR-129	-22.4	464
bta-miR-129	-22.1	358
bta-miR-129	-20.7	597
bta-miR-129	-20.5	65
bta-miR-129	-20.5	270
bta-miR-129	-20.2	123
bta-miR-129	-19.9	184
bta-miR-129	-19.7	406

bta-miR-129	-19.3	559
bta-miR-129	-18.3	326
bta-miR-129	-17.1	206
bta-miR-129	-16.8	617
bta-miR-129	-16.3	30
bta-miR-129	-16.1	97
bta-miR-129	-15.9	494
bta-miR-129	-15.6	514
bta-miR-129	-13.7	575
bta-miR-129	-13.4	135
bta-miR-129	-13.4	308
bta-miR-129	-13.2	736
bta-miR-129	-12.7	377
bta-miR-129	-12.5	663
bta-miR-129	-12.1	532
bta-miR-129	-11.1	389
bta-miR-129	-11.1	455
bta-miR-129	-10.7	252
bta-miR-129	-10.3	586
bta-miR-129-5p	-26.1	8
bta-miR-129-5p	-24.9	155
bta-miR-129-5p	-24.6	424
bta-miR-129-5p	-23.9	108
bta-miR-129-5p	-23.2	233
bta-miR-129-5p	-22.4	464
bta-miR-129-5p	-22.1	358
bta-miR-129-5p	-20.7	597
bta-miR-129-5p	-20.5	65
bta-miR-129-5p	-20.5	270
bta-miR-129-5p	-20.2	123
bta-miR-129-5p	-19.9	184
bta-miR-129-5p	-19.7	406
bta-miR-129-5p	-19.3	559
bta-miR-129-5p	-18.3	326
bta-miR-129-5p	-17.1	206
bta-miR-129-5p	-16.8	617
bta-miR-129-5p	-16.3	30
bta-miR-129-5p	-16.1	97
bta-miR-129-5p	-15.9	494
bta-miR-129-5p	-15.6	514
bta-miR-129-5p	-13.7	575
bta-miR-129-5p	-13.4	135
bta-miR-129-5p	-13.4	308
bta-miR-129-5p	-13.2	736
bta-miR-129-5p	-12.7	377
bta-miR-129-5p	-12.5	663
bta-miR-129-5p	-12.1	532
bta-miR-129-5p	-11.1	389
bta-miR-129-5p	-11.1	455
bta-miR-129-5p	-10.7	252
bta-miR-129-5p	-10.3	586
bta-miR-129-3p	-31.1	606
bta-miR-129-3p	-23.7	66
bta-miR-129-3p	-22.2	448
bta-miR-129-3p	-21.0	256
bta-miR-129-3p	-19.9	408
bta-miR-129-3p	-18.9	628
bta-miR-129-3p	-18.0	473
bta-miR-129-3p	-16.0	13

bta-miR-129-3p	-16.0	497
bta-miR-129-3p	-14.7	95
bta-miR-129-3p	-14.1	538
bta-miR-129-3p	-13.7	584
bta-miR-129-3p	-13.7	172
bta-miR-129-3p	-12.9	359
bta-miR-129-3p	-12.5	231
bta-miR-129-3p	-12.5	654
bta-miR-129-3p	-11.9	697
bta-miR-129-3p	-11.2	30
bta-miR-129-3p	-11.1	152
bta-miR-129-3p	-10.6	206
bta-miR-129-3p	-10.2	562
bta-miR-129-3p	-10.2	729
bta-miR-130a	-27.7	359
bta-miR-130a	-20.6	109
bta-miR-130a	-20.6	473
bta-miR-130a	-19.8	624
bta-miR-130a	-19.8	408
bta-miR-130a	-18.8	167
bta-miR-130a	-18.4	51
bta-miR-130a	-18.4	428
bta-miR-130a	-17.5	562
bta-miR-130a	-17.4	244
bta-miR-130a	-17.0	3
bta-miR-130a	-17.0	518
bta-miR-130a	-15.9	191
bta-miR-130a	-15.2	668
bta-miR-130a	-15.0	590
bta-miR-130a	-14.7	30
bta-miR-130a	-14.5	126
bta-miR-130a	-14.4	306
bta-miR-130a	-13.7	653
bta-miR-130a	-13.6	716
bta-miR-130a	-13.2	88
bta-miR-130a	-12.9	336
bta-miR-130a	-12.8	95
bta-miR-130a	-12.6	141
bta-miR-130a	-12.6	692
bta-miR-130a	-12.1	283
bta-miR-130a	-11.7	538
bta-miR-130a	-11.1	235
bta-miR-130a	-10.4	388
bta-miR-130b	-23.4	359
bta-miR-130b	-21.2	483
bta-miR-130b	-19.1	562
bta-miR-130b	-19.0	167
bta-miR-130b	-18.5	633
bta-miR-130b	-18.3	518
bta-miR-130b	-18.1	88
bta-miR-130b	-18.1	408
bta-miR-130b	-17.9	428
bta-miR-130b	-17.9	244
bta-miR-130b	-17.2	3
bta-miR-130b	-16.9	30
bta-miR-130b	-16.9	283
bta-miR-130b	-16.6	668
bta-miR-130b	-16.5	51
bta-miR-130b	-16.0	142

bta-miR-130b	-14.3	260
bta-miR-130b	-13.7	191
bta-miR-130b	-13.5	697
bta-miR-130b	-12.9	388
bta-miR-130b	-12.7	624
bta-miR-130b	-12.4	715
bta-miR-130b	-12.4	457
bta-miR-130b	-11.9	590
bta-miR-130b	-11.8	119
bta-miR-130b	-11.1	235
bta-miR-130b	-11.0	544
bta-miR-130b	-10.1	743
bta-miR-130b	-10.0	336
bta-miR-133a	-27.5	456
bta-miR-133a	-25.2	419
bta-miR-133a	-25.1	605
bta-miR-133a	-23.6	64
bta-miR-133a	-19.7	12
bta-miR-133a	-19.2	559
bta-miR-133a	-18.6	495
bta-miR-133a	-17.9	107
bta-miR-133a	-17.8	586
bta-miR-133a	-17.1	38
bta-miR-133a	-16.5	165
bta-miR-133a	-15.9	219
bta-miR-133a	-15.9	737
bta-miR-133a	-15.5	630
bta-miR-133a	-15.4	653
bta-miR-133a	-14.5	243
bta-miR-133a	-14.5	358
bta-miR-133a	-12.0	404
bta-miR-133a	-11.1	93
bta-miR-133a	-10.8	698
bta-miR-133a	-10.6	337
bta-miR-133a	-10.2	344
bta-miR-133b	-27.5	456
bta-miR-133b	-25.2	419
bta-miR-133b	-25.1	605
bta-miR-133b	-23.9	64
bta-miR-133b	-19.7	12
bta-miR-133b	-19.2	559
bta-miR-133b	-17.8	586
bta-miR-133b	-17.6	494
bta-miR-133b	-17.1	38
bta-miR-133b	-16.3	108
bta-miR-133b	-15.9	219
bta-miR-133b	-15.9	737
bta-miR-133b	-15.5	630
bta-miR-133b	-15.4	653
bta-miR-133b	-15.1	166
bta-miR-133b	-14.5	243
bta-miR-133b	-14.5	358
bta-miR-133b	-12.2	393
bta-miR-133b	-11.1	93
bta-miR-133b	-10.8	698
bta-miR-133b	-10.6	337
bta-miR-133b	-10.2	344
bta-miR-134	-23.6	382
bta-miR-134	-23.3	468

bta-miR-134	-21.5	34
bta-miR-134	-21.4	136
bta-miR-134	-20.8	2
bta-miR-134	-19.6	109
bta-miR-134	-19.4	657
bta-miR-134	-19.3	581
bta-miR-134	-18.4	624
bta-miR-134	-17.6	260
bta-miR-134	-17.4	549
bta-miR-134	-16.8	502
bta-miR-134	-16.5	331
bta-miR-134	-16.5	416
bta-miR-134	-16.3	73
bta-miR-134	-15.3	738
bta-miR-134	-15.1	189
bta-miR-134	-14.3	156
bta-miR-134	-14.2	367
bta-miR-134	-14.0	210
bta-miR-134	-12.8	520
bta-miR-134	-12.7	454
bta-miR-134	-12.2	690
bta-miR-134	-12.2	59
bta-miR-134	-11.7	246
bta-miR-134	-11.5	572
bta-miR-134	-11.0	309
bta-miR-134	-10.3	290
bta-miR-135a	-19.5	232
bta-miR-135a	-19.2	22
bta-miR-135a	-18.9	172
bta-miR-135a	-18.8	447
bta-miR-135a	-17.7	606
bta-miR-135a	-17.4	147
bta-miR-135a	-17.2	106
bta-miR-135a	-16.9	66
bta-miR-135a	-16.9	382
bta-miR-135a	-15.9	263
bta-miR-135a	-15.6	467
bta-miR-135a	-15.3	415
bta-miR-135a	-15.2	654
bta-miR-135a	-14.9	206
bta-miR-135a	-14.5	492
bta-miR-135a	-13.7	731
bta-miR-135a	-12.7	555
bta-miR-135a	-12.0	567
bta-miR-135a	-11.9	345
bta-miR-135a	-11.5	299
bta-miR-135a	-11.3	630
bta-miR-135a	-10.5	594
bta-miR-135a	-10.1	364
bta-miR-135a	-10.1	434
bta-miR-135b	-21.5	447
bta-miR-135b	-20.4	606
bta-miR-135b	-19.5	22
bta-miR-135b	-18.3	147
bta-miR-135b	-18.2	172
bta-miR-135b	-18.1	206
bta-miR-135b	-18.1	106
bta-miR-135b	-17.4	66
bta-miR-135b	-17.3	654

bta-miR-135b	-16.9	382
bta-miR-135b	-16.6	492
bta-miR-135b	-15.9	263
bta-miR-135b	-15.3	415
bta-miR-135b	-14.9	467
bta-miR-135b	-14.1	345
bta-miR-135b	-13.5	630
bta-miR-135b	-12.5	567
bta-miR-135b	-12.2	554
bta-miR-135b	-11.8	738
bta-miR-135b	-11.1	7
bta-miR-135b	-10.5	299
bta-miR-135b	-10.2	598
bta-miR-135b	-10.1	364
bta-miR-135b	-10.1	434
bta-miR-136	-22.6	52
bta-miR-136	-21.6	209
bta-miR-136	-21.0	596
bta-miR-136	-21.0	259
bta-miR-136	-20.6	461
bta-miR-136	-19.6	342
bta-miR-136	-19.3	431
bta-miR-136	-18.4	484
bta-miR-136	-16.1	391
bta-miR-136	-16.0	191
bta-miR-136	-15.9	98
bta-miR-136	-15.8	5
bta-miR-136	-15.4	639
bta-miR-136	-15.1	164
bta-miR-136	-14.3	363
bta-miR-136	-14.1	572
bta-miR-136	-13.4	23
bta-miR-136	-12.9	325
bta-miR-136	-12.7	629
bta-miR-136	-12.6	292
bta-miR-136	-12.3	715
bta-miR-136	-11.9	143
bta-miR-136	-11.3	451
bta-miR-136	-11.3	692
bta-miR-136	-11.0	564
bta-miR-136	-10.4	120
bta-miR-136	-10.4	454
bta-miR-136	-10.1	236
bta-miR-136	-10.0	247
bta-miR-137	-19.8	627
bta-miR-137	-17.4	14
bta-miR-137	-16.9	543
bta-miR-137	-16.4	39
bta-miR-137	-15.6	493
bta-miR-137	-14.0	458
bta-miR-137	-13.8	521
bta-miR-137	-13.5	594
bta-miR-137	-13.5	686
bta-miR-137	-13.1	69
bta-miR-137	-13.1	185
bta-miR-137	-13.1	658
bta-miR-137	-12.7	256
bta-miR-137	-12.7	377
bta-miR-137	-12.2	708

bta-miR-137	-11.6	206
bta-miR-137	-11.1	421
bta-miR-137	-11.0	341
bta-miR-137	-11.0	477
bta-miR-137	-10.9	614
bta-miR-137	-10.8	112
bta-miR-137	-10.8	561
bta-miR-137	-10.5	277
bta-miR-137	-10.2	305
bta-miR-141	-20.7	4
bta-miR-141	-20.3	342
bta-miR-141	-19.7	474
bta-miR-141	-18.9	384
bta-miR-141	-18.3	501
bta-miR-141	-17.9	564
bta-miR-141	-17.1	120
bta-miR-141	-17.0	23
bta-miR-141	-17.0	52
bta-miR-141	-16.0	639
bta-miR-141	-16.0	237
bta-miR-141	-14.8	87
bta-miR-141	-14.5	258
bta-miR-141	-14.1	416
bta-miR-141	-13.3	716
bta-miR-141	-13.3	613
bta-miR-141	-13.2	665
bta-miR-141	-13.1	143
bta-miR-141	-13.1	210
bta-miR-141	-12.6	532
bta-miR-141	-12.3	191
bta-miR-141	-12.3	164
bta-miR-141	-12.0	692
bta-miR-141	-11.9	681
bta-miR-141	-11.7	74
bta-miR-141	-11.6	428
bta-miR-141	-11.6	457
bta-miR-141	-11.0	109
bta-miR-141	-10.9	299
bta-miR-141	-10.6	624
bta-miR-141	-10.3	228
bta-miR-143	-21.8	622
bta-miR-143	-21.5	506
bta-miR-143	-20.5	481
bta-miR-143	-20.2	356
bta-miR-143	-20.1	269
bta-miR-143	-19.2	455
bta-miR-143	-18.8	559
bta-miR-143	-18.5	12
bta-miR-143	-17.4	114
bta-miR-143	-17.2	71
bta-miR-143	-17.0	184
bta-miR-143	-15.7	597
bta-miR-143	-15.6	407
bta-miR-143	-15.6	242
bta-miR-143	-14.2	653
bta-miR-143	-13.4	539
bta-miR-143	-12.8	388
bta-miR-143	-12.1	155
bta-miR-143	-12.0	218

bta-miR-143	-12.0	582
bta-miR-143	-11.9	609
bta-miR-143	-11.7	698
bta-miR-143	-11.7	434
bta-miR-143	-11.1	738
bta-miR-143	-10.8	293
bta-miR-143	-10.8	344
bta-miR-143	-10.0	50
bta-miR-144	-19.5	379
bta-miR-144	-17.8	203
bta-miR-144	-17.7	40
bta-miR-144	-17.6	517
bta-miR-144	-17.0	575
bta-miR-144	-16.8	1
bta-miR-144	-15.3	544
bta-miR-144	-14.7	80
bta-miR-144	-13.9	477
bta-miR-144	-13.4	632
bta-miR-144	-13.1	294
bta-miR-144	-12.9	411
bta-miR-144	-12.8	358
bta-miR-144	-12.2	106
bta-miR-144	-11.3	395
bta-miR-144	-11.3	19
bta-miR-144	-11.2	662
bta-miR-144	-10.9	172
bta-miR-144	-10.8	565
bta-miR-144	-10.7	267
bta-miR-144	-10.0	149
bta-miR-146b	-24.9	165
bta-miR-146b	-23.1	472
bta-miR-146b	-21.4	602
bta-miR-146b	-19.7	358
bta-miR-146b	-19.1	424
bta-miR-146b	-18.5	106
bta-miR-146b	-17.9	261
bta-miR-146b	-17.8	224
bta-miR-146b	-16.9	560
bta-miR-146b	-16.8	407
bta-miR-146b	-16.7	72
bta-miR-146b	-16.6	698
bta-miR-146b	-16.2	29
bta-miR-146b	-15.9	444
bta-miR-146b	-15.8	653
bta-miR-146b	-15.5	308
bta-miR-146b	-14.8	516
bta-miR-146b	-13.9	629
bta-miR-146b	-13.4	10
bta-miR-146b	-12.5	581
bta-miR-146b	-11.4	198
bta-miR-146b	-10.9	243
bta-miR-146b	-10.1	215
bta-miR-146a	-22.5	165
bta-miR-146a	-20.7	472
bta-miR-146a	-20.1	700
bta-miR-146a	-19.3	366
bta-miR-146a	-19.2	602
bta-miR-146a	-18.1	261
bta-miR-146a	-16.7	225

bta-miR-146a	-16.6	424
bta-miR-146a	-16.1	106
bta-miR-146a	-15.2	654
bta-miR-146a	-15.0	454
bta-miR-146a	-14.6	560
bta-miR-146a	-14.4	407
bta-miR-146a	-14.3	509
bta-miR-146a	-14.2	72
bta-miR-146a	-13.9	629
bta-miR-146a	-13.8	29
bta-miR-146a	-13.7	308
bta-miR-146a	-13.0	340
bta-miR-146a	-12.7	581
bta-miR-146a	-11.4	206
bta-miR-146a	-11.2	523
bta-miR-146a	-11.2	10
bta-miR-147	-23.4	198
bta-miR-147	-23.4	450
bta-miR-147	-21.2	171
bta-miR-147	-21.0	278
bta-miR-147	-20.5	64
bta-miR-147	-19.6	100
bta-miR-147	-19.6	482
bta-miR-147	-18.8	555
bta-miR-147	-18.6	407
bta-miR-147	-18.3	653
bta-miR-147	-17.6	617
bta-miR-147	-17.4	361
bta-miR-147	-16.6	12
bta-miR-147	-16.6	496
bta-miR-147	-15.8	125
bta-miR-147	-14.5	37
bta-miR-147	-14.3	265
bta-miR-147	-13.7	219
bta-miR-147	-13.6	586
bta-miR-147	-13.6	605
bta-miR-147	-12.4	698
bta-miR-147	-11.8	85
bta-miR-147	-11.8	238
bta-miR-147	-11.6	342
bta-miR-147	-11.2	471
bta-miR-147	-10.8	731
bta-miR-147	-10.6	154
bta-miR-147	-10.6	308
bta-miR-152	-24.0	572
bta-miR-152	-22.0	33
bta-miR-152	-20.7	5
bta-miR-152	-20.3	409
bta-miR-152	-19.8	362
bta-miR-152	-19.6	467
bta-miR-152	-19.5	77
bta-miR-152	-18.5	514
bta-miR-152	-17.8	247
bta-miR-152	-17.5	276
bta-miR-152	-17.0	539
bta-miR-152	-16.9	637
bta-miR-152	-16.5	384
bta-miR-152	-16.3	110
bta-miR-152	-15.7	163

bta-miR-152	-15.6	59
bta-miR-152	-14.8	596
bta-miR-152	-14.3	660
bta-miR-152	-13.9	135
bta-miR-152	-13.6	18
bta-miR-152	-13.4	330
bta-miR-152	-13.1	209
bta-miR-152	-11.8	552
bta-miR-152	-11.7	199
bta-miR-152	-11.5	740
bta-miR-152	-11.1	616
bta-miR-152	-10.3	691
bta-miR-153	-21.6	537
bta-miR-153	-19.2	4
bta-miR-153	-17.6	632
bta-miR-153	-17.4	54
bta-miR-153	-17.2	412
bta-miR-153	-15.4	354
bta-miR-153	-13.8	286
bta-miR-153	-13.7	258
bta-miR-153	-13.7	679
bta-miR-153	-13.5	489
bta-miR-153	-13.5	738
bta-miR-153	-13.3	520
bta-miR-153	-13.1	566
bta-miR-153	-12.9	147
bta-miR-153	-12.7	660
bta-miR-153	-11.9	77
bta-miR-153	-11.7	187
bta-miR-153	-11.4	589
bta-miR-153	-10.9	25
bta-miR-153	-10.6	702
bta-miR-153	-10.6	113
bta-miR-154	-30.9	620
bta-miR-154	-24.4	452
bta-miR-154	-24.0	479
bta-miR-154	-23.1	405
bta-miR-154	-22.8	69
bta-miR-154	-20.9	153
bta-miR-154	-20.5	560
bta-miR-154	-20.2	11
bta-miR-154	-20.1	114
bta-miR-154	-18.5	220
bta-miR-154	-17.7	698
bta-miR-154	-17.1	348
bta-miR-154	-16.7	591
bta-miR-154	-16.7	268
bta-miR-154	-13.2	38
bta-miR-154	-12.9	738
bta-miR-154	-12.2	372
bta-miR-154	-12.2	654
bta-miR-154	-12.1	445
bta-miR-154	-10.8	574
bta-miR-154	-10.8	294
bta-miR-154	-10.1	312
bta-miR-155	-20.8	361
bta-miR-155	-19.5	473
bta-miR-155	-19.5	162
bta-miR-155	-19.2	189

bta-miR-155	-19.1	30
bta-miR-155	-18.9	247
bta-miR-155	-18.2	579
bta-miR-155	-17.7	3
bta-miR-155	-17.7	636
bta-miR-155	-17.5	141
bta-miR-155	-17.1	698
bta-miR-155	-16.8	327
bta-miR-155	-16.2	400
bta-miR-155	-15.8	429
bta-miR-155	-15.4	109
bta-miR-155	-14.4	458
bta-miR-155	-14.4	210
bta-miR-155	-13.4	320
bta-miR-155	-13.3	510
bta-miR-155	-13.2	87
bta-miR-155	-12.9	283
bta-miR-155	-12.5	134
bta-miR-155	-11.9	341
bta-miR-155	-11.5	560
bta-miR-155	-11.3	624
bta-miR-155	-10.9	416
bta-miR-155	-10.8	381
bta-miR-155	-10.3	537
bta-miR-16a	-20.8	571
bta-miR-16a	-19.5	408
bta-miR-16a	-17.6	483
bta-miR-16a	-17.1	515
bta-miR-16a	-16.7	729
bta-miR-16a	-16.5	34
bta-miR-16a	-16.4	73
bta-miR-16a	-16.3	669
bta-miR-16a	-15.0	9
bta-miR-16a	-14.9	99
bta-miR-16a	-14.9	367
bta-miR-16a	-14.5	253
bta-miR-16a	-13.9	560
bta-miR-16a	-13.7	161
bta-miR-16a	-13.3	594
bta-miR-16a	-12.9	341
bta-miR-16a	-12.5	649
bta-miR-16a	-11.9	454
bta-miR-16a	-11.3	283
bta-miR-16a	-10.7	52
bta-miR-16a	-10.6	126
bta-miR-181d	-22.2	324
bta-miR-181d	-21.6	162
bta-miR-181d	-21.5	361
bta-miR-181d	-21.3	594
bta-miR-181d	-20.9	473
bta-miR-181d	-17.5	425
bta-miR-181d	-17.2	208
bta-miR-181d	-17.0	109
bta-miR-181d	-16.9	397
bta-miR-181d	-16.9	1
bta-miR-181d	-16.9	341
bta-miR-181d	-16.9	563
bta-miR-181d	-16.8	510
bta-miR-181d	-16.3	244

bta-miR-181d	-15.8	30
bta-miR-181d	-15.7	51
bta-miR-181d	-15.1	185
bta-miR-181d	-15.1	135
bta-miR-181d	-14.0	624
bta-miR-181d	-13.5	317
bta-miR-181d	-13.2	702
bta-miR-181d	-12.8	86
bta-miR-181d	-11.6	586
bta-miR-181d	-11.4	126
bta-miR-181d	-11.1	308
bta-miR-181d	-11.0	275
bta-miR-181d	-11.0	458
bta-miR-181d	-10.9	534
bta-miR-181d	-10.1	666
bta-miR-182	-25.6	544
bta-miR-182	-24.7	659
bta-miR-182	-24.4	376
bta-miR-182	-22.9	113
bta-miR-182	-21.4	17
bta-miR-182	-20.9	81
bta-miR-182	-20.1	447
bta-miR-182	-19.3	613
bta-miR-182	-19.1	265
bta-miR-182	-18.9	496
bta-miR-182	-16.8	344
bta-miR-182	-16.5	584
bta-miR-182	-16.4	422
bta-miR-182	-16.2	231
bta-miR-182	-15.7	473
bta-miR-182	-14.6	691
bta-miR-182	-14.5	313
bta-miR-182	-14.0	730
bta-miR-182	-14.0	524
bta-miR-182	-12.7	1
bta-miR-182	-12.3	146
bta-miR-182	-11.9	162
bta-miR-182	-11.8	247
bta-miR-182	-11.5	51
bta-miR-182	-11.4	174
bta-miR-182	-11.0	59
bta-miR-182	-10.8	202
bta-miR-183	-26.3	495
bta-miR-183	-21.9	376
bta-miR-183	-21.6	447
bta-miR-183	-21.3	107
bta-miR-183	-19.6	632
bta-miR-183	-19.5	17
bta-miR-183	-19.2	80
bta-miR-183	-19.0	730
bta-miR-183	-18.8	575
bta-miR-183	-18.1	264
bta-miR-183	-18.0	544
bta-miR-183	-17.8	347
bta-miR-183	-17.7	313
bta-miR-183	-17.5	1
bta-miR-183	-17.1	228
bta-miR-183	-16.8	588
bta-miR-183	-15.7	473

bta-miR-183	-14.9	420
bta-miR-183	-14.7	686
bta-miR-183	-14.6	295
bta-miR-183	-14.2	59
bta-miR-183	-13.8	175
bta-miR-183	-13.7	145
bta-miR-183	-13.2	665
bta-miR-183	-13.0	607
bta-miR-183	-12.7	164
bta-miR-183	-12.1	51
bta-miR-183	-11.5	427
bta-miR-183	-10.6	709
bta-miR-183	-10.5	249
bta-miR-183	-10.2	200
bta-miR-183	-10.2	337
bta-miR-183	-10.0	327
bta-miR-184	-21.4	73
bta-miR-184	-21.3	247
bta-miR-184	-20.8	361
bta-miR-184	-20.6	510
bta-miR-184	-19.9	429
bta-miR-184	-19.8	540
bta-miR-184	-18.5	109
bta-miR-184	-18.2	30
bta-miR-184	-18.0	191
bta-miR-184	-17.9	571
bta-miR-184	-17.7	624
bta-miR-184	-17.1	401
bta-miR-184	-16.4	327
bta-miR-184	-16.1	483
bta-miR-184	-15.3	649
bta-miR-184	-15.3	126
bta-miR-184	-14.7	7
bta-miR-184	-14.7	698
bta-miR-184	-14.6	277
bta-miR-184	-13.3	96
bta-miR-184	-13.1	214
bta-miR-184	-12.6	54
bta-miR-184	-12.3	682
bta-miR-184	-12.0	167
bta-miR-184	-11.2	261
bta-miR-184	-11.2	475
bta-miR-184	-11.0	298
bta-miR-185	-26.7	277
bta-miR-185	-25.9	83
bta-miR-185	-22.7	107
bta-miR-185	-22.6	559
bta-miR-185	-22.1	450
bta-miR-185	-22.0	660
bta-miR-185	-20.7	176
bta-miR-185	-19.7	597
bta-miR-185	-19.7	238
bta-miR-185	-18.5	9
bta-miR-185	-18.4	369
bta-miR-185	-18.4	479
bta-miR-185	-18.1	299
bta-miR-185	-17.5	200
bta-miR-185	-17.4	67
bta-miR-185	-17.2	421

bta-miR-185	-15.9	710
bta-miR-185	-15.5	631
bta-miR-185	-14.0	685
bta-miR-185	-13.9	575
bta-miR-185	-13.2	410
bta-miR-185	-13.0	354
bta-miR-185	-12.8	51
bta-miR-185	-11.9	1
bta-miR-185	-11.7	738
bta-miR-185	-11.7	139
bta-miR-185	-11.7	154
bta-miR-185	-11.5	263
bta-miR-185	-11.1	519
bta-miR-185	-11.1	473
bta-miR-185	-10.7	37
bta-miR-185	-10.7	529
bta-miR-185	-10.5	219
bta-miR-185	-10.0	697
bta-miR-187	-25.9	115
bta-miR-187	-22.5	342
bta-miR-187	-22.3	462
bta-miR-187	-21.9	582
bta-miR-187	-21.6	61
bta-miR-187	-21.1	619
bta-miR-187	-20.5	402
bta-miR-187	-20.3	155
bta-miR-187	-19.1	12
bta-miR-187	-18.5	267
bta-miR-187	-18.4	560
bta-miR-187	-17.6	85
bta-miR-187	-17.1	740
bta-miR-187	-16.8	488
bta-miR-187	-16.7	362
bta-miR-187	-16.4	432
bta-miR-187	-16.1	185
bta-miR-187	-14.6	33
bta-miR-187	-13.3	696
bta-miR-187	-12.4	292
bta-miR-187	-12.0	643
bta-miR-187	-11.9	211
bta-miR-187	-11.8	662
bta-miR-187	-10.9	231
bta-miR-188	-31.3	162
bta-miR-188	-28.1	30
bta-miR-188	-26.0	578
bta-miR-188	-25.6	474
bta-miR-188	-25.2	398
bta-miR-188	-21.6	510
bta-miR-188	-21.5	244
bta-miR-188	-21.4	320
bta-miR-188	-21.2	109
bta-miR-188	-21.0	136
bta-miR-188	-21.0	425
bta-miR-188	-20.2	208
bta-miR-188	-18.8	1
bta-miR-188	-18.4	361
bta-miR-188	-17.7	624
bta-miR-188	-17.4	563
bta-miR-188	-17.0	637

bta-miR-188	-15.8	86
bta-miR-188	-15.1	692
bta-miR-188	-14.8	341
bta-miR-188	-13.6	185
bta-miR-188	-13.6	457
bta-miR-188	-13.2	536
bta-miR-188	-12.4	665
bta-miR-188	-12.3	715
bta-miR-188	-11.8	292
bta-miR-188	-11.1	309
bta-miR-188	-11.0	271
bta-miR-188	-10.5	20
bta-miR-188	-10.4	384
bta-miR-188	-10.2	73
bta-miR-24	-25.3	1
bta-miR-24	-24.4	256
bta-miR-24	-24.3	92
bta-miR-24	-22.7	403
bta-miR-24	-22.7	617
bta-miR-24	-21.8	39
bta-miR-24	-21.7	586
bta-miR-24	-18.9	172
bta-miR-24	-18.9	364
bta-miR-24	-18.4	477
bta-miR-24	-16.2	229
bta-miR-24	-16.1	560
bta-miR-24	-15.9	432
bta-miR-24	-14.3	739
bta-miR-24	-14.3	117
bta-miR-24	-13.2	80
bta-miR-24	-13.2	276
bta-miR-24	-13.2	536
bta-miR-24	-13.2	385
bta-miR-24	-13.1	643
bta-miR-24	-12.3	504
bta-miR-24	-12.2	144
bta-miR-24	-11.5	459
bta-miR-24	-11.4	292
bta-miR-24	-10.6	337
bta-miR-24	-10.0	165
bta-miR-190a	-15.9	91
bta-miR-190a	-15.7	34
bta-miR-190a	-15.5	473
bta-miR-190a	-14.5	261
bta-miR-190a	-14.1	341
bta-miR-190a	-13.6	367
bta-miR-190a	-13.1	13
bta-miR-190a	-13.0	298
bta-miR-190a	-12.9	520
bta-miR-190a	-12.0	595
bta-miR-190a	-11.8	111
bta-miR-190a	-11.0	192
bta-miR-190a	-10.9	639
bta-miR-190a	-10.7	431
bta-miR-190a	-10.6	563
bta-miR-190a	-10.4	402
bta-miR-190a	-10.2	544
bta-miR-190b	-19.4	29
bta-miR-190b	-17.0	262

bta-miR-190b	-16.6	91
bta-miR-190b	-15.7	472
bta-miR-190b	-15.5	330
bta-miR-190b	-15.4	243
bta-miR-190b	-15.4	12
bta-miR-190b	-14.7	142
bta-miR-190b	-14.6	166
bta-miR-190b	-14.3	596
bta-miR-190b	-14.3	119
bta-miR-190b	-13.9	361
bta-miR-190b	-13.0	406
bta-miR-190b	-12.7	429
bta-miR-190b	-12.7	514
bta-miR-190b	-12.0	297
bta-miR-190b	-12.0	188
bta-miR-190b	-11.7	572
bta-miR-190b	-11.3	639
bta-miR-190b	-10.9	108
bta-miR-190b	-10.3	135
bta-miR-190b	-10.3	324
bta-miR-190b	-10.1	460
bta-miR-193b	-29.8	65
bta-miR-193b	-28.0	11
bta-miR-193b	-26.8	405
bta-miR-193b	-26.7	604
bta-miR-193b	-24.8	445
bta-miR-193b	-22.8	108
bta-miR-193b	-22.3	265
bta-miR-193b	-21.6	496
bta-miR-193b	-19.6	652
bta-miR-193b	-18.2	357
bta-miR-193b	-17.2	203
bta-miR-193b	-15.9	171
bta-miR-193b	-14.6	520
bta-miR-193b	-14.4	557
bta-miR-193b	-13.9	41
bta-miR-193b	-13.1	597
bta-miR-193b	-12.5	541
bta-miR-193b	-12.2	738
bta-miR-193b	-11.7	155
bta-miR-193b	-11.3	96
bta-miR-193b	-11.2	241
bta-miR-193b	-10.7	698
bta-miR-193b	-10.0	388
bta-miR-194	-19.5	467
bta-miR-194	-18.7	409
bta-miR-194	-18.3	606
bta-miR-194	-18.1	431
bta-miR-194	-17.9	60
bta-miR-194	-17.3	228
bta-miR-194	-17.3	90
bta-miR-194	-17.2	363
bta-miR-194	-17.1	572
bta-miR-194	-16.8	22
bta-miR-194	-16.1	169
bta-miR-194	-16.0	655
bta-miR-194	-14.4	739
bta-miR-194	-13.7	214
bta-miR-194	-13.3	146

bta-miR-194	-13.3	514
bta-miR-194	-12.9	543
bta-miR-194	-12.8	594
bta-miR-194	-12.5	353
bta-miR-194	-12.2	280
bta-miR-194	-12.0	5
bta-miR-194	-11.6	325
bta-miR-194	-11.0	191
bta-miR-194	-11.0	497
bta-miR-194	-11.0	317
bta-miR-194	-10.6	704
bta-miR-194	-10.4	560
bta-miR-194	-10.2	136
bta-miR-194	-10.2	696
bta-miR-194	-10.1	401
bta-miR-196a	-22.5	98
bta-miR-196a	-21.8	168
bta-miR-196a	-21.1	342
bta-miR-196a	-20.0	60
bta-miR-196a	-19.0	142
bta-miR-196a	-18.8	409
bta-miR-196a	-16.8	484
bta-miR-196a	-16.5	596
bta-miR-196a	-16.5	650
bta-miR-196a	-15.9	430
bta-miR-196a	-15.8	260
bta-miR-196a	-15.4	555
bta-miR-196a	-15.2	192
bta-miR-196a	-15.1	33
bta-miR-196a	-15.0	9
bta-miR-196a	-14.7	330
bta-miR-196a	-14.5	699
bta-miR-196a	-14.2	299
bta-miR-196a	-13.8	514
bta-miR-196a	-13.7	272
bta-miR-196a	-13.0	734
bta-miR-196a	-12.9	372
bta-miR-196a	-12.5	572
bta-miR-196a	-12.1	236
bta-miR-196a	-11.1	462
bta-miR-196a	-10.9	123
bta-miR-196a	-10.4	215
bta-miR-196b	-26.4	168
bta-miR-196b	-25.2	98
bta-miR-196b	-23.9	59
bta-miR-196b	-22.0	401
bta-miR-196b	-22.0	259
bta-miR-196b	-21.4	596
bta-miR-196b	-20.8	430
bta-miR-196b	-20.6	191
bta-miR-196b	-20.0	142
bta-miR-196b	-19.4	484
bta-miR-196b	-19.2	330
bta-miR-196b	-18.3	567
bta-miR-196b	-18.0	715
bta-miR-196b	-16.6	33
bta-miR-196b	-15.8	650
bta-miR-196b	-14.8	5
bta-miR-196b	-14.1	372

bta-miR-196b	-13.9	214
bta-miR-196b	-13.7	466
bta-miR-196b	-13.5	558
bta-miR-196b	-12.9	631
bta-miR-196b	-12.8	513
bta-miR-196b	-11.7	681
bta-miR-196b	-11.4	299
bta-miR-196b	-11.4	133
bta-miR-196b	-10.8	324
bta-miR-197	-31.3	616
bta-miR-197	-27.7	420
bta-miR-197	-26.7	13
bta-miR-197	-20.0	477
bta-miR-197	-18.7	66
bta-miR-197	-18.0	586
bta-miR-197	-17.4	216
bta-miR-197	-15.7	364
bta-miR-197	-14.4	266
bta-miR-197	-13.0	560
bta-miR-197	-12.8	344
bta-miR-197	-12.7	112
bta-miR-197	-12.2	504
bta-miR-197	-11.9	405
bta-miR-197	-11.5	174
bta-miR-197	-11.4	657
bta-miR-197	-10.1	185
bta-miR-197	-10.1	84
bta-miR-197	-10.1	740
bta-miR-202	-21.7	612
bta-miR-202	-17.6	11
bta-miR-202	-16.2	442
bta-miR-202	-15.7	479
bta-miR-202	-15.3	68
bta-miR-202	-14.9	265
bta-miR-202	-14.3	172
bta-miR-202	-14.0	349
bta-miR-202	-14.0	405
bta-miR-202	-13.5	574
bta-miR-202	-13.2	377
bta-miR-202	-13.2	196
bta-miR-202	-11.9	517
bta-miR-202	-11.5	735
bta-miR-202	-11.4	304
bta-miR-202	-10.5	652
bta-miR-202	-10.4	543
bta-miR-202	-10.2	223
bta-miR-206	-20.0	381
bta-miR-206	-19.8	52
bta-miR-206	-19.7	185
bta-miR-206	-19.5	120
bta-miR-206	-18.7	299
bta-miR-206	-18.5	484
bta-miR-206	-18.2	84
bta-miR-206	-17.0	457
bta-miR-206	-15.3	636
bta-miR-206	-15.1	5
bta-miR-206	-15.1	277
bta-miR-206	-15.0	670
bta-miR-206	-14.8	237

bta-miR-206	-14.6	564
bta-miR-206	-14.3	698
bta-miR-206	-14.0	415
bta-miR-206	-13.6	363
bta-miR-206	-13.2	160
bta-miR-206	-11.1	321
bta-miR-206	-10.8	528
bta-miR-206	-10.1	69
bta-miR-206	-10.1	602
bta-miR-208a	-18.9	558
bta-miR-208a	-18.3	516
bta-miR-208a	-18.2	281
bta-miR-208a	-18.0	10
bta-miR-208a	-17.8	485
bta-miR-208a	-17.7	66
bta-miR-208a	-17.5	373
bta-miR-208a	-16.0	651
bta-miR-208a	-15.3	406
bta-miR-208a	-15.0	694
bta-miR-208a	-14.9	242
bta-miR-208a	-14.6	736
bta-miR-208a	-14.4	616
bta-miR-208a	-14.3	455
bta-miR-208a	-14.1	35
bta-miR-208a	-13.6	197
bta-miR-208a	-13.6	546
bta-miR-208a	-13.5	122
bta-miR-208a	-12.6	602
bta-miR-208a	-12.1	573
bta-miR-208a	-11.6	172
bta-miR-208a	-11.5	2
bta-miR-208a	-11.5	423
bta-miR-208a	-11.3	307
bta-miR-208a	-11.3	106
bta-miR-208a	-11.2	631
bta-miR-208a	-10.9	669
bta-miR-208a	-10.2	154
bta-miR-208b	-19.3	508
bta-miR-208b	-17.8	271
bta-miR-208b	-16.2	242
bta-miR-208b	-14.7	381
bta-miR-208b	-14.4	651
bta-miR-208b	-14.1	28
bta-miR-208b	-13.8	122
bta-miR-208b	-13.7	556
bta-miR-208b	-13.7	681
bta-miR-208b	-13.6	636
bta-miR-208b	-13.3	485
bta-miR-208b	-13.0	66
bta-miR-208b	-12.9	422
bta-miR-208b	-12.8	339
bta-miR-208b	-12.5	602
bta-miR-208b	-12.5	2
bta-miR-208b	-11.9	406
bta-miR-208b	-11.9	455
bta-miR-208b	-10.9	106
bta-miR-208b	-10.8	471
bta-miR-208b	-10.7	698
bta-miR-208b	-10.7	10

bta-miR-208b	-10.4	737
bta-miR-208b	-10.3	361
bta-miR-208b	-10.2	49
bta-miR-208b	-10.1	570
bta-miR-211	-24.1	605
bta-miR-211	-22.4	586
bta-miR-211	-20.9	66
bta-miR-211	-20.7	419
bta-miR-211	-19.9	206
bta-miR-211	-19.7	494
bta-miR-211	-18.8	172
bta-miR-211	-17.7	12
bta-miR-211	-16.0	555
bta-miR-211	-15.0	270
bta-miR-211	-14.0	464
bta-miR-211	-12.4	86
bta-miR-211	-11.7	344
bta-miR-211	-11.4	35
bta-miR-211	-11.3	654
bta-miR-211	-10.9	405
bta-miR-211	-10.8	199
bta-miR-211	-10.6	723
bta-miR-211	-10.3	452
bta-miR-212	-25.3	604
bta-miR-212	-25.2	558
bta-miR-212	-24.6	464
bta-miR-212	-22.4	651
bta-miR-212	-22.1	419
bta-miR-212	-21.8	197
bta-miR-212	-21.7	63
bta-miR-212	-21.4	376
bta-miR-212	-21.2	17
bta-miR-212	-21.1	232
bta-miR-212	-19.9	265
bta-miR-212	-19.0	494
bta-miR-212	-18.0	113
bta-miR-212	-17.6	154
bta-miR-212	-15.7	85
bta-miR-212	-14.9	454
bta-miR-212	-14.7	347
bta-miR-212	-14.7	44
bta-miR-212	-14.2	4
bta-miR-212	-14.0	737
bta-miR-212	-13.4	624
bta-miR-212	-12.7	687
bta-miR-212	-12.5	527
bta-miR-212	-11.8	586
bta-miR-212	-10.9	222
bta-miR-212	-10.6	514
bta-miR-216a	-24.9	421
bta-miR-216a	-21.4	66
bta-miR-216a	-20.4	476
bta-miR-216a	-20.3	161
bta-miR-216a	-19.3	7
bta-miR-216a	-19.3	573
bta-miR-216a	-18.9	112
bta-miR-216a	-17.8	559
bta-miR-216a	-17.3	199
bta-miR-216a	-17.3	606

bta-miR-216a	-16.7	408
bta-miR-216a	-16.2	22
bta-miR-216a	-16.2	263
bta-miR-216a	-16.0	354
bta-miR-216a	-15.4	739
bta-miR-216a	-15.1	534
bta-miR-216a	-14.9	182
bta-miR-216a	-14.7	342
bta-miR-216a	-14.5	514
bta-miR-216a	-14.3	382
bta-miR-216a	-14.3	232
bta-miR-216a	-14.2	663
bta-miR-216a	-13.9	299
bta-miR-216a	-13.7	461
bta-miR-216a	-13.4	696
bta-miR-216a	-12.5	445
bta-miR-216a	-12.4	147
bta-miR-216a	-11.8	45
bta-miR-216a	-11.3	96
bta-miR-216a	-11.3	643
bta-miR-216a	-11.0	326
bta-miR-216b	-22.6	416
bta-miR-216b	-21.6	55
bta-miR-216b	-20.6	488
bta-miR-216b	-19.1	587
bta-miR-216b	-19.1	185
bta-miR-216b	-18.9	2
bta-miR-216b	-18.8	161
bta-miR-216b	-18.0	353
bta-miR-216b	-17.1	206
bta-miR-216b	-16.7	473
bta-miR-216b	-15.9	112
bta-miR-216b	-15.5	299
bta-miR-216b	-15.3	235
bta-miR-216b	-14.9	147
bta-miR-216b	-14.5	551
bta-miR-216b	-14.2	23
bta-miR-216b	-13.6	88
bta-miR-216b	-13.6	616
bta-miR-216b	-13.6	663
bta-miR-216b	-13.2	534
bta-miR-216b	-13.1	373
bta-miR-216b	-12.6	257
bta-miR-216b	-12.4	692
bta-miR-216b	-12.3	445
bta-miR-216b	-12.2	73
bta-miR-216b	-11.8	740
bta-miR-216b	-11.7	514
bta-miR-216b	-11.6	408
bta-miR-216b	-10.9	271
bta-miR-216b	-10.9	344
bta-miR-216b	-10.7	581
bta-miR-216b	-10.3	483
bta-miR-216b	-10.2	383
bta-miR-217	-28.5	391
bta-miR-217	-24.0	460
bta-miR-217	-23.2	80
bta-miR-217	-22.2	572
bta-miR-217	-21.2	4

bta-miR-217	-19.5	52
bta-miR-217	-18.7	235
bta-miR-217	-17.9	670
bta-miR-217	-17.8	342
bta-miR-217	-17.6	511
bta-miR-217	-17.2	596
bta-miR-217	-16.9	169
bta-miR-217	-16.0	107
bta-miR-217	-15.9	208
bta-miR-217	-14.9	416
bta-miR-217	-14.6	34
bta-miR-217	-14.4	623
bta-miR-217	-13.2	532
bta-miR-217	-13.1	715
bta-miR-217	-13.0	650
bta-miR-217	-12.8	272
bta-miR-217	-11.5	499
bta-miR-217	-11.1	691
bta-miR-217	-11.0	136
bta-miR-217	-10.8	298
bta-miR-217	-10.2	120
bta-miR-219-5p	-19.8	609
bta-miR-219-5p	-17.5	350
bta-miR-219-5p	-16.7	405
bta-miR-219-5p	-15.6	240
bta-miR-219-5p	-15.5	652
bta-miR-219-5p	-15.4	486
bta-miR-219-5p	-14.9	434
bta-miR-219-5p	-14.0	66
bta-miR-219-5p	-13.9	696
bta-miR-219-5p	-13.8	101
bta-miR-219-5p	-13.8	172
bta-miR-219-5p	-13.6	25
bta-miR-219-5p	-13.4	81
bta-miR-219-5p	-12.5	218
bta-miR-219-5p	-11.8	736
bta-miR-219-5p	-11.4	574
bta-miR-219-5p	-11.2	506
bta-miR-219-5p	-11.2	1
bta-miR-219-5p	-11.1	422
bta-miR-219-5p	-11.1	545
bta-miR-219-5p	-10.8	377
bta-miR-219-5p	-10.5	338
bta-miR-219-5p	-10.5	473
bta-miR-219-5p	-10.5	628
bta-miR-219-3p	-25.8	41
bta-miR-219-3p	-24.0	94
bta-miR-219-3p	-19.1	164
bta-miR-219-3p	-18.5	232
bta-miR-219-3p	-18.2	113
bta-miR-219-3p	-17.9	554
bta-miR-219-3p	-17.9	16
bta-miR-219-3p	-17.8	454
bta-miR-219-3p	-17.4	418
bta-miR-219-3p	-17.3	479
bta-miR-219-3p	-16.9	664
bta-miR-219-3p	-16.8	128
bta-miR-219-3p	-16.8	589
bta-miR-219-3p	-16.0	359

bta-miR-219-3p	-16.0	395
bta-miR-219-3p	-15.8	256
bta-miR-219-3p	-15.7	616
bta-miR-219-3p	-15.1	507
bta-miR-219-3p	-14.7	313
bta-miR-219-3p	-14.5	1
bta-miR-219-3p	-14.5	278
bta-miR-219-3p	-14.2	183
bta-miR-219-3p	-14.0	374
bta-miR-219-3p	-13.9	575
bta-miR-219-3p	-12.6	737
bta-miR-219-3p	-12.3	84
bta-miR-219-3p	-11.7	710
bta-miR-219-3p	-11.2	644
bta-miR-219-3p	-11.0	472
bta-miR-219-3p	-10.7	342
bta-miR-219-3p	-10.6	155
bta-miR-219-3p	-10.5	525
bta-miR-219-3p	-10.3	67
bta-miR-223	-23.9	615
bta-miR-223	-23.3	69
bta-miR-223	-19.4	174
bta-miR-223	-18.8	478
bta-miR-223	-18.1	448
bta-miR-223	-17.6	16
bta-miR-223	-17.0	344
bta-miR-223	-16.7	113
bta-miR-223	-16.2	267
bta-miR-223	-15.3	545
bta-miR-223	-15.3	417
bta-miR-223	-14.3	730
bta-miR-223	-14.1	576
bta-miR-223	-14.0	598
bta-miR-223	-13.3	372
bta-miR-223	-12.2	660
bta-miR-223	-11.8	505
bta-miR-223	-11.0	221
bta-miR-223	-10.5	41
bta-miR-223	-10.5	364
bta-miR-223	-10.3	199
bta-miR-223	-10.3	568
bta-miR-223	-10.1	1
bta-miR-224	-26.2	617
bta-miR-224	-21.4	480
bta-miR-224	-21.0	507
bta-miR-224	-20.0	113
bta-miR-224	-19.5	174
bta-miR-224	-19.0	228
bta-miR-224	-18.9	404
bta-miR-224	-18.8	726
bta-miR-224	-18.4	11
bta-miR-224	-18.1	70
bta-miR-224	-17.5	576
bta-miR-224	-17.4	334
bta-miR-224	-16.8	454
bta-miR-224	-16.2	422
bta-miR-224	-15.4	265
bta-miR-224	-15.3	599
bta-miR-224	-14.6	49

bta-miR-224	-14.2	537
bta-miR-224	-13.7	34
bta-miR-224	-13.7	370
bta-miR-224	-13.6	558
bta-miR-224	-12.4	662
bta-miR-224	-12.1	197
bta-miR-224	-11.4	147
bta-miR-224	-10.9	136
bta-miR-224	-10.8	709
bta-miR-224	-10.5	313
bta-miR-224	-10.0	445
bta-miR-28	-26.5	262
bta-miR-28	-22.9	14
bta-miR-28	-22.6	495
bta-miR-28	-22.5	607
bta-miR-28	-22.0	410
bta-miR-28	-20.2	67
bta-miR-28	-18.7	229
bta-miR-28	-18.4	346
bta-miR-28	-18.3	737
bta-miR-28	-18.0	449
bta-miR-28	-17.6	193
bta-miR-28	-17.5	107
bta-miR-28	-16.8	370
bta-miR-28	-16.1	567
bta-miR-28	-15.8	469
bta-miR-28	-15.8	653
bta-miR-28	-15.6	629
bta-miR-28	-15.5	516
bta-miR-28	-15.0	150
bta-miR-28	-14.2	542
bta-miR-28	-14.1	703
bta-miR-28	-13.1	302
bta-miR-28	-12.4	35
bta-miR-28	-12.3	586
bta-miR-28	-11.4	173
bta-miR-28	-11.3	393
bta-miR-28	-11.3	433
bta-miR-28	-11.2	1
bta-miR-28	-11.0	554
bta-miR-28	-10.6	93
bta-miR-28	-10.1	212
bta-miR-296	-28.7	451
bta-miR-296	-28.6	557
bta-miR-296	-28.3	153
bta-miR-296	-28.2	240
bta-miR-296	-26.7	102
bta-miR-296	-24.3	414
bta-miR-296	-24.1	313
bta-miR-296	-23.2	348
bta-miR-296	-22.6	394
bta-miR-296	-22.4	479
bta-miR-296	-22.1	574
bta-miR-296	-21.5	177
bta-miR-296	-21.5	69
bta-miR-296	-21.2	28
bta-miR-296	-20.7	129
bta-miR-296	-19.1	280
bta-miR-296	-18.7	620

bta-miR-296	-16.6	663
bta-miR-296	-16.1	3
bta-miR-296	-16.1	693
bta-miR-296	-14.8	371
bta-miR-296	-13.9	220
bta-miR-296	-13.5	517
bta-miR-296	-13.1	58
bta-miR-296	-12.5	735
bta-miR-296	-12.2	91
bta-miR-296	-11.7	710
bta-miR-296	-11.0	599
bta-miR-296	-10.8	610
bta-miR-296	-10.8	642
bta-miR-296	-10.8	271
bta-miR-296	-10.7	342
bta-miR-296	-10.4	652
bta-miR-296	-10.3	682
bta-miR-299	-21.7	81
bta-miR-299	-20.6	412
bta-miR-299	-20.1	614
bta-miR-299	-20.0	266
bta-miR-299	-19.1	7
bta-miR-299	-18.7	450
bta-miR-299	-18.2	497
bta-miR-299	-17.6	658
bta-miR-299	-16.8	543
bta-miR-299	-16.5	113
bta-miR-299	-16.0	62
bta-miR-299	-15.6	478
bta-miR-299	-15.5	583
bta-miR-299	-14.7	176
bta-miR-299	-14.2	598
bta-miR-299	-13.9	229
bta-miR-299	-13.8	40
bta-miR-299	-13.2	738
bta-miR-299	-13.2	359
bta-miR-299	-10.4	339
bta-miR-299	-10.0	570
bta-miR-29d	-19.9	72
bta-miR-29d	-19.2	1
bta-miR-29d	-16.3	435
bta-miR-29d	-16.1	27
bta-miR-29d	-15.8	463
bta-miR-29d	-15.2	93
bta-miR-29d	-15.1	709
bta-miR-29d	-15.0	404
bta-miR-29d	-14.6	617
bta-miR-29d	-14.5	374
bta-miR-29d	-14.1	346
bta-miR-29d	-14.0	576
bta-miR-29d	-13.9	263
bta-miR-29d	-13.1	496
bta-miR-29d	-13.1	223
bta-miR-29d	-11.9	146
bta-miR-29d	-11.8	529
bta-miR-29d	-11.1	173
bta-miR-29d	-11.0	656
bta-miR-29d	-10.6	672
bta-miR-29d	-10.6	311

bta-miR-29d	-10.5	277
bta-miR-29d	-10.3	589
bta-miR-301a	-22.8	518
bta-miR-301a	-21.8	408
bta-miR-301a	-21.3	359
bta-miR-301a	-20.2	560
bta-miR-301a	-17.3	624
bta-miR-301a	-17.2	664
bta-miR-301a	-17.1	3
bta-miR-301a	-16.8	473
bta-miR-301a	-15.9	73
bta-miR-301a	-15.6	116
bta-miR-301a	-15.4	167
bta-miR-301a	-14.9	539
bta-miR-301a	-14.5	266
bta-miR-301a	-14.4	589
bta-miR-301a	-13.8	730
bta-miR-301a	-13.2	51
bta-miR-301a	-13.0	293
bta-miR-301a	-11.7	188
bta-miR-301a	-11.5	693
bta-miR-301a	-11.2	344
bta-miR-301a	-10.7	149
bta-miR-301a	-10.5	452
bta-miR-301b	-21.9	518
bta-miR-301b	-21.8	560
bta-miR-301b	-20.6	359
bta-miR-301b	-17.8	624
bta-miR-301b	-17.0	539
bta-miR-301b	-17.0	664
bta-miR-301b	-16.8	473
bta-miR-301b	-16.6	266
bta-miR-301b	-16.1	408
bta-miR-301b	-15.9	167
bta-miR-301b	-15.9	73
bta-miR-301b	-15.5	20
bta-miR-301b	-15.2	290
bta-miR-301b	-14.1	1
bta-miR-301b	-14.0	116
bta-miR-301b	-13.3	589
bta-miR-301b	-12.8	244
bta-miR-301b	-12.3	738
bta-miR-301b	-11.7	188
bta-miR-301b	-11.3	697
bta-miR-301b	-11.2	344
bta-miR-301b	-10.3	144
bta-miR-301b	-10.3	429
bta-miR-302a	-22.0	55
bta-miR-302a	-21.2	594
bta-miR-302a	-20.8	401
bta-miR-302a	-19.5	5
bta-miR-302a	-18.6	191
bta-miR-302a	-18.1	263
bta-miR-302a	-17.7	457
bta-miR-302a	-17.4	489
bta-miR-302a	-17.1	361
bta-miR-302a	-15.6	225
bta-miR-302a	-15.0	156
bta-miR-302a	-14.9	81

bta-miR-302a	-14.8	100
bta-miR-302a	-14.8	736
bta-miR-302a	-14.3	552
bta-miR-302a	-14.2	649
bta-miR-302a	-14.1	332
bta-miR-302a	-14.0	437
bta-miR-302a	-13.3	696
bta-miR-302a	-11.5	299
bta-miR-302a	-11.3	629
bta-miR-302a	-11.3	422
bta-miR-302d	-22.1	613
bta-miR-302d	-19.6	403
bta-miR-302d	-19.6	586
bta-miR-302d	-19.5	61
bta-miR-302d	-18.1	13
bta-miR-302d	-17.9	194
bta-miR-302d	-17.8	470
bta-miR-302d	-16.7	350
bta-miR-302d	-16.7	437
bta-miR-302d	-16.5	225
bta-miR-302d	-16.0	366
bta-miR-302d	-15.0	497
bta-miR-302d	-14.9	81
bta-miR-302d	-14.8	736
bta-miR-302d	-14.7	103
bta-miR-302d	-14.5	156
bta-miR-302d	-14.3	267
bta-miR-302d	-13.5	649
bta-miR-302d	-12.5	698
bta-miR-302d	-11.3	422
bta-miR-302d	-10.5	630
bta-miR-302d	-10.3	36
bta-miR-302d	-10.1	556
bta-miR-302b	-22.6	594
bta-miR-302b	-20.3	58
bta-miR-302b	-19.6	403
bta-miR-302b	-18.8	13
bta-miR-302b	-18.2	360
bta-miR-302b	-17.9	194
bta-miR-302b	-17.9	457
bta-miR-302b	-16.5	225
bta-miR-302b	-15.2	156
bta-miR-302b	-15.0	497
bta-miR-302b	-14.9	81
bta-miR-302b	-14.8	736
bta-miR-302b	-14.7	103
bta-miR-302b	-14.7	265
bta-miR-302b	-14.3	697
bta-miR-302b	-14.2	649
bta-miR-302b	-14.0	437
bta-miR-302b	-13.8	553
bta-miR-302b	-13.2	337
bta-miR-302b	-11.3	422
bta-miR-302b	-10.5	630
bta-miR-302b	-10.3	36
bta-miR-302c	-24.1	594
bta-miR-302c	-22.1	58
bta-miR-302c	-22.1	401
bta-miR-302c	-20.9	13

bta-miR-302c	-20.1	263
bta-miR-302c	-19.4	474
bta-miR-302c	-19.2	495
bta-miR-302c	-19.0	431
bta-miR-302c	-19.0	362
bta-miR-302c	-18.9	185
bta-miR-302c	-18.8	222
bta-miR-302c	-18.5	331
bta-miR-302c	-17.2	564
bta-miR-302c	-15.4	655
bta-miR-302c	-15.2	86
bta-miR-302c	-14.1	136
bta-miR-302c	-13.9	730
bta-miR-302c	-13.0	535
bta-miR-302c	-12.4	164
bta-miR-302c	-12.1	698
bta-miR-302c	-11.9	34
bta-miR-302c	-10.6	299
bta-miR-302c	-10.5	630
bta-miR-30f	-25.4	610
bta-miR-30f	-25.3	435
bta-miR-30f	-23.4	65
bta-miR-30f	-22.7	12
bta-miR-30f	-18.9	403
bta-miR-30f	-17.3	520
bta-miR-30f	-17.0	198
bta-miR-30f	-17.0	346
bta-miR-30f	-16.8	450
bta-miR-30f	-16.0	653
bta-miR-30f	-15.8	586
bta-miR-30f	-15.3	234
bta-miR-30f	-14.7	539
bta-miR-30f	-14.1	171
bta-miR-30f	-13.0	496
bta-miR-30f	-12.9	184
bta-miR-30f	-12.3	221
bta-miR-30f	-11.9	559
bta-miR-30f	-11.8	280
bta-miR-30f	-11.8	92
bta-miR-32	-21.3	359
bta-miR-32	-18.9	562
bta-miR-32	-18.5	473
bta-miR-32	-18.3	7
bta-miR-32	-18.0	644
bta-miR-32	-16.6	81
bta-miR-32	-16.0	256
bta-miR-32	-15.8	525
bta-miR-32	-15.1	624
bta-miR-32	-14.4	35
bta-miR-32	-13.6	499
bta-miR-32	-13.4	412
bta-miR-32	-13.4	668
bta-miR-32	-12.7	697
bta-miR-32	-12.4	177
bta-miR-32	-11.9	283
bta-miR-32	-11.7	548
bta-miR-32	-11.5	729
bta-miR-32	-11.3	454
bta-miR-32	-11.2	388

bta-miR-32	-11.1	225
bta-miR-32	-11.0	606
bta-miR-32	-10.9	428
bta-miR-32	-10.3	107
bta-miR-323	-23.1	575
bta-miR-323	-23.1	357
bta-miR-323	-22.1	8
bta-miR-323	-19.1	630
bta-miR-323	-18.5	68
bta-miR-323	-17.7	448
bta-miR-323	-17.1	506
bta-miR-323	-16.6	411
bta-miR-323	-15.8	532
bta-miR-323	-15.3	239
bta-miR-323	-15.1	598
bta-miR-323	-14.8	738
bta-miR-323	-14.3	479
bta-miR-323	-13.8	174
bta-miR-323	-13.8	218
bta-miR-323	-13.2	663
bta-miR-323	-13.1	28
bta-miR-323	-12.6	563
bta-miR-323	-12.2	341
bta-miR-323	-12.0	101
bta-miR-323	-11.6	280
bta-miR-323	-11.4	434
bta-miR-323	-11.0	497
bta-miR-323	-10.3	266
bta-miR-323	-10.0	1
bta-miR-324	-30.5	408
bta-miR-324	-30.0	468
bta-miR-324	-29.3	593
bta-miR-324	-29.2	353
bta-miR-324	-26.1	160
bta-miR-324	-24.4	99
bta-miR-324	-23.0	253
bta-miR-324	-22.6	64
bta-miR-324	-22.3	22
bta-miR-324	-21.4	624
bta-miR-324	-21.3	492
bta-miR-324	-19.3	563
bta-miR-324	-17.7	383
bta-miR-324	-17.5	3
bta-miR-324	-16.1	649
bta-miR-324	-15.6	297
bta-miR-324	-15.2	206
bta-miR-324	-15.0	234
bta-miR-324	-14.7	126
bta-miR-324	-14.3	319
bta-miR-324	-13.4	326
bta-miR-324	-12.9	693
bta-miR-324	-12.5	728
bta-miR-324	-12.1	137
bta-miR-324	-12.1	529
bta-miR-324	-11.1	272
bta-miR-324	-10.3	45
bta-miR-324	-10.3	189
bta-miR-324	-10.2	148
bta-miR-326	-32.3	614

bta-miR-326	-29.1	220
bta-miR-326	-27.1	413
bta-miR-326	-26.8	448
bta-miR-326	-25.1	69
bta-miR-326	-20.6	347
bta-miR-326	-20.1	153
bta-miR-326	-19.5	498
bta-miR-326	-18.9	6
bta-miR-326	-17.4	265
bta-miR-326	-17.3	582
bta-miR-326	-16.9	114
bta-miR-326	-15.0	177
bta-miR-326	-13.4	658
bta-miR-326	-12.6	40
bta-miR-326	-11.8	308
bta-miR-326	-11.8	244
bta-miR-326	-11.6	30
bta-miR-326	-11.4	740
bta-miR-326	-11.0	109
bta-miR-326	-10.6	576
bta-miR-326	-10.3	330
bta-miR-326	-10.1	142
bta-miR-326	-10.1	709
bta-miR-326	-10.0	135
bta-miR-326	-10.0	324
bta-miR-326	-10.0	33
bta-miR-328	-33.5	610
bta-miR-328	-30.4	450
bta-miR-328	-27.9	413
bta-miR-328	-24.8	69
bta-miR-328	-22.9	230
bta-miR-328	-20.9	268
bta-miR-328	-20.7	114
bta-miR-328	-20.0	199
bta-miR-328	-19.7	12
bta-miR-328	-17.9	498
bta-miR-328	-17.4	166
bta-miR-328	-16.3	576
bta-miR-328	-14.9	630
bta-miR-328	-14.5	343
bta-miR-328	-14.5	654
bta-miR-328	-14.1	218
bta-miR-328	-13.6	440
bta-miR-328	-13.5	370
bta-miR-328	-12.5	41
bta-miR-328	-12.2	728
bta-miR-328	-11.7	155
bta-miR-328	-11.6	92
bta-miR-328	-11.4	555
bta-miR-328	-10.0	405
bta-miR-329a	-21.8	612
bta-miR-329a	-20.7	12
bta-miR-329a	-17.4	645
bta-miR-329a	-17.1	365
bta-miR-329a	-16.8	445
bta-miR-329a	-16.7	506
bta-miR-329a	-16.0	268
bta-miR-329a	-15.9	405
bta-miR-329a	-15.9	574

bta-miR-329a	-15.5	68
bta-miR-329a	-14.1	26
bta-miR-329a	-13.7	663
bta-miR-329a	-13.2	544
bta-miR-329a	-13.0	125
bta-miR-329a	-12.8	226
bta-miR-329a	-12.8	174
bta-miR-329a	-12.7	153
bta-miR-329a	-12.6	479
bta-miR-329a	-12.1	114
bta-miR-329a	-11.4	423
bta-miR-329a	-11.3	737
bta-miR-329a	-10.6	604
bta-miR-329a	-10.3	340
bta-miR-329a	-10.2	50
bta-miR-329a	-10.0	243
bta-miR-329a	-10.0	557
bta-miR-329b	-25.6	452
bta-miR-329b	-24.9	220
bta-miR-329b	-24.1	265
bta-miR-329b	-23.9	494
bta-miR-329b	-22.5	616
bta-miR-329b	-21.7	197
bta-miR-329b	-21.7	351
bta-miR-329b	-21.4	100
bta-miR-329b	-19.2	410
bta-miR-329b	-18.8	46
bta-miR-329b	-18.6	159
bta-miR-329b	-18.5	597
bta-miR-329b	-16.0	330
bta-miR-329b	-15.9	128
bta-miR-329b	-15.8	303
bta-miR-329b	-15.5	514
bta-miR-329b	-15.4	558
bta-miR-329b	-15.0	701
bta-miR-329b	-14.4	11
bta-miR-329b	-14.0	479
bta-miR-329b	-13.6	79
bta-miR-329b	-13.5	429
bta-miR-329b	-13.0	731
bta-miR-329b	-12.6	383
bta-miR-329b	-12.5	652
bta-miR-329b	-12.4	247
bta-miR-329b	-10.9	184
bta-miR-329b	-10.7	439
bta-miR-329b	-10.7	320
bta-miR-329b	-10.5	33
bta-miR-329b	-10.3	142
bta-miR-329b	-10.2	401
bta-miR-330	-31.5	5
bta-miR-330	-31.3	475
bta-miR-330	-26.1	396
bta-miR-330	-25.7	567
bta-miR-330	-24.2	650
bta-miR-330	-23.5	59
bta-miR-330	-22.6	502
bta-miR-330	-21.6	364
bta-miR-330	-20.6	537
bta-miR-330	-20.0	606

bta-miR-330	-17.6	262
bta-miR-330	-16.3	90
bta-miR-330	-14.4	146
bta-miR-330	-14.1	683
bta-miR-330	-13.8	214
bta-miR-330	-13.7	432
bta-miR-330	-13.4	730
bta-miR-330	-13.0	30
bta-miR-330	-12.9	345
bta-miR-330	-12.2	190
bta-miR-330	-12.0	231
bta-miR-330	-11.1	630
bta-miR-330	-11.0	386
bta-miR-330	-10.9	289
bta-miR-330	-10.6	123
bta-miR-330	-10.6	560
bta-miR-335	-19.3	606
bta-miR-335	-17.0	488
bta-miR-335	-15.6	540
bta-miR-335	-14.6	522
bta-miR-335	-14.6	654
bta-miR-335	-14.0	683
bta-miR-335	-13.5	288
bta-miR-335	-13.3	578
bta-miR-335	-12.7	250
bta-miR-335	-12.2	73
bta-miR-335	-12.1	383
bta-miR-335	-12.1	743
bta-miR-335	-11.6	18
bta-miR-335	-10.9	641
bta-miR-335	-10.8	343
bta-miR-335	-10.2	2
bta-miR-335	-10.1	428
bta-miR-338	-22.9	407
bta-miR-338	-21.9	573
bta-miR-338	-20.2	85
bta-miR-338	-19.8	1
bta-miR-338	-19.0	352
bta-miR-338	-18.1	732
bta-miR-338	-18.0	45
bta-miR-338	-17.2	170
bta-miR-338	-17.1	607
bta-miR-338	-16.2	198
bta-miR-338	-16.0	253
bta-miR-338	-15.9	646
bta-miR-338	-15.9	487
bta-miR-338	-13.7	432
bta-miR-338	-13.7	541
bta-miR-338	-12.9	560
bta-miR-338	-12.9	448
bta-miR-338	-12.8	469
bta-miR-338	-12.5	140
bta-miR-338	-11.7	688
bta-miR-338	-11.6	373
bta-miR-338	-11.5	298
bta-miR-338	-11.2	23
bta-miR-338	-11.1	516
bta-miR-338	-10.0	116
bta-miR-339	-30.2	622

bta-miR-339	-28.4	583
bta-miR-339	-28.3	472
bta-miR-339	-27.1	417
bta-miR-339	-26.8	204
bta-miR-339	-24.0	356
bta-miR-339	-23.2	26
bta-miR-339	-21.4	242
bta-miR-339	-19.5	84
bta-miR-339	-18.8	108
bta-miR-339	-18.1	166
bta-miR-339	-17.1	1
bta-miR-339	-17.1	394
bta-miR-339	-17.0	281
bta-miR-339	-16.4	559
bta-miR-339	-15.1	455
bta-miR-339	-14.7	187
bta-miR-339	-14.0	502
bta-miR-339	-12.9	337
bta-miR-339	-12.8	614
bta-miR-339	-12.2	377
bta-miR-339	-12.0	711
bta-miR-339	-11.9	465
bta-miR-339	-11.5	130
bta-miR-339	-11.2	659
bta-miR-339	-11.1	139
bta-miR-339	-11.1	72
bta-miR-339	-10.9	313
bta-miR-339	-10.9	518
bta-miR-33a	-20.6	358
bta-miR-33a	-19.7	483
bta-miR-33a	-18.2	17
bta-miR-33a	-17.0	66
bta-miR-33a	-16.8	654
bta-miR-33a	-16.6	408
bta-miR-33a	-15.8	516
bta-miR-33a	-15.7	251
bta-miR-33a	-15.5	585
bta-miR-33a	-15.3	109
bta-miR-33a	-14.8	538
bta-miR-33a	-14.7	172
bta-miR-33a	-13.7	624
bta-miR-33a	-13.6	686
bta-miR-33a	-13.4	728
bta-miR-33a	-12.6	41
bta-miR-33a	-12.5	271
bta-miR-33a	-12.4	450
bta-miR-33a	-11.8	560
bta-miR-33a	-10.0	3
bta-miR-33b	-19.6	358
bta-miR-33b	-19.1	254
bta-miR-33b	-18.9	94
bta-miR-33b	-18.4	7
bta-miR-33b	-18.3	485
bta-miR-33b	-18.0	62
bta-miR-33b	-15.9	516
bta-miR-33b	-15.9	654
bta-miR-33b	-15.4	41
bta-miR-33b	-14.5	167
bta-miR-33b	-14.4	606

bta-miR-33b	-14.1	199
bta-miR-33b	-13.9	410
bta-miR-33b	-13.8	554
bta-miR-33b	-12.9	686
bta-miR-33b	-12.5	728
bta-miR-33b	-12.3	118
bta-miR-33b	-12.2	80
bta-miR-33b	-11.2	229
bta-miR-33b	-10.8	566
bta-miR-33b	-10.7	633
bta-miR-33b	-10.5	451
bta-miR-33b	-10.4	278
bta-miR-33b	-10.2	589
bta-miR-33b	-10.1	335
bta-miR-340	-22.7	605
bta-miR-340	-22.2	408
bta-miR-340	-21.7	72
bta-miR-340	-19.0	464
bta-miR-340	-18.9	623
bta-miR-340	-18.3	429
bta-miR-340	-18.2	12
bta-miR-340	-17.2	216
bta-miR-340	-17.1	576
bta-miR-340	-16.5	155
bta-miR-340	-15.7	252
bta-miR-340	-15.6	357
bta-miR-340	-15.0	548
bta-miR-340	-13.8	115
bta-miR-340	-13.1	733
bta-miR-340	-13.1	185
bta-miR-340	-13.0	645
bta-miR-340	-13.0	350
bta-miR-340	-12.5	450
bta-miR-340	-12.1	685
bta-miR-340	-10.4	504
bta-miR-340	-10.0	274
bta-miR-346	-33.7	614
bta-miR-346	-26.6	411
bta-miR-346	-26.1	69
bta-miR-346	-25.7	479
bta-miR-346	-24.6	16
bta-miR-346	-22.1	575
bta-miR-346	-21.3	447
bta-miR-346	-20.7	239
bta-miR-346	-20.3	109
bta-miR-346	-18.7	349
bta-miR-346	-17.6	174
bta-miR-346	-16.4	654
bta-miR-346	-14.0	218
bta-miR-346	-13.4	548
bta-miR-346	-13.1	506
bta-miR-346	-12.1	377
bta-miR-346	-12.1	737
bta-miR-346	-11.7	278
bta-miR-346	-11.6	8
bta-miR-346	-11.2	154
bta-miR-346	-10.7	38
bta-miR-362-5p	-24.2	567
bta-miR-362-5p	-23.4	473

bta-miR-362-5p	-22.8	156
bta-miR-362-5p	-21.9	189
bta-miR-362-5p	-21.6	51
bta-miR-362-5p	-20.9	361
bta-miR-362-5p	-19.9	109
bta-miR-362-5p	-19.8	382
bta-miR-362-5p	-19.3	416
bta-miR-362-5p	-19.3	9
bta-miR-362-5p	-19.1	457
bta-miR-362-5p	-19.1	603
bta-miR-362-5p	-18.5	73
bta-miR-362-5p	-17.9	492
bta-miR-362-5p	-17.1	262
bta-miR-362-5p	-16.6	305
bta-miR-362-5p	-16.6	649
bta-miR-362-5p	-14.6	698
bta-miR-362-5p	-14.1	510
bta-miR-362-5p	-13.6	145
bta-miR-362-5p	-13.5	232
bta-miR-362-5p	-12.8	397
bta-miR-362-5p	-12.4	341
bta-miR-362-5p	-12.1	131
bta-miR-362-5p	-12.1	324
bta-miR-362-5p	-11.8	737
bta-miR-362-5p	-10.7	533
bta-miR-362-5p	-10.3	98
bta-miR-362-3p	-19.7	4
bta-miR-362-3p	-15.8	357
bta-miR-362-3p	-15.6	626
bta-miR-362-3p	-15.1	499
bta-miR-362-3p	-14.9	248
bta-miR-362-3p	-14.6	611
bta-miR-362-3p	-14.5	537
bta-miR-362-3p	-14.5	401
bta-miR-362-3p	-13.6	28
bta-miR-362-3p	-13.3	596
bta-miR-362-3p	-13.1	208
bta-miR-362-3p	-12.9	715
bta-miR-362-3p	-12.6	70
bta-miR-362-3p	-12.5	564
bta-miR-362-3p	-12.4	430
bta-miR-362-3p	-12.0	233
bta-miR-362-3p	-11.5	97
bta-miR-362-3p	-11.2	659
bta-miR-362-3p	-10.9	474
bta-miR-362-3p	-10.4	188
bta-miR-362-3p	-10.3	309
bta-miR-367	-20.9	5
bta-miR-367	-19.9	515
bta-miR-367	-17.9	408
bta-miR-367	-17.5	55
bta-miR-367	-17.4	341
bta-miR-367	-17.0	578
bta-miR-367	-16.9	367
bta-miR-367	-16.8	636
bta-miR-367	-16.7	147
bta-miR-367	-16.7	473
bta-miR-367	-16.6	535
bta-miR-367	-16.3	244

bta-miR-367	-15.2	667
bta-miR-367	-14.3	30
bta-miR-367	-13.5	190
bta-miR-367	-13.3	739
bta-miR-367	-13.0	457
bta-miR-367	-12.9	109
bta-miR-367	-12.5	693
bta-miR-367	-12.3	296
bta-miR-367	-12.3	554
bta-miR-367	-12.1	429
bta-miR-367	-11.7	86
bta-miR-367	-11.1	615
bta-miR-367	-11.1	210
bta-miR-367	-10.9	231
bta-miR-370	-31.9	457
bta-miR-370	-29.4	571
bta-miR-370	-29.0	149
bta-miR-370	-28.9	13
bta-miR-370	-26.7	415
bta-miR-370	-25.8	51
bta-miR-370	-25.8	237
bta-miR-370	-24.7	83
bta-miR-370	-24.7	308
bta-miR-370	-23.5	126
bta-miR-370	-21.2	504
bta-miR-370	-19.9	616
bta-miR-370	-18.9	182
bta-miR-370	-18.4	660
bta-miR-370	-17.8	367
bta-miR-370	-17.2	392
bta-miR-370	-16.1	209
bta-miR-370	-15.2	277
bta-miR-370	-14.3	484
bta-miR-370	-14.2	328
bta-miR-370	-14.0	543
bta-miR-370	-13.4	341
bta-miR-370	-13.3	739
bta-miR-370	-13.1	636
bta-miR-370	-11.1	71
bta-miR-370	-10.7	702
bta-miR-370	-10.6	120
bta-miR-370	-10.6	607
bta-miR-370	-10.2	680
bta-miR-370	-10.0	289
bta-miR-370	-10.0	384
bta-miR-292	-23.1	606
bta-miR-292	-22.3	6
bta-miR-292	-22.0	56
bta-miR-292	-21.1	86
bta-miR-292	-21.0	469
bta-miR-292	-20.6	394
bta-miR-292	-20.0	262
bta-miR-292	-18.4	189
bta-miR-292	-17.8	137
bta-miR-292	-17.8	643
bta-miR-292	-17.8	367
bta-miR-292	-17.3	435
bta-miR-292	-16.7	350
bta-miR-292	-15.8	736

bta-miR-292	-15.7	488
bta-miR-292	-15.2	30
bta-miR-292	-13.8	629
bta-miR-292	-13.7	225
bta-miR-292	-13.6	586
bta-miR-292	-13.5	275
bta-miR-292	-13.1	299
bta-miR-292	-12.2	529
bta-miR-292	-12.0	505
bta-miR-292	-11.9	424
bta-miR-292	-11.6	702
bta-miR-292	-11.5	556
bta-miR-292	-10.2	167
bta-miR-292	-10.0	326
bta-miR-374b	-20.3	56
bta-miR-374b	-17.0	649
bta-miR-374b	-16.6	605
bta-miR-374b	-15.1	12
bta-miR-374b	-14.9	415
bta-miR-374b	-13.7	520
bta-miR-374b	-13.4	537
bta-miR-374b	-13.4	80
bta-miR-374b	-12.8	262
bta-miR-374b	-12.7	737
bta-miR-374b	-12.7	167
bta-miR-374b	-12.4	180
bta-miR-374b	-12.4	489
bta-miR-374b	-12.2	193
bta-miR-374b	-11.9	560
bta-miR-374b	-11.5	23
bta-miR-374b	-11.3	442
bta-miR-374b	-11.2	275
bta-miR-374b	-11.2	623
bta-miR-374b	-10.9	456
bta-miR-374b	-10.7	367
bta-miR-374b	-10.6	472
bta-miR-374b	-10.6	108
bta-miR-374b	-10.4	702
bta-miR-374b	-10.3	425
bta-miR-375	-24.5	23
bta-miR-375	-23.9	161
bta-miR-375	-22.1	476
bta-miR-375	-20.9	354
bta-miR-375	-20.6	412
bta-miR-375	-19.5	428
bta-miR-375	-19.2	560
bta-miR-375	-18.3	578
bta-miR-375	-18.3	81
bta-miR-375	-18.0	223
bta-miR-375	-17.8	263
bta-miR-375	-17.8	619
bta-miR-375	-16.0	299
bta-miR-375	-15.7	9
bta-miR-375	-15.4	121
bta-miR-375	-14.7	184
bta-miR-375	-14.3	55
bta-miR-375	-14.2	707
bta-miR-375	-14.0	242
bta-miR-375	-13.9	455

bta-miR-375	-13.3	108
bta-miR-375	-13.3	497
bta-miR-375	-13.1	543
bta-miR-375	-12.9	740
bta-miR-375	-12.7	660
bta-miR-375	-12.6	332
bta-miR-375	-12.2	606
bta-miR-375	-11.4	147
bta-miR-375	-11.4	377
bta-miR-375	-11.3	204
bta-miR-375	-10.6	401
bta-miR-377	-19.5	614
bta-miR-377	-19.0	66
bta-miR-377	-18.1	353
bta-miR-377	-18.1	654
bta-miR-377	-16.9	571
bta-miR-377	-16.3	517
bta-miR-377	-16.3	537
bta-miR-377	-15.7	469
bta-miR-377	-15.7	13
bta-miR-377	-14.8	739
bta-miR-377	-14.2	271
bta-miR-377	-13.9	415
bta-miR-377	-13.8	106
bta-miR-377	-13.8	557
bta-miR-377	-13.7	22
bta-miR-377	-12.6	240
bta-miR-377	-12.5	450
bta-miR-377	-12.4	307
bta-miR-377	-12.4	667
bta-miR-377	-11.7	172
bta-miR-377	-11.2	202
bta-miR-377	-11.0	84
bta-miR-377	-11.0	548
bta-miR-377	-10.9	2
bta-miR-377	-10.9	46
bta-miR-377	-10.5	696
bta-miR-377	-10.5	126
bta-miR-377	-10.5	408
bta-miR-377	-10.1	149
bta-miR-378	-25.9	563
bta-miR-378	-24.7	212
bta-miR-378	-24.5	473
bta-miR-378	-24.4	73
bta-miR-378	-23.6	109
bta-miR-378	-21.9	586
bta-miR-378	-21.8	425
bta-miR-378	-21.5	186
bta-miR-378	-21.3	381
bta-miR-378	-19.1	361
bta-miR-378	-19.0	502
bta-miR-378	-18.0	308
bta-miR-378	-17.9	51
bta-miR-378	-17.7	4
bta-miR-378	-17.5	143
bta-miR-378	-17.1	639
bta-miR-378	-16.5	130
bta-miR-378	-15.7	681
bta-miR-378	-15.4	623

bta-miR-378	-15.0	244
bta-miR-378	-14.9	716
bta-miR-378	-14.2	520
bta-miR-378	-13.0	284
bta-miR-378	-12.3	331
bta-miR-378	-11.5	415
bta-miR-378	-10.2	270
bta-miR-378	-10.0	466
bta-miR-379	-22.3	91
bta-miR-379	-21.2	458
bta-miR-379	-20.1	561
bta-miR-379	-19.6	388
bta-miR-379	-18.0	650
bta-miR-379	-16.4	502
bta-miR-379	-16.0	283
bta-miR-379	-15.9	14
bta-miR-379	-14.9	230
bta-miR-379	-14.9	416
bta-miR-379	-14.7	309
bta-miR-379	-14.6	363
bta-miR-379	-14.1	248
bta-miR-379	-13.8	34
bta-miR-379	-13.4	57
bta-miR-379	-12.9	531
bta-miR-379	-12.4	146
bta-miR-379	-12.0	4
bta-miR-379	-11.9	691
bta-miR-379	-11.1	169
bta-miR-379	-11.1	707
bta-miR-379	-11.1	583
bta-miR-379	-10.8	209
bta-miR-379	-10.5	619
bta-miR-379	-10.3	77
bta-miR-379	-10.3	122
bta-miR-379	-10.3	741
bta-miR-379	-10.1	343
bta-miR-381	-29.4	238
bta-miR-381	-27.9	66
bta-miR-381	-25.8	613
bta-miR-381	-24.4	199
bta-miR-381	-23.6	410
bta-miR-381	-23.0	348
bta-miR-381	-20.6	494
bta-miR-381	-20.5	106
bta-miR-381	-19.3	278
bta-miR-381	-18.5	22
bta-miR-381	-18.3	451
bta-miR-381	-17.1	172
bta-miR-381	-16.5	654
bta-miR-381	-14.8	559
bta-miR-381	-14.8	479
bta-miR-381	-14.5	737
bta-miR-381	-14.1	376
bta-miR-381	-14.0	149
bta-miR-381	-13.6	264
bta-miR-381	-13.5	440
bta-miR-381	-13.4	220
bta-miR-381	-13.1	537
bta-miR-381	-12.4	307

bta-miR-381	-12.3	635
bta-miR-381	-12.2	571
bta-miR-381	-12.1	167
bta-miR-381	-11.9	128
bta-miR-381	-11.5	257
bta-miR-381	-11.5	595
bta-miR-381	-11.4	1
bta-miR-381	-11.0	695
bta-miR-381	-10.9	551
bta-miR-381	-10.3	51
bta-miR-381	-10.0	228
bta-miR-382	-22.4	621
bta-miR-382	-20.6	589
bta-miR-382	-19.9	356
bta-miR-382	-19.5	114
bta-miR-382	-19.2	233
bta-miR-382	-19.0	188
bta-miR-382	-18.3	312
bta-miR-382	-18.0	52
bta-miR-382	-17.8	258
bta-miR-382	-17.4	140
bta-miR-382	-17.0	499
bta-miR-382	-16.9	85
bta-miR-382	-16.5	164
bta-miR-382	-16.2	463
bta-miR-382	-15.9	561
bta-miR-382	-15.4	1
bta-miR-382	-15.1	401
bta-miR-382	-14.4	710
bta-miR-382	-14.3	423
bta-miR-382	-13.9	28
bta-miR-382	-13.3	663
bta-miR-382	-12.4	580
bta-miR-382	-12.2	294
bta-miR-382	-12.0	445
bta-miR-382	-11.6	692
bta-miR-382	-11.5	341
bta-miR-382	-11.4	133
bta-miR-382	-10.6	515
bta-miR-383	-22.4	559
bta-miR-383	-22.1	50
bta-miR-383	-21.9	198
bta-miR-383	-21.7	482
bta-miR-383	-21.0	407
bta-miR-383	-20.1	119
bta-miR-383	-19.8	361
bta-miR-383	-19.3	456
bta-miR-383	-18.5	669
bta-miR-383	-17.8	166
bta-miR-383	-15.8	72
bta-miR-383	-15.7	331
bta-miR-383	-15.4	308
bta-miR-383	-15.3	243
bta-miR-383	-15.0	707
bta-miR-383	-15.0	12
bta-miR-383	-14.8	623
bta-miR-383	-14.5	270
bta-miR-383	-14.5	511
bta-miR-383	-14.4	155

bta-miR-383	-14.4	97
bta-miR-383	-14.3	136
bta-miR-383	-12.2	393
bta-miR-383	-12.0	426
bta-miR-383	-11.2	733
bta-miR-383	-11.0	651
bta-miR-383	-10.7	594
bta-miR-383	-10.0	294
bta-miR-383	-10.0	532
bta-miR-409a	-27.7	606
bta-miR-409a	-25.6	493
bta-miR-409a	-25.4	554
bta-miR-409a	-23.1	408
bta-miR-409a	-23.0	66
bta-miR-409a	-22.2	3
bta-miR-409a	-21.6	428
bta-miR-409a	-20.0	469
bta-miR-409a	-19.2	573
bta-miR-409a	-18.9	654
bta-miR-409a	-18.1	271
bta-miR-409a	-17.8	525
bta-miR-409a	-17.1	379
bta-miR-409a	-17.1	95
bta-miR-409a	-16.1	686
bta-miR-409a	-15.8	172
bta-miR-409a	-15.1	30
bta-miR-409a	-14.9	738
bta-miR-409a	-14.8	352
bta-miR-409a	-14.3	199
bta-miR-409a	-13.1	220
bta-miR-409a	-12.9	307
bta-miR-409a	-10.4	251
bta-miR-409a	-10.2	155
bta-miR-409a	-10.2	519
bta-miR-410	-20.5	10
bta-miR-410	-19.7	423
bta-miR-410	-19.0	84
bta-miR-410	-18.8	560
bta-miR-410	-18.4	575
bta-miR-410	-17.9	516
bta-miR-410	-17.7	602
bta-miR-410	-17.2	376
bta-miR-410	-16.9	481
bta-miR-410	-15.6	51
bta-miR-410	-15.2	454
bta-miR-410	-15.0	241
bta-miR-410	-15.0	631
bta-miR-410	-14.7	539
bta-miR-410	-14.6	408
bta-miR-410	-14.0	154
bta-miR-410	-13.8	106
bta-miR-410	-13.4	122
bta-miR-410	-13.1	473
bta-miR-410	-12.8	199
bta-miR-410	-12.8	649
bta-miR-410	-12.7	278
bta-miR-410	-12.6	342
bta-miR-410	-12.6	71
bta-miR-410	-12.4	358

bta-miR-410	-12.2	30
bta-miR-410	-11.8	669
bta-miR-410	-11.6	269
bta-miR-410	-11.4	165
bta-miR-410	-11.0	746
bta-miR-410	-10.9	304
bta-miR-410	-10.3	623
bta-miR-411	-18.6	555
bta-miR-411	-18.4	454
bta-miR-411	-18.2	412
bta-miR-411	-18.2	518
bta-miR-411	-17.2	618
bta-miR-411	-17.0	8
bta-miR-411	-14.6	71
bta-miR-411	-14.6	654
bta-miR-411	-14.4	379
bta-miR-411	-14.1	25
bta-miR-411	-13.3	729
bta-miR-411	-13.1	359
bta-miR-411	-13.0	585
bta-miR-411	-12.9	109
bta-miR-411	-11.9	172
bta-miR-411	-11.7	685
bta-miR-411	-11.3	473
bta-miR-411	-11.0	280
bta-miR-411	-10.8	544
bta-miR-411	-10.6	497
bta-miR-411	-10.3	305
bta-miR-411	-10.1	154
bta-miR-412	-25.7	605
bta-miR-412	-25.0	407
bta-miR-412	-24.0	12
bta-miR-412	-23.9	488
bta-miR-412	-23.8	64
bta-miR-412	-20.8	353
bta-miR-412	-20.5	559
bta-miR-412	-19.7	106
bta-miR-412	-19.7	473
bta-miR-412	-19.6	170
bta-miR-412	-18.9	623
bta-miR-412	-18.7	586
bta-miR-412	-18.6	264
bta-miR-412	-17.9	435
bta-miR-412	-17.8	37
bta-miR-412	-16.9	653
bta-miR-412	-16.8	224
bta-miR-412	-15.6	456
bta-miR-412	-13.4	199
bta-miR-412	-13.1	86
bta-miR-412	-12.7	737
bta-miR-412	-12.5	155
bta-miR-412	-12.5	698
bta-miR-412	-12.4	119
bta-miR-412	-12.3	517
bta-miR-412	-11.1	341
bta-miR-412	-10.3	282
bta-miR-421	-23.3	244
bta-miR-421	-23.1	30
bta-miR-421	-18.9	359

bta-miR-421	-18.8	71
bta-miR-421	-17.7	518
bta-miR-421	-17.6	428
bta-miR-421	-17.3	565
bta-miR-421	-16.7	167
bta-miR-421	-15.9	461
bta-miR-421	-15.6	622
bta-miR-421	-15.4	408
bta-miR-421	-15.0	51
bta-miR-421	-14.7	109
bta-miR-421	-14.6	3
bta-miR-421	-13.9	330
bta-miR-421	-13.3	283
bta-miR-421	-13.2	659
bta-miR-421	-13.2	206
bta-miR-421	-13.0	96
bta-miR-421	-13.0	235
bta-miR-421	-12.8	483
bta-miR-421	-12.2	307
bta-miR-421	-12.0	119
bta-miR-421	-11.1	551
bta-miR-421	-10.9	18
bta-miR-421	-10.3	126
bta-miR-421	-10.2	142
bta-miR-421	-10.1	135
bta-miR-421	-10.1	324
bta-miR-421	-10.1	697
bta-miR-429	-20.6	488
bta-miR-429	-20.1	115
bta-miR-429	-19.5	597
bta-miR-429	-19.2	413
bta-miR-429	-18.8	230
bta-miR-429	-18.5	359
bta-miR-429	-17.0	662
bta-miR-429	-17.0	19
bta-miR-429	-16.9	507
bta-miR-429	-16.6	155
bta-miR-429	-16.6	85
bta-miR-429	-15.3	270
bta-miR-429	-14.9	463
bta-miR-429	-14.6	617
bta-miR-429	-13.5	560
bta-miR-429	-13.3	177
bta-miR-429	-12.6	9
bta-miR-429	-12.6	204
bta-miR-429	-12.4	64
bta-miR-429	-12.4	741
bta-miR-429	-11.9	341
bta-miR-429	-11.6	428
bta-miR-429	-11.0	261
bta-miR-429	-10.3	654
bta-miR-431	-28.2	402
bta-miR-431	-26.1	60
bta-miR-431	-24.7	5
bta-miR-431	-24.1	581
bta-miR-431	-23.5	474
bta-miR-431	-23.3	352
bta-miR-431	-22.8	111
bta-miR-431	-22.4	616

bta-miR-431	-20.7	249
bta-miR-431	-20.1	542
bta-miR-431	-19.3	185
bta-miR-431	-18.4	654
bta-miR-431	-18.1	156
bta-miR-431	-18.0	561
bta-miR-431	-16.9	87
bta-miR-431	-15.9	206
bta-miR-431	-15.4	229
bta-miR-431	-15.4	377
bta-miR-431	-14.8	738
bta-miR-431	-13.7	519
bta-miR-431	-13.4	331
bta-miR-431	-13.1	31
bta-miR-431	-12.4	445
bta-miR-431	-12.3	292
bta-miR-431	-11.3	457
bta-miR-431	-11.1	685
bta-miR-431	-10.7	276
bta-miR-432	-24.4	361
bta-miR-432	-24.2	561
bta-miR-432	-24.1	325
bta-miR-432	-23.5	407
bta-miR-432	-23.2	161
bta-miR-432	-22.5	260
bta-miR-432	-22.5	117
bta-miR-432	-22.4	458
bta-miR-432	-21.2	511
bta-miR-432	-20.0	13
bta-miR-432	-19.3	587
bta-miR-432	-19.2	299
bta-miR-432	-17.9	655
bta-miR-432	-17.7	207
bta-miR-432	-17.7	624
bta-miR-432	-17.3	73
bta-miR-432	-17.2	136
bta-miR-432	-16.8	244
bta-miR-432	-16.3	696
bta-miR-432	-16.0	384
bta-miR-432	-15.2	489
bta-miR-432	-15.1	52
bta-miR-432	-12.5	537
bta-miR-432	-12.1	734
bta-miR-432	-11.3	109
bta-miR-432	-10.9	482
bta-miR-433	-23.4	415
bta-miR-433	-23.3	473
bta-miR-433	-23.0	13
bta-miR-433	-22.8	452
bta-miR-433	-22.8	160
bta-miR-433	-22.7	275
bta-miR-433	-21.5	216
bta-miR-433	-21.1	66
bta-miR-433	-20.3	571
bta-miR-433	-20.1	620
bta-miR-433	-19.9	234
bta-miR-433	-19.3	319
bta-miR-433	-19.3	593
bta-miR-433	-19.1	353

bta-miR-433	-17.8	109
bta-miR-433	-17.7	654
bta-miR-433	-16.4	86
bta-miR-433	-16.2	494
bta-miR-433	-15.9	383
bta-miR-433	-14.6	266
bta-miR-433	-14.3	137
bta-miR-433	-14.1	199
bta-miR-433	-14.0	1
bta-miR-433	-13.9	50
bta-miR-433	-13.9	436
bta-miR-433	-13.3	341
bta-miR-433	-12.8	559
bta-miR-433	-12.8	739
bta-miR-433	-12.6	523
bta-miR-433	-12.5	702
bta-miR-433	-12.0	542
bta-miR-433	-11.8	185
bta-miR-433	-10.9	125
bta-miR-433	-10.9	407
bta-miR-433	-10.9	613
bta-miR-433	-10.6	149
bta-miR-433	-10.3	308
bta-miR-448	-25.6	81
bta-miR-448	-22.9	505
bta-miR-448	-21.8	478
bta-miR-448	-21.5	266
bta-miR-448	-19.7	347
bta-miR-448	-19.1	621
bta-miR-448	-18.7	15
bta-miR-448	-18.1	176
bta-miR-448	-17.9	417
bta-miR-448	-17.0	598
bta-miR-448	-16.4	450
bta-miR-448	-15.2	576
bta-miR-448	-15.2	113
bta-miR-448	-14.5	70
bta-miR-448	-14.3	710
bta-miR-448	-14.3	658
bta-miR-448	-14.1	297
bta-miR-448	-14.0	230
bta-miR-448	-13.0	40
bta-miR-448	-11.9	635
bta-miR-448	-11.7	609
bta-miR-448	-11.1	401
bta-miR-448	-11.0	383
bta-miR-448	-11.0	614
bta-miR-448	-10.6	539
bta-miR-448	-10.4	464
bta-miR-448	-10.3	690
bta-miR-448	-10.1	160
bta-miR-449a	-27.3	99
bta-miR-449a	-26.2	408
bta-miR-449a	-23.8	12
bta-miR-449a	-21.0	563
bta-miR-449a	-20.1	164
bta-miR-449a	-20.0	636
bta-miR-449a	-19.9	72
bta-miR-449a	-19.1	341

bta-miR-449a	-19.0	456
bta-miR-449a	-18.3	515
bta-miR-449a	-18.2	367
bta-miR-449a	-17.6	483
bta-miR-449a	-17.2	34
bta-miR-449a	-17.1	126
bta-miR-449a	-16.7	263
bta-miR-449a	-15.7	595
bta-miR-449a	-15.5	693
bta-miR-449a	-13.7	294
bta-miR-449a	-13.7	237
bta-miR-449a	-13.2	739
bta-miR-449a	-12.4	200
bta-miR-449a	-10.7	610
bta-miR-449a	-10.7	431
bta-miR-449a	-10.4	1
bta-miR-449a	-10.1	392
bta-miR-449b	-27.2	408
bta-miR-449b	-26.4	99
bta-miR-449b	-25.3	8
bta-miR-449b	-21.8	563
bta-miR-449b	-21.0	66
bta-miR-449b	-20.2	164
bta-miR-449b	-20.0	456
bta-miR-449b	-19.1	515
bta-miR-449b	-19.0	645
bta-miR-449b	-18.7	342
bta-miR-449b	-18.4	483
bta-miR-449b	-18.1	126
bta-miR-449b	-17.0	263
bta-miR-449b	-16.5	698
bta-miR-449b	-16.2	34
bta-miR-449b	-15.3	155
bta-miR-449b	-15.0	372
bta-miR-449b	-14.7	294
bta-miR-449b	-14.2	596
bta-miR-449b	-13.0	198
bta-miR-449b	-12.9	237
bta-miR-449b	-12.6	663
bta-miR-449b	-12.5	623
bta-miR-449b	-12.5	729
bta-miR-449b	-10.7	435
bta-miR-449b	-10.6	544
bta-miR-449b	-10.2	392
bta-miR-449c	-27.3	12
bta-miR-449c	-25.8	414
bta-miR-449c	-24.7	169
bta-miR-449c	-23.6	107
bta-miR-449c	-22.7	515
bta-miR-449c	-22.3	342
bta-miR-449c	-22.2	64
bta-miR-449c	-21.7	198
bta-miR-449c	-20.1	462
bta-miR-449c	-19.9	545
bta-miR-449c	-19.6	623
bta-miR-449c	-18.3	34
bta-miR-449c	-17.7	237
bta-miR-449c	-16.9	435
bta-miR-449c	-16.9	653

bta-miR-449c	-16.1	125
bta-miR-449c	-16.1	605
bta-miR-449c	-16.0	572
bta-miR-449c	-15.9	282
bta-miR-449c	-15.2	370
bta-miR-449c	-13.6	263
bta-miR-449c	-13.4	495
bta-miR-449c	-13.3	698
bta-miR-449c	-13.0	737
bta-miR-449c	-12.5	392
bta-miR-449c	-12.2	155
bta-miR-449c	-12.0	1
bta-miR-449c	-11.7	219
bta-miR-449c	-10.9	94
bta-miR-449c	-10.1	228
bta-miR-449c	-10.1	589
bta-miR-451	-19.6	406
bta-miR-451	-18.9	600
bta-miR-451	-17.9	262
bta-miR-451	-17.3	624
bta-miR-451	-16.3	11
bta-miR-451	-15.3	71
bta-miR-451	-15.0	486
bta-miR-451	-13.5	516
bta-miR-451	-12.7	648
bta-miR-451	-12.1	166
bta-miR-451	-12.0	432
bta-miR-451	-11.9	574
bta-miR-451	-11.8	145
bta-miR-451	-11.5	701
bta-miR-451	-11.5	216
bta-miR-451	-11.3	731
bta-miR-451	-11.2	351
bta-miR-451	-11.1	107
bta-miR-451	-10.4	188
bta-miR-451	-10.3	475
bta-miR-452	-23.3	461
bta-miR-452	-21.8	566
bta-miR-452	-20.8	262
bta-miR-452	-20.5	681
bta-miR-452	-20.1	626
bta-miR-452	-18.9	502
bta-miR-452	-17.6	276
bta-miR-452	-17.3	541
bta-miR-452	-17.0	55
bta-miR-452	-17.0	181
bta-miR-452	-16.9	395
bta-miR-452	-16.6	97
bta-miR-452	-16.2	14
bta-miR-452	-15.1	707
bta-miR-452	-14.9	356
bta-miR-452	-14.5	212
bta-miR-452	-14.1	235
bta-miR-452	-13.5	310
bta-miR-452	-13.3	376
bta-miR-452	-13.3	491
bta-miR-452	-13.2	657
bta-miR-452	-12.9	139
bta-miR-452	-12.8	421

bta-miR-452	-12.7	165
bta-miR-452	-12.2	2
bta-miR-452	-11.9	596
bta-miR-452	-11.4	74
bta-miR-452	-10.8	521
bta-miR-452	-10.6	288
bta-miR-452	-10.2	37
bta-miR-452	-10.2	128
bta-miR-453	-24.6	359
bta-miR-453	-24.5	93
bta-miR-453	-24.4	697
bta-miR-453	-24.0	619
bta-miR-453	-23.5	25
bta-miR-453	-23.4	497
bta-miR-453	-22.9	412
bta-miR-453	-21.8	562
bta-miR-453	-21.1	455
bta-miR-453	-20.7	185
bta-miR-453	-20.7	126
bta-miR-453	-20.1	585
bta-miR-453	-18.5	257
bta-miR-453	-18.4	312
bta-miR-453	-16.8	69
bta-miR-453	-16.6	644
bta-miR-453	-16.2	476
bta-miR-453	-15.9	231
bta-miR-453	-15.3	281
bta-miR-453	-14.9	378
bta-miR-453	-13.7	167
bta-miR-453	-13.3	211
bta-miR-453	-12.2	3
bta-miR-453	-12.0	730
bta-miR-453	-11.9	53
bta-miR-453	-11.3	518
bta-miR-453	-11.1	607
bta-miR-454	-24.3	361
bta-miR-454	-21.6	473
bta-miR-454	-20.3	401
bta-miR-454	-19.7	167
bta-miR-454	-19.1	109
bta-miR-454	-18.8	5
bta-miR-454	-18.8	191
bta-miR-454	-17.7	429
bta-miR-454	-17.6	59
bta-miR-454	-17.6	624
bta-miR-454	-17.5	260
bta-miR-454	-16.8	510
bta-miR-454	-16.3	563
bta-miR-454	-14.5	30
bta-miR-454	-14.2	275
bta-miR-454	-14.1	681
bta-miR-454	-13.8	78
bta-miR-454	-13.1	652
bta-miR-454	-12.5	596
bta-miR-454	-12.2	715
bta-miR-454	-11.5	540
bta-miR-454	-11.0	211
bta-miR-454	-10.7	341
bta-miR-454	-10.6	247

bta-miR-454	-10.6	141
bta-miR-454	-10.1	320
bta-miR-454	-10.1	327
bta-miR-483	-29.9	604
bta-miR-483	-25.3	443
bta-miR-483	-21.3	218
bta-miR-483	-20.8	265
bta-miR-483	-20.0	66
bta-miR-483	-17.7	414
bta-miR-483	-17.3	11
bta-miR-483	-16.5	478
bta-miR-483	-15.5	348
bta-miR-483	-13.8	505
bta-miR-483	-13.2	597
bta-miR-483	-13.0	366
bta-miR-483	-12.7	153
bta-miR-483	-12.4	736
bta-miR-483	-11.9	570
bta-miR-483	-10.7	202
bta-miR-483	-10.7	240
bta-miR-485	-28.4	454
bta-miR-485	-26.3	405
bta-miR-485	-22.9	102
bta-miR-485	-22.5	294
bta-miR-485	-21.8	9
bta-miR-485	-21.5	384
bta-miR-485	-20.4	145
bta-miR-485	-19.4	263
bta-miR-485	-19.4	551
bta-miR-485	-19.1	53
bta-miR-485	-18.6	312
bta-miR-485	-18.0	233
bta-miR-485	-17.6	480
bta-miR-485	-17.2	178
bta-miR-485	-17.1	496
bta-miR-485	-17.0	655
bta-miR-485	-16.6	611
bta-miR-485	-15.8	83
bta-miR-485	-14.9	23
bta-miR-485	-14.7	354
bta-miR-485	-14.2	704
bta-miR-485	-14.1	35
bta-miR-485	-13.9	730
bta-miR-485	-13.4	596
bta-miR-485	-11.3	532
bta-miR-485	-11.1	128
bta-miR-485	-10.9	687
bta-miR-485	-10.9	370
bta-miR-485	-10.6	170
bta-miR-485	-10.5	434
bta-miR-485	-10.2	252
bta-miR-486	-28.8	477
bta-miR-486	-26.7	615
bta-miR-486	-25.2	267
bta-miR-486	-22.8	82
bta-miR-486	-22.3	421
bta-miR-486	-21.1	597
bta-miR-486	-20.8	39
bta-miR-486	-20.7	449

bta-miR-486	-19.3	155
bta-miR-486	-18.7	112
bta-miR-486	-16.9	5
bta-miR-486	-16.5	397
bta-miR-486	-15.7	190
bta-miR-486	-14.2	576
bta-miR-486	-13.8	370
bta-miR-486	-12.9	657
bta-miR-486	-12.5	516
bta-miR-486	-12.2	232
bta-miR-486	-12.1	71
bta-miR-486	-12.1	742
bta-miR-486	-11.9	280
bta-miR-486	-11.4	351
bta-miR-486	-10.9	698
bta-miR-486	-10.8	542
bta-miR-486	-10.4	183
bta-miR-488	-20.9	99
bta-miR-488	-19.6	482
bta-miR-488	-19.3	595
bta-miR-488	-18.7	155
bta-miR-488	-18.1	341
bta-miR-488	-18.1	12
bta-miR-488	-18.0	570
bta-miR-488	-17.6	408
bta-miR-488	-17.1	51
bta-miR-488	-16.8	445
bta-miR-488	-16.7	229
bta-miR-488	-16.6	275
bta-miR-488	-15.5	500
bta-miR-488	-15.1	119
bta-miR-488	-15.1	366
bta-miR-488	-14.9	188
bta-miR-488	-14.4	298
bta-miR-488	-14.3	468
bta-miR-488	-13.8	607
bta-miR-488	-13.5	623
bta-miR-488	-13.3	558
bta-miR-488	-13.1	83
bta-miR-488	-12.4	669
bta-miR-488	-12.1	698
bta-miR-488	-11.9	130
bta-miR-488	-11.2	175
bta-miR-488	-11.1	393
bta-miR-488	-11.0	263
bta-miR-488	-10.6	731
bta-miR-488	-10.2	37
bta-miR-488	-10.2	216
bta-miR-488	-10.1	649
bta-miR-490	-27.9	22
bta-miR-490	-26.2	615
bta-miR-490	-24.0	198
bta-miR-490	-22.7	80
bta-miR-490	-22.7	228
bta-miR-490	-22.0	456
bta-miR-490	-21.4	419
bta-miR-490	-20.9	487
bta-miR-490	-20.4	107
bta-miR-490	-19.8	270

bta-miR-490	-19.8	559
bta-miR-490	-19.8	351
bta-miR-490	-18.9	64
bta-miR-490	-18.0	653
bta-miR-490	-17.7	170
bta-miR-490	-17.5	4
bta-miR-490	-16.2	376
bta-miR-490	-15.5	41
bta-miR-490	-15.0	580
bta-miR-490	-14.8	709
bta-miR-490	-14.6	258
bta-miR-490	-14.1	605
bta-miR-490	-12.7	130
bta-miR-490	-12.0	527
bta-miR-490	-11.9	140
bta-miR-490	-11.5	729
bta-miR-490	-11.4	308
bta-miR-490	-11.2	440
bta-miR-490	-10.8	328
bta-miR-490	-10.8	637
bta-miR-491	-23.2	9
bta-miR-491	-22.5	91
bta-miR-491	-22.0	261
bta-miR-491	-21.8	474
bta-miR-491	-21.5	607
bta-miR-491	-21.3	497
bta-miR-491	-20.4	309
bta-miR-491	-20.2	396
bta-miR-491	-19.7	230
bta-miR-491	-19.2	451
bta-miR-491	-18.6	564
bta-miR-491	-17.3	703
bta-miR-491	-17.3	140
bta-miR-491	-16.7	58
bta-miR-491	-16.5	168
bta-miR-491	-16.1	363
bta-miR-491	-16.1	630
bta-miR-491	-15.8	657
bta-miR-491	-14.4	329
bta-miR-491	-13.9	150
bta-miR-491	-13.5	210
bta-miR-491	-13.2	738
bta-miR-491	-11.6	541
bta-miR-491	-11.3	587
bta-miR-491	-11.3	40
bta-miR-491	-11.2	284
bta-miR-491	-10.7	680
bta-miR-491	-10.4	443
bta-miR-493	-28.5	484
bta-miR-493	-23.8	402
bta-miR-493	-22.2	60
bta-miR-493	-21.1	111
bta-miR-493	-21.1	270
bta-miR-493	-20.2	368
bta-miR-493	-19.1	596
bta-miR-493	-18.9	431
bta-miR-493	-18.7	12
bta-miR-493	-16.8	155
bta-miR-493	-16.8	231

bta-miR-493	-16.0	39
bta-miR-493	-16.0	192
bta-miR-493	-16.0	654
bta-miR-493	-15.9	581
bta-miR-493	-14.9	82
bta-miR-493	-14.8	507
bta-miR-493	-14.4	454
bta-miR-493	-13.9	560
bta-miR-493	-13.5	346
bta-miR-493	-12.6	292
bta-miR-493	-12.1	740
bta-miR-493	-11.7	696
bta-miR-493	-11.4	632
bta-miR-493	-10.5	216
bta-miR-493	-10.5	476
bta-miR-493	-10.2	176
bta-miR-494	-22.7	575
bta-miR-494	-18.7	539
bta-miR-494	-18.0	506
bta-miR-494	-17.8	620
bta-miR-494	-17.4	83
bta-miR-494	-16.4	240
bta-miR-494	-15.0	657
bta-miR-494	-14.4	16
bta-miR-494	-14.0	452
bta-miR-494	-13.9	368
bta-miR-494	-13.8	400
bta-miR-494	-13.1	423
bta-miR-494	-12.9	710
bta-miR-494	-12.7	479
bta-miR-494	-12.6	279
bta-miR-494	-12.5	679
bta-miR-494	-11.7	133
bta-miR-494	-11.3	111
bta-miR-494	-11.1	357
bta-miR-494	-11.1	154
bta-miR-494	-10.8	174
bta-miR-494	-10.5	202
bta-miR-494	-10.4	342
bta-miR-494	-10.4	59
bta-miR-494	-10.3	69
bta-miR-494	-10.3	218
bta-miR-494	-10.2	562
bta-miR-494	-10.0	324
bta-miR-494	-10.0	330
bta-miR-495	-20.3	506
bta-miR-495	-18.2	630
bta-miR-495	-18.0	375
bta-miR-495	-17.4	16
bta-miR-495	-17.3	450
bta-miR-495	-17.1	350
bta-miR-495	-16.3	654
bta-miR-495	-16.1	544
bta-miR-495	-14.7	232
bta-miR-495	-13.8	613
bta-miR-495	-13.7	68
bta-miR-495	-13.5	279
bta-miR-495	-13.3	479
bta-miR-495	-13.1	423

bta-miR-495	-13.0	574
bta-miR-495	-12.3	197
bta-miR-495	-12.1	408
bta-miR-495	-12.0	114
bta-miR-495	-12.0	265
bta-miR-495	-11.9	560
bta-miR-495	-11.5	736
bta-miR-495	-11.3	167
bta-miR-495	-11.0	598
bta-miR-495	-10.6	697
bta-miR-495	-10.5	43
bta-miR-495	-10.5	313
bta-miR-495	-10.3	176
bta-miR-495	-10.3	341
bta-miR-495	-10.1	218
bta-miR-496	-18.3	615
bta-miR-496	-17.2	12
bta-miR-496	-16.9	240
bta-miR-496	-15.8	663
bta-miR-496	-15.7	348
bta-miR-496	-15.6	482
bta-miR-496	-15.5	499
bta-miR-496	-15.4	72
bta-miR-496	-15.3	576
bta-miR-496	-14.6	408
bta-miR-496	-14.4	126
bta-miR-496	-14.4	265
bta-miR-496	-13.6	370
bta-miR-496	-13.5	101
bta-miR-496	-13.2	737
bta-miR-496	-13.2	450
bta-miR-496	-13.1	545
bta-miR-496	-13.0	424
bta-miR-496	-12.8	155
bta-miR-496	-12.4	51
bta-miR-496	-12.2	23
bta-miR-496	-12.1	598
bta-miR-496	-11.8	176
bta-miR-496	-11.7	38
bta-miR-496	-11.7	563
bta-miR-496	-10.8	1
bta-miR-496	-10.8	523
bta-miR-496	-10.6	292
bta-miR-496	-10.4	119
bta-miR-496	-10.1	429
bta-miR-496	-10.1	698
bta-miR-500	-30.1	161
bta-miR-500	-29.0	55
bta-miR-500	-26.0	578
bta-miR-500	-21.7	259
bta-miR-500	-20.9	396
bta-miR-500	-20.3	429
bta-miR-500	-20.3	5
bta-miR-500	-20.0	473
bta-miR-500	-18.5	211
bta-miR-500	-18.3	616
bta-miR-500	-17.6	109
bta-miR-500	-17.4	364
bta-miR-500	-17.1	186

bta-miR-500	-16.0	30
bta-miR-500	-15.5	139
bta-miR-500	-14.4	694
bta-miR-500	-14.3	657
bta-miR-500	-14.2	236
bta-miR-500	-14.0	73
bta-miR-500	-13.8	317
bta-miR-500	-13.2	459
bta-miR-500	-13.1	326
bta-miR-500	-12.8	514
bta-miR-500	-12.3	567
bta-miR-500	-12.2	132
bta-miR-500	-11.6	739
bta-miR-500	-11.5	92
bta-miR-500	-10.7	540
bta-miR-500	-10.3	121
bta-miR-500	-10.2	330
bta-miR-502a	-25.0	1
bta-miR-502a	-22.9	356
bta-miR-502a	-22.2	498
bta-miR-502a	-21.9	93
bta-miR-502a	-21.9	464
bta-miR-502a	-21.0	580
bta-miR-502a	-20.9	233
bta-miR-502a	-19.6	27
bta-miR-502a	-19.5	404
bta-miR-502a	-18.9	163
bta-miR-502a	-18.5	187
bta-miR-502a	-18.1	616
bta-miR-502a	-17.7	258
bta-miR-502a	-17.4	54
bta-miR-502a	-17.2	426
bta-miR-502a	-15.8	276
bta-miR-502a	-15.6	313
bta-miR-502a	-15.3	635
bta-miR-502a	-15.1	209
bta-miR-502a	-14.9	730
bta-miR-502a	-14.9	130
bta-miR-502a	-14.7	566
bta-miR-502a	-14.6	537
bta-miR-502a	-13.9	686
bta-miR-502a	-13.9	660
bta-miR-502a	-13.1	550
bta-miR-502a	-12.2	293
bta-miR-502a	-11.9	389
bta-miR-502a	-11.8	711
bta-miR-502a	-11.3	328
bta-miR-502a	-11.1	81
bta-miR-502a	-10.6	337
bta-miR-502a	-10.3	453
bta-miR-502b	-23.1	163
bta-miR-502b	-22.5	401
bta-miR-502b	-22.0	89
bta-miR-502b	-21.5	426
bta-miR-502b	-21.0	580
bta-miR-502b	-20.6	248
bta-miR-502b	-20.1	2
bta-miR-502b	-19.4	467
bta-miR-502b	-19.0	360

bta-miR-502b	-18.5	208
bta-miR-502b	-18.3	54
bta-miR-502b	-17.7	689
bta-miR-502b	-17.0	30
bta-miR-502b	-16.7	616
bta-miR-502b	-16.6	624
bta-miR-502b	-16.2	514
bta-miR-502b	-15.3	73
bta-miR-502b	-14.8	235
bta-miR-502b	-14.7	566
bta-miR-502b	-13.9	313
bta-miR-502b	-13.6	276
bta-miR-502b	-13.5	665
bta-miR-502b	-12.8	188
bta-miR-502b	-12.7	130
bta-miR-502b	-12.2	307
bta-miR-502b	-12.2	118
bta-miR-502b	-12.0	19
bta-miR-502b	-11.9	389
bta-miR-502b	-11.5	537
bta-miR-502b	-11.4	289
bta-miR-502b	-11.3	328
bta-miR-502b	-11.2	715
bta-miR-502b	-10.7	337
bta-miR-502b	-10.6	550
bta-miR-502b	-10.6	744
bta-miR-504	-29.1	615
bta-miR-504	-28.7	352
bta-miR-504	-22.9	559
bta-miR-504	-21.9	410
bta-miR-504	-21.8	66
bta-miR-504	-21.7	1
bta-miR-504	-21.6	100
bta-miR-504	-21.3	595
bta-miR-504	-20.6	372
bta-miR-504	-20.2	199
bta-miR-504	-20.0	265
bta-miR-504	-20.0	229
bta-miR-504	-19.1	480
bta-miR-504	-18.8	429
bta-miR-504	-18.0	654
bta-miR-504	-17.6	165
bta-miR-504	-17.4	517
bta-miR-504	-16.8	22
bta-miR-504	-16.6	451
bta-miR-504	-15.0	730
bta-miR-504	-14.1	584
bta-miR-504	-13.8	118
bta-miR-504	-12.6	185
bta-miR-504	-11.1	154
bta-miR-504	-10.7	697
bta-miR-504	-10.5	538
bta-miR-505	-27.5	598
bta-miR-505	-24.6	479
bta-miR-505	-23.6	68
bta-miR-505	-22.0	405
bta-miR-505	-21.2	506
bta-miR-505	-20.3	169
bta-miR-505	-20.2	366

bta-miR-505	-19.8	114
bta-miR-505	-18.7	232
bta-miR-505	-18.2	575
bta-miR-505	-18.1	631
bta-miR-505	-17.0	11
bta-miR-505	-16.1	28
bta-miR-505	-16.1	338
bta-miR-505	-15.7	453
bta-miR-505	-14.3	269
bta-miR-505	-14.0	426
bta-miR-505	-12.5	102
bta-miR-505	-12.1	49
bta-miR-505	-11.9	197
bta-miR-505	-11.9	558
bta-miR-505	-11.5	539
bta-miR-505	-11.4	663
bta-miR-505	-11.2	737
bta-miR-505	-10.1	710
bta-miR-505	-10.0	88
bta-miR-539	-24.7	568
bta-miR-539	-21.8	602
bta-miR-539	-21.0	167
bta-miR-539	-19.2	381
bta-miR-539	-18.1	271
bta-miR-539	-16.9	654
bta-miR-539	-16.5	630
bta-miR-539	-16.5	206
bta-miR-539	-16.0	488
bta-miR-539	-15.9	122
bta-miR-539	-15.2	425
bta-miR-539	-15.0	64
bta-miR-539	-14.8	364
bta-miR-539	-14.7	30
bta-miR-539	-14.1	473
bta-miR-539	-13.9	148
bta-miR-539	-13.9	408
bta-miR-539	-13.6	304
bta-miR-539	-13.4	702
bta-miR-539	-13.3	593
bta-miR-539	-13.0	10
bta-miR-539	-12.6	106
bta-miR-539	-11.9	244
bta-miR-539	-11.3	160
bta-miR-539	-10.8	326
bta-miR-541	-33.7	11
bta-miR-541	-32.4	414
bta-miR-541	-25.3	575
bta-miR-541	-25.2	84
bta-miR-541	-24.1	481
bta-miR-541	-23.7	269
bta-miR-541	-22.4	154
bta-miR-541	-21.8	449
bta-miR-541	-21.6	369
bta-miR-541	-21.4	109
bta-miR-541	-21.3	622
bta-miR-541	-20.9	219
bta-miR-541	-20.9	308
bta-miR-541	-18.5	344
bta-miR-541	-17.8	539

bta-miR-541	-17.8	38
bta-miR-541	-17.4	654
bta-miR-541	-16.0	518
bta-miR-541	-15.7	604
bta-miR-541	-15.2	177
bta-miR-541	-14.6	389
bta-miR-541	-14.3	59
bta-miR-541	-14.0	687
bta-miR-541	-14.0	132
bta-miR-541	-13.9	737
bta-miR-541	-13.7	708
bta-miR-541	-13.5	257
bta-miR-541	-11.6	142
bta-miR-541	-10.7	199
bta-miR-541	-10.6	1
bta-miR-541	-10.5	566
bta-miR-541	-10.4	330
bta-miR-543	-20.8	506
bta-miR-543	-19.4	16
bta-miR-543	-19.2	350
bta-miR-543	-19.0	574
bta-miR-543	-18.4	654
bta-miR-543	-17.4	613
bta-miR-543	-17.0	167
bta-miR-543	-17.0	450
bta-miR-543	-16.8	68
bta-miR-543	-16.8	479
bta-miR-543	-15.6	544
bta-miR-543	-14.7	279
bta-miR-543	-14.3	232
bta-miR-543	-14.2	113
bta-miR-543	-14.2	405
bta-miR-543	-14.0	36
bta-miR-543	-14.0	560
bta-miR-543	-13.0	380
bta-miR-543	-12.6	197
bta-miR-543	-12.4	429
bta-miR-543	-11.8	341
bta-miR-543	-11.6	736
bta-miR-543	-10.8	265
bta-miR-543	-10.6	326
bta-miR-543	-10.4	178
bta-miR-543	-10.1	218
bta-miR-543	-10.0	153
bta-miR-544b	-16.4	434
bta-miR-544b	-16.0	600
bta-miR-544b	-14.8	84
bta-miR-544b	-14.2	2
bta-miR-544b	-14.2	184
bta-miR-544b	-13.8	455
bta-miR-544b	-13.4	231
bta-miR-544b	-13.4	345
bta-miR-544b	-13.2	249
bta-miR-544b	-12.7	281
bta-miR-544b	-12.6	66
bta-miR-544b	-12.5	500
bta-miR-544b	-12.3	371
bta-miR-544b	-12.2	653
bta-miR-544b	-11.7	546

bta-miR-544b	-11.7	570
bta-miR-544b	-11.5	414
bta-miR-544b	-11.1	701
bta-miR-544b	-10.7	627
bta-miR-544b	-10.4	114
bta-miR-544a	-19.7	178
bta-miR-544a	-17.6	414
bta-miR-544a	-17.3	71
bta-miR-544a	-17.3	585
bta-miR-544a	-17.2	13
bta-miR-544a	-15.0	695
bta-miR-544a	-14.5	508
bta-miR-544a	-14.1	249
bta-miR-544a	-13.4	546
bta-miR-544a	-13.4	642
bta-miR-544a	-13.0	281
bta-miR-544a	-13.0	213
bta-miR-544a	-12.9	481
bta-miR-544a	-12.6	371
bta-miR-544a	-12.5	437
bta-miR-544a	-12.5	738
bta-miR-544a	-12.4	114
bta-miR-544a	-11.9	57
bta-miR-544a	-11.9	345
bta-miR-544a	-11.9	229
bta-miR-544a	-11.8	614
bta-miR-544a	-10.7	156
bta-miR-544a	-10.5	624
bta-miR-544a	-10.2	657
bta-miR-544a	-10.0	455
bta-miR-551a	-24.5	478
bta-miR-551a	-24.1	598
bta-miR-551a	-22.1	220
bta-miR-551a	-22.0	405
bta-miR-551a	-21.9	69
bta-miR-551a	-21.1	25
bta-miR-551a	-19.1	629
bta-miR-551a	-17.9	570
bta-miR-551a	-17.8	731
bta-miR-551a	-17.7	439
bta-miR-551a	-17.0	9
bta-miR-551a	-16.6	102
bta-miR-551a	-16.2	338
bta-miR-551a	-15.4	270
bta-miR-551a	-14.6	166
bta-miR-551a	-14.0	366
bta-miR-551a	-12.4	508
bta-miR-551a	-11.8	464
bta-miR-551a	-11.3	124
bta-miR-551a	-11.1	697
bta-miR-551a	-10.8	240
bta-miR-551a	-10.6	49
bta-miR-551a	-10.3	542
bta-miR-551a	-10.2	197
bta-miR-551a	-10.1	621
bta-miR-551b	-29.8	403
bta-miR-551b	-25.1	27
bta-miR-551b	-23.6	644
bta-miR-551b	-23.2	92

bta-miR-551b	-22.2	591
bta-miR-551b	-21.9	454
bta-miR-551b	-20.8	216
bta-miR-551b	-20.5	70
bta-miR-551b	-20.2	337
bta-miR-551b	-19.7	730
bta-miR-551b	-19.3	6
bta-miR-551b	-18.5	561
bta-miR-551b	-17.7	369
bta-miR-551b	-17.4	507
bta-miR-551b	-16.2	166
bta-miR-551b	-15.0	257
bta-miR-551b	-14.6	432
bta-miR-551b	-13.4	280
bta-miR-551b	-12.8	543
bta-miR-551b	-12.4	124
bta-miR-551b	-12.1	697
bta-miR-551b	-11.6	629
bta-miR-551b	-11.1	486
bta-miR-551b	-10.9	187
bta-miR-562	-19.7	78
bta-miR-562	-18.9	265
bta-miR-562	-17.2	616
bta-miR-562	-16.9	497
bta-miR-562	-16.7	555
bta-miR-562	-16.3	411
bta-miR-562	-16.1	727
bta-miR-562	-16.0	176
bta-miR-562	-15.7	444
bta-miR-562	-15.3	375
bta-miR-562	-15.2	106
bta-miR-562	-14.4	228
bta-miR-562	-13.4	66
bta-miR-562	-13.0	661
bta-miR-562	-12.9	15
bta-miR-562	-12.8	599
bta-miR-562	-12.7	478
bta-miR-562	-12.6	524
bta-miR-562	-12.6	576
bta-miR-562	-12.4	355
bta-miR-562	-12.4	40
bta-miR-562	-12.0	199
bta-miR-562	-11.6	423
bta-miR-562	-11.5	165
bta-miR-562	-11.3	464
bta-miR-562	-11.3	632
bta-miR-562	-10.4	433
bta-miR-568	-16.8	543
bta-miR-568	-14.8	524
bta-miR-568	-14.6	15
bta-miR-568	-14.1	377
bta-miR-568	-12.2	658
bta-miR-568	-11.5	488
bta-miR-568	-11.3	266
bta-miR-568	-10.6	583
bta-miR-568	-10.5	353
bta-miR-568	-10.3	293
bta-miR-582	-23.0	411
bta-miR-582	-22.2	12

bta-miR-582	-20.6	358
bta-miR-582	-17.8	63
bta-miR-582	-17.7	738
bta-miR-582	-16.7	448
bta-miR-582	-16.3	658
bta-miR-582	-15.9	472
bta-miR-582	-15.5	632
bta-miR-582	-15.2	555
bta-miR-582	-14.8	171
bta-miR-582	-12.9	598
bta-miR-582	-12.5	274
bta-miR-582	-12.5	112
bta-miR-582	-12.4	340
bta-miR-582	-11.9	204
bta-miR-582	-11.5	37
bta-miR-582	-11.5	700
bta-miR-582	-11.4	517
bta-miR-582	-10.5	381
bta-miR-582	-10.4	434
bta-miR-582	-10.2	504
bta-miR-582	-10.0	730
bta-miR-584	-26.5	107
bta-miR-584	-25.3	258
bta-miR-584	-21.3	463
bta-miR-584	-21.0	410
bta-miR-584	-20.3	9
bta-miR-584	-20.2	597
bta-miR-584	-19.9	165
bta-miR-584	-19.7	623
bta-miR-584	-18.4	137
bta-miR-584	-18.3	33
bta-miR-584	-18.2	234
bta-miR-584	-17.0	503
bta-miR-584	-16.7	655
bta-miR-584	-16.6	576
bta-miR-584	-16.5	356
bta-miR-584	-16.3	60
bta-miR-584	-15.5	386
bta-miR-584	-15.2	326
bta-miR-584	-15.0	211
bta-miR-584	-15.0	182
bta-miR-584	-14.6	545
bta-miR-584	-14.3	80
bta-miR-584	-14.1	432
bta-miR-584	-13.2	123
bta-miR-584	-12.4	639
bta-miR-584	-12.4	692
bta-miR-584	-12.4	566
bta-miR-584	-11.5	737
bta-miR-584	-11.4	98
bta-miR-584	-11.2	310
bta-miR-584	-10.7	282
bta-miR-584	-10.4	247
bta-miR-592	-15.4	353
bta-miR-592	-15.4	8
bta-miR-592	-15.4	383
bta-miR-592	-15.3	649
bta-miR-592	-15.2	577
bta-miR-592	-15.1	53

bta-miR-592	-14.5	271
bta-miR-592	-14.5	488
bta-miR-592	-13.6	535
bta-miR-592	-13.5	516
bta-miR-592	-13.1	206
bta-miR-592	-13.1	618
bta-miR-592	-12.0	106
bta-miR-592	-11.7	565
bta-miR-592	-11.2	428
bta-miR-592	-11.2	144
bta-miR-592	-10.5	180
bta-miR-592	-10.4	738
bta-miR-592	-10.3	469
bta-miR-599	-17.7	113
bta-miR-599	-17.5	344
bta-miR-599	-15.9	404
bta-miR-599	-15.2	551
bta-miR-599	-14.7	591
bta-miR-599	-14.7	178
bta-miR-599	-13.5	93
bta-miR-599	-13.0	376
bta-miR-599	-12.6	154
bta-miR-599	-12.2	7
bta-miR-599	-12.1	730
bta-miR-599	-11.6	39
bta-miR-599	-11.5	267
bta-miR-599	-11.4	642
bta-miR-599	-11.1	629
bta-miR-599	-10.8	504
bta-miR-599	-10.8	437
bta-miR-599	-10.6	217
bta-miR-599	-10.2	695
bta-miR-599	-10.0	662
bta-miR-615	-31.1	460
bta-miR-615	-29.8	4
bta-miR-615	-29.4	592
bta-miR-615	-28.4	60
bta-miR-615	-28.3	97
bta-miR-615	-26.9	401
bta-miR-615	-25.9	215
bta-miR-615	-25.5	311
bta-miR-615	-24.7	168
bta-miR-615	-24.4	344
bta-miR-615	-23.9	258
bta-miR-615	-22.1	490
bta-miR-615	-22.1	566
bta-miR-615	-20.5	432
bta-miR-615	-19.7	650
bta-miR-615	-18.7	631
bta-miR-615	-18.4	692
bta-miR-615	-18.0	80
bta-miR-615	-17.9	140
bta-miR-615	-16.9	194
bta-miR-615	-15.2	739
bta-miR-615	-14.0	367
bta-miR-615	-13.8	515
bta-miR-615	-13.0	280
bta-miR-615	-12.9	378
bta-miR-615	-12.2	37

bta-miR-615	-12.0	551
bta-miR-615	-10.8	584
bta-miR-615	-10.2	291
bta-miR-628	-19.3	474
bta-miR-628	-18.0	261
bta-miR-628	-17.5	625
bta-miR-628	-16.2	14
bta-miR-628	-15.6	363
bta-miR-628	-15.4	32
bta-miR-628	-15.3	512
bta-miR-628	-15.2	74
bta-miR-628	-14.4	390
bta-miR-628	-14.3	190
bta-miR-628	-13.6	210
bta-miR-628	-13.5	58
bta-miR-628	-12.7	533
bta-miR-628	-12.5	110
bta-miR-628	-12.3	578
bta-miR-628	-11.8	7
bta-miR-628	-11.6	169
bta-miR-628	-11.4	284
bta-miR-628	-11.1	681
bta-miR-628	-10.7	496
bta-miR-628	-10.6	95
bta-miR-628	-10.3	416
bta-miR-628	-10.1	716
bta-miR-631	-29.0	560
bta-miR-631	-25.1	403
bta-miR-631	-23.8	66
bta-miR-631	-23.0	615
bta-miR-631	-22.8	13
bta-miR-631	-22.2	448
bta-miR-631	-20.8	112
bta-miR-631	-19.9	477
bta-miR-631	-19.5	216
bta-miR-631	-18.9	660
bta-miR-631	-18.3	267
bta-miR-631	-18.2	586
bta-miR-631	-17.5	154
bta-miR-631	-16.8	356
bta-miR-631	-15.1	432
bta-miR-631	-15.1	185
bta-miR-631	-14.3	92
bta-miR-631	-14.3	49
bta-miR-631	-13.6	386
bta-miR-631	-13.3	504
bta-miR-631	-13.1	650
bta-miR-631	-12.5	242
bta-miR-631	-12.5	730
bta-miR-631	-12.0	337
bta-miR-631	-11.4	685
bta-miR-631	-11.2	1
bta-miR-631	-11.0	281
bta-miR-631	-10.7	471
bta-miR-631	-10.3	634
bta-miR-631	-10.1	294
bta-miR-653	-18.4	605
bta-miR-653	-17.2	64
bta-miR-653	-16.6	198

bta-miR-653	-15.3	170
bta-miR-653	-15.1	238
bta-miR-653	-14.0	366
bta-miR-653	-13.6	1
bta-miR-653	-13.3	280
bta-miR-653	-13.2	439
bta-miR-653	-12.2	219
bta-miR-653	-12.0	635
bta-miR-653	-11.9	105
bta-miR-653	-11.6	340
bta-miR-653	-11.4	494
bta-miR-653	-11.3	28
bta-miR-653	-11.2	570
bta-miR-653	-10.9	80
bta-miR-653	-10.8	516
bta-miR-653	-10.7	229
bta-miR-653	-10.4	738
bta-miR-653	-10.4	410
bta-miR-653	-10.1	158
bta-miR-653	-10.1	548
bta-miR-654	-25.4	615
bta-miR-654	-20.5	11
bta-miR-654	-19.3	174
bta-miR-654	-18.6	574
bta-miR-654	-18.0	478
bta-miR-654	-17.5	218
bta-miR-654	-16.5	36
bta-miR-654	-16.4	83
bta-miR-654	-16.3	406
bta-miR-654	-15.1	337
bta-miR-654	-14.9	266
bta-miR-654	-14.7	64
bta-miR-654	-14.6	659
bta-miR-654	-14.6	198
bta-miR-654	-14.3	448
bta-miR-654	-14.0	365
bta-miR-654	-12.7	125
bta-miR-654	-12.7	738
bta-miR-654	-12.1	546
bta-miR-654	-11.8	153
bta-miR-654	-11.7	1
bta-miR-654	-10.5	500
bta-miR-655	-19.1	12
bta-miR-655	-18.8	449
bta-miR-655	-16.5	613
bta-miR-655	-16.0	499
bta-miR-655	-15.7	659
bta-miR-655	-15.5	418
bta-miR-655	-15.3	239
bta-miR-655	-15.1	348
bta-miR-655	-15.0	80
bta-miR-655	-14.9	174
bta-miR-655	-13.9	265
bta-miR-655	-13.9	737
bta-miR-655	-13.9	479
bta-miR-655	-13.8	37
bta-miR-655	-13.7	201
bta-miR-655	-13.6	575
bta-miR-655	-13.3	220

bta-miR-655	-12.9	440
bta-miR-655	-12.8	539
bta-miR-655	-12.5	645
bta-miR-655	-12.3	114
bta-miR-655	-12.1	407
bta-miR-655	-11.9	464
bta-miR-655	-11.5	65
bta-miR-655	-10.8	154
bta-miR-655	-10.8	280
bta-miR-655	-10.6	125
bta-miR-655	-10.6	340
bta-miR-656	-18.5	68
bta-miR-656	-17.8	614
bta-miR-656	-16.1	575
bta-miR-656	-15.4	411
bta-miR-656	-14.8	11
bta-miR-656	-14.3	174
bta-miR-656	-13.4	544
bta-miR-656	-13.3	447
bta-miR-656	-13.2	737
bta-miR-656	-12.9	496
bta-miR-656	-12.6	357
bta-miR-656	-12.4	654
bta-miR-656	-12.2	434
bta-miR-656	-11.9	280
bta-miR-656	-11.9	241
bta-miR-656	-11.5	218
bta-miR-656	-10.8	115
bta-miR-656	-10.7	2
bta-miR-656	-10.1	479
bta-miR-656	-10.0	28
bta-miR-658	-29.3	408
bta-miR-658	-28.7	563
bta-miR-658	-27.3	237
bta-miR-658	-26.7	483
bta-miR-658	-26.0	73
bta-miR-658	-25.9	169
bta-miR-658	-25.6	126
bta-miR-658	-25.4	34
bta-miR-658	-24.3	367
bta-miR-658	-24.3	454
bta-miR-658	-23.4	269
bta-miR-658	-22.5	669
bta-miR-658	-21.2	622
bta-miR-658	-20.3	311
bta-miR-658	-18.4	702
bta-miR-658	-18.2	9
bta-miR-658	-18.0	341
bta-miR-658	-17.1	156
bta-miR-658	-16.5	200
bta-miR-658	-15.0	391
bta-miR-658	-14.8	734
bta-miR-658	-13.7	595
bta-miR-658	-13.5	541
bta-miR-658	-12.0	649
bta-miR-658	-11.8	229
bta-miR-665	-28.0	418
bta-miR-665	-27.8	69
bta-miR-665	-26.4	613

bta-miR-665	-26.0	479
bta-miR-665	-22.2	448
bta-miR-665	-20.8	268
bta-miR-665	-20.5	506
bta-miR-665	-19.9	576
bta-miR-665	-19.8	12
bta-miR-665	-19.6	109
bta-miR-665	-18.1	175
bta-miR-665	-16.9	308
bta-miR-665	-16.7	395
bta-miR-665	-15.9	631
bta-miR-665	-15.3	41
bta-miR-665	-15.2	663
bta-miR-665	-14.9	361
bta-miR-665	-14.4	232
bta-miR-665	-13.6	559
bta-miR-665	-13.5	155
bta-miR-665	-13.1	739
bta-miR-665	-13.1	532
bta-miR-665	-13.1	599
bta-miR-665	-13.0	205
bta-miR-665	-12.5	473
bta-miR-665	-12.4	693
bta-miR-665	-11.5	126
bta-miR-665	-10.8	371
bta-miR-665	-10.5	261
bta-miR-665	-10.2	30
bta-miR-670	-23.6	53
bta-miR-670	-21.1	594
bta-miR-670	-20.4	161
bta-miR-670	-19.2	259
bta-miR-670	-18.1	483
bta-miR-670	-17.0	119
bta-miR-670	-17.0	295
bta-miR-670	-16.9	616
bta-miR-670	-16.1	88
bta-miR-670	-15.3	408
bta-miR-670	-15.1	207
bta-miR-670	-15.0	13
bta-miR-670	-14.8	431
bta-miR-670	-14.6	144
bta-miR-670	-14.4	461
bta-miR-670	-14.4	649
bta-miR-670	-13.7	365
bta-miR-670	-13.7	695
bta-miR-670	-13.4	569
bta-miR-670	-13.0	520
bta-miR-670	-12.1	73
bta-miR-670	-11.5	666
bta-miR-670	-11.1	382
bta-miR-670	-10.8	540
bta-miR-670	-10.7	181
bta-miR-670	-10.2	341
bta-miR-670	-10.1	740
bta-miR-670	-10.0	326
bta-miR-671	-33.4	391
bta-miR-671	-31.2	461
bta-miR-671	-30.7	107
bta-miR-671	-30.1	248

bta-miR-671	-30.0	164
bta-miR-671	-29.0	45
bta-miR-671	-28.6	503
bta-miR-671	-26.2	564
bta-miR-671	-25.0	622
bta-miR-671	-24.2	132
bta-miR-671	-23.2	317
bta-miR-671	-21.0	80
bta-miR-671	-20.8	205
bta-miR-671	-20.6	27
bta-miR-671	-20.4	692
bta-miR-671	-19.4	361
bta-miR-671	-19.1	4
bta-miR-671	-18.6	596
bta-miR-671	-18.4	653
bta-miR-671	-18.3	429
bta-miR-671	-15.8	487
bta-miR-671	-13.6	299
bta-miR-671	-13.3	615
bta-miR-671	-13.1	669
bta-miR-671	-12.8	96
bta-miR-671	-12.7	740
bta-miR-671	-11.8	453
bta-miR-671	-11.5	125
bta-miR-671	-11.0	342
bta-miR-671	-10.8	533
bta-miR-671	-10.5	378
bta-miR-708	-26.6	12
bta-miR-708	-26.0	168
bta-miR-708	-22.7	262
bta-miR-708	-22.4	409
bta-miR-708	-22.4	609
bta-miR-708	-19.5	362
bta-miR-708	-19.5	545
bta-miR-708	-19.3	514
bta-miR-708	-19.0	198
bta-miR-708	-18.8	98
bta-miR-708	-18.1	34
bta-miR-708	-18.0	450
bta-miR-708	-17.9	72
bta-miR-708	-16.8	236
bta-miR-708	-16.0	495
bta-miR-708	-15.5	572
bta-miR-708	-15.4	650
bta-miR-708	-15.1	125
bta-miR-708	-15.1	692
bta-miR-708	-13.6	629
bta-miR-708	-13.5	474
bta-miR-708	-13.0	142
bta-miR-708	-12.3	737
bta-miR-708	-12.0	1
bta-miR-708	-12.0	583
bta-miR-708	-11.8	330
bta-miR-708	-10.1	62
bta-miR-744	-30.7	415
bta-miR-744	-28.0	13
bta-miR-744	-27.6	86
bta-miR-744	-26.8	457
bta-miR-744	-26.0	156

bta-miR-744	-24.7	234
bta-miR-744	-24.6	116
bta-miR-744	-23.4	547
bta-miR-744	-22.8	356
bta-miR-744	-22.6	380
bta-miR-744	-21.1	38
bta-miR-744	-21.1	185
bta-miR-744	-20.2	617
bta-miR-744	-19.8	493
bta-miR-744	-19.0	585
bta-miR-744	-18.9	250
bta-miR-744	-18.4	292
bta-miR-744	-17.8	654
bta-miR-744	-17.6	322
bta-miR-744	-17.6	66
bta-miR-744	-15.9	401
bta-miR-744	-15.3	568
bta-miR-744	-13.7	276
bta-miR-744	-13.6	737
bta-miR-744	-13.6	526
bta-miR-744	-13.4	697
bta-miR-744	-13.0	139
bta-miR-744	-11.1	209
bta-miR-744	-10.3	342
bta-miR-744	-10.2	4
bta-miR-758	-22.5	614
bta-miR-758	-21.4	411
bta-miR-758	-20.2	496
bta-miR-758	-20.1	38
bta-miR-758	-19.1	370
bta-miR-758	-19.1	265
bta-miR-758	-18.6	478
bta-miR-758	-18.5	80
bta-miR-758	-18.4	566
bta-miR-758	-18.1	113
bta-miR-758	-18.0	448
bta-miR-758	-17.7	599
bta-miR-758	-17.0	12
bta-miR-758	-15.7	228
bta-miR-758	-15.5	738
bta-miR-758	-14.0	661
bta-miR-758	-13.4	182
bta-miR-758	-13.3	62
bta-miR-758	-12.2	143
bta-miR-758	-12.1	126
bta-miR-758	-11.9	155
bta-miR-758	-11.8	341
bta-miR-758	-11.0	26
bta-miR-758	-10.7	709
bta-miR-758	-10.3	358
bta-miR-758	-10.2	252
bta-miR-759	-21.0	548
bta-miR-759	-19.4	8
bta-miR-759	-16.9	44
bta-miR-759	-15.8	485
bta-miR-759	-15.5	377
bta-miR-759	-15.3	651
bta-miR-759	-14.4	417
bta-miR-759	-14.2	332

bta-miR-759	-13.9	77
bta-miR-759	-13.3	529
bta-miR-759	-13.2	610
bta-miR-759	-13.1	252
bta-miR-759	-12.8	100
bta-miR-759	-12.6	199
bta-miR-759	-12.3	566
bta-miR-759	-12.1	684
bta-miR-759	-11.9	221
bta-miR-759	-11.9	277
bta-miR-759	-11.3	436
bta-miR-759	-10.9	519
bta-miR-759	-10.8	393
bta-miR-759	-10.7	736
bta-miR-759	-10.7	473
bta-miR-759	-10.3	628
bta-miR-759	-10.0	177
bta-miR-760	-29.1	609
bta-miR-760	-28.1	418
bta-miR-760	-25.9	481
bta-miR-760	-25.4	71
bta-miR-760	-21.3	12
bta-miR-760	-19.7	576
bta-miR-760	-18.1	155
bta-miR-760	-17.6	359
bta-miR-760	-17.0	211
bta-miR-760	-16.8	262
bta-miR-760	-15.6	114
bta-miR-760	-15.2	518
bta-miR-760	-14.3	654
bta-miR-760	-13.9	454
bta-miR-760	-13.2	740
bta-miR-760	-13.0	389
bta-miR-760	-12.9	539
bta-miR-760	-12.3	698
bta-miR-760	-12.2	39
bta-miR-760	-11.7	231
bta-miR-760	-10.4	89
bta-miR-760	-10.1	185
bta-miR-760	-10.0	190
bta-miR-761	-25.4	96
bta-miR-761	-24.8	42
bta-miR-761	-22.0	377
bta-miR-761	-21.7	7
bta-miR-761	-21.7	543
bta-miR-761	-20.9	658
bta-miR-761	-20.0	566
bta-miR-761	-19.8	412
bta-miR-761	-19.4	257
bta-miR-761	-18.5	356
bta-miR-761	-18.3	161
bta-miR-761	-17.3	463
bta-miR-761	-17.2	199
bta-miR-761	-16.4	235
bta-miR-761	-16.2	497
bta-miR-761	-15.3	614
bta-miR-761	-14.8	126
bta-miR-761	-14.5	69
bta-miR-761	-13.6	293

bta-miR-761	-13.6	739
bta-miR-761	-13.5	311
bta-miR-761	-13.3	326
bta-miR-761	-13.0	430
bta-miR-761	-12.9	146
bta-miR-761	-12.8	644
bta-miR-761	-12.5	174
bta-miR-761	-12.0	526
bta-miR-761	-11.8	594
bta-miR-761	-11.1	30
bta-miR-761	-10.0	137
bta-miR-763	-28.5	408
bta-miR-763	-27.5	457
bta-miR-763	-26.4	8
bta-miR-763	-26.1	563
bta-miR-763	-25.5	136
bta-miR-763	-24.9	504
bta-miR-763	-23.3	109
bta-miR-763	-22.9	244
bta-miR-763	-21.4	51
bta-miR-763	-21.0	185
bta-miR-763	-20.8	698
bta-miR-763	-19.5	86
bta-miR-763	-19.4	381
bta-miR-763	-18.6	624
bta-miR-763	-18.1	483
bta-miR-763	-17.9	308
bta-miR-763	-17.6	354
bta-miR-763	-17.1	207
bta-miR-763	-16.4	655
bta-miR-763	-15.2	275
bta-miR-763	-14.9	594
bta-miR-763	-14.6	534
bta-miR-763	-14.2	328
bta-miR-763	-14.1	231
bta-miR-763	-11.4	519
bta-miR-763	-10.9	616
bta-miR-763	-10.8	676
bta-miR-763	-10.0	551
bta-miR-767	-26.0	95
bta-miR-767	-24.7	18
bta-miR-767	-22.3	584
bta-miR-767	-21.8	359
bta-miR-767	-21.7	62
bta-miR-767	-21.2	488
bta-miR-767	-19.9	256
bta-miR-767	-18.9	462
bta-miR-767	-18.9	408
bta-miR-767	-18.8	562
bta-miR-767	-18.4	126
bta-miR-767	-18.2	618
bta-miR-767	-17.5	235
bta-miR-767	-16.5	156
bta-miR-767	-16.0	729
bta-miR-767	-15.9	644
bta-miR-767	-15.8	185
bta-miR-767	-15.3	436
bta-miR-767	-14.9	518
bta-miR-767	-14.9	305

bta-miR-767	-14.1	207
bta-miR-767	-14.0	3
bta-miR-767	-13.9	697
bta-miR-767	-13.3	388
bta-miR-767	-12.9	45
bta-miR-767	-12.8	344
bta-miR-767	-11.9	664
bta-miR-767	-10.4	293
bta-miR-769	-25.4	22
bta-miR-769	-23.8	605
bta-miR-769	-23.8	419
bta-miR-769	-23.3	464
bta-miR-769	-23.0	559
bta-miR-769	-21.3	262
bta-miR-769	-21.3	155
bta-miR-769	-21.3	65
bta-miR-769	-20.0	361
bta-miR-769	-19.9	198
bta-miR-769	-19.9	97
bta-miR-769	-18.7	586
bta-miR-769	-17.9	234
bta-miR-769	-17.8	125
bta-miR-769	-17.3	698
bta-miR-769	-16.1	504
bta-miR-769	-16.0	400
bta-miR-769	-15.4	9
bta-miR-769	-14.4	627
bta-miR-769	-14.2	645
bta-miR-769	-13.0	737
bta-miR-769	-12.8	299
bta-miR-769	-12.6	184
bta-miR-769	-12.5	344
bta-miR-769	-12.1	487
bta-miR-769	-11.8	454
bta-miR-769	-11.5	50
bta-miR-769	-10.3	327
bta-miR-769	-10.0	320
bta-miR-769	-10.0	532
bta-miR-873	-27.8	68
bta-miR-873	-23.1	348
bta-miR-873	-22.5	620
bta-miR-873	-22.4	268
bta-miR-873	-22.4	450
bta-miR-873	-20.8	479
bta-miR-873	-20.1	371
bta-miR-873	-19.6	570
bta-miR-873	-19.5	412
bta-miR-873	-18.6	114
bta-miR-873	-17.5	41
bta-miR-873	-17.0	219
bta-miR-873	-16.9	176
bta-miR-873	-16.7	598
bta-miR-873	-15.8	154
bta-miR-873	-15.4	506
bta-miR-873	-15.0	11
bta-miR-873	-14.9	737
bta-miR-873	-14.8	129
bta-miR-873	-14.1	702
bta-miR-873	-13.3	397

bta-miR-873	-12.7	308
bta-miR-873	-12.2	649
bta-miR-873	-12.1	544
bta-miR-873	-12.0	145
bta-miR-873	-12.0	239
bta-miR-873	-11.0	613
bta-miR-873	-10.3	680
bta-miR-873	-10.2	431
bta-miR-874	-30.5	258
bta-miR-874	-29.9	463
bta-miR-874	-29.5	576
bta-miR-874	-27.6	4
bta-miR-874	-27.1	395
bta-miR-874	-26.0	609
bta-miR-874	-24.1	39
bta-miR-874	-23.5	498
bta-miR-874	-22.8	422
bta-miR-874	-22.7	107
bta-miR-874	-19.7	165
bta-miR-874	-18.4	356
bta-miR-874	-18.0	625
bta-miR-874	-17.1	191
bta-miR-874	-16.4	233
bta-miR-874	-16.3	662
bta-miR-874	-16.1	87
bta-miR-874	-15.8	140
bta-miR-874	-15.4	307
bta-miR-874	-15.0	209
bta-miR-874	-13.8	545
bta-miR-874	-13.7	247
bta-miR-874	-13.3	708
bta-miR-874	-13.3	741
bta-miR-874	-13.2	639
bta-miR-874	-12.2	692
bta-miR-874	-11.8	323
bta-miR-874	-11.1	133
bta-miR-874	-10.4	27
bta-miR-874	-10.1	386
bta-miR-875	-21.1	5
bta-miR-875	-19.1	431
bta-miR-875	-18.6	594
bta-miR-875	-18.0	402
bta-miR-875	-17.2	473
bta-miR-875	-16.7	549
bta-miR-875	-16.5	183
bta-miR-875	-16.0	210
bta-miR-875	-16.0	161
bta-miR-875	-15.9	60
bta-miR-875	-15.8	341
bta-miR-875	-15.5	363
bta-miR-875	-15.5	581
bta-miR-875	-15.3	91
bta-miR-875	-15.2	621
bta-miR-875	-14.6	489
bta-miR-875	-14.0	23
bta-miR-875	-14.0	261
bta-miR-875	-13.7	73
bta-miR-875	-13.5	510
bta-miR-875	-13.3	730

bta-miR-875	-11.5	229
bta-miR-875	-11.2	425
bta-miR-875	-11.2	148
bta-miR-875	-11.1	452
bta-miR-875	-10.7	384
bta-miR-875	-10.6	308
bta-miR-875	-10.5	642
bta-miR-876	-18.7	185
bta-miR-876	-18.6	220
bta-miR-876	-17.6	497
bta-miR-876	-16.8	17
bta-miR-876	-16.4	40
bta-miR-876	-16.4	464
bta-miR-876	-16.1	598
bta-miR-876	-15.9	617
bta-miR-876	-15.7	172
bta-miR-876	-15.5	113
bta-miR-876	-14.8	377
bta-miR-876	-14.5	299
bta-miR-876	-14.4	704
bta-miR-876	-14.1	145
bta-miR-876	-14.1	543
bta-miR-876	-14.0	422
bta-miR-876	-13.7	266
bta-miR-876	-13.4	567
bta-miR-876	-13.4	337
bta-miR-876	-13.4	658
bta-miR-876	-12.3	730
bta-miR-876	-11.9	94
bta-miR-876	-11.4	400
bta-miR-876	-11.2	449
bta-miR-876	-10.4	239
bta-miR-877	-26.0	362
bta-miR-877	-25.8	561
bta-miR-877	-23.1	33
bta-miR-877	-22.4	110
bta-miR-877	-22.3	461
bta-miR-877	-21.2	248
bta-miR-877	-21.1	168
bta-miR-877	-20.7	401
bta-miR-877	-18.1	206
bta-miR-877	-17.8	502
bta-miR-877	-17.8	692
bta-miR-877	-17.3	626
bta-miR-877	-17.2	668
bta-miR-877	-16.9	481
bta-miR-877	-16.6	423
bta-miR-877	-16.2	521
bta-miR-877	-16.0	84
bta-miR-877	-15.8	276
bta-miR-877	-14.8	740
bta-miR-877	-14.8	543
bta-miR-877	-14.0	307
bta-miR-877	-13.6	657
bta-miR-877	-13.5	330
bta-miR-877	-13.5	2
bta-miR-877	-11.2	236
bta-miR-877	-10.7	142
bta-miR-877	-10.6	321

bta-miR-877	-10.1	267
bta-miR-885	-30.0	604
bta-miR-885	-23.8	11
bta-miR-885	-21.8	66
bta-miR-885	-20.1	479
bta-miR-885	-20.0	411
bta-miR-885	-18.7	201
bta-miR-885	-18.1	622
bta-miR-885	-18.1	442
bta-miR-885	-16.9	349
bta-miR-885	-15.9	239
bta-miR-885	-15.0	575
bta-miR-885	-13.3	172
bta-miR-885	-13.2	654
bta-miR-885	-12.8	517
bta-miR-885	-11.3	376
bta-miR-885	-10.9	85
bta-miR-885	-10.9	270
bta-miR-885	-10.6	40
bta-miR-885	-10.4	154
bta-miR-885	-10.3	109
bta-miR-885	-10.2	539
bta-miR-885	-10.0	424
bta-miR-92a	-31.0	10
bta-miR-92a	-28.9	602
bta-miR-92a	-26.0	358
bta-miR-92a	-24.8	481
bta-miR-92a	-24.2	411
bta-miR-92a	-22.0	66
bta-miR-92a	-20.5	114
bta-miR-92a	-19.6	499
bta-miR-92a	-19.6	442
bta-miR-92a	-18.2	264
bta-miR-92a	-17.7	575
bta-miR-92a	-17.2	623
bta-miR-92a	-15.9	154
bta-miR-92a	-14.8	35
bta-miR-92a	-14.0	199
bta-miR-92a	-13.4	176
bta-miR-92a	-13.3	560
bta-miR-92a	-13.3	596
bta-miR-92a	-11.9	737
bta-miR-92a	-11.7	462
bta-miR-92a	-11.7	654
bta-miR-92a	-11.1	241
bta-miR-92a	-10.9	86
bta-miR-92a	-10.7	106
bta-miR-92a	-10.5	304
bta-miR-92a	-10.2	341
bta-miR-92b	-29.7	614
bta-miR-92b	-28.6	11
bta-miR-92b	-25.9	412
bta-miR-92b	-25.3	479
bta-miR-92b	-24.4	69
bta-miR-92b	-24.0	357
bta-miR-92b	-23.1	592
bta-miR-92b	-21.1	499
bta-miR-92b	-20.8	441
bta-miR-92b	-17.2	218

bta-miR-92b	-17.1	167
bta-miR-92b	-16.3	560
bta-miR-92b	-16.2	109
bta-miR-92b	-15.4	268
bta-miR-92b	-14.4	28
bta-miR-92b	-13.3	185
bta-miR-92b	-13.2	240
bta-miR-92b	-12.5	338
bta-miR-92b	-12.3	154
bta-miR-92b	-12.1	126
bta-miR-92b	-12.0	654
bta-miR-92b	-11.7	738
bta-miR-92b	-11.2	308
bta-miR-92b	-10.9	609
bta-miR-92b	-10.5	462
bta-miR-92b	-10.0	543
bta-miR-935	-29.4	614
bta-miR-935	-26.1	63
bta-miR-935	-22.9	393
bta-miR-935	-21.3	448
bta-miR-935	-18.3	171
bta-miR-935	-18.3	481
bta-miR-935	-17.3	265
bta-miR-935	-16.6	576
bta-miR-935	-16.0	17
bta-miR-935	-15.2	113
bta-miR-935	-15.1	653
bta-miR-935	-14.7	351
bta-miR-935	-14.5	198
bta-miR-935	-13.1	419
bta-miR-935	-12.9	539
bta-miR-935	-12.6	738
bta-miR-935	-12.6	228
bta-miR-935	-11.8	517
bta-miR-935	-10.9	84
bta-miR-935	-10.8	376
bta-miR-935	-10.0	154
bta-miR-940	-34.8	615
bta-miR-940	-31.3	411
bta-miR-940	-29.9	463
bta-miR-940	-28.1	62
bta-miR-940	-24.0	108
bta-miR-940	-24.0	167
bta-miR-940	-23.4	220
bta-miR-940	-23.2	497
bta-miR-940	-22.9	265
bta-miR-940	-22.4	438
bta-miR-940	-21.6	25
bta-miR-940	-20.5	357
bta-miR-940	-18.6	654
bta-miR-940	-17.5	280
bta-miR-940	-17.2	586
bta-miR-940	-16.3	370
bta-miR-940	-16.2	12
bta-miR-940	-15.2	559
bta-miR-940	-14.6	199
bta-miR-940	-13.6	97
bta-miR-940	-13.0	738
bta-miR-940	-12.6	324

bta-miR-940	-12.4	429
bta-miR-940	-11.8	155
bta-miR-940	-11.2	135
bta-miR-940	-10.0	330
bta-miR-95	-20.8	415
bta-miR-95	-19.8	344
bta-miR-95	-18.9	13
bta-miR-95	-18.6	585
bta-miR-95	-16.7	293
bta-miR-95	-16.6	262
bta-miR-95	-16.4	73
bta-miR-95	-16.0	488
bta-miR-95	-15.9	730
bta-miR-95	-15.3	624
bta-miR-95	-15.2	550
bta-miR-95	-15.0	156
bta-miR-95	-14.1	388
bta-miR-95	-13.8	96
bta-miR-95	-13.7	235
bta-miR-95	-13.4	519
bta-miR-95	-13.1	457
bta-miR-95	-12.7	465
bta-miR-95	-12.7	697
bta-miR-95	-12.3	126
bta-miR-95	-12.0	668
bta-miR-95	-11.8	51
bta-miR-95	-11.8	185
bta-miR-95	-11.8	370
bta-miR-95	-11.8	327
bta-miR-95	-10.9	320
bta-miR-95	-10.0	116
bta-miR-96	-25.3	653
bta-miR-96	-24.1	12
bta-miR-96	-21.9	407
bta-miR-96	-19.8	72
bta-miR-96	-18.9	539
bta-miR-96	-18.8	605
bta-miR-96	-17.8	224
bta-miR-96	-17.7	447
bta-miR-96	-17.6	518
bta-miR-96	-17.5	573
bta-miR-96	-17.2	108
bta-miR-96	-16.7	725
bta-miR-96	-16.5	344
bta-miR-96	-16.4	472
bta-miR-96	-16.1	371
bta-miR-96	-14.5	171
bta-miR-96	-14.2	488
bta-miR-96	-13.8	198
bta-miR-96	-13.6	243
bta-miR-96	-13.2	270
bta-miR-96	-12.1	687
bta-miR-96	-11.6	633
bta-miR-96	-11.5	51
bta-miR-96	-10.7	154
bta-miR-96	-10.2	428
bta-miR-9	-22.6	12
bta-miR-9	-20.4	81
bta-miR-9	-20.2	555

bta-miR-9	-19.2	106
bta-miR-9	-19.2	222
bta-miR-9	-18.9	414
bta-miR-9	-17.8	454
bta-miR-9	-17.5	170
bta-miR-9	-17.4	621
bta-miR-9	-17.1	493
bta-miR-9	-16.5	473
bta-miR-9	-15.7	584
bta-miR-9	-15.0	264
bta-miR-9	-15.0	354
bta-miR-9	-14.7	37
bta-miR-9	-14.5	150
bta-miR-9	-13.6	733
bta-miR-9	-13.6	64
bta-miR-9	-13.5	654
bta-miR-9	-13.0	122
bta-miR-9	-12.2	379
bta-miR-9	-12.2	340
bta-miR-9	-12.2	602
bta-miR-9	-12.1	705
bta-miR-9	-11.6	517
bta-miR-9	-10.9	300
bta-miR-9*	-18.0	176
bta-miR-9*	-14.3	657
bta-miR-9*	-14.2	2
bta-miR-9*	-13.7	506
bta-miR-9*	-13.6	546
bta-miR-9*	-13.4	449
bta-miR-9*	-13.3	614
bta-miR-9*	-12.7	69
bta-miR-9*	-12.6	576
bta-miR-9*	-12.3	463
bta-miR-9*	-12.0	632
bta-miR-9*	-11.9	742
bta-miR-9*	-11.7	41
bta-miR-9*	-11.6	391
bta-miR-9*	-11.5	269
bta-miR-9*	-11.2	418
bta-miR-9*	-10.7	530
bta-miR-9*	-10.6	597
bta-miR-9*	-10.2	704
bta-miR-9*	-10.2	692
bta-miR-9*	-10.0	81
bta-miR-489	-20.0	8
bta-miR-489	-18.1	654
bta-miR-489	-17.9	562
bta-miR-489	-17.8	537
bta-miR-489	-17.3	86
bta-miR-489	-16.2	519
bta-miR-489	-15.2	360
bta-miR-489	-15.1	244
bta-miR-489	-14.6	624
bta-miR-489	-14.3	408
bta-miR-489	-14.2	109
bta-miR-489	-13.9	696
bta-miR-489	-13.7	51
bta-miR-489	-12.8	743
bta-miR-489	-12.6	140

bta-miR-489	-12.5	473
bta-miR-489	-12.3	425
bta-miR-489	-11.8	126
bta-miR-489	-11.4	73
bta-miR-489	-11.1	283
bta-miR-489	-11.0	492
bta-miR-489	-10.9	342
bta-miR-489	-10.8	266
bta-miR-489	-10.8	307
bta-miR-489	-10.4	167
bta-miR-489	-10.3	230
bta-miR-1224	-26.6	467
bta-miR-1224	-24.8	132
bta-miR-1224	-24.2	426
bta-miR-1224	-24.0	317
bta-miR-1224	-22.8	236
bta-miR-1224	-22.6	502
bta-miR-1224	-22.2	86
bta-miR-1224	-21.9	161
bta-miR-1224	-21.3	209
bta-miR-1224	-21.1	579
bta-miR-1224	-20.7	396
bta-miR-1224	-20.5	30
bta-miR-1224	-20.1	107
bta-miR-1224	-19.0	623
bta-miR-1224	-18.8	276
bta-miR-1224	-17.6	561
bta-miR-1224	-17.0	342
bta-miR-1224	-16.1	5
bta-miR-1224	-15.9	693
bta-miR-1224	-15.5	58
bta-miR-1224	-14.7	380
bta-miR-1224	-13.2	410
bta-miR-1224	-12.9	308
bta-miR-1224	-12.7	457
bta-miR-1224	-12.3	533
bta-miR-1224	-12.1	650
bta-miR-1224	-11.1	670
bta-miR-1224	-10.3	368
bta-miR-376b	-19.4	709
bta-miR-376b	-18.7	198
bta-miR-376b	-17.0	81
bta-miR-376b	-16.9	615
bta-miR-376b	-16.6	12
bta-miR-376b	-16.6	227
bta-miR-376b	-16.2	464
bta-miR-376b	-16.0	352
bta-miR-376b	-15.7	573
bta-miR-376b	-15.6	661
bta-miR-376b	-15.3	266
bta-miR-376b	-15.0	419
bta-miR-376b	-14.5	497
bta-miR-376b	-14.1	65
bta-miR-376b	-13.4	383
bta-miR-376b	-13.3	171
bta-miR-376b	-12.0	303
bta-miR-376b	-11.9	105
bta-miR-376b	-11.6	638
bta-miR-376b	-11.2	595

bta-miR-376b	-11.1	605
bta-miR-376b	-10.8	691
bta-miR-376b	-10.4	36
bta-miR-376b	-10.2	282
bta-miR-376b	-10.1	2
bta-miR-376b	-10.1	738
bta-miR-376b	-10.1	58
bta-miR-376b	-10.1	537
bta-miR-376d	-19.3	709
bta-miR-376d	-19.2	659
bta-miR-376d	-18.7	81
bta-miR-376d	-18.1	618
bta-miR-376d	-17.0	266
bta-miR-376d	-16.6	202
bta-miR-376d	-16.2	497
bta-miR-376d	-16.2	464
bta-miR-376d	-15.7	576
bta-miR-376d	-15.6	15
bta-miR-376d	-14.7	231
bta-miR-376d	-13.9	422
bta-miR-376d	-13.6	377
bta-miR-376d	-13.0	544
bta-miR-376d	-12.2	58
bta-miR-376d	-12.0	177
bta-miR-376d	-11.8	69
bta-miR-376d	-11.6	638
bta-miR-376d	-11.0	309
bta-miR-376d	-10.8	691
bta-miR-376d	-10.6	113
bta-miR-376d	-10.1	2
bta-miR-376d	-10.0	412
bta-miR-376c	-23.2	444
bta-miR-376c	-19.2	486
bta-miR-376c	-19.1	600
bta-miR-376c	-18.3	204
bta-miR-376c	-18.1	351
bta-miR-376c	-17.8	104
bta-miR-376c	-17.0	65
bta-miR-376c	-17.0	170
bta-miR-376c	-15.8	651
bta-miR-376c	-15.0	303
bta-miR-376c	-14.9	11
bta-miR-376c	-13.9	94
bta-miR-376c	-13.9	406
bta-miR-376c	-13.8	269
bta-miR-376c	-13.0	730
bta-miR-376c	-12.6	618
bta-miR-376c	-12.6	701
bta-miR-376c	-12.3	27
bta-miR-376c	-12.2	242
bta-miR-376c	-11.4	149
bta-miR-376c	-11.3	464
bta-miR-376c	-11.2	558
bta-miR-376c	-10.4	124
bta-miR-376c	-10.3	471
bta-miR-764	-29.7	444
bta-miR-764	-25.6	610
bta-miR-764	-24.6	366
bta-miR-764	-22.7	11

bta-miR-764	-20.4	224
bta-miR-764	-20.3	174
bta-miR-764	-20.2	348
bta-miR-764	-20.1	405
bta-miR-764	-19.6	69
bta-miR-764	-17.5	265
bta-miR-764	-17.1	26
bta-miR-764	-16.5	479
bta-miR-764	-16.1	101
bta-miR-764	-13.9	630
bta-miR-764	-13.8	574
bta-miR-764	-13.3	658
bta-miR-764	-12.9	433
bta-miR-764	-12.9	543
bta-miR-764	-12.3	153
bta-miR-764	-12.1	731
bta-miR-764	-12.0	37
bta-miR-764	-11.1	597
bta-miR-764	-10.5	312
bta-miR-764	-10.0	517
bta-miR-1225-3p	-35.8	609
bta-miR-1225-3p	-35.2	477
bta-miR-1225-3p	-34.6	69
bta-miR-1225-3p	-27.1	448
bta-miR-1225-3p	-27.0	266
bta-miR-1225-3p	-26.6	403
bta-miR-1225-3p	-22.9	12
bta-miR-1225-3p	-22.7	369
bta-miR-1225-3p	-19.8	112
bta-miR-1225-3p	-16.9	598
bta-miR-1225-3p	-15.8	155
bta-miR-1225-3p	-14.0	654
bta-miR-1225-3p	-14.0	216
bta-miR-1225-3p	-12.9	740
bta-miR-1225-3p	-12.7	560
bta-miR-1225-3p	-12.7	631
bta-miR-1225-3p	-12.6	199
bta-miR-1225-3p	-11.4	507
bta-miR-1225-3p	-10.7	427
bta-miR-29e	-21.8	65
bta-miR-29e	-18.9	366
bta-miR-29e	-16.9	13
bta-miR-29e	-16.4	735
bta-miR-29e	-15.8	558
bta-miR-29e	-15.3	204
bta-miR-29e	-15.3	652
bta-miR-29e	-15.3	403
bta-miR-29e	-15.0	92
bta-miR-29e	-15.0	429
bta-miR-29e	-14.6	604
bta-miR-29e	-14.0	471
bta-miR-29e	-14.0	159
bta-miR-29e	-13.8	264
bta-miR-29e	-13.4	535
bta-miR-29e	-13.2	339
bta-miR-29e	-13.0	711
bta-miR-29e	-12.6	35
bta-miR-29e	-12.1	499
bta-miR-29e	-11.8	628

bta-miR-29e	-11.8	685
bta-miR-29e	-10.2	238
bta-miR-29e	-10.2	299
bta-miR-1282	-24.8	604
bta-miR-1282	-22.2	197
bta-miR-1282	-20.2	434
bta-miR-1282	-20.0	264
bta-miR-1282	-19.5	65
bta-miR-1282	-18.5	471
bta-miR-1282	-18.1	414
bta-miR-1282	-16.1	622
bta-miR-1282	-16.0	11
bta-miR-1282	-15.8	100
bta-miR-1282	-15.2	171
bta-miR-1282	-14.1	494
bta-miR-1282	-14.0	153
bta-miR-1282	-13.7	558
bta-miR-1282	-13.3	652
bta-miR-1282	-12.9	349
bta-miR-1282	-10.7	29
bta-miR-1282	-10.6	124
bta-miR-1282	-10.5	238
bta-miR-1282	-10.0	737
bta-miR-1284	-27.7	405
bta-miR-1284	-26.7	47
bta-miR-1284	-23.8	614
bta-miR-1284	-23.1	11
bta-miR-1284	-23.0	572
bta-miR-1284	-22.6	470
bta-miR-1284	-22.3	171
bta-miR-1284	-22.1	102
bta-miR-1284	-21.0	70
bta-miR-1284	-19.2	506
bta-miR-1284	-18.9	441
bta-miR-1284	-18.7	596
bta-miR-1284	-17.1	557
bta-miR-1284	-16.7	203
bta-miR-1284	-16.7	240
bta-miR-1284	-16.7	361
bta-miR-1284	-15.7	652
bta-miR-1284	-14.1	28
bta-miR-1284	-13.7	225
bta-miR-1284	-13.6	279
bta-miR-1284	-13.3	424
bta-miR-1284	-12.4	541
bta-miR-1284	-12.1	4
bta-miR-1284	-12.1	735
bta-miR-1284	-11.3	153
bta-miR-1284	-10.9	338
bta-miR-1284	-10.0	386
bta-miR-376a	-19.3	709
bta-miR-376a	-18.7	81
bta-miR-376a	-18.1	618
bta-miR-376a	-17.7	660
bta-miR-376a	-17.0	266
bta-miR-376a	-16.6	202
bta-miR-376a	-16.2	497
bta-miR-376a	-16.2	464
bta-miR-376a	-16.2	13

bta-miR-376a	-15.7	576
bta-miR-376a	-14.7	231
bta-miR-376a	-14.5	172
bta-miR-376a	-14.5	420
bta-miR-376a	-12.3	367
bta-miR-376a	-12.2	58
bta-miR-376a	-11.8	69
bta-miR-376a	-11.7	396
bta-miR-376a	-11.6	638
bta-miR-376a	-11.5	545
bta-miR-376a	-11.0	309
bta-miR-376a	-10.8	691
bta-miR-376a	-10.6	113
bta-miR-376a	-10.1	2
bta-miR-376a	-10.0	412
bta-miR-1291	-30.8	66
bta-miR-1291	-29.3	602
bta-miR-1291	-26.2	451
bta-miR-1291	-25.2	250
bta-miR-1291	-23.9	560
bta-miR-1291	-23.4	387
bta-miR-1291	-21.3	6
bta-miR-1291	-21.3	492
bta-miR-1291	-20.7	92
bta-miR-1291	-18.7	275
bta-miR-1291	-18.5	628
bta-miR-1291	-18.1	38
bta-miR-1291	-17.9	418
bta-miR-1291	-17.3	216
bta-miR-1291	-17.2	172
bta-miR-1291	-14.9	146
bta-miR-1291	-14.6	685
bta-miR-1291	-14.2	529
bta-miR-1291	-13.8	427
bta-miR-1291	-12.9	364
bta-miR-1291	-12.3	118
bta-miR-1291	-10.8	738
bta-miR-1179	-23.2	164
bta-miR-1179	-20.9	340
bta-miR-1179	-19.8	603
bta-miR-1179	-19.6	407
bta-miR-1179	-18.9	568
bta-miR-1179	-18.8	364
bta-miR-1179	-18.0	511
bta-miR-1179	-17.4	468
bta-miR-1179	-17.3	426
bta-miR-1179	-16.0	723
bta-miR-1179	-15.5	261
bta-miR-1179	-15.3	52
bta-miR-1179	-15.2	107
bta-miR-1179	-15.0	210
bta-miR-1179	-14.7	450
bta-miR-1179	-14.4	5
bta-miR-1179	-14.0	650
bta-miR-1179	-13.1	229
bta-miR-1179	-12.1	495
bta-miR-1179	-12.1	136
bta-miR-1179	-11.8	120
bta-miR-1179	-11.6	92

bta-miR-1179	-11.6	699
bta-miR-1179	-11.0	29
bta-miR-1179	-10.7	630
bta-miR-1179	-10.6	188
bta-miR-1179	-10.6	670
bta-miR-1179	-10.1	325
bta-miR-1301	-26.9	54
bta-miR-1301	-24.6	102
bta-miR-1301	-24.1	177
bta-miR-1301	-24.0	624
bta-miR-1301	-23.9	4
bta-miR-1301	-22.7	475
bta-miR-1301	-22.6	394
bta-miR-1301	-20.8	258
bta-miR-1301	-20.5	137
bta-miR-1301	-18.7	429
bta-miR-1301	-18.1	350
bta-miR-1301	-18.0	574
bta-miR-1301	-17.7	537
bta-miR-1301	-17.7	589
bta-miR-1301	-17.3	502
bta-miR-1301	-15.6	208
bta-miR-1301	-14.2	731
bta-miR-1301	-13.8	30
bta-miR-1301	-13.0	613
bta-miR-1301	-12.2	690
bta-miR-1301	-12.1	458
bta-miR-1301	-12.1	236
bta-miR-1301	-11.9	657
bta-miR-1301	-11.3	131
bta-miR-1301	-10.6	315
bta-miR-1301	-10.5	90
bta-miR-1301	-10.4	385
bta-miR-1301	-10.0	291
bta-miR-1256	-24.5	235
bta-miR-1256	-23.5	344
bta-miR-1256	-22.1	436
bta-miR-1256	-22.1	391
bta-miR-1256	-20.3	215
bta-miR-1256	-19.9	501
bta-miR-1256	-19.8	14
bta-miR-1256	-19.8	608
bta-miR-1256	-18.8	566
bta-miR-1256	-18.6	476
bta-miR-1256	-17.8	267
bta-miR-1256	-17.7	60
bta-miR-1256	-17.2	174
bta-miR-1256	-16.8	628
bta-miR-1256	-16.3	369
bta-miR-1256	-14.7	650
bta-miR-1256	-14.2	112
bta-miR-1256	-14.1	198
bta-miR-1256	-14.0	418
bta-miR-1256	-12.7	734
bta-miR-1256	-11.4	1
bta-miR-1256	-10.9	539
bta-miR-1256	-10.9	681
bta-miR-1256	-10.5	51
bta-miR-1256	-10.4	94

bta-miR-1835	-30.8	243
bta-miR-1835	-29.8	463
bta-miR-1835	-29.7	617
bta-miR-1835	-28.0	29
bta-miR-1835	-26.3	165
bta-miR-1835	-24.4	407
bta-miR-1835	-24.0	4
bta-miR-1835	-23.4	107
bta-miR-1835	-22.2	559
bta-miR-1835	-22.0	361
bta-miR-1835	-21.0	513
bta-miR-1835	-19.9	205
bta-miR-1835	-19.6	596
bta-miR-1835	-18.7	389
bta-miR-1835	-18.1	431
bta-miR-1835	-17.3	128
bta-miR-1835	-16.4	80
bta-miR-1835	-16.1	282
bta-miR-1835	-15.4	650
bta-miR-1835	-15.4	496
bta-miR-1835	-14.2	326
bta-miR-1835	-14.2	532
bta-miR-1835	-14.1	181
bta-miR-1835	-12.3	691
bta-miR-1835	-12.2	234
bta-miR-1835	-11.5	737
bta-miR-1835	-11.1	98
bta-miR-1835	-10.8	321
bta-miR-1835	-10.6	276
bta-miR-1835	-10.5	715
bta-miR-1835	-10.3	155
bta-miR-1281	-29.3	609
bta-miR-1281	-25.5	443
bta-miR-1281	-20.3	220
bta-miR-1281	-19.8	69
bta-miR-1281	-16.2	411
bta-miR-1281	-15.9	479
bta-miR-1281	-15.8	348
bta-miR-1281	-15.0	266
bta-miR-1281	-15.0	598
bta-miR-1281	-14.5	630
bta-miR-1281	-14.1	12
bta-miR-1281	-12.6	154
bta-miR-1281	-12.4	496
bta-miR-1281	-11.7	737
bta-miR-1281	-10.8	434
bta-miR-1281	-10.7	202
bta-miR-1281	-10.7	371
bta-miR-1281	-10.6	114
bta-miR-1281	-10.1	37
bta-miR-1251	-26.4	408
bta-miR-1251	-21.9	473
bta-miR-1251	-21.9	608
bta-miR-1251	-20.4	70
bta-miR-1251	-20.3	20
bta-miR-1251	-19.8	108
bta-miR-1251	-19.1	358
bta-miR-1251	-18.6	171
bta-miR-1251	-18.2	425

bta-miR-1251	-17.4	258
bta-miR-1251	-17.0	3
bta-miR-1251	-16.7	205
bta-miR-1251	-16.4	559
bta-miR-1251	-15.7	517
bta-miR-1251	-15.7	50
bta-miR-1251	-15.2	741
bta-miR-1251	-14.6	653
bta-miR-1251	-14.0	448
bta-miR-1251	-12.9	379
bta-miR-1251	-11.9	243
bta-miR-1251	-11.7	697
bta-miR-1251	-11.4	307
bta-miR-1251	-10.8	155
bta-miR-1251	-10.8	343
bta-miR-1251	-10.3	596
bta-miR-1251	-10.3	544
bta-miR-1296	-33.1	609
bta-miR-1296	-33.0	220
bta-miR-1296	-28.8	414
bta-miR-1296	-28.0	449
bta-miR-1296	-27.5	69
bta-miR-1296	-23.6	266
bta-miR-1296	-23.4	350
bta-miR-1296	-21.4	480
bta-miR-1296	-21.3	101
bta-miR-1296	-19.8	17
bta-miR-1296	-19.4	171
bta-miR-1296	-19.3	125
bta-miR-1296	-19.2	37
bta-miR-1296	-16.3	1
bta-miR-1296	-16.1	570
bta-miR-1296	-16.0	515
bta-miR-1296	-15.5	371
bta-miR-1296	-15.5	661
bta-miR-1296	-15.0	404
bta-miR-1296	-14.4	201
bta-miR-1296	-14.4	589
bta-miR-1296	-13.7	629
bta-miR-1296	-13.6	737
bta-miR-1296	-13.2	559
bta-miR-1296	-12.0	252
bta-miR-1296	-11.8	325
bta-miR-1296	-11.4	308
bta-miR-1296	-11.2	154
bta-miR-1296	-10.9	709
bta-miR-1296	-10.7	439
bta-miR-1296	-10.6	119
bta-miR-1296	-10.2	698
bta-miR-1193	-27.3	8
bta-miR-1193	-22.4	617
bta-miR-1193	-20.8	407
bta-miR-1193	-19.8	63
bta-miR-1193	-19.6	435
bta-miR-1193	-19.1	464
bta-miR-1193	-17.8	263
bta-miR-1193	-17.6	496
bta-miR-1193	-16.9	566
bta-miR-1193	-15.9	349

bta-miR-1193	-15.8	35
bta-miR-1193	-15.5	94
bta-miR-1193	-15.1	166
bta-miR-1193	-14.6	224
bta-miR-1193	-14.5	737
bta-miR-1193	-14.4	598
bta-miR-1193	-14.0	371
bta-miR-1193	-12.4	653
bta-miR-1193	-12.2	123
bta-miR-1193	-11.8	532
bta-miR-1193	-11.4	393
bta-miR-1193	-10.9	704
bta-miR-1193	-10.7	332
bta-miR-1193	-10.2	26
bta-miR-1193	-10.0	584
bta-miR-1197	-21.8	79
bta-miR-1197	-20.5	16
bta-miR-1197	-20.3	371
bta-miR-1197	-19.5	219
bta-miR-1197	-19.3	566
bta-miR-1197	-18.4	496
bta-miR-1197	-18.4	418
bta-miR-1197	-18.3	614
bta-miR-1197	-17.8	348
bta-miR-1197	-17.3	450
bta-miR-1197	-15.3	239
bta-miR-1197	-15.2	517
bta-miR-1197	-14.8	653
bta-miR-1197	-14.1	34
bta-miR-1197	-14.1	198
bta-miR-1197	-13.7	539
bta-miR-1197	-13.7	481
bta-miR-1197	-13.5	265
bta-miR-1197	-13.3	1
bta-miR-1197	-13.3	101
bta-miR-1197	-13.2	405
bta-miR-1197	-12.7	731
bta-miR-1197	-12.5	174
bta-miR-1197	-12.5	51
bta-miR-1197	-12.2	126
bta-miR-1197	-12.2	709
bta-miR-1197	-11.5	279
bta-miR-1197	-11.3	154
bta-miR-1197	-10.9	305
bta-miR-1197	-10.7	65
bta-miR-1197	-10.1	598
bta-miR-1839	-23.6	374
bta-miR-1839	-21.2	573
bta-miR-1839	-21.2	546
bta-miR-1839	-19.5	278
bta-miR-1839	-19.5	108
bta-miR-1839	-19.2	410
bta-miR-1839	-18.4	736
bta-miR-1839	-17.1	1
bta-miR-1839	-17.0	480
bta-miR-1839	-16.9	635
bta-miR-1839	-16.7	70
bta-miR-1839	-16.3	350
bta-miR-1839	-15.3	240

bta-miR-1839	-15.2	651
bta-miR-1839	-15.1	455
bta-miR-1839	-15.0	517
bta-miR-1839	-14.5	176
bta-miR-1839	-14.4	202
bta-miR-1839	-13.8	16
bta-miR-1839	-13.1	684
bta-miR-1839	-12.6	49
bta-miR-1839	-12.4	308
bta-miR-1839	-12.1	152
bta-miR-1839	-11.9	702
bta-miR-1839	-10.9	621
bta-miR-1839	-10.5	123
bta-miR-1839	-10.1	35
bta-miR-1307	-29.0	416
bta-miR-1307	-27.2	1
bta-miR-1307	-24.5	112
bta-miR-1307	-22.3	172
bta-miR-1307	-22.1	569
bta-miR-1307	-20.1	352
bta-miR-1307	-20.0	590
bta-miR-1307	-19.6	457
bta-miR-1307	-19.2	39
bta-miR-1307	-18.9	660
bta-miR-1307	-18.4	83
bta-miR-1307	-18.3	305
bta-miR-1307	-18.3	543
bta-miR-1307	-18.0	229
bta-miR-1307	-17.8	155
bta-miR-1307	-17.4	482
bta-miR-1307	-16.8	130
bta-miR-1307	-16.4	403
bta-miR-1307	-16.2	20
bta-miR-1307	-16.2	738
bta-miR-1307	-16.1	628
bta-miR-1307	-15.8	504
bta-miR-1307	-15.3	256
bta-miR-1307	-13.8	204
bta-miR-1307	-13.6	326
bta-miR-1307	-13.6	372
bta-miR-1307	-13.2	611
bta-miR-1307	-11.5	438
bta-miR-1307	-10.2	693
bta-miR-1298	-23.9	106
bta-miR-1298	-23.0	415
bta-miR-1298	-22.6	4
bta-miR-1298	-22.3	473
bta-miR-1298	-19.6	66
bta-miR-1298	-19.5	383
bta-miR-1298	-19.2	149
bta-miR-1298	-18.9	206
bta-miR-1298	-18.9	551
bta-miR-1298	-18.1	705
bta-miR-1298	-17.8	654
bta-miR-1298	-17.7	606
bta-miR-1298	-17.5	369
bta-miR-1298	-17.1	21
bta-miR-1298	-16.8	343
bta-miR-1298	-16.7	575

bta-miR-1298	-15.3	235
bta-miR-1298	-15.0	39
bta-miR-1298	-14.9	740
bta-miR-1298	-14.6	256
bta-miR-1298	-13.9	183
bta-miR-1298	-13.4	441
bta-miR-1298	-12.9	271
bta-miR-1298	-12.5	454
bta-miR-1298	-12.0	297
bta-miR-1298	-11.8	125
bta-miR-1298	-11.5	407
bta-miR-1298	-10.6	517
bta-miR-1287	-22.7	42
bta-miR-1287	-22.6	466
bta-miR-1287	-22.2	403
bta-miR-1287	-20.4	72
bta-miR-1287	-20.4	115
bta-miR-1287	-20.4	155
bta-miR-1287	-19.9	500
bta-miR-1287	-19.8	130
bta-miR-1287	-19.4	570
bta-miR-1287	-19.1	352
bta-miR-1287	-18.8	9
bta-miR-1287	-17.3	310
bta-miR-1287	-17.1	377
bta-miR-1287	-16.5	623
bta-miR-1287	-15.8	233
bta-miR-1287	-15.8	424
bta-miR-1287	-15.0	275
bta-miR-1287	-14.8	520
bta-miR-1287	-13.9	731
bta-miR-1287	-13.7	693
bta-miR-1287	-13.2	659
bta-miR-1287	-12.8	452
bta-miR-1287	-11.6	337
bta-miR-1287	-11.4	28
bta-miR-1287	-11.1	207
bta-miR-1287	-10.8	102
bta-miR-1287	-10.3	558
bta-miR-1248	-26.7	12
bta-miR-1248	-26.2	64
bta-miR-1248	-25.1	575
bta-miR-1248	-24.2	419
bta-miR-1248	-18.5	487
bta-miR-1248	-18.5	198
bta-miR-1248	-17.6	351
bta-miR-1248	-17.5	653
bta-miR-1248	-17.1	516
bta-miR-1248	-17.1	101
bta-miR-1248	-16.5	407
bta-miR-1248	-15.9	155
bta-miR-1248	-15.5	456
bta-miR-1248	-15.3	258
bta-miR-1248	-13.8	181
bta-miR-1248	-12.9	381
bta-miR-1248	-12.7	282
bta-miR-1248	-12.7	623
bta-miR-1248	-12.7	737
bta-miR-1248	-11.5	550

bta-miR-1248	-10.9	234
bta-miR-1248	-10.9	687
bta-miR-1248	-10.5	559
bta-miR-1185	-21.6	199
bta-miR-1185	-21.5	601
bta-miR-1185	-21.4	66
bta-miR-1185	-18.4	630
bta-miR-1185	-17.2	445
bta-miR-1185	-16.8	10
bta-miR-1185	-16.8	487
bta-miR-1185	-16.3	270
bta-miR-1185	-16.0	172
bta-miR-1185	-14.6	571
bta-miR-1185	-14.6	105
bta-miR-1185	-14.6	414
bta-miR-1185	-14.6	701
bta-miR-1185	-14.4	464
bta-miR-1185	-14.1	29
bta-miR-1185	-13.8	224
bta-miR-1185	-13.8	518
bta-miR-1185	-13.3	352
bta-miR-1185	-13.1	653
bta-miR-1185	-12.5	303
bta-miR-1185	-12.4	479
bta-miR-1185	-12.3	372
bta-miR-1185	-12.0	238
bta-miR-1185	-11.5	257
bta-miR-1185	-11.3	149
bta-miR-1185	-10.9	733
bta-miR-1185	-10.8	96
bta-miR-1185	-10.0	395
bta-miR-1306	-31.3	599
bta-miR-1306	-25.0	66
bta-miR-1306	-24.0	423
bta-miR-1306	-22.9	481
bta-miR-1306	-20.9	3
bta-miR-1306	-18.0	621
bta-miR-1306	-15.6	266
bta-miR-1306	-15.5	172
bta-miR-1306	-13.8	371
bta-miR-1306	-13.7	217
bta-miR-1306	-13.2	343
bta-miR-1306	-13.1	24
bta-miR-1306	-13.0	199
bta-miR-1306	-12.7	415
bta-miR-1306	-12.7	550
bta-miR-1306	-12.2	635
bta-miR-1306	-12.0	565
bta-miR-1306	-11.7	94
bta-miR-1306	-11.5	738
bta-miR-1306	-11.1	695
bta-miR-1306	-10.0	507
bta-miR-1271	-22.8	411
bta-miR-1271	-22.4	473
bta-miR-1271	-21.1	545
bta-miR-1271	-20.9	1
bta-miR-1271	-20.7	356
bta-miR-1271	-20.0	102
bta-miR-1271	-19.4	69

bta-miR-1271	-18.9	616
bta-miR-1271	-18.9	730
bta-miR-1271	-17.9	584
bta-miR-1271	-17.3	654
bta-miR-1271	-17.0	517
bta-miR-1271	-17.0	452
bta-miR-1271	-16.7	41
bta-miR-1271	-16.3	267
bta-miR-1271	-14.5	217
bta-miR-1271	-14.4	493
bta-miR-1271	-13.6	702
bta-miR-1271	-12.2	126
bta-miR-1271	-12.2	174
bta-miR-1271	-12.2	240
bta-miR-1271	-12.0	569
bta-miR-1271	-11.5	51
bta-miR-1271	-11.0	438
bta-miR-1271	-10.8	307
bta-miR-1271	-10.1	145
bta-miR-1249	-38.6	611
bta-miR-1249	-30.0	443
bta-miR-1249	-26.6	62
bta-miR-1249	-25.0	404
bta-miR-1249	-20.7	479
bta-miR-1249	-18.9	588
bta-miR-1249	-17.4	12
bta-miR-1249	-17.3	265
bta-miR-1249	-14.4	93
bta-miR-1249	-14.0	220
bta-miR-1249	-13.2	171
bta-miR-1249	-10.6	371
bta-miR-1249	-10.3	41
bta-miR-2284i	-19.7	620
bta-miR-2284i	-19.7	269
bta-miR-2284i	-18.8	159
bta-miR-2284i	-18.5	592
bta-miR-2284i	-18.0	61
bta-miR-2284i	-17.9	408
bta-miR-2284i	-17.2	232
bta-miR-2284i	-16.7	197
bta-miR-2284i	-16.5	94
bta-miR-2284i	-16.0	508
bta-miR-2284i	-15.6	450
bta-miR-2284i	-15.5	558
bta-miR-2284i	-15.1	22
bta-miR-2284i	-14.8	126
bta-miR-2284i	-14.4	306
bta-miR-2284i	-14.2	486
bta-miR-2284i	-14.1	351
bta-miR-2284i	-13.6	383
bta-miR-2284i	-13.5	702
bta-miR-2284i	-13.2	652
bta-miR-2284i	-12.9	326
bta-miR-2284i	-12.2	9
bta-miR-2284i	-12.0	462
bta-miR-2284i	-11.7	735
bta-miR-2284i	-11.5	81
bta-miR-2284i	-11.2	51
bta-miR-2284i	-10.6	429

bta-miR-2284i	-10.5	610
bta-miR-2286	-26.3	381
bta-miR-2286	-22.6	81
bta-miR-2286	-22.3	420
bta-miR-2286	-21.5	654
bta-miR-2286	-20.4	149
bta-miR-2286	-20.3	457
bta-miR-2286	-20.0	343
bta-miR-2286	-19.5	535
bta-miR-2286	-18.7	1
bta-miR-2286	-18.5	306
bta-miR-2286	-18.4	35
bta-miR-2286	-18.4	106
bta-miR-2286	-17.6	563
bta-miR-2286	-17.3	235
bta-miR-2286	-16.9	488
bta-miR-2286	-16.7	22
bta-miR-2286	-15.3	199
bta-miR-2286	-15.2	256
bta-miR-2286	-14.3	690
bta-miR-2286	-13.3	616
bta-miR-2286	-12.9	328
bta-miR-2286	-11.5	740
bta-miR-2286	-11.4	137
bta-miR-2286	-11.2	61
bta-miR-2286	-10.8	368
bta-miR-2286	-10.8	287
bta-miR-2286	-10.4	181
bta-miR-2286	-10.3	596
bta-miR-2286	-10.1	483
bta-miR-2287	-29.6	114
bta-miR-2287	-27.8	450
bta-miR-2287	-27.6	584
bta-miR-2287	-27.1	375
bta-miR-2287	-26.1	153
bta-miR-2287	-26.0	560
bta-miR-2287	-24.1	79
bta-miR-2287	-23.2	41
bta-miR-2287	-22.1	411
bta-miR-2287	-21.7	20
bta-miR-2287	-20.8	244
bta-miR-2287	-19.9	307
bta-miR-2287	-18.8	357
bta-miR-2287	-18.5	546
bta-miR-2287	-18.4	480
bta-miR-2287	-17.6	265
bta-miR-2287	-16.5	136
bta-miR-2287	-16.3	743
bta-miR-2287	-16.3	659
bta-miR-2287	-16.2	3
bta-miR-2287	-15.7	185
bta-miR-2287	-15.4	337
bta-miR-2287	-15.2	227
bta-miR-2287	-14.5	202
bta-miR-2287	-14.2	616
bta-miR-2287	-13.4	280
bta-miR-2287	-13.4	696
bta-miR-2287	-13.3	518
bta-miR-2287	-13.3	395

bta-miR-2287	-12.6	327
bta-miR-2287	-12.5	631
bta-miR-2287	-12.2	107
bta-miR-2287	-11.1	62
bta-miR-2287	-10.5	473
bta-miR-2288	-22.3	119
bta-miR-2288	-20.5	406
bta-miR-2288	-20.5	481
bta-miR-2288	-19.9	376
bta-miR-2288	-19.2	563
bta-miR-2288	-18.3	49
bta-miR-2288	-18.3	361
bta-miR-2288	-17.9	143
bta-miR-2288	-17.8	648
bta-miR-2288	-15.8	508
bta-miR-2288	-15.5	318
bta-miR-2288	-14.9	233
bta-miR-2288	-14.7	166
bta-miR-2288	-14.6	468
bta-miR-2288	-14.4	197
bta-miR-2288	-14.2	91
bta-miR-2288	-14.0	731
bta-miR-2288	-13.9	584
bta-miR-2288	-13.5	11
bta-miR-2288	-13.4	298
bta-miR-2288	-13.0	698
bta-miR-2288	-11.3	455
bta-miR-2288	-10.8	273
bta-miR-2289	-22.9	617
bta-miR-2289	-20.8	412
bta-miR-2289	-19.9	352
bta-miR-2289	-17.7	448
bta-miR-2289	-16.8	24
bta-miR-2289	-16.6	478
bta-miR-2289	-16.3	229
bta-miR-2289	-15.9	172
bta-miR-2289	-15.9	589
bta-miR-2289	-15.7	306
bta-miR-2289	-15.5	69
bta-miR-2289	-14.8	114
bta-miR-2289	-14.7	269
bta-miR-2289	-13.7	155
bta-miR-2289	-13.6	428
bta-miR-2289	-13.3	545
bta-miR-2289	-12.8	497
bta-miR-2289	-12.4	377
bta-miR-2289	-12.3	1
bta-miR-2289	-11.5	658
bta-miR-2289	-11.1	697
bta-miR-2289	-11.0	42
bta-miR-2289	-10.8	570
bta-miR-2289	-10.7	344
bta-miR-2289	-10.6	738
bta-miR-2289	-10.3	524
bta-miR-2285a	-16.2	596
bta-miR-2285a	-15.5	484
bta-miR-2285a	-15.1	277
bta-miR-2285a	-13.2	169
bta-miR-2285a	-13.1	650

bta-miR-2285a	-13.0	34
bta-miR-2285a	-12.9	452
bta-miR-2285a	-11.7	67
bta-miR-2285a	-11.7	237
bta-miR-2285a	-11.7	572
bta-miR-2285a	-11.4	374
bta-miR-2285a	-11.2	505
bta-miR-2285a	-10.9	136
bta-miR-2285a	-10.8	1
bta-miR-2285a	-10.7	710
bta-miR-2285a	-10.5	309
bta-miR-2285a	-10.5	348
bta-miR-2285a	-10.1	670
bta-miR-2290	-24.7	49
bta-miR-2290	-24.2	586
bta-miR-2290	-23.6	457
bta-miR-2290	-23.5	184
bta-miR-2290	-23.5	403
bta-miR-2290	-22.5	122
bta-miR-2290	-22.2	84
bta-miR-2290	-21.5	228
bta-miR-2290	-21.3	560
bta-miR-2290	-20.2	10
bta-miR-2290	-20.0	417
bta-miR-2290	-19.1	485
bta-miR-2290	-18.4	257
bta-miR-2290	-17.8	160
bta-miR-2290	-17.7	353
bta-miR-2290	-17.5	737
bta-miR-2290	-16.8	508
bta-miR-2290	-16.6	623
bta-miR-2290	-16.1	320
bta-miR-2290	-15.0	332
bta-miR-2290	-14.8	30
bta-miR-2290	-13.9	611
bta-miR-2290	-13.7	654
bta-miR-2290	-13.6	272
bta-miR-2290	-13.3	210
bta-miR-2290	-10.8	383
bta-miR-2290	-10.8	451
bta-miR-2290	-10.6	698
bta-miR-2290	-10.4	523
bta-miR-2290	-10.2	634
bta-miR-2291	-28.7	30
bta-miR-2291	-28.1	244
bta-miR-2291	-27.6	387
bta-miR-2291	-26.0	109
bta-miR-2291	-24.9	427
bta-miR-2291	-24.4	167
bta-miR-2291	-24.3	513
bta-miR-2291	-23.5	473
bta-miR-2291	-22.8	360
bta-miR-2291	-21.0	132
bta-miR-2291	-20.0	3
bta-miR-2291	-20.0	624
bta-miR-2291	-19.7	561
bta-miR-2291	-18.7	283
bta-miR-2291	-17.9	73
bta-miR-2291	-17.2	740

bta-miR-2291	-16.5	686
bta-miR-2291	-15.3	538
bta-miR-2291	-15.2	591
bta-miR-2291	-15.0	329
bta-miR-2291	-13.2	92
bta-miR-2291	-12.8	323
bta-miR-2291	-12.2	657
bta-miR-2291	-11.9	206
bta-miR-2291	-11.6	236
bta-miR-2291	-11.4	276
bta-miR-2291	-11.0	413
bta-miR-2292	-23.1	109
bta-miR-2292	-22.7	257
bta-miR-2292	-21.8	185
bta-miR-2292	-21.4	86
bta-miR-2292	-20.8	562
bta-miR-2292	-20.4	30
bta-miR-2292	-20.4	425
bta-miR-2292	-20.0	3
bta-miR-2292	-19.9	395
bta-miR-2292	-18.6	511
bta-miR-2292	-18.5	457
bta-miR-2292	-18.1	697
bta-miR-2292	-18.1	51
bta-miR-2292	-17.7	360
bta-miR-2292	-16.2	488
bta-miR-2292	-16.1	664
bta-miR-2292	-16.0	136
bta-miR-2292	-14.8	307
bta-miR-2292	-14.4	624
bta-miR-2292	-13.9	244
bta-miR-2292	-13.0	213
bta-miR-2292	-12.8	596
bta-miR-2292	-12.6	530
bta-miR-2292	-12.6	325
bta-miR-2292	-12.6	234
bta-miR-2292	-11.7	164
bta-miR-2292	-11.6	640
bta-miR-2292	-11.1	380
bta-miR-2292	-10.7	286
bta-miR-2292	-10.3	483
bta-miR-2293	-14.4	592
bta-miR-2293	-13.8	483
bta-miR-2293	-13.3	167
bta-miR-2293	-12.7	20
bta-miR-2293	-12.6	264
bta-miR-2293	-12.3	358
bta-miR-2293	-12.3	101
bta-miR-2293	-12.1	151
bta-miR-2293	-12.0	222
bta-miR-2293	-11.7	727
bta-miR-2293	-11.6	199
bta-miR-2293	-11.0	556
bta-miR-2293	-10.3	435
bta-miR-2294	-27.0	85
bta-miR-2294	-26.9	559
bta-miR-2294	-25.4	265
bta-miR-2294	-25.2	617
bta-miR-2294	-24.4	472

bta-miR-2294	-23.5	376
bta-miR-2294	-22.2	37
bta-miR-2294	-22.2	65
bta-miR-2294	-22.1	407
bta-miR-2294	-21.6	198
bta-miR-2294	-21.6	586
bta-miR-2294	-21.0	12
bta-miR-2294	-20.4	115
bta-miR-2294	-20.1	506
bta-miR-2294	-19.5	222
bta-miR-2294	-18.7	351
bta-miR-2294	-17.6	653
bta-miR-2294	-17.6	155
bta-miR-2294	-17.0	428
bta-miR-2294	-16.6	293
bta-miR-2294	-16.5	448
bta-miR-2294	-15.4	128
bta-miR-2294	-14.2	687
bta-miR-2294	-14.0	252
bta-miR-2294	-13.9	710
bta-miR-2294	-13.9	184
bta-miR-2294	-13.0	525
bta-miR-2294	-11.9	321
bta-miR-2294	-11.5	544
bta-miR-2294	-10.6	742
bta-miR-2294	-10.2	58
bta-miR-2294	-10.1	1
bta-miR-2295	-29.1	308
bta-miR-2295	-27.3	418
bta-miR-2295	-26.9	125
bta-miR-2295	-26.8	454
bta-miR-2295	-26.5	83
bta-miR-2295	-25.7	507
bta-miR-2295	-23.5	203
bta-miR-2295	-22.2	241
bta-miR-2295	-22.2	570
bta-miR-2295	-21.5	279
bta-miR-2295	-21.0	155
bta-miR-2295	-20.8	385
bta-miR-2295	-19.5	349
bta-miR-2295	-19.2	177
bta-miR-2295	-17.8	28
bta-miR-2295	-16.1	50
bta-miR-2295	-16.0	710
bta-miR-2295	-15.6	630
bta-miR-2295	-14.5	691
bta-miR-2295	-14.4	480
bta-miR-2295	-13.4	672
bta-miR-2295	-13.4	1
bta-miR-2295	-12.6	70
bta-miR-2295	-12.2	117
bta-miR-2295	-11.2	738
bta-miR-2295	-10.8	550
bta-miR-2295	-10.3	331
bta-miR-2295	-10.0	224
bta-miR-2295	-10.0	594
bta-miR-2296	-28.4	65
bta-miR-2296	-25.0	450
bta-miR-2296	-24.9	494

bta-miR-2296	-24.6	278
bta-miR-2296	-22.2	617
bta-miR-2296	-22.0	22
bta-miR-2296	-21.8	419
bta-miR-2296	-21.6	198
bta-miR-2296	-20.7	171
bta-miR-2296	-19.3	472
bta-miR-2296	-19.1	597
bta-miR-2296	-18.9	239
bta-miR-2296	-17.2	107
bta-miR-2296	-16.6	261
bta-miR-2296	-16.4	308
bta-miR-2296	-15.9	575
bta-miR-2296	-15.7	361
bta-miR-2296	-15.1	653
bta-miR-2296	-13.8	738
bta-miR-2296	-13.5	543
bta-miR-2296	-12.9	8
bta-miR-2296	-12.8	559
bta-miR-2296	-12.7	405
bta-miR-2296	-12.6	517
bta-miR-2296	-12.6	154
bta-miR-2296	-12.4	125
bta-miR-2296	-12.3	710
bta-miR-2296	-11.0	50
bta-miR-2296	-10.8	383
bta-miR-2296	-10.5	639
bta-miR-2296	-10.4	609
bta-miR-2296	-10.0	687
bta-miR-2297	-23.2	598
bta-miR-2297	-20.9	219
bta-miR-2297	-20.4	434
bta-miR-2297	-16.7	347
bta-miR-2297	-14.2	176
bta-miR-2297	-14.2	620
bta-miR-2297	-14.0	499
bta-miR-2297	-12.7	69
bta-miR-2297	-11.8	16
bta-miR-2297	-11.7	265
bta-miR-2297	-11.3	731
bta-miR-2297	-10.5	450
bta-miR-2298	-16.8	493
bta-miR-2298	-16.6	606
bta-miR-2298	-16.4	360
bta-miR-2298	-16.3	199
bta-miR-2298	-15.5	66
bta-miR-2298	-15.1	25
bta-miR-2298	-15.1	271
bta-miR-2298	-14.9	644
bta-miR-2298	-14.4	554
bta-miR-2298	-14.4	156
bta-miR-2298	-13.3	585
bta-miR-2298	-13.2	450
bta-miR-2298	-13.0	100
bta-miR-2298	-12.7	116
bta-miR-2298	-12.2	731
bta-miR-2298	-12.0	220
bta-miR-2298	-11.7	238
bta-miR-2298	-11.7	478

bta-miR-2298	-11.3	4
bta-miR-2298	-10.9	531
bta-miR-2298	-10.2	344
bta-miR-2298	-10.1	415
bta-miR-2298	-10.0	46
bta-miR-2299-5p	-26.6	222
bta-miR-2299-5p	-21.4	442
bta-miR-2299-5p	-20.0	345
bta-miR-2299-5p	-19.6	495
bta-miR-2299-5p	-19.4	67
bta-miR-2299-5p	-18.9	607
bta-miR-2299-5p	-17.2	170
bta-miR-2299-5p	-16.9	200
bta-miR-2299-5p	-15.7	390
bta-miR-2299-5p	-14.9	263
bta-miR-2299-5p	-14.5	583
bta-miR-2299-5p	-13.9	626
bta-miR-2299-5p	-13.8	9
bta-miR-2299-5p	-13.4	720
bta-miR-2299-5p	-12.7	107
bta-miR-2299-5p	-11.3	476
bta-miR-2299-5p	-11.3	371
bta-miR-2299-5p	-11.1	241
bta-miR-2299-5p	-11.0	313
bta-miR-2299-5p	-10.9	334
bta-miR-2299-5p	-10.5	657
bta-miR-2299-5p	-10.4	187
bta-miR-2299-3p	-27.3	420
bta-miR-2299-3p	-26.3	86
bta-miR-2299-3p	-25.0	13
bta-miR-2299-3p	-24.0	457
bta-miR-2299-3p	-23.8	116
bta-miR-2299-3p	-23.4	231
bta-miR-2299-3p	-21.5	306
bta-miR-2299-3p	-20.0	493
bta-miR-2299-3p	-19.8	585
bta-miR-2299-3p	-19.7	367
bta-miR-2299-3p	-19.6	199
bta-miR-2299-3p	-19.5	560
bta-miR-2299-3p	-18.0	38
bta-miR-2299-3p	-17.9	617
bta-miR-2299-3p	-17.9	156
bta-miR-2299-3p	-17.1	343
bta-miR-2299-3p	-17.0	708
bta-miR-2299-3p	-16.3	256
bta-miR-2299-3p	-16.0	137
bta-miR-2299-3p	-14.5	514
bta-miR-2299-3p	-14.4	185
bta-miR-2299-3p	-13.7	658
bta-miR-2299-3p	-13.3	329
bta-miR-2299-3p	-13.3	738
bta-miR-2299-3p	-12.1	283
bta-miR-2299-3p	-11.7	481
bta-miR-2299-3p	-11.4	538
bta-miR-2299-3p	-11.1	606
bta-miR-2299-3p	-10.2	436
bta-miR-2299-3p	-10.1	686
bta-miR-2300a-5p	-26.9	614
bta-miR-2300a-5p	-25.5	448

bta-miR-2300a-5p	-22.5	418
bta-miR-2300a-5p	-21.5	176
bta-miR-2300a-5p	-19.8	69
bta-miR-2300a-5p	-19.5	350
bta-miR-2300a-5p	-18.0	731
bta-miR-2300a-5p	-17.3	268
bta-miR-2300a-5p	-17.1	598
bta-miR-2300a-5p	-16.4	16
bta-miR-2300a-5p	-15.8	570
bta-miR-2300a-5p	-15.7	232
bta-miR-2300a-5p	-13.9	101
bta-miR-2300a-5p	-13.6	483
bta-miR-2300a-5p	-13.1	633
bta-miR-2300a-5p	-12.0	373
bta-miR-2300a-5p	-11.9	506
bta-miR-2300a-5p	-11.8	129
bta-miR-2300a-5p	-11.5	556
bta-miR-2300a-5p	-10.9	383
bta-miR-2300a-5p	-10.6	1
bta-miR-2300a-5p	-10.6	50
bta-miR-2300a-5p	-10.4	698
bta-miR-2300b-3p	-22.3	111
bta-miR-2300b-3p	-22.1	401
bta-miR-2300b-3p	-20.7	261
bta-miR-2300b-3p	-20.5	168
bta-miR-2300b-3p	-20.3	5
bta-miR-2300b-3p	-20.1	559
bta-miR-2300b-3p	-18.7	692
bta-miR-2300b-3p	-17.3	511
bta-miR-2300b-3p	-17.2	33
bta-miR-2300b-3p	-16.9	58
bta-miR-2300b-3p	-16.2	209
bta-miR-2300b-3p	-16.1	650
bta-miR-2300b-3p	-15.9	282
bta-miR-2300b-3p	-15.8	596
bta-miR-2300b-3p	-15.2	363
bta-miR-2300b-3p	-14.7	467
bta-miR-2300b-3p	-13.8	309
bta-miR-2300b-3p	-13.5	540
bta-miR-2300b-3p	-13.5	139
bta-miR-2300b-3p	-13.0	737
bta-miR-2300b-3p	-12.7	456
bta-miR-2300b-3p	-12.3	155
bta-miR-2300b-3p	-12.1	629
bta-miR-2300b-3p	-11.8	491
bta-miR-2300b-3p	-11.0	248
bta-miR-2300b-3p	-11.0	395
bta-miR-2300b-3p	-10.9	432
bta-miR-2300b-3p	-10.4	583
bta-miR-2284s	-26.6	460
bta-miR-2284s	-24.2	60
bta-miR-2284s	-23.8	401
bta-miR-2284s	-22.2	97
bta-miR-2284s	-21.4	596
bta-miR-2284s	-21.2	258
bta-miR-2284s	-21.1	4
bta-miR-2284s	-18.7	24
bta-miR-2284s	-18.2	215
bta-miR-2284s	-17.6	566

bta-miR-2284s	-17.2	369
bta-miR-2284s	-16.5	622
bta-miR-2284s	-16.1	650
bta-miR-2284s	-15.8	730
bta-miR-2284s	-15.5	342
bta-miR-2284s	-15.4	173
bta-miR-2284s	-15.3	692
bta-miR-2284s	-14.5	140
bta-miR-2284s	-14.2	490
bta-miR-2284s	-12.2	432
bta-miR-2284s	-11.9	120
bta-miR-2284s	-11.5	311
bta-miR-2284s	-11.2	551
bta-miR-2284s	-11.1	191
bta-miR-2284s	-11.0	204
bta-miR-2284s	-10.9	635
bta-miR-2284s	-10.4	51
bta-miR-2301	-26.8	446
bta-miR-2301	-22.4	114
bta-miR-2301	-21.5	174
bta-miR-2301	-20.9	227
bta-miR-2301	-19.6	614
bta-miR-2301	-19.1	499
bta-miR-2301	-18.2	265
bta-miR-2301	-18.0	68
bta-miR-2301	-18.0	479
bta-miR-2301	-17.9	348
bta-miR-2301	-17.7	36
bta-miR-2301	-17.3	11
bta-miR-2301	-16.8	418
bta-miR-2301	-15.1	563
bta-miR-2301	-14.4	403
bta-miR-2301	-14.3	598
bta-miR-2301	-13.3	371
bta-miR-2301	-13.0	154
bta-miR-2301	-13.0	313
bta-miR-2301	-12.7	338
bta-miR-2301	-12.7	736
bta-miR-2301	-12.4	659
bta-miR-2301	-12.0	94
bta-miR-2301	-11.9	218
bta-miR-2301	-11.8	129
bta-miR-2301	-11.5	143
bta-miR-2301	-11.5	197
bta-miR-2301	-10.0	575
bta-miR-2302	-29.8	477
bta-miR-2302	-29.6	82
bta-miR-2302	-29.0	416
bta-miR-2302	-28.2	448
bta-miR-2302	-25.3	231
bta-miR-2302	-25.1	173
bta-miR-2302	-24.0	14
bta-miR-2302	-23.8	369
bta-miR-2302	-23.6	576
bta-miR-2302	-22.7	621
bta-miR-2302	-22.5	267
bta-miR-2302	-21.4	155
bta-miR-2302	-21.1	497
bta-miR-2302	-21.0	547

bta-miR-2302	-19.5	40
bta-miR-2302	-17.6	307
bta-miR-2302	-17.5	127
bta-miR-2302	-17.1	660
bta-miR-2302	-17.1	395
bta-miR-2302	-15.6	322
bta-miR-2302	-15.4	597
bta-miR-2302	-15.3	61
bta-miR-2302	-14.8	356
bta-miR-2302	-14.6	337
bta-miR-2302	-14.6	683
bta-miR-2302	-14.2	615
bta-miR-2302	-13.0	523
bta-miR-2302	-12.7	740
bta-miR-2302	-12.0	251
bta-miR-2302	-11.9	641
bta-miR-2302	-11.6	703
bta-miR-2302	-10.4	2
bta-miR-2302	-10.2	539
bta-miR-2303	-17.8	263
bta-miR-2303	-15.8	182
bta-miR-2303	-15.6	495
bta-miR-2303	-15.1	4
bta-miR-2303	-15.1	44
bta-miR-2303	-14.9	87
bta-miR-2303	-14.1	376
bta-miR-2303	-13.4	597
bta-miR-2303	-13.2	433
bta-miR-2303	-12.4	692
bta-miR-2303	-12.3	206
bta-miR-2303	-12.3	544
bta-miR-2303	-12.1	343
bta-miR-2303	-12.1	617
bta-miR-2303	-12.1	639
bta-miR-2303	-11.9	516
bta-miR-2303	-11.7	128
bta-miR-2303	-11.7	476
bta-miR-2303	-11.5	170
bta-miR-2303	-11.4	224
bta-miR-2303	-11.3	410
bta-miR-2303	-11.3	451
bta-miR-2303	-11.3	742
bta-miR-2303	-10.6	294
bta-miR-2303	-10.5	393
bta-miR-2285d	-17.2	51
bta-miR-2285d	-15.8	483
bta-miR-2285d	-15.0	596
bta-miR-2285d	-14.4	429
bta-miR-2285d	-13.9	237
bta-miR-2285d	-13.6	2
bta-miR-2285d	-13.6	283
bta-miR-2285d	-13.5	650
bta-miR-2285d	-13.4	331
bta-miR-2285d	-13.4	669
bta-miR-2285d	-13.2	408
bta-miR-2285d	-12.1	520
bta-miR-2285d	-12.0	563
bta-miR-2285d	-11.9	200
bta-miR-2285d	-11.7	164

bta-miR-2285d	-11.6	126
bta-miR-2285d	-11.5	30
bta-miR-2285d	-11.5	626
bta-miR-2285d	-11.5	99
bta-miR-2285d	-11.3	698
bta-miR-2285d	-11.3	277
bta-miR-2285d	-11.1	457
bta-miR-2285d	-10.9	308
bta-miR-2285d	-10.1	725
bta-miR-2304	-21.0	148
bta-miR-2304	-20.5	109
bta-miR-2304	-19.4	488
bta-miR-2304	-18.5	415
bta-miR-2304	-16.8	586
bta-miR-2304	-16.7	381
bta-miR-2304	-16.4	319
bta-miR-2304	-16.1	353
bta-miR-2304	-15.9	10
bta-miR-2304	-15.9	46
bta-miR-2304	-15.8	560
bta-miR-2304	-15.6	228
bta-miR-2304	-15.5	128
bta-miR-2304	-15.3	185
bta-miR-2304	-15.3	469
bta-miR-2304	-14.7	84
bta-miR-2304	-13.3	647
bta-miR-2304	-12.5	298
bta-miR-2304	-12.3	66
bta-miR-2304	-12.1	269
bta-miR-2304	-11.7	739
bta-miR-2304	-11.4	698
bta-miR-2304	-10.8	170
bta-miR-2304	-10.6	454
bta-miR-2304	-10.2	620
bta-miR-2305	-37.5	316
bta-miR-2305	-33.4	134
bta-miR-2305	-29.8	396
bta-miR-2305	-27.6	245
bta-miR-2305	-27.4	110
bta-miR-2305	-25.1	31
bta-miR-2305	-24.0	190
bta-miR-2305	-23.4	461
bta-miR-2305	-22.4	561
bta-miR-2305	-22.0	362
bta-miR-2305	-21.4	284
bta-miR-2305	-19.4	511
bta-miR-2305	-18.7	74
bta-miR-2305	-17.5	625
bta-miR-2305	-16.5	2
bta-miR-2305	-15.7	665
bta-miR-2305	-15.5	484
bta-miR-2305	-15.4	691
bta-miR-2305	-13.7	740
bta-miR-2305	-13.5	537
bta-miR-2305	-13.0	165
bta-miR-2305	-12.9	342
bta-miR-2305	-12.8	585
bta-miR-2305	-11.3	270
bta-miR-2305	-11.1	641

bta-miR-2305	-10.5	213
bta-miR-2305	-10.1	96
bta-miR-2306	-31.7	395
bta-miR-2306	-27.8	96
bta-miR-2306	-23.4	7
bta-miR-2306	-22.9	583
bta-miR-2306	-22.3	257
bta-miR-2306	-22.0	209
bta-miR-2306	-21.7	62
bta-miR-2306	-21.6	436
bta-miR-2306	-21.5	610
bta-miR-2306	-21.1	365
bta-miR-2306	-21.0	473
bta-miR-2306	-17.6	498
bta-miR-2306	-17.3	544
bta-miR-2306	-17.1	644
bta-miR-2306	-16.8	34
bta-miR-2306	-15.2	630
bta-miR-2306	-15.1	167
bta-miR-2306	-14.8	140
bta-miR-2306	-14.5	344
bta-miR-2306	-13.3	737
bta-miR-2306	-12.9	575
bta-miR-2306	-12.8	89
bta-miR-2306	-12.7	703
bta-miR-2306	-12.4	191
bta-miR-2306	-11.7	293
bta-miR-2306	-10.8	461
bta-miR-2306	-10.5	123
bta-miR-2307	-21.9	262
bta-miR-2307	-21.3	624
bta-miR-2307	-19.6	475
bta-miR-2307	-19.1	37
bta-miR-2307	-18.6	9
bta-miR-2307	-18.3	67
bta-miR-2307	-17.7	167
bta-miR-2307	-17.0	200
bta-miR-2307	-16.5	613
bta-miR-2307	-15.6	447
bta-miR-2307	-14.5	285
bta-miR-2307	-14.2	520
bta-miR-2307	-13.8	415
bta-miR-2307	-13.7	371
bta-miR-2307	-13.7	126
bta-miR-2307	-13.2	586
bta-miR-2307	-12.4	235
bta-miR-2307	-11.7	436
bta-miR-2307	-11.6	107
bta-miR-2307	-11.5	542
bta-miR-2307	-11.3	654
bta-miR-2307	-11.0	344
bta-miR-2307	-11.0	502
bta-miR-2307	-10.9	737
bta-miR-2307	-10.7	464
bta-miR-2307	-10.4	218
bta-miR-2308	-25.8	1
bta-miR-2308	-23.7	109
bta-miR-2308	-23.5	393
bta-miR-2308	-22.6	73

bta-miR-2308	-21.0	576
bta-miR-2308	-20.9	211
bta-miR-2308	-20.8	262
bta-miR-2308	-20.5	473
bta-miR-2308	-20.1	57
bta-miR-2308	-19.7	138
bta-miR-2308	-19.4	537
bta-miR-2308	-17.5	39
bta-miR-2308	-17.2	624
bta-miR-2308	-17.1	185
bta-miR-2308	-16.7	428
bta-miR-2308	-16.6	283
bta-miR-2308	-15.8	678
bta-miR-2308	-15.7	341
bta-miR-2308	-15.6	519
bta-miR-2308	-15.1	657
bta-miR-2308	-13.5	739
bta-miR-2308	-13.4	562
bta-miR-2308	-13.4	696
bta-miR-2308	-13.2	315
bta-miR-2308	-13.0	491
bta-miR-2308	-12.9	364
bta-miR-2308	-12.6	380
bta-miR-2308	-11.4	172
bta-miR-2308	-11.4	606
bta-miR-2308	-10.1	33
bta-miR-2308	-10.1	504
bta-miR-2309	-30.5	561
bta-miR-2309	-29.7	90
bta-miR-2309	-29.5	307
bta-miR-2309	-27.0	126
bta-miR-2309	-26.3	33
bta-miR-2309	-25.7	247
bta-miR-2309	-25.7	409
bta-miR-2309	-24.8	457
bta-miR-2309	-21.8	191
bta-miR-2309	-21.7	362
bta-miR-2309	-21.7	156
bta-miR-2309	-20.9	582
bta-miR-2309	-19.9	502
bta-miR-2309	-16.6	2
bta-miR-2309	-16.5	287
bta-miR-2309	-16.3	658
bta-miR-2309	-16.1	684
bta-miR-2309	-15.6	328
bta-miR-2309	-15.1	231
bta-miR-2309	-14.9	388
bta-miR-2309	-14.6	639
bta-miR-2309	-14.5	483
bta-miR-2309	-13.9	524
bta-miR-2309	-13.8	266
bta-miR-2309	-12.9	740
bta-miR-2309	-12.9	545
bta-miR-2309	-12.5	618
bta-miR-2309	-10.4	185
bta-miR-2309	-10.1	73
bta-miR-2310	-23.4	349
bta-miR-2310	-21.6	268
bta-miR-2310	-20.0	453

bta-miR-2310	-19.0	154
bta-miR-2310	-18.5	101
bta-miR-2310	-17.6	598
bta-miR-2310	-16.7	480
bta-miR-2310	-16.3	177
bta-miR-2310	-15.6	224
bta-miR-2310	-15.1	326
bta-miR-2310	-15.0	405
bta-miR-2310	-14.8	499
bta-miR-2310	-14.3	16
bta-miR-2310	-14.0	69
bta-miR-2310	-12.9	620
bta-miR-2310	-12.6	138
bta-miR-2310	-12.5	303
bta-miR-2310	-12.0	727
bta-miR-2310	-11.8	650
bta-miR-2310	-11.6	429
bta-miR-2310	-10.5	558
bta-miR-2310	-10.4	204
bta-miR-2310	-10.3	49
bta-miR-2310	-10.0	320
bta-miR-1603	-18.1	102
bta-miR-1603	-17.3	196
bta-miR-1603	-17.2	349
bta-miR-1603	-16.0	149
bta-miR-1603	-15.7	592
bta-miR-1603	-14.6	442
bta-miR-1603	-14.6	326
bta-miR-1603	-14.3	69
bta-miR-1603	-14.0	652
bta-miR-1603	-13.9	221
bta-miR-1603	-13.8	470
bta-miR-1603	-13.7	267
bta-miR-1603	-13.6	411
bta-miR-1603	-13.5	11
bta-miR-1603	-13.3	726
bta-miR-1603	-13.3	124
bta-miR-1603	-13.2	504
bta-miR-1603	-12.8	556
bta-miR-1603	-12.1	300
bta-miR-1603	-11.9	49
bta-miR-1603	-11.5	486
bta-miR-1603	-10.5	172
bta-miR-2284l	-18.9	470
bta-miR-2284l	-18.7	506
bta-miR-2284l	-17.8	357
bta-miR-2284l	-17.6	162
bta-miR-2284l	-17.0	402
bta-miR-2284l	-16.7	28
bta-miR-2284l	-16.3	108
bta-miR-2284l	-16.1	232
bta-miR-2284l	-15.7	273
bta-miR-2284l	-15.7	557
bta-miR-2284l	-15.5	47
bta-miR-2284l	-15.5	418
bta-miR-2284l	-15.4	659
bta-miR-2284l	-15.3	91
bta-miR-2284l	-14.8	689
bta-miR-2284l	-14.6	308

bta-miR-2284l	-14.5	620
bta-miR-2284l	-14.4	581
bta-miR-2284l	-14.1	431
bta-miR-2284l	-14.0	176
bta-miR-2284l	-14.0	592
bta-miR-2284l	-13.7	724
bta-miR-2284l	-13.6	11
bta-miR-2284l	-13.6	338
bta-miR-2284l	-13.6	706
bta-miR-2284l	-13.3	327
bta-miR-2284l	-13.1	196
bta-miR-2284l	-12.8	453
bta-miR-2284l	-11.3	142
bta-miR-2284l	-11.1	385
bta-miR-2284l	-10.1	79
bta-miR-2284l	-10.1	215
bta-miR-2284l	-10.0	320
bta-miR-2489	-22.6	570
bta-miR-2489	-21.4	17
bta-miR-2489	-20.9	129
bta-miR-2489	-19.5	256
bta-miR-2489	-18.9	92
bta-miR-2489	-18.5	349
bta-miR-2489	-18.5	622
bta-miR-2489	-18.0	455
bta-miR-2489	-17.2	418
bta-miR-2489	-17.1	49
bta-miR-2489	-17.0	273
bta-miR-2489	-16.5	247
bta-miR-2489	-16.4	313
bta-miR-2489	-16.3	371
bta-miR-2489	-16.0	2
bta-miR-2489	-15.3	178
bta-miR-2489	-14.8	546
bta-miR-2489	-14.7	204
bta-miR-2489	-14.7	508
bta-miR-2489	-14.5	395
bta-miR-2489	-14.1	701
bta-miR-2489	-13.2	329
bta-miR-2489	-13.0	586
bta-miR-2489	-12.9	672
bta-miR-2489	-12.9	154
bta-miR-2489	-12.2	737
bta-miR-2489	-12.1	648
bta-miR-2489	-12.0	531
bta-miR-2489	-11.5	235
bta-miR-2489	-11.1	165
bta-miR-2489	-10.7	305
bta-miR-2489	-10.3	71
bta-miR-2311	-22.6	593
bta-miR-2311	-22.4	367
bta-miR-2311	-21.6	483
bta-miR-2311	-20.7	119
bta-miR-2311	-20.2	429
bta-miR-2311	-19.6	53
bta-miR-2311	-19.4	408
bta-miR-2311	-19.3	234
bta-miR-2311	-19.2	160
bta-miR-2311	-18.3	9

bta-miR-2311	-16.8	649
bta-miR-2311	-16.7	341
bta-miR-2311	-16.7	271
bta-miR-2311	-16.5	453
bta-miR-2311	-16.4	199
bta-miR-2311	-16.3	510
bta-miR-2311	-15.9	624
bta-miR-2311	-15.4	563
bta-miR-2311	-15.2	28
bta-miR-2311	-14.1	109
bta-miR-2311	-13.2	306
bta-miR-2311	-12.9	86
bta-miR-2311	-11.9	669
bta-miR-2311	-11.8	138
bta-miR-2311	-11.5	536
bta-miR-2311	-11.3	218
bta-miR-2311	-10.7	705
bta-miR-2311	-10.6	609
bta-miR-2311	-10.0	473
bta-miR-2284j	-18.3	366
bta-miR-2284j	-16.9	405
bta-miR-2284j	-15.4	60
bta-miR-2284j	-15.3	166
bta-miR-2284j	-15.1	507
bta-miR-2284j	-15.1	621
bta-miR-2284j	-14.9	273
bta-miR-2284j	-14.9	443
bta-miR-2284j	-14.9	470
bta-miR-2284j	-14.7	592
bta-miR-2284j	-14.4	111
bta-miR-2284j	-14.4	11
bta-miR-2284j	-14.0	338
bta-miR-2284j	-13.7	423
bta-miR-2284j	-13.1	308
bta-miR-2284j	-12.6	706
bta-miR-2284j	-12.5	91
bta-miR-2284j	-12.5	196
bta-miR-2284j	-12.4	724
bta-miR-2284j	-11.6	229
bta-miR-2284j	-11.5	81
bta-miR-2284j	-11.3	581
bta-miR-2284j	-10.9	24
bta-miR-2284j	-10.9	216
bta-miR-2284j	-10.4	48
bta-miR-2284j	-10.4	124
bta-miR-2284j	-10.4	546
bta-miR-2284j	-10.3	490
bta-miR-2312	-14.1	611
bta-miR-2312	-13.9	657
bta-miR-2312	-13.6	2
bta-miR-2312	-13.3	449
bta-miR-2312	-12.8	265
bta-miR-2312	-12.2	47
bta-miR-2312	-11.8	530
bta-miR-2312	-11.2	405
bta-miR-2312	-10.7	36
bta-miR-2312	-10.7	597
bta-miR-2312	-10.5	225
bta-miR-2312	-10.4	291

bta-miR-2312	-10.3	551
bta-miR-2312	-10.0	202
bta-miR-2313	-26.1	248
bta-miR-2313	-26.1	408
bta-miR-2313	-25.0	473
bta-miR-2313	-24.3	562
bta-miR-2313	-24.2	88
bta-miR-2313	-24.0	167
bta-miR-2313	-22.9	360
bta-miR-2313	-22.7	502
bta-miR-2313	-22.2	624
bta-miR-2313	-20.9	3
bta-miR-2313	-20.7	30
bta-miR-2313	-18.0	426
bta-miR-2313	-17.7	206
bta-miR-2313	-17.7	276
bta-miR-2313	-16.1	697
bta-miR-2313	-16.0	654
bta-miR-2313	-15.7	531
bta-miR-2313	-15.0	307
bta-miR-2313	-14.9	231
bta-miR-2313	-14.9	126
bta-miR-2313	-14.9	457
bta-miR-2313	-14.3	191
bta-miR-2313	-13.4	395
bta-miR-2313	-12.2	606
bta-miR-2313	-11.9	69
bta-miR-2313	-11.0	21
bta-miR-2313	-10.8	155
bta-miR-2313	-10.4	285
bta-miR-2313*	-25.2	488
bta-miR-2313*	-24.7	66
bta-miR-2313*	-24.6	605
bta-miR-2313*	-22.8	408
bta-miR-2313*	-20.9	167
bta-miR-2313*	-20.8	17
bta-miR-2313*	-19.9	654
bta-miR-2313*	-18.4	266
bta-miR-2313*	-18.1	354
bta-miR-2313*	-17.9	424
bta-miR-2313*	-16.1	464
bta-miR-2313*	-15.9	107
bta-miR-2313*	-15.8	576
bta-miR-2313*	-15.8	544
bta-miR-2313*	-15.3	199
bta-miR-2313*	-15.3	518
bta-miR-2313*	-14.2	376
bta-miR-2313*	-13.1	230
bta-miR-2313*	-12.7	729
bta-miR-2313*	-11.8	305
bta-miR-2313*	-10.7	697
bta-miR-2313*	-10.5	119
bta-miR-2313*	-10.4	4
bta-miR-2284t*	-18.5	240
bta-miR-2284t*	-17.8	349
bta-miR-2284t*	-17.7	506
bta-miR-2284t*	-17.5	11
bta-miR-2284t*	-17.5	28
bta-miR-2284t*	-17.1	429

bta-miR-2284t*	-16.8	620
bta-miR-2284t*	-16.2	405
bta-miR-2284t*	-15.8	166
bta-miR-2284t*	-14.8	470
bta-miR-2284t*	-14.5	541
bta-miR-2284t*	-14.1	261
bta-miR-2284t*	-14.0	69
bta-miR-2284t*	-13.9	215
bta-miR-2284t*	-13.6	652
bta-miR-2284t*	-13.3	273
bta-miR-2284t*	-13.3	108
bta-miR-2284t*	-12.7	557
bta-miR-2284t*	-12.7	596
bta-miR-2284t*	-12.2	581
bta-miR-2284t*	-12.0	327
bta-miR-2284t*	-11.6	91
bta-miR-2284t*	-11.3	308
bta-miR-2284t*	-11.1	47
bta-miR-2284t*	-11.1	460
bta-miR-2284t*	-10.9	196
bta-miR-2284t*	-10.7	223
bta-miR-2284t*	-10.7	442
bta-miR-2284t*	-10.3	124
bta-miR-2284t*	-10.3	320
bta-miR-2284t*	-10.1	735
bta-miR-2284t	-19.1	331
bta-miR-2284t	-17.1	483
bta-miR-2284t	-16.3	199
bta-miR-2284t	-15.4	126
bta-miR-2284t	-15.1	596
bta-miR-2284t	-15.1	51
bta-miR-2284t	-14.7	156
bta-miR-2284t	-14.1	425
bta-miR-2284t	-13.7	8
bta-miR-2284t	-13.5	618
bta-miR-2284t	-13.1	374
bta-miR-2284t	-13.1	669
bta-miR-2284t	-12.9	299
bta-miR-2284t	-12.9	350
bta-miR-2284t	-12.5	698
bta-miR-2284t	-12.2	468
bta-miR-2284t	-12.0	515
bta-miR-2284t	-11.9	277
bta-miR-2284t	-11.8	99
bta-miR-2284t	-11.5	556
bta-miR-2284t	-11.4	535
bta-miR-2284t	-11.2	645
bta-miR-2284t	-11.1	244
bta-miR-2284t	-10.8	71
bta-miR-2284t	-10.6	408
bta-miR-2284t	-10.6	34
bta-miR-2284t	-10.0	361
bta-miR-2285b	-17.8	263
bta-miR-2285b	-15.8	5
bta-miR-2285b	-15.1	200
bta-miR-2285b	-14.8	34
bta-miR-2285b	-14.4	169
bta-miR-2285b	-14.3	613
bta-miR-2285b	-14.2	78

bta-miR-2285b	-14.0	704
bta-miR-2285b	-13.9	127
bta-miR-2285b	-13.8	237
bta-miR-2285b	-13.6	596
bta-miR-2285b	-12.8	652
bta-miR-2285b	-12.5	572
bta-miR-2285b	-12.5	384
bta-miR-2285b	-12.3	484
bta-miR-2285b	-12.2	539
bta-miR-2285b	-12.0	143
bta-miR-2285b	-12.0	449
bta-miR-2285b	-11.8	99
bta-miR-2285b	-11.4	468
bta-miR-2285b	-11.3	331
bta-miR-2285b	-10.8	431
bta-miR-2285b	-10.7	409
bta-miR-2285b	-10.3	16
bta-miR-2314	-22.1	44
bta-miR-2314	-21.4	252
bta-miR-2314	-20.8	598
bta-miR-2314	-20.6	4
bta-miR-2314	-20.3	482
bta-miR-2314	-19.9	410
bta-miR-2314	-18.0	85
bta-miR-2314	-17.4	645
bta-miR-2314	-16.9	566
bta-miR-2314	-16.5	198
bta-miR-2314	-16.1	620
bta-miR-2314	-15.9	165
bta-miR-2314	-15.2	277
bta-miR-2314	-15.2	449
bta-miR-2314	-15.0	377
bta-miR-2314	-12.8	140
bta-miR-2314	-11.8	389
bta-miR-2314	-11.6	344
bta-miR-2314	-11.4	26
bta-miR-2314	-10.9	544
bta-miR-2314	-10.9	692
bta-miR-2314	-10.5	516
bta-miR-2314	-10.4	182
bta-miR-2314	-10.3	222
bta-miR-2315	-22.0	462
bta-miR-2315	-19.5	91
bta-miR-2315	-18.3	409
bta-miR-2315	-18.1	164
bta-miR-2315	-18.0	41
bta-miR-2315	-17.6	232
bta-miR-2315	-16.6	515
bta-miR-2315	-16.1	376
bta-miR-2315	-16.0	480
bta-miR-2315	-14.6	205
bta-miR-2315	-14.5	263
bta-miR-2315	-14.5	572
bta-miR-2315	-14.4	663
bta-miR-2315	-14.0	127
bta-miR-2315	-13.7	9
bta-miR-2315	-12.7	622
bta-miR-2315	-12.7	596
bta-miR-2315	-12.4	546

bta-miR-2315	-12.2	495
bta-miR-2315	-12.0	325
bta-miR-2315	-11.9	392
bta-miR-2315	-11.8	305
bta-miR-2315	-11.8	734
bta-miR-2315	-11.5	67
bta-miR-2315	-11.4	358
bta-miR-2315	-10.9	431
bta-miR-2284d	-20.3	357
bta-miR-2284d	-18.1	102
bta-miR-2284d	-17.9	166
bta-miR-2284d	-17.5	405
bta-miR-2284d	-16.5	260
bta-miR-2284d	-16.4	429
bta-miR-2284d	-16.4	506
bta-miR-2284d	-16.1	47
bta-miR-2284d	-15.5	470
bta-miR-2284d	-15.2	441
bta-miR-2284d	-15.1	273
bta-miR-2284d	-14.3	240
bta-miR-2284d	-14.1	620
bta-miR-2284d	-14.1	188
bta-miR-2284d	-14.1	306
bta-miR-2284d	-13.8	327
bta-miR-2284d	-13.5	90
bta-miR-2284d	-12.9	689
bta-miR-2284d	-12.8	710
bta-miR-2284d	-12.6	28
bta-miR-2284d	-12.3	570
bta-miR-2284d	-12.1	214
bta-miR-2284d	-11.4	81
bta-miR-2284d	-11.2	596
bta-miR-2284d	-10.9	124
bta-miR-2284d	-10.4	223
bta-miR-2284d	-10.2	320
bta-miR-2284d	-10.2	340
bta-miR-2284d	-10.0	157
bta-miR-2316	-31.5	402
bta-miR-2316	-27.1	246
bta-miR-2316	-26.9	60
bta-miR-2316	-26.8	426
bta-miR-2316	-26.7	581
bta-miR-2316	-26.6	2
bta-miR-2316	-25.0	561
bta-miR-2316	-24.4	86
bta-miR-2316	-23.6	163
bta-miR-2316	-23.5	33
bta-miR-2316	-23.2	457
bta-miR-2316	-21.7	192
bta-miR-2316	-21.6	491
bta-miR-2316	-21.6	116
bta-miR-2316	-21.4	617
bta-miR-2316	-20.5	135
bta-miR-2316	-19.2	330
bta-miR-2316	-17.7	654
bta-miR-2316	-16.8	307
bta-miR-2316	-16.7	386
bta-miR-2316	-14.3	681
bta-miR-2316	-13.2	482

bta-miR-2316	-12.7	236
bta-miR-2316	-12.7	740
bta-miR-2316	-11.6	530
bta-miR-2316	-11.3	451
bta-miR-2316	-10.4	177
bta-miR-2316	-10.0	21
bta-miR-2317	-18.7	81
bta-miR-2317	-18.7	7
bta-miR-2317	-18.2	459
bta-miR-2317	-18.1	277
bta-miR-2317	-18.1	38
bta-miR-2317	-17.2	616
bta-miR-2317	-16.3	257
bta-miR-2317	-15.9	597
bta-miR-2317	-15.7	343
bta-miR-2317	-15.1	658
bta-miR-2317	-15.0	538
bta-miR-2317	-14.9	217
bta-miR-2317	-14.1	182
bta-miR-2317	-13.5	423
bta-miR-2317	-12.4	112
bta-miR-2317	-12.4	374
bta-miR-2317	-12.3	516
bta-miR-2317	-11.5	575
bta-miR-2317	-10.6	488
bta-miR-2317	-10.4	395
bta-miR-2317	-10.3	154
bta-miR-2317	-10.3	144
bta-miR-2317	-10.2	443
bta-miR-2317	-10.1	740
bta-miR-1343	-36.4	109
bta-miR-1343	-35.3	426
bta-miR-1343	-33.8	468
bta-miR-1343	-33.4	163
bta-miR-1343	-32.3	60
bta-miR-1343	-30.8	5
bta-miR-1343	-27.8	606
bta-miR-1343	-27.6	259
bta-miR-1343	-25.6	564
bta-miR-1343	-23.8	307
bta-miR-1343	-23.3	402
bta-miR-1343	-22.9	363
bta-miR-1343	-22.0	235
bta-miR-1343	-19.8	503
bta-miR-1343	-18.4	328
bta-miR-1343	-18.0	139
bta-miR-1343	-17.3	657
bta-miR-1343	-17.2	199
bta-miR-1343	-17.2	587
bta-miR-1343	-16.1	695
bta-miR-1343	-15.7	631
bta-miR-1343	-15.3	738
bta-miR-1343	-15.1	537
bta-miR-1343	-13.3	519
bta-miR-1343	-11.8	96
bta-miR-1343	-11.3	38
bta-miR-1343	-10.8	155
bta-miR-1343*	-35.9	614
bta-miR-1343*	-29.1	265

bta-miR-1343*	-24.8	450
bta-miR-1343*	-23.8	16
bta-miR-1343*	-23.4	221
bta-miR-1343*	-23.2	411
bta-miR-1343*	-22.4	80
bta-miR-1343*	-22.3	348
bta-miR-1343*	-20.5	497
bta-miR-1343*	-20.2	584
bta-miR-1343*	-18.9	155
bta-miR-1343*	-18.7	109
bta-miR-1343*	-18.0	376
bta-miR-1343*	-17.5	659
bta-miR-1343*	-17.4	69
bta-miR-1343*	-15.6	201
bta-miR-1343*	-14.9	244
bta-miR-1343*	-14.6	126
bta-miR-1343*	-13.8	251
bta-miR-1343*	-13.6	329
bta-miR-1343*	-13.5	544
bta-miR-1343*	-13.4	308
bta-miR-1343*	-13.4	176
bta-miR-1343*	-13.2	141
bta-miR-1343*	-12.8	323
bta-miR-1343*	-12.7	1
bta-miR-1343*	-12.5	482
bta-miR-1343*	-12.1	517
bta-miR-1343*	-11.7	697
bta-miR-1343*	-11.4	116
bta-miR-1343*	-10.8	441
bta-miR-1343*	-10.2	51
bta-miR-1343*	-10.2	737
bta-miR-2318	-16.6	8
bta-miR-2318	-15.7	277
bta-miR-2318	-13.7	576
bta-miR-2318	-12.9	200
bta-miR-2318	-12.8	371
bta-miR-2318	-12.2	38
bta-miR-2318	-12.1	464
bta-miR-2318	-12.0	126
bta-miR-2318	-11.5	629
bta-miR-2318	-11.4	681
bta-miR-2318	-11.3	235
bta-miR-2318	-10.9	89
bta-miR-2318	-10.6	617
bta-miR-2318	-10.2	344
bta-miR-2318	-10.1	544
bta-miR-2318	-10.0	175
bta-miR-2319a	-22.9	421
bta-miR-2319a	-21.0	356
bta-miR-2319a	-19.8	584
bta-miR-2319a	-19.5	78
bta-miR-2319a	-18.6	477
bta-miR-2319a	-18.0	3
bta-miR-2319a	-17.9	186
bta-miR-2319a	-17.5	112
bta-miR-2319a	-17.1	41
bta-miR-2319a	-16.0	604
bta-miR-2319a	-15.6	517
bta-miR-2319a	-15.0	216

bta-miR-2319a	-14.3	394
bta-miR-2319a	-14.1	558
bta-miR-2319a	-14.0	621
bta-miR-2319a	-13.9	342
bta-miR-2319a	-13.8	240
bta-miR-2319a	-13.4	167
bta-miR-2319a	-12.4	650
bta-miR-2319a	-12.2	20
bta-miR-2319a	-12.0	307
bta-miR-2319a	-11.4	152
bta-miR-2319a	-11.2	271
bta-miR-2319a	-11.0	740
bta-miR-2319a	-10.6	379
bta-miR-2319a	-10.2	697
bta-miR-2319b	-22.7	421
bta-miR-2319b	-20.9	356
bta-miR-2319b	-19.8	584
bta-miR-2319b	-19.5	78
bta-miR-2319b	-18.6	477
bta-miR-2319b	-17.9	186
bta-miR-2319b	-17.9	3
bta-miR-2319b	-17.5	112
bta-miR-2319b	-17.1	41
bta-miR-2319b	-15.6	517
bta-miR-2319b	-15.3	619
bta-miR-2319b	-14.9	216
bta-miR-2319b	-14.1	558
bta-miR-2319b	-13.9	342
bta-miR-2319b	-13.6	267
bta-miR-2319b	-13.4	167
bta-miR-2319b	-13.0	606
bta-miR-2319b	-12.6	411
bta-miR-2319b	-12.4	650
bta-miR-2319b	-12.2	20
bta-miR-2319b	-12.0	307
bta-miR-2319b	-11.4	152
bta-miR-2319b	-11.3	240
bta-miR-2319b	-11.0	740
bta-miR-2319b	-10.6	379
bta-miR-2319b	-10.2	697
bta-miR-2320	-31.6	408
bta-miR-2320	-29.0	563
bta-miR-2320	-28.4	364
bta-miR-2320	-27.8	6
bta-miR-2320	-26.0	92
bta-miR-2320	-25.1	483
bta-miR-2320	-22.9	606
bta-miR-2320	-22.0	61
bta-miR-2320	-21.2	436
bta-miR-2320	-21.2	167
bta-miR-2320	-20.7	237
bta-miR-2320	-19.6	261
bta-miR-2320	-17.8	517
bta-miR-2320	-17.7	730
bta-miR-2320	-17.6	126
bta-miR-2320	-17.0	199
bta-miR-2320	-16.4	342
bta-miR-2320	-16.3	698
bta-miR-2320	-16.1	653

bta-miR-2320	-15.3	311
bta-miR-2320	-14.3	387
bta-miR-2320	-13.9	537
bta-miR-2320	-13.7	221
bta-miR-2320	-13.0	591
bta-miR-2320	-12.9	473
bta-miR-2320	-11.5	327
bta-miR-2320	-11.3	51
bta-miR-2320	-11.1	187
bta-miR-2320	-10.2	639
bta-miR-2320*	-23.1	470
bta-miR-2320*	-21.3	599
bta-miR-2320*	-20.6	414
bta-miR-2320*	-20.4	80
bta-miR-2320*	-19.4	264
bta-miR-2320*	-19.1	199
bta-miR-2320*	-18.4	11
bta-miR-2320*	-18.3	446
bta-miR-2320*	-18.0	66
bta-miR-2320*	-17.8	103
bta-miR-2320*	-17.4	557
bta-miR-2320*	-17.2	371
bta-miR-2320*	-17.1	494
bta-miR-2320*	-16.8	628
bta-miR-2320*	-15.6	159
bta-miR-2320*	-15.3	216
bta-miR-2320*	-15.0	51
bta-miR-2320*	-14.4	735
bta-miR-2320*	-14.4	652
bta-miR-2320*	-13.3	350
bta-miR-2320*	-12.8	507
bta-miR-2320*	-11.6	303
bta-miR-2320*	-11.3	124
bta-miR-2320*	-11.0	238
bta-miR-2320*	-10.6	333
bta-miR-2320*	-10.4	622
bta-miR-2284n	-15.1	405
bta-miR-2284n	-15.0	240
bta-miR-2284n	-15.0	506
bta-miR-2284n	-14.5	108
bta-miR-2284n	-14.5	166
bta-miR-2284n	-14.4	620
bta-miR-2284n	-14.2	366
bta-miR-2284n	-14.0	273
bta-miR-2284n	-13.6	338
bta-miR-2284n	-13.6	47
bta-miR-2284n	-13.4	724
bta-miR-2284n	-13.0	429
bta-miR-2284n	-12.8	470
bta-miR-2284n	-12.8	308
bta-miR-2284n	-12.7	11
bta-miR-2284n	-12.5	596
bta-miR-2284n	-12.4	28
bta-miR-2284n	-11.2	460
bta-miR-2284n	-10.8	557
bta-miR-2284n	-10.7	91
bta-miR-2284n	-10.7	196
bta-miR-2284n	-10.6	574
bta-miR-2284n	-10.5	490

bta-miR-2284n	-10.4	124
bta-miR-2284n	-10.3	327
bta-miR-2284n	-10.2	215
bta-miR-2284g	-18.2	11
bta-miR-2284g	-18.1	443
bta-miR-2284g	-17.9	414
bta-miR-2284g	-17.6	166
bta-miR-2284g	-17.0	47
bta-miR-2284g	-16.3	620
bta-miR-2284g	-15.9	220
bta-miR-2284g	-15.4	102
bta-miR-2284g	-15.4	470
bta-miR-2284g	-15.0	196
bta-miR-2284g	-15.0	265
bta-miR-2284g	-14.9	83
bta-miR-2284g	-14.4	308
bta-miR-2284g	-14.3	586
bta-miR-2284g	-14.0	239
bta-miR-2284g	-14.0	124
bta-miR-2284g	-13.8	563
bta-miR-2284g	-13.3	405
bta-miR-2284g	-12.8	361
bta-miR-2284g	-12.6	604
bta-miR-2284g	-12.6	338
bta-miR-2284g	-12.4	178
bta-miR-2284g	-12.3	496
bta-miR-2284g	-11.8	735
bta-miR-2284g	-11.7	153
bta-miR-2284g	-11.6	434
bta-miR-2284g	-11.5	28
bta-miR-2284g	-11.2	279
bta-miR-2284g	-10.9	663
bta-miR-2284g	-10.2	36
bta-miR-2284g	-10.0	698
bta-miR-2321	-18.9	351
bta-miR-2321	-18.5	171
bta-miR-2321	-18.0	449
bta-miR-2321	-18.0	558
bta-miR-2321	-17.8	731
bta-miR-2321	-17.7	197
bta-miR-2321	-17.3	114
bta-miR-2321	-17.1	46
bta-miR-2321	-16.3	405
bta-miR-2321	-16.1	10
bta-miR-2321	-15.8	573
bta-miR-2321	-15.4	505
bta-miR-2321	-15.2	100
bta-miR-2321	-15.2	471
bta-miR-2321	-15.0	332
bta-miR-2321	-14.2	149
bta-miR-2321	-14.0	228
bta-miR-2321	-13.9	603
bta-miR-2321	-13.7	419
bta-miR-2321	-13.2	79
bta-miR-2321	-13.0	164
bta-miR-2321	-12.9	312
bta-miR-2321	-12.9	651
bta-miR-2321	-11.6	272
bta-miR-2321	-11.5	698

bta-miR-2321	-10.7	595
bta-miR-1721	-20.6	626
bta-miR-1721	-19.6	39
bta-miR-1721	-19.1	200
bta-miR-1721	-19.1	276
bta-miR-1721	-17.6	476
bta-miR-1721	-17.5	502
bta-miR-1721	-17.2	376
bta-miR-1721	-15.4	139
bta-miR-1721	-15.1	9
bta-miR-1721	-14.9	567
bta-miR-1721	-14.7	98
bta-miR-1721	-14.3	396
bta-miR-1721	-13.9	262
bta-miR-1721	-13.6	345
bta-miR-1721	-13.4	654
bta-miR-1721	-13.4	692
bta-miR-1721	-12.7	597
bta-miR-1721	-12.5	232
bta-miR-1721	-12.1	177
bta-miR-1721	-12.0	310
bta-miR-1721	-12.0	533
bta-miR-1721	-11.5	67
bta-miR-1721	-10.9	450
bta-miR-2322	-22.1	614
bta-miR-2322	-20.8	709
bta-miR-2322	-20.6	412
bta-miR-2322	-20.0	478
bta-miR-2322	-20.0	266
bta-miR-2322	-18.8	438
bta-miR-2322	-18.1	217
bta-miR-2322	-18.0	355
bta-miR-2322	-17.4	69
bta-miR-2322	-16.4	174
bta-miR-2322	-16.3	658
bta-miR-2322	-15.9	26
bta-miR-2322	-15.9	11
bta-miR-2322	-15.8	729
bta-miR-2322	-14.2	113
bta-miR-2322	-12.8	569
bta-miR-2322	-12.1	694
bta-miR-2322	-11.3	598
bta-miR-2322	-10.8	538
bta-miR-2322	-10.1	57
bta-miR-2322*	-25.2	196
bta-miR-2322*	-24.8	225
bta-miR-2322*	-24.2	69
bta-miR-2322*	-23.6	445
bta-miR-2322*	-20.5	414
bta-miR-2322*	-20.1	348
bta-miR-2322*	-19.4	557
bta-miR-2322*	-19.1	153
bta-miR-2322*	-17.8	280
bta-miR-2322*	-17.7	38
bta-miR-2322*	-17.6	620
bta-miR-2322*	-17.5	114
bta-miR-2322*	-16.9	178
bta-miR-2322*	-16.5	479
bta-miR-2322*	-16.0	507

bta-miR-2322*	-16.0	11
bta-miR-2322*	-16.0	389
bta-miR-2322*	-15.1	464
bta-miR-2322*	-14.4	575
bta-miR-2322*	-14.0	253
bta-miR-2322*	-13.6	698
bta-miR-2322*	-12.6	143
bta-miR-2322*	-12.5	97
bta-miR-2322*	-12.3	735
bta-miR-2322*	-12.2	663
bta-miR-2322*	-12.0	313
bta-miR-2322*	-11.5	586
bta-miR-2322*	-11.3	27
bta-miR-2322*	-11.3	435
bta-miR-2322*	-11.1	527
bta-miR-2322*	-10.8	600
bta-miR-2322*	-10.5	650
bta-miR-2322*	-10.4	372
bta-miR-2323	-25.3	13
bta-miR-2323	-25.0	262
bta-miR-2323	-20.7	645
bta-miR-2323	-19.6	408
bta-miR-2323	-18.9	597
bta-miR-2323	-17.8	477
bta-miR-2323	-17.7	551
bta-miR-2323	-17.3	200
bta-miR-2323	-17.0	450
bta-miR-2323	-16.0	617
bta-miR-2323	-15.9	67
bta-miR-2323	-14.3	376
bta-miR-2323	-13.2	165
bta-miR-2323	-13.0	344
bta-miR-2323	-12.4	235
bta-miR-2323	-12.2	107
bta-miR-2323	-11.9	520
bta-miR-2323	-11.4	49
bta-miR-2323	-11.2	358
bta-miR-2323	-11.0	579
bta-miR-2323	-10.7	738
bta-miR-2323	-10.4	432
bta-miR-2324	-37.3	125
bta-miR-2324	-29.9	80
bta-miR-2324	-29.9	308
bta-miR-2324	-29.7	349
bta-miR-2324	-28.8	414
bta-miR-2324	-27.9	155
bta-miR-2324	-27.8	456
bta-miR-2324	-27.2	234
bta-miR-2324	-26.7	10
bta-miR-2324	-24.8	376
bta-miR-2324	-24.2	559
bta-miR-2324	-23.9	50
bta-miR-2324	-21.9	482
bta-miR-2324	-20.3	183
bta-miR-2324	-18.9	602
bta-miR-2324	-18.5	653
bta-miR-2324	-18.0	265
bta-miR-2324	-17.8	511
bta-miR-2324	-16.4	114

bta-miR-2324	-16.2	582
bta-miR-2324	-15.6	698
bta-miR-2324	-14.3	401
bta-miR-2324	-14.3	737
bta-miR-2324	-14.1	532
bta-miR-2324	-12.4	1
bta-miR-2324	-11.5	37
bta-miR-2324	-11.0	147
bta-miR-2324	-10.6	170
bta-miR-2324	-10.2	682
bta-miR-2324	-10.1	67
bta-miR-2324	-10.1	337
bta-miR-2325a	-18.3	348
bta-miR-2325a	-16.6	451
bta-miR-2325a	-16.5	102
bta-miR-2325a	-16.4	479
bta-miR-2325a	-16.3	166
bta-miR-2325a	-15.4	223
bta-miR-2325a	-15.0	327
bta-miR-2325a	-14.5	405
bta-miR-2325a	-14.1	268
bta-miR-2325a	-13.8	142
bta-miR-2325a	-13.7	506
bta-miR-2325a	-13.4	188
bta-miR-2325a	-13.4	28
bta-miR-2325a	-12.9	47
bta-miR-2325a	-12.3	592
bta-miR-2325a	-11.8	650
bta-miR-2325a	-11.6	429
bta-miR-2325a	-11.4	724
bta-miR-2325a	-10.9	308
bta-miR-2325a	-10.7	620
bta-miR-2325a	-10.3	135
bta-miR-2325a	-10.0	320
bta-miR-2326	-28.8	437
bta-miR-2326	-28.5	596
bta-miR-2326	-23.3	409
bta-miR-2326	-21.9	67
bta-miR-2326	-21.8	466
bta-miR-2326	-20.5	491
bta-miR-2326	-18.6	236
bta-miR-2326	-18.3	620
bta-miR-2326	-18.1	164
bta-miR-2326	-16.5	209
bta-miR-2326	-16.1	98
bta-miR-2326	-14.6	5
bta-miR-2326	-13.1	513
bta-miR-2326	-12.6	452
bta-miR-2326	-11.8	127
bta-miR-2326	-11.6	345
bta-miR-2326	-11.6	200
bta-miR-2326	-11.1	25
bta-miR-2326	-11.1	400
bta-miR-2326	-10.6	363
bta-miR-2326	-10.5	567
bta-miR-2326	-10.4	316
bta-miR-2326	-10.2	390
bta-miR-2326	-10.1	90
bta-miR-2327	-33.2	408

bta-miR-2327	-32.1	8
bta-miR-2327	-27.6	229
bta-miR-2327	-25.5	515
bta-miR-2327	-24.2	654
bta-miR-2327	-23.6	81
bta-miR-2327	-23.1	344
bta-miR-2327	-22.6	381
bta-miR-2327	-22.6	544
bta-miR-2327	-22.0	119
bta-miR-2327	-21.4	277
bta-miR-2327	-20.7	483
bta-miR-2327	-20.2	164
bta-miR-2327	-20.1	454
bta-miR-2327	-20.1	618
bta-miR-2327	-17.9	38
bta-miR-2327	-17.8	581
bta-miR-2327	-16.0	328
bta-miR-2327	-16.0	199
bta-miR-2327	-15.6	257
bta-miR-2327	-13.8	698
bta-miR-2327	-13.1	66
bta-miR-2327	-12.5	730
bta-miR-2327	-12.4	263
bta-miR-2327	-11.9	433
bta-miR-2327	-11.8	603
bta-miR-2328	-36.9	596
bta-miR-2328	-29.9	40
bta-miR-2328	-29.7	462
bta-miR-2328	-26.4	424
bta-miR-2328	-22.6	402
bta-miR-2328	-21.9	155
bta-miR-2328	-21.8	564
bta-miR-2328	-19.2	17
bta-miR-2328	-18.8	257
bta-miR-2328	-16.7	92
bta-miR-2328	-16.6	632
bta-miR-2328	-16.6	495
bta-miR-2328	-16.1	220
bta-miR-2328	-15.6	363
bta-miR-2328	-14.5	740
bta-miR-2328	-13.8	185
bta-miR-2328	-11.4	30
bta-miR-2328	-11.4	342
bta-miR-2328	-10.7	117
bta-miR-2328	-10.7	235
bta-miR-2328	-10.4	275
bta-miR-2328	-10.1	12
bta-miR-2328*	-32.8	242
bta-miR-2328*	-30.4	395
bta-miR-2328*	-29.5	155
bta-miR-2328*	-28.2	576
bta-miR-2328*	-27.8	134
bta-miR-2328*	-26.3	356
bta-miR-2328*	-26.1	464
bta-miR-2328*	-25.0	85
bta-miR-2328*	-24.9	115
bta-miR-2328*	-24.9	424
bta-miR-2328*	-24.7	321
bta-miR-2328*	-24.5	185

bta-miR-2328*	-23.3	27
bta-miR-2328*	-23.1	204
bta-miR-2328*	-21.8	507
bta-miR-2328*	-19.2	623
bta-miR-2328*	-18.0	709
bta-miR-2328*	-17.2	563
bta-miR-2328*	-16.4	5
bta-miR-2328*	-16.4	488
bta-miR-2328*	-15.9	271
bta-miR-2328*	-15.3	308
bta-miR-2328*	-14.9	669
bta-miR-2328*	-13.3	109
bta-miR-2328*	-13.1	72
bta-miR-2328*	-12.6	741
bta-miR-2328*	-12.3	691
bta-miR-2328*	-11.8	378
bta-miR-2328*	-11.6	284
bta-miR-2328*	-11.5	657
bta-miR-2328*	-11.4	342
bta-miR-2328*	-11.2	610
bta-miR-2328*	-10.9	419
bta-miR-2328*	-10.5	543
bta-miR-2328*	-10.1	236
bta-miR-2328*	-10.0	447
bta-miR-2329-3p	-20.2	182
bta-miR-2329-3p	-18.9	457
bta-miR-2329-3p	-18.7	43
bta-miR-2329-3p	-17.5	86
bta-miR-2329-3p	-17.1	113
bta-miR-2329-3p	-16.2	549
bta-miR-2329-3p	-16.0	369
bta-miR-2329-3p	-16.0	263
bta-miR-2329-3p	-15.7	7
bta-miR-2329-3p	-15.4	420
bta-miR-2329-3p	-15.1	205
bta-miR-2329-3p	-15.1	490
bta-miR-2329-3p	-14.7	138
bta-miR-2329-3p	-14.3	619
bta-miR-2329-3p	-14.2	597
bta-miR-2329-3p	-14.1	165
bta-miR-2329-3p	-13.9	282
bta-miR-2329-3p	-13.0	342
bta-miR-2329-3p	-12.8	228
bta-miR-2329-3p	-12.7	642
bta-miR-2329-3p	-12.2	692
bta-miR-2329-3p	-12.1	577
bta-miR-2329-3p	-11.9	393
bta-miR-2329-3p	-11.8	25
bta-miR-2329-3p	-11.1	737
bta-miR-2329-3p	-10.7	249
bta-miR-2329-3p	-10.4	72
bta-miR-2329-3p	-10.4	407
bta-miR-2329-3p	-10.2	107
bta-miR-2329-3p	-10.1	156
bta-miR-2329-3p	-10.1	520
bta-miR-2329-5p	-23.7	19
bta-miR-2329-5p	-21.2	60
bta-miR-2329-5p	-20.8	412
bta-miR-2329-5p	-19.3	567

bta-miR-2329-5p	-18.8	87
bta-miR-2329-5p	-18.6	617
bta-miR-2329-5p	-18.0	537
bta-miR-2329-5p	-15.6	5
bta-miR-2329-5p	-15.1	229
bta-miR-2329-5p	-14.9	263
bta-miR-2329-5p	-14.9	359
bta-miR-2329-5p	-14.7	448
bta-miR-2329-5p	-13.3	178
bta-miR-2329-5p	-13.3	217
bta-miR-2329-5p	-13.2	476
bta-miR-2329-5p	-12.5	518
bta-miR-2329-5p	-12.4	40
bta-miR-2329-5p	-12.2	660
bta-miR-2329-5p	-11.7	204
bta-miR-2329-5p	-11.7	386
bta-miR-2329-5p	-10.9	598
bta-miR-2329-5p	-10.7	730
bta-miR-2329-5p	-10.7	497
bta-miR-2329-5p	-10.6	242
bta-miR-2329-5p	-10.6	559
bta-miR-2329-5p	-10.4	633
bta-miR-2330	-34.8	107
bta-miR-2330	-29.6	462
bta-miR-2330	-27.5	67
bta-miR-2330	-27.3	136
bta-miR-2330	-27.3	409
bta-miR-2330	-26.1	234
bta-miR-2330	-25.4	515
bta-miR-2330	-25.0	9
bta-miR-2330	-24.6	572
bta-miR-2330	-22.4	309
bta-miR-2330	-21.9	191
bta-miR-2330	-20.5	342
bta-miR-2330	-20.4	376
bta-miR-2330	-20.4	545
bta-miR-2330	-20.2	261
bta-miR-2330	-19.7	34
bta-miR-2330	-19.5	639
bta-miR-2330	-16.8	607
bta-miR-2330	-15.5	681
bta-miR-2330	-15.2	655
bta-miR-2330	-13.6	286
bta-miR-2330	-13.0	398
bta-miR-2330	-12.8	739
bta-miR-2330	-12.0	55
bta-miR-2330	-11.8	211
bta-miR-2330	-11.8	495
bta-miR-2330	-11.5	1
bta-miR-2330	-11.5	485
bta-miR-2330	-11.1	455
bta-miR-2330	-10.7	432
bta-miR-2330	-10.3	703
bta-miR-2330*	-29.6	605
bta-miR-2330*	-25.3	424
bta-miR-2330*	-21.2	12
bta-miR-2330*	-20.9	66
bta-miR-2330*	-19.3	263
bta-miR-2330*	-19.0	624

bta-miR-2330*	-18.8	405
bta-miR-2330*	-18.8	479
bta-miR-2330*	-17.3	106
bta-miR-2330*	-16.0	167
bta-miR-2330*	-15.8	206
bta-miR-2330*	-14.5	576
bta-miR-2330*	-14.4	229
bta-miR-2330*	-13.6	94
bta-miR-2330*	-13.3	126
bta-miR-2330*	-12.6	155
bta-miR-2330*	-12.5	359
bta-miR-2330*	-12.0	116
bta-miR-2330*	-11.7	560
bta-miR-2330*	-11.5	30
bta-miR-2330*	-11.4	85
bta-miR-2330*	-11.0	661
bta-miR-2330*	-10.6	738
bta-miR-2330*	-10.5	51
bta-miR-2330*	-10.5	698
bta-miR-2330*	-10.3	457
bta-miR-2331	-33.2	1
bta-miR-2331	-27.6	407
bta-miR-2331	-27.3	604
bta-miR-2331	-27.1	57
bta-miR-2331	-27.0	488
bta-miR-2331	-22.9	454
bta-miR-2331	-22.1	94
bta-miR-2331	-20.9	203
bta-miR-2331	-20.8	371
bta-miR-2331	-20.4	563
bta-miR-2331	-19.8	635
bta-miR-2331	-19.4	263
bta-miR-2331	-18.6	149
bta-miR-2331	-18.6	343
bta-miR-2331	-18.2	39
bta-miR-2331	-16.4	172
bta-miR-2331	-14.3	695
bta-miR-2331	-13.5	291
bta-miR-2331	-13.4	737
bta-miR-2331	-13.3	121
bta-miR-2331	-13.0	434
bta-miR-2331	-12.5	81
bta-miR-2331	-12.2	548
bta-miR-2331	-10.8	188
bta-miR-2331	-10.7	592
bta-miR-2331	-10.4	519
bta-miR-2331	-10.0	129
bta-miR-2331*	-25.4	472
bta-miR-2331*	-24.0	393
bta-miR-2331*	-22.6	58
bta-miR-2331*	-22.1	72
bta-miR-2331*	-22.0	586
bta-miR-2331*	-20.8	617
bta-miR-2331*	-19.6	108
bta-miR-2331*	-18.4	5
bta-miR-2331*	-17.7	282
bta-miR-2331*	-17.5	155
bta-miR-2331*	-16.8	653
bta-miR-2331*	-16.4	425

bta-miR-2331*	-16.1	741
bta-miR-2331*	-15.3	551
bta-miR-2331*	-15.1	243
bta-miR-2331*	-15.0	205
bta-miR-2331*	-14.7	183
bta-miR-2331*	-13.7	29
bta-miR-2331*	-13.7	691
bta-miR-2331*	-13.4	456
bta-miR-2331*	-13.2	517
bta-miR-2331*	-13.2	637
bta-miR-2331*	-13.0	358
bta-miR-2331*	-11.0	679
bta-miR-2331*	-10.7	703
bta-miR-2331*	-10.0	502
bta-miR-2332	-25.7	577
bta-miR-2332	-24.1	395
bta-miR-2332	-22.6	2
bta-miR-2332	-22.3	466
bta-miR-2332	-20.0	88
bta-miR-2332	-19.8	491
bta-miR-2332	-18.5	549
bta-miR-2332	-18.5	359
bta-miR-2332	-17.5	257
bta-miR-2332	-16.9	513
bta-miR-2332	-16.8	291
bta-miR-2332	-16.8	412
bta-miR-2332	-16.3	139
bta-miR-2332	-16.0	642
bta-miR-2332	-15.9	233
bta-miR-2332	-15.6	615
bta-miR-2332	-15.5	170
bta-miR-2332	-15.2	43
bta-miR-2332	-15.1	331
bta-miR-2332	-14.6	344
bta-miR-2332	-14.6	566
bta-miR-2332	-14.4	736
bta-miR-2332	-14.0	707
bta-miR-2332	-13.9	436
bta-miR-2332	-13.8	70
bta-miR-2332	-13.6	677
bta-miR-2332	-12.9	214
bta-miR-2332	-11.4	374
bta-miR-2332	-10.6	537
bta-miR-2332	-10.5	34
bta-miR-2332	-10.2	202
bta-miR-2332	-10.2	132
bta-miR-2333	-30.2	622
bta-miR-2333	-27.4	473
bta-miR-2333	-26.5	109
bta-miR-2333	-25.0	71
bta-miR-2333	-23.4	12
bta-miR-2333	-23.1	414
bta-miR-2333	-22.8	368
bta-miR-2333	-21.6	269
bta-miR-2333	-19.3	499
bta-miR-2333	-17.9	563
bta-miR-2333	-17.8	51
bta-miR-2333	-17.7	220
bta-miR-2333	-17.6	167

bta-miR-2333	-16.3	33
bta-miR-2333	-16.3	596
bta-miR-2333	-16.2	539
bta-miR-2333	-15.5	450
bta-miR-2333	-15.3	361
bta-miR-2333	-14.2	663
bta-miR-2333	-13.8	389
bta-miR-2333	-13.4	740
bta-miR-2333	-13.0	401
bta-miR-2333	-12.8	4
bta-miR-2333	-11.9	615
bta-miR-2333	-10.9	155
bta-miR-2333	-10.7	707
bta-miR-2333	-10.5	330
bta-miR-2333	-10.5	209
bta-miR-2333	-10.0	97
bta-miR-2333	-10.0	308
bta-miR-2334	-26.9	616
bta-miR-2334	-22.0	404
bta-miR-2334	-22.0	16
bta-miR-2334	-21.7	69
bta-miR-2334	-21.7	499
bta-miR-2334	-21.4	266
bta-miR-2334	-21.4	435
bta-miR-2334	-21.3	102
bta-miR-2334	-20.4	730
bta-miR-2334	-20.2	598
bta-miR-2334	-19.6	202
bta-miR-2334	-18.9	478
bta-miR-2334	-18.3	347
bta-miR-2334	-17.5	176
bta-miR-2334	-17.3	377
bta-miR-2334	-17.0	566
bta-miR-2334	-15.5	240
bta-miR-2334	-15.2	149
bta-miR-2334	-14.3	423
bta-miR-2334	-14.0	653
bta-miR-2334	-13.4	544
bta-miR-2334	-12.0	159
bta-miR-2334	-11.8	10
bta-miR-2334	-11.3	56
bta-miR-2334	-10.6	121
bta-miR-2334	-10.2	293
bta-miR-2335	-16.8	37
bta-miR-2335	-14.8	263
bta-miR-2335	-14.8	200
bta-miR-2335	-13.4	516
bta-miR-2335	-13.4	9
bta-miR-2335	-13.3	386
bta-miR-2335	-12.6	476
bta-miR-2335	-12.2	638
bta-miR-2335	-11.6	238
bta-miR-2335	-11.3	545
bta-miR-2335	-10.9	290
bta-miR-2335	-10.4	80
bta-miR-2335	-10.0	1
bta-miR-2335	-10.0	449
bta-miR-2335	-10.0	613
bta-miR-2336	-17.5	487

bta-miR-2336	-17.4	70
bta-miR-2336	-16.6	205
bta-miR-2336	-16.0	632
bta-miR-2336	-15.8	424
bta-miR-2336	-14.4	548
bta-miR-2336	-14.4	605
bta-miR-2336	-14.3	56
bta-miR-2336	-13.9	517
bta-miR-2336	-13.7	166
bta-miR-2336	-13.4	22
bta-miR-2336	-13.1	394
bta-miR-2336	-12.7	381
bta-miR-2336	-12.6	734
bta-miR-2336	-12.1	270
bta-miR-2336	-12.0	464
bta-miR-2336	-11.6	584
bta-miR-2336	-11.2	282
bta-miR-2336	-11.0	243
bta-miR-2336	-10.4	108
bta-miR-2336	-10.4	185
bta-miR-2336	-10.2	343
bta-miR-2337	-22.4	443
bta-miR-2337	-19.9	610
bta-miR-2337	-16.9	499
bta-miR-2337	-16.3	226
bta-miR-2337	-15.9	470
bta-miR-2337	-15.6	174
bta-miR-2337	-15.3	113
bta-miR-2337	-15.0	338
bta-miR-2337	-14.0	405
bta-miR-2337	-13.9	371
bta-miR-2337	-13.6	725
bta-miR-2337	-13.3	36
bta-miR-2337	-13.0	68
bta-miR-2337	-12.9	574
bta-miR-2337	-12.6	11
bta-miR-2337	-12.5	268
bta-miR-2337	-12.4	418
bta-miR-2337	-11.4	201
bta-miR-2337	-11.3	630
bta-miR-2337	-11.2	558
bta-miR-2337	-11.2	313
bta-miR-2337	-11.1	218
bta-miR-2337	-10.8	124
bta-miR-2337	-10.5	355
bta-miR-2337	-10.3	151
bta-miR-2337	-10.2	663
bta-miR-2337	-10.1	129
bta-miR-199c	-21.3	515
bta-miR-199c	-21.3	409
bta-miR-199c	-18.7	376
bta-miR-199c	-18.4	34
bta-miR-199c	-18.3	572
bta-miR-199c	-18.0	618
bta-miR-199c	-17.3	7
bta-miR-199c	-16.9	92
bta-miR-199c	-16.5	263
bta-miR-199c	-16.0	199
bta-miR-199c	-16.0	655

bta-miR-199c	-15.8	359
bta-miR-199c	-15.3	544
bta-miR-199c	-15.3	69
bta-miR-199c	-15.0	632
bta-miR-199c	-14.8	109
bta-miR-199c	-14.6	123
bta-miR-199c	-14.4	484
bta-miR-199c	-13.7	185
bta-miR-199c	-13.4	728
bta-miR-199c	-13.3	229
bta-miR-199c	-13.1	143
bta-miR-199c	-12.5	462
bta-miR-199c	-11.8	606
bta-miR-199c	-11.5	342
bta-miR-199c	-10.7	697
bta-miR-199c	-10.5	164
bta-miR-2338	-33.3	24
bta-miR-2338	-31.7	235
bta-miR-2338	-28.6	107
bta-miR-2338	-27.9	388
bta-miR-2338	-26.2	463
bta-miR-2338	-25.8	415
bta-miR-2338	-25.4	565
bta-miR-2338	-25.2	165
bta-miR-2338	-22.5	69
bta-miR-2338	-22.2	353
bta-miR-2338	-20.8	619
bta-miR-2338	-20.4	493
bta-miR-2338	-20.3	202
bta-miR-2338	-19.2	3
bta-miR-2338	-18.8	543
bta-miR-2338	-18.7	128
bta-miR-2338	-18.6	282
bta-miR-2338	-17.0	653
bta-miR-2338	-16.2	319
bta-miR-2338	-16.1	55
bta-miR-2338	-15.0	678
bta-miR-2338	-12.4	191
bta-miR-2338	-12.2	737
bta-miR-2338	-11.6	596
bta-miR-2338	-11.5	708
bta-miR-2338	-11.2	524
bta-miR-2338	-10.7	276
bta-miR-2339	-24.3	414
bta-miR-2339	-23.8	487
bta-miR-2339	-21.9	9
bta-miR-2339	-21.2	85
bta-miR-2339	-20.8	559
bta-miR-2339	-20.7	205
bta-miR-2339	-19.7	623
bta-miR-2339	-19.3	243
bta-miR-2339	-18.1	108
bta-miR-2339	-17.8	166
bta-miR-2339	-17.7	457
bta-miR-2339	-17.4	472
bta-miR-2339	-17.3	605
bta-miR-2339	-17.1	381
bta-miR-2339	-16.9	50
bta-miR-2339	-16.8	520

bta-miR-2339	-16.1	653
bta-miR-2339	-14.4	361
bta-miR-2339	-14.4	29
bta-miR-2339	-14.3	694
bta-miR-2339	-13.7	539
bta-miR-2339	-13.0	586
bta-miR-2339	-12.6	282
bta-miR-2339	-11.7	403
bta-miR-2339	-11.6	742
bta-miR-2339	-11.3	308
bta-miR-2339	-11.1	435
bta-miR-2339	-10.5	641
bta-miR-2339	-10.0	154
bta-miR-1814a	-21.9	227
bta-miR-1814a	-19.3	469
bta-miR-1814a	-18.2	333
bta-miR-1814a	-17.4	557
bta-miR-1814a	-16.5	405
bta-miR-1814a	-15.8	103
bta-miR-1814a	-15.8	494
bta-miR-1814a	-15.2	268
bta-miR-1814a	-14.8	188
bta-miR-1814a	-14.4	441
bta-miR-1814a	-14.4	122
bta-miR-1814a	-14.2	355
bta-miR-1814a	-14.2	151
bta-miR-1814a	-13.9	589
bta-miR-1814a	-13.8	22
bta-miR-1814a	-12.6	726
bta-miR-1814a	-12.4	301
bta-miR-1814a	-12.0	371
bta-miR-1814a	-11.6	616
bta-miR-1814a	-11.6	65
bta-miR-1814a	-11.6	652
bta-miR-1814a	-11.5	171
bta-miR-1814a	-10.4	573
bta-miR-2340	-25.4	67
bta-miR-2340	-25.1	150
bta-miR-2340	-25.0	597
bta-miR-2340	-24.9	420
bta-miR-2340	-24.0	477
bta-miR-2340	-23.5	199
bta-miR-2340	-21.0	106
bta-miR-2340	-20.4	373
bta-miR-2340	-18.7	621
bta-miR-2340	-18.0	240
bta-miR-2340	-18.0	22
bta-miR-2340	-17.6	558
bta-miR-2340	-16.7	264
bta-miR-2340	-16.4	354
bta-miR-2340	-16.2	300
bta-miR-2340	-15.0	405
bta-miR-2340	-14.6	49
bta-miR-2340	-14.6	94
bta-miR-2340	-14.3	698
bta-miR-2340	-14.2	4
bta-miR-2340	-14.0	319
bta-miR-2340	-13.4	736
bta-miR-2340	-13.0	129

bta-miR-2340	-12.7	651
bta-miR-2340	-11.7	614
bta-miR-2340	-11.2	185
bta-miR-2340	-10.5	520
bta-miR-2341	-22.5	107
bta-miR-2341	-21.5	561
bta-miR-2341	-21.0	393
bta-miR-2341	-19.4	277
bta-miR-2341	-19.4	456
bta-miR-2341	-18.2	511
bta-miR-2341	-17.6	419
bta-miR-2341	-17.0	72
bta-miR-2341	-16.5	632
bta-miR-2341	-16.3	165
bta-miR-2341	-15.7	669
bta-miR-2341	-15.5	50
bta-miR-2341	-15.4	308
bta-miR-2341	-14.9	361
bta-miR-2341	-14.5	9
bta-miR-2341	-14.4	125
bta-miR-2341	-14.3	243
bta-miR-2341	-13.9	482
bta-miR-2341	-13.8	716
bta-miR-2341	-13.2	198
bta-miR-2341	-12.2	539
bta-miR-2341	-10.9	29
bta-miR-2341	-10.7	495
bta-miR-2341	-10.3	263
bta-miR-2341	-10.2	623
bta-miR-2341	-10.1	742
bta-miR-2342	-26.9	51
bta-miR-2342	-25.1	479
bta-miR-2342	-24.0	175
bta-miR-2342	-22.3	204
bta-miR-2342	-21.7	584
bta-miR-2342	-20.0	557
bta-miR-2342	-19.1	357
bta-miR-2342	-18.7	408
bta-miR-2342	-18.4	241
bta-miR-2342	-18.0	668
bta-miR-2342	-17.9	620
bta-miR-2342	-17.7	114
bta-miR-2342	-17.5	153
bta-miR-2342	-17.5	697
bta-miR-2342	-16.9	452
bta-miR-2342	-16.7	71
bta-miR-2342	-16.4	331
bta-miR-2342	-15.7	307
bta-miR-2342	-15.6	517
bta-miR-2342	-15.3	7
bta-miR-2342	-14.9	395
bta-miR-2342	-14.3	41
bta-miR-2342	-14.1	464
bta-miR-2342	-14.0	271
bta-miR-2342	-13.7	86
bta-miR-2342	-12.9	283
bta-miR-2342	-11.2	738
bta-miR-2342	-10.5	30
bta-miR-2342	-10.2	712

bta-miR-2342	-10.0	226
bta-miR-2343	-25.3	113
bta-miR-2343	-24.3	555
bta-miR-2343	-23.7	316
bta-miR-2343	-23.2	233
bta-miR-2343	-22.7	464
bta-miR-2343	-22.3	389
bta-miR-2343	-20.4	411
bta-miR-2343	-19.5	151
bta-miR-2343	-19.1	78
bta-miR-2343	-19.0	174
bta-miR-2343	-18.2	663
bta-miR-2343	-18.0	355
bta-miR-2343	-17.9	139
bta-miR-2343	-17.7	15
bta-miR-2343	-16.8	252
bta-miR-2343	-16.7	36
bta-miR-2343	-16.7	202
bta-miR-2343	-16.4	618
bta-miR-2343	-16.2	505
bta-miR-2343	-15.8	281
bta-miR-2343	-14.7	1
bta-miR-2343	-14.6	527
bta-miR-2343	-14.1	341
bta-miR-2343	-13.5	687
bta-miR-2343	-11.8	594
bta-miR-2343	-10.9	452
bta-miR-2343	-10.6	739
bta-miR-2343	-10.5	375
bta-miR-2343	-10.5	651
bta-miR-2343	-10.2	266
bta-miR-2344	-23.9	36
bta-miR-2344	-23.2	559
bta-miR-2344	-22.6	274
bta-miR-2344	-21.9	635
bta-miR-2344	-21.8	4
bta-miR-2344	-21.0	475
bta-miR-2344	-20.6	125
bta-miR-2344	-19.9	504
bta-miR-2344	-19.3	419
bta-miR-2344	-18.1	397
bta-miR-2344	-18.0	108
bta-miR-2344	-17.6	352
bta-miR-2344	-16.9	238
bta-miR-2344	-16.8	451
bta-miR-2344	-16.2	689
bta-miR-2344	-15.6	188
bta-miR-2344	-15.3	663
bta-miR-2344	-15.1	72
bta-miR-2344	-15.0	738
bta-miR-2344	-13.4	310
bta-miR-2344	-13.0	587
bta-miR-2344	-12.8	530
bta-miR-2344	-12.7	164
bta-miR-2344	-12.5	258
bta-miR-2344	-11.8	382
bta-miR-2344	-11.0	211
bta-miR-2344	-11.0	328
bta-miR-2344	-10.9	23

bta-miR-2344	-10.6	342
bta-miR-2344	-10.5	710
bta-miR-2345	-17.1	151
bta-miR-2345	-16.6	598
bta-miR-2345	-16.1	94
bta-miR-2345	-14.9	352
bta-miR-2345	-14.2	196
bta-miR-2345	-13.6	225
bta-miR-2345	-13.6	406
bta-miR-2345	-13.4	265
bta-miR-2345	-13.3	176
bta-miR-2345	-13.3	375
bta-miR-2345	-13.3	478
bta-miR-2345	-12.6	439
bta-miR-2345	-12.0	727
bta-miR-2345	-11.5	615
bta-miR-2345	-11.3	574
bta-miR-2345	-10.8	499
bta-miR-2345	-10.6	119
bta-miR-2345	-10.5	65
bta-miR-2345	-10.0	41
bta-miR-2346	-26.0	126
bta-miR-2346	-21.9	44
bta-miR-2346	-21.2	237
bta-miR-2346	-21.0	361
bta-miR-2346	-20.3	473
bta-miR-2346	-19.5	420
bta-miR-2346	-19.5	563
bta-miR-2346	-18.6	156
bta-miR-2346	-18.4	308
bta-miR-2346	-18.2	8
bta-miR-2346	-17.9	199
bta-miR-2346	-17.1	81
bta-miR-2346	-17.1	109
bta-miR-2346	-16.1	337
bta-miR-2346	-15.8	270
bta-miR-2346	-15.6	499
bta-miR-2346	-15.2	395
bta-miR-2346	-14.3	455
bta-miR-2346	-13.8	28
bta-miR-2346	-13.3	669
bta-miR-2346	-12.8	624
bta-miR-2346	-12.3	698
bta-miR-2346	-12.3	185
bta-miR-2346	-11.9	67
bta-miR-2346	-11.5	520
bta-miR-2346	-10.1	602
bta-miR-2346	-10.0	737
bta-miR-2347	-22.1	465
bta-miR-2347	-21.2	181
bta-miR-2347	-21.0	107
bta-miR-2347	-20.7	50
bta-miR-2347	-20.5	664
bta-miR-2347	-19.9	243
bta-miR-2347	-19.8	567
bta-miR-2347	-19.4	623
bta-miR-2347	-19.4	542
bta-miR-2347	-19.3	422
bta-miR-2347	-18.4	389

bta-miR-2347	-17.6	81
bta-miR-2347	-17.3	205
bta-miR-2347	-17.2	499
bta-miR-2347	-17.1	9
bta-miR-2347	-15.0	282
bta-miR-2347	-14.9	523
bta-miR-2347	-14.3	361
bta-miR-2347	-14.0	125
bta-miR-2347	-13.3	165
bta-miR-2347	-13.1	707
bta-miR-2347	-12.6	27
bta-miR-2347	-12.0	65
bta-miR-2347	-11.6	653
bta-miR-2347	-11.5	737
bta-miR-2347	-10.7	170
bta-miR-2347	-10.5	456
bta-miR-2347	-10.1	228
bta-miR-2347	-10.1	596
bta-miR-2348	-28.6	115
bta-miR-2348	-28.0	414
bta-miR-2348	-27.2	308
bta-miR-2348	-26.5	85
bta-miR-2348	-25.2	560
bta-miR-2348	-23.9	387
bta-miR-2348	-23.8	185
bta-miR-2348	-23.6	155
bta-miR-2348	-22.1	457
bta-miR-2348	-21.8	12
bta-miR-2348	-21.4	342
bta-miR-2348	-19.7	482
bta-miR-2348	-19.5	232
bta-miR-2348	-18.6	38
bta-miR-2348	-17.8	620
bta-miR-2348	-17.1	586
bta-miR-2348	-16.5	696
bta-miR-2348	-16.3	137
bta-miR-2348	-16.3	258
bta-miR-2348	-15.3	661
bta-miR-2348	-14.5	283
bta-miR-2348	-14.4	742
bta-miR-2348	-13.1	106
bta-miR-2348	-13.1	61
bta-miR-2348	-11.8	519
bta-miR-2348	-10.8	548
bta-miR-2348	-10.6	643
bta-miR-2348	-10.5	377
bta-miR-2348	-10.2	148
bta-miR-2349	-28.6	344
bta-miR-2349	-27.2	1
bta-miR-2349	-25.5	433
bta-miR-2349	-25.3	82
bta-miR-2349	-24.1	616
bta-miR-2349	-24.1	204
bta-miR-2349	-22.9	566
bta-miR-2349	-22.8	403
bta-miR-2349	-22.3	498
bta-miR-2349	-21.3	25
bta-miR-2349	-20.4	464
bta-miR-2349	-19.8	60

bta-miR-2349	-19.6	365
bta-miR-2349	-18.8	588
bta-miR-2349	-18.2	730
bta-miR-2349	-17.1	258
bta-miR-2349	-16.6	659
bta-miR-2349	-15.7	126
bta-miR-2349	-14.1	166
bta-miR-2349	-13.5	291
bta-miR-2349	-12.9	541
bta-miR-2349	-12.6	386
bta-miR-2349	-12.0	150
bta-miR-2349	-11.3	51
bta-miR-2349	-10.9	684
bta-miR-2284p	-19.4	258
bta-miR-2284p	-17.8	368
bta-miR-2284p	-17.7	59
bta-miR-2284p	-16.2	626
bta-miR-2284p	-16.0	401
bta-miR-2284p	-16.0	596
bta-miR-2284p	-15.9	232
bta-miR-2284p	-15.8	499
bta-miR-2284p	-15.6	89
bta-miR-2284p	-14.7	111
bta-miR-2284p	-14.2	168
bta-miR-2284p	-14.0	191
bta-miR-2284p	-13.9	580
bta-miR-2284p	-13.6	5
bta-miR-2284p	-13.5	476
bta-miR-2284p	-13.1	711
bta-miR-2284p	-12.9	451
bta-miR-2284p	-12.9	689
bta-miR-2284p	-12.8	330
bta-miR-2284p	-12.5	310
bta-miR-2284p	-12.0	281
bta-miR-2284p	-12.0	421
bta-miR-2284p	-12.0	431
bta-miR-2284p	-11.9	31
bta-miR-2284p	-11.9	567
bta-miR-2284p	-11.1	462
bta-miR-2284p	-11.1	216
bta-miR-2284p	-11.1	663
bta-miR-2284p	-10.6	552
bta-miR-2284p	-10.3	357
bta-miR-2284p	-10.2	163
bta-miR-2284p	-10.0	324
bta-miR-2284p	-10.0	641
bta-miR-2350	-34.7	216
bta-miR-2350	-32.2	438
bta-miR-2350	-26.2	609
bta-miR-2350	-25.5	267
bta-miR-2350	-25.5	61
bta-miR-2350	-21.5	337
bta-miR-2350	-21.3	174
bta-miR-2350	-20.6	92
bta-miR-2350	-20.3	403
bta-miR-2350	-20.0	587
bta-miR-2350	-18.7	6
bta-miR-2350	-18.6	477
bta-miR-2350	-16.5	497

bta-miR-2350	-15.3	629
bta-miR-2350	-14.1	730
bta-miR-2350	-13.5	238
bta-miR-2350	-12.8	364
bta-miR-2350	-11.2	561
bta-miR-2350	-10.9	154
bta-miR-2351	-16.3	403
bta-miR-2351	-15.4	508
bta-miR-2351	-15.2	486
bta-miR-2351	-14.1	351
bta-miR-2351	-13.7	371
bta-miR-2351	-13.1	11
bta-miR-2351	-12.9	620
bta-miR-2351	-12.5	121
bta-miR-2351	-12.3	149
bta-miR-2351	-11.9	172
bta-miR-2351	-11.8	49
bta-miR-2351	-11.7	197
bta-miR-2351	-11.3	268
bta-miR-2351	-11.0	590
bta-miR-2351	-10.8	300
bta-miR-2351	-10.6	432
bta-miR-2351	-10.5	737
bta-miR-2351	-10.4	100
bta-miR-2351	-10.1	69
bta-miR-2352	-19.9	85
bta-miR-2352	-19.9	205
bta-miR-2352	-19.7	495
bta-miR-2352	-19.5	381
bta-miR-2352	-18.9	456
bta-miR-2352	-17.9	243
bta-miR-2352	-17.6	107
bta-miR-2352	-17.6	419
bta-miR-2352	-17.5	50
bta-miR-2352	-17.5	270
bta-miR-2352	-16.6	623
bta-miR-2352	-16.4	576
bta-miR-2352	-14.8	653
bta-miR-2352	-14.2	559
bta-miR-2352	-14.1	527
bta-miR-2352	-14.0	170
bta-miR-2352	-13.9	401
bta-miR-2352	-13.8	358
bta-miR-2352	-13.4	2
bta-miR-2352	-13.3	16
bta-miR-2352	-13.0	669
bta-miR-2352	-12.8	687
bta-miR-2352	-11.8	232
bta-miR-2352	-11.0	71
bta-miR-2352	-11.0	472
bta-miR-2352	-11.0	715
bta-miR-2352	-10.3	605
bta-miR-2353	-30.4	572
bta-miR-2353	-25.8	52
bta-miR-2353	-24.2	462
bta-miR-2353	-23.9	515
bta-miR-2353	-23.8	392
bta-miR-2353	-23.6	87
bta-miR-2353	-23.2	2

bta-miR-2353	-21.5	545
bta-miR-2353	-20.8	200
bta-miR-2353	-20.0	237
bta-miR-2353	-19.7	136
bta-miR-2353	-18.5	639
bta-miR-2353	-18.5	607
bta-miR-2353	-17.8	681
bta-miR-2353	-17.1	426
bta-miR-2353	-16.2	164
bta-miR-2353	-16.0	358
bta-miR-2353	-15.7	277
bta-miR-2353	-15.1	715
bta-miR-2353	-14.7	19
bta-miR-2353	-11.0	309
bta-miR-2353	-10.7	191
bta-miR-2353	-10.4	626
bta-miR-2353	-10.3	127
bta-miR-2353	-10.1	386
bta-miR-2353	-10.1	38
bta-miR-2354	-21.8	415
bta-miR-2354	-19.2	369
bta-miR-2354	-18.3	92
bta-miR-2354	-16.7	197
bta-miR-2354	-16.4	351
bta-miR-2354	-16.0	730
bta-miR-2354	-16.0	156
bta-miR-2354	-15.5	457
bta-miR-2354	-14.8	508
bta-miR-2354	-14.7	649
bta-miR-2354	-14.4	13
bta-miR-2354	-14.4	568
bta-miR-2354	-12.8	124
bta-miR-2354	-12.6	269
bta-miR-2354	-12.4	239
bta-miR-2354	-12.3	175
bta-miR-2354	-12.2	483
bta-miR-2354	-12.2	49
bta-miR-2354	-11.8	301
bta-miR-2354	-11.7	313
bta-miR-2354	-11.5	698
bta-miR-2354	-11.1	600
bta-miR-2354	-10.9	68
bta-miR-2354	-10.6	617
bta-miR-2354	-10.3	334
bta-miR-2354	-10.2	559
bta-miR-2355*	-22.3	525
bta-miR-2355*	-22.1	1
bta-miR-2355*	-20.6	96
bta-miR-2355*	-19.3	457
bta-miR-2355*	-19.1	544
bta-miR-2355*	-18.7	404
bta-miR-2355*	-18.5	488
bta-miR-2355*	-18.4	230
bta-miR-2355*	-18.2	22
bta-miR-2355*	-15.7	199
bta-miR-2355*	-15.7	577
bta-miR-2355*	-15.6	643
bta-miR-2355*	-14.5	354
bta-miR-2355*	-14.3	606

bta-miR-2355*	-14.2	696
bta-miR-2355*	-13.1	381
bta-miR-2355*	-12.8	428
bta-miR-2355*	-12.5	421
bta-miR-2355*	-12.2	164
bta-miR-2355*	-12.2	667
bta-miR-2355*	-12.1	88
bta-miR-2355*	-11.5	78
bta-miR-2355*	-10.7	286
bta-miR-2355*	-10.3	143
bta-miR-2355*	-10.1	514
bta-miR-2355	-24.3	95
bta-miR-2355	-23.5	577
bta-miR-2355	-22.6	164
bta-miR-2355	-22.4	256
bta-miR-2355	-21.5	58
bta-miR-2355	-21.4	484
bta-miR-2355	-20.0	190
bta-miR-2355	-19.9	395
bta-miR-2355	-18.2	613
bta-miR-2355	-17.4	236
bta-miR-2355	-17.4	466
bta-miR-2355	-16.8	445
bta-miR-2355	-16.7	363
bta-miR-2355	-16.4	4
bta-miR-2355	-16.0	139
bta-miR-2355	-15.1	337
bta-miR-2355	-14.3	74
bta-miR-2355	-14.1	639
bta-miR-2355	-14.0	117
bta-miR-2355	-13.4	692
bta-miR-2355	-13.4	625
bta-miR-2355	-12.9	513
bta-miR-2355	-12.7	34
bta-miR-2355	-11.3	276
bta-miR-2355	-11.2	713
bta-miR-2355	-10.4	502
bta-miR-2355	-10.2	316
bta-miR-2355	-10.1	551
bta-miR-2356	-28.8	128
bta-miR-2356	-24.5	276
bta-miR-2356	-23.6	192
bta-miR-2356	-21.8	100
bta-miR-2356	-20.7	421
bta-miR-2356	-20.5	237
bta-miR-2356	-19.8	707
bta-miR-2356	-19.4	474
bta-miR-2356	-19.3	39
bta-miR-2356	-19.2	572
bta-miR-2356	-18.8	259
bta-miR-2356	-18.7	309
bta-miR-2356	-18.3	625
bta-miR-2356	-17.8	351
bta-miR-2356	-17.8	9
bta-miR-2356	-17.7	163
bta-miR-2356	-17.6	502
bta-miR-2356	-16.6	82
bta-miR-2356	-16.0	390
bta-miR-2356	-15.1	58

bta-miR-2356	-14.6	542
bta-miR-2356	-13.8	376
bta-miR-2356	-12.8	596
bta-miR-2356	-12.2	71
bta-miR-2356	-12.2	454
bta-miR-2356	-11.6	692
bta-miR-2356	-11.4	329
bta-miR-2356	-11.3	521
bta-miR-2356	-10.5	675
bta-miR-2356	-10.4	740
bta-miR-2356	-10.0	1
bta-miR-2357	-25.4	353
bta-miR-2357	-25.1	147
bta-miR-2357	-24.1	106
bta-miR-2357	-23.8	382
bta-miR-2357	-22.5	540
bta-miR-2357	-22.3	55
bta-miR-2357	-22.3	514
bta-miR-2357	-21.6	461
bta-miR-2357	-20.6	6
bta-miR-2357	-20.6	86
bta-miR-2357	-19.4	654
bta-miR-2357	-19.3	577
bta-miR-2357	-18.5	276
bta-miR-2357	-17.5	420
bta-miR-2357	-16.3	624
bta-miR-2357	-15.9	38
bta-miR-2357	-15.8	206
bta-miR-2357	-15.6	256
bta-miR-2357	-15.2	489
bta-miR-2357	-15.2	428
bta-miR-2357	-14.6	681
bta-miR-2357	-13.6	235
bta-miR-2357	-12.4	172
bta-miR-2357	-12.3	740
bta-miR-2357	-11.8	599
bta-miR-2357	-10.8	130
bta-miR-2357	-10.8	244
bta-miR-2357	-10.6	638
bta-miR-2357	-10.6	369
bta-miR-2357	-10.4	325
bta-miR-2284u	-19.6	442
bta-miR-2284u	-19.4	220
bta-miR-2284u	-16.5	101
bta-miR-2284u	-16.4	338
bta-miR-2284u	-16.2	405
bta-miR-2284u	-15.7	11
bta-miR-2284u	-15.3	592
bta-miR-2284u	-15.2	166
bta-miR-2284u	-14.7	269
bta-miR-2284u	-14.6	47
bta-miR-2284u	-14.4	177
bta-miR-2284u	-14.3	608
bta-miR-2284u	-14.2	735
bta-miR-2284u	-13.7	506
bta-miR-2284u	-13.5	80
bta-miR-2284u	-13.0	470
bta-miR-2284u	-12.9	357
bta-miR-2284u	-12.6	308

bta-miR-2284u	-12.3	629
bta-miR-2284u	-12.0	563
bta-miR-2284u	-11.6	124
bta-miR-2284u	-11.1	240
bta-miR-2284u	-10.9	24
bta-miR-2358	-20.3	401
bta-miR-2358	-20.2	161
bta-miR-2358	-20.0	461
bta-miR-2358	-19.4	317
bta-miR-2358	-19.1	354
bta-miR-2358	-19.1	572
bta-miR-2358	-18.3	692
bta-miR-2358	-18.0	502
bta-miR-2358	-17.9	236
bta-miR-2358	-17.2	127
bta-miR-2358	-17.1	14
bta-miR-2358	-16.1	90
bta-miR-2358	-15.2	626
bta-miR-2358	-14.4	277
bta-miR-2358	-13.7	533
bta-miR-2358	-13.1	206
bta-miR-2358	-12.9	55
bta-miR-2358	-12.9	480
bta-miR-2358	-12.9	590
bta-miR-2358	-12.8	143
bta-miR-2358	-12.3	426
bta-miR-2358	-11.9	662
bta-miR-2358	-11.8	729
bta-miR-2358	-11.7	178
bta-miR-2358	-11.5	561
bta-miR-2358	-11.4	382
bta-miR-2358	-11.0	188
bta-miR-2358	-10.9	120
bta-miR-2358	-10.1	646
bta-miR-2358	-10.1	309
bta-miR-2358	-10.0	711
bta-miR-2359	-17.5	218
bta-miR-2359	-16.3	101
bta-miR-2359	-16.1	174
bta-miR-2359	-15.8	445
bta-miR-2359	-15.6	610
bta-miR-2359	-13.1	196
bta-miR-2359	-13.1	479
bta-miR-2359	-12.3	366
bta-miR-2359	-12.1	598
bta-miR-2359	-11.8	348
bta-miR-2359	-11.3	153
bta-miR-2359	-10.9	506
bta-miR-2359	-10.8	725
bta-miR-2359	-10.8	68
bta-miR-2359	-10.7	239
bta-miR-2359	-10.1	12
bta-miR-2359	-10.1	268
bta-miR-2360	-32.5	412
bta-miR-2360	-30.4	463
bta-miR-2360	-30.3	562
bta-miR-2360	-27.2	118
bta-miR-2360	-26.9	380
bta-miR-2360	-24.8	306

bta-miR-2360	-24.2	509
bta-miR-2360	-23.8	70
bta-miR-2360	-23.5	230
bta-miR-2360	-22.7	13
bta-miR-2360	-22.2	153
bta-miR-2360	-21.6	42
bta-miR-2360	-20.7	270
bta-miR-2360	-20.4	343
bta-miR-2360	-20.0	184
bta-miR-2360	-19.4	654
bta-miR-2360	-18.5	621
bta-miR-2360	-18.1	538
bta-miR-2360	-17.4	681
bta-miR-2360	-17.2	487
bta-miR-2360	-15.8	728
bta-miR-2360	-13.7	589
bta-miR-2360	-13.3	701
bta-miR-2360	-12.4	5
bta-miR-2360	-12.2	261
bta-miR-2360	-10.0	407
bta-miR-2361	-17.5	606
bta-miR-2361	-16.4	172
bta-miR-2361	-15.2	348
bta-miR-2361	-14.4	101
bta-miR-2361	-13.2	149
bta-miR-2361	-12.7	223
bta-miR-2361	-12.3	405
bta-miR-2361	-12.3	440
bta-miR-2361	-12.1	371
bta-miR-2361	-11.9	189
bta-miR-2361	-11.8	723
bta-miR-2361	-11.4	271
bta-miR-2361	-11.2	469
bta-miR-2361	-10.7	47
bta-miR-2361	-10.3	122
bta-miR-2361	-10.1	332
bta-miR-2362	-22.9	449
bta-miR-2362	-22.6	200
bta-miR-2362	-21.6	617
bta-miR-2362	-21.4	235
bta-miR-2362	-20.8	67
bta-miR-2362	-20.6	277
bta-miR-2362	-20.5	505
bta-miR-2362	-20.1	89
bta-miR-2362	-19.5	44
bta-miR-2362	-19.3	576
bta-miR-2362	-18.8	551
bta-miR-2362	-18.3	128
bta-miR-2362	-18.1	332
bta-miR-2362	-17.6	482
bta-miR-2362	-17.4	8
bta-miR-2362	-16.9	395
bta-miR-2362	-16.4	414
bta-miR-2362	-14.7	354
bta-miR-2362	-13.0	174
bta-miR-2362	-12.7	116
bta-miR-2362	-12.5	311
bta-miR-2362	-12.3	28
bta-miR-2362	-12.3	153

bta-miR-2362	-12.3	692
bta-miR-2362	-11.8	659
bta-miR-2362	-11.3	380
bta-miR-2362	-11.0	737
bta-miR-2362	-10.2	220
bta-miR-2362	-10.2	531
bta-miR-2362	-10.1	605
bta-miR-2363	-19.9	16
bta-miR-2363	-19.6	564
bta-miR-2363	-19.3	422
bta-miR-2363	-18.1	476
bta-miR-2363	-17.5	400
bta-miR-2363	-16.5	357
bta-miR-2363	-15.8	209
bta-miR-2363	-15.5	79
bta-miR-2363	-14.7	580
bta-miR-2363	-14.5	690
bta-miR-2363	-13.4	496
bta-miR-2363	-13.4	521
bta-miR-2363	-13.2	303
bta-miR-2363	-13.1	714
bta-miR-2363	-12.9	248
bta-miR-2363	-12.9	659
bta-miR-2363	-12.7	111
bta-miR-2363	-12.7	535
bta-miR-2363	-12.5	626
bta-miR-2363	-12.3	191
bta-miR-2363	-12.1	229
bta-miR-2363	-12.0	59
bta-miR-2363	-11.8	145
bta-miR-2363	-11.7	325
bta-miR-2363	-11.3	443
bta-miR-2363	-11.1	461
bta-miR-2363	-10.8	372
bta-miR-2363	-10.8	120
bta-miR-2363	-10.6	605
bta-miR-2363	-10.3	163
bta-miR-2363	-10.3	341
bta-miR-2363	-10.1	378
bta-miR-2364	-27.7	427
bta-miR-2364	-27.4	132
bta-miR-2364	-26.8	185
bta-miR-2364	-26.1	30
bta-miR-2364	-25.0	87
bta-miR-2364	-24.8	109
bta-miR-2364	-24.6	378
bta-miR-2364	-23.4	244
bta-miR-2364	-23.4	473
bta-miR-2364	-23.2	156
bta-miR-2364	-23.1	206
bta-miR-2364	-22.9	582
bta-miR-2364	-22.3	324
bta-miR-2364	-20.0	360
bta-miR-2364	-19.2	561
bta-miR-2364	-19.0	395
bta-miR-2364	-18.6	307
bta-miR-2364	-18.4	457
bta-miR-2364	-17.6	502
bta-miR-2364	-16.1	3

bta-miR-2364	-14.0	668
bta-miR-2364	-13.3	697
bta-miR-2364	-12.8	235
bta-miR-2364	-12.7	271
bta-miR-2364	-12.5	626
bta-miR-2364	-12.4	283
bta-miR-2364	-12.0	524
bta-miR-2364	-11.4	644
bta-miR-2364	-10.9	20
bta-miR-2364	-10.5	740
bta-miR-2364	-10.3	447
bta-miR-2364	-10.3	126
bta-miR-2365	-20.1	443
bta-miR-2365	-19.8	592
bta-miR-2365	-17.8	94
bta-miR-2365	-15.7	175
bta-miR-2365	-15.7	347
bta-miR-2365	-15.3	266
bta-miR-2365	-15.2	479
bta-miR-2365	-14.6	36
bta-miR-2365	-14.4	612
bta-miR-2365	-14.2	405
bta-miR-2365	-13.8	227
bta-miR-2365	-13.4	198
bta-miR-2365	-13.1	68
bta-miR-2365	-11.6	505
bta-miR-2365	-11.4	728
bta-miR-2365	-11.4	15
bta-miR-2365	-11.2	653
bta-miR-2365	-10.7	151
bta-miR-2366	-26.9	200
bta-miR-2366	-25.2	515
bta-miR-2366	-25.0	622
bta-miR-2366	-23.7	462
bta-miR-2366	-23.0	9
bta-miR-2366	-22.5	363
bta-miR-2366	-22.3	127
bta-miR-2366	-22.3	567
bta-miR-2366	-21.9	409
bta-miR-2366	-20.4	84
bta-miR-2366	-20.1	241
bta-miR-2366	-19.9	280
bta-miR-2366	-19.9	164
bta-miR-2366	-19.0	51
bta-miR-2366	-18.0	107
bta-miR-2366	-17.9	670
bta-miR-2366	-16.4	392
bta-miR-2366	-15.6	325
bta-miR-2366	-15.3	710
bta-miR-2366	-14.7	639
bta-miR-2366	-14.6	499
bta-miR-2366	-14.5	429
bta-miR-2366	-12.7	71
bta-miR-2366	-12.4	654
bta-miR-2366	-11.8	269
bta-miR-2366	-11.7	603
bta-miR-2366	-11.7	693
bta-miR-2366	-11.5	39
bta-miR-2366	-10.7	1

bta-miR-2366	-10.4	311
bta-miR-2366	-10.4	588
bta-miR-2366	-10.3	154
bta-miR-2366	-10.3	351
bta-miR-2366	-10.2	386
bta-miR-2284f	-16.7	443
bta-miR-2284f	-15.1	173
bta-miR-2284f	-14.9	232
bta-miR-2284f	-13.9	258
bta-miR-2284f	-13.7	60
bta-miR-2284f	-13.6	97
bta-miR-2284f	-13.4	416
bta-miR-2284f	-13.4	596
bta-miR-2284f	-13.0	14
bta-miR-2284f	-12.8	499
bta-miR-2284f	-12.7	620
bta-miR-2284f	-12.6	338
bta-miR-2284f	-12.3	460
bta-miR-2284f	-12.1	215
bta-miR-2284f	-11.9	369
bta-miR-2284f	-11.5	77
bta-miR-2284f	-11.1	309
bta-miR-2284f	-11.0	566
bta-miR-2284f	-11.0	689
bta-miR-2284f	-10.9	202
bta-miR-2284f	-10.8	276
bta-miR-2284f	-10.7	574
bta-miR-2284f	-10.5	724
bta-miR-2284a	-19.7	168
bta-miR-2284a	-18.9	357
bta-miR-2284a	-18.3	47
bta-miR-2284a	-17.6	470
bta-miR-2284a	-17.4	240
bta-miR-2284a	-17.3	327
bta-miR-2284a	-17.2	28
bta-miR-2284a	-16.6	108
bta-miR-2284a	-16.5	260
bta-miR-2284a	-16.5	506
bta-miR-2284a	-16.4	405
bta-miR-2284a	-15.4	273
bta-miR-2284a	-15.1	429
bta-miR-2284a	-15.0	188
bta-miR-2284a	-14.4	141
bta-miR-2284a	-14.1	306
bta-miR-2284a	-14.0	129
bta-miR-2284a	-14.0	620
bta-miR-2284a	-13.3	320
bta-miR-2284a	-13.0	90
bta-miR-2284a	-12.9	689
bta-miR-2284a	-12.8	592
bta-miR-2284a	-12.7	710
bta-miR-2284a	-12.1	442
bta-miR-2284a	-12.0	557
bta-miR-2284a	-11.8	159
bta-miR-2284a	-11.4	81
bta-miR-2284a	-11.2	574
bta-miR-2284a	-11.0	208
bta-miR-2284a	-10.2	340
bta-miR-2284a	-10.1	223

bta-miR-2284a	-10.1	668
bta-miR-2284k	-22.8	405
bta-miR-2284k	-21.5	357
bta-miR-2284k	-17.9	470
bta-miR-2284k	-17.8	162
bta-miR-2284k	-17.5	506
bta-miR-2284k	-17.4	47
bta-miR-2284k	-16.4	102
bta-miR-2284k	-16.4	423
bta-miR-2284k	-15.7	567
bta-miR-2284k	-15.7	731
bta-miR-2284k	-15.3	28
bta-miR-2284k	-15.2	240
bta-miR-2284k	-15.0	453
bta-miR-2284k	-14.7	124
bta-miR-2284k	-14.5	574
bta-miR-2284k	-14.4	620
bta-miR-2284k	-14.3	273
bta-miR-2284k	-14.0	186
bta-miR-2284k	-13.9	9
bta-miR-2284k	-13.6	596
bta-miR-2284k	-13.5	306
bta-miR-2284k	-13.4	83
bta-miR-2284k	-13.2	338
bta-miR-2284k	-13.0	323
bta-miR-2284k	-12.7	652
bta-miR-2284k	-12.2	173
bta-miR-2284k	-12.2	222
bta-miR-2284k	-12.0	701
bta-miR-2284k	-11.6	208
bta-miR-2284k	-11.1	541
bta-miR-2284k	-10.6	557
bta-miR-2284k	-10.5	385
bta-miR-2284k	-10.3	490
bta-miR-2284k	-10.2	142
bta-miR-1814b	-17.1	371
bta-miR-1814b	-16.9	485
bta-miR-1814b	-14.0	556
bta-miR-1814b	-13.9	197
bta-miR-1814b	-13.7	104
bta-miR-1814b	-13.7	648
bta-miR-1814b	-13.3	406
bta-miR-1814b	-13.3	454
bta-miR-1814b	-13.0	148
bta-miR-1814b	-12.8	269
bta-miR-1814b	-12.6	731
bta-miR-1814b	-12.3	332
bta-miR-1814b	-12.1	178
bta-miR-1814b	-12.1	603
bta-miR-1814b	-12.0	123
bta-miR-1814b	-10.9	65
bta-miR-1814b	-10.1	586
bta-miR-1814b	-10.1	218
bta-miR-1814b	-10.0	35
bta-miR-2367	-26.7	475
bta-miR-2367	-26.3	5
bta-miR-2367	-26.0	416
bta-miR-2367	-25.1	567
bta-miR-2367	-21.6	614

bta-miR-2367	-20.5	60
bta-miR-2367	-18.2	345
bta-miR-2367	-18.0	215
bta-miR-2367	-17.1	262
bta-miR-2367	-16.3	92
bta-miR-2367	-15.1	155
bta-miR-2367	-14.8	450
bta-miR-2367	-14.1	740
bta-miR-2367	-13.4	657
bta-miR-2367	-13.2	185
bta-miR-2367	-12.5	541
bta-miR-2367	-12.1	26
bta-miR-2367	-11.8	692
bta-miR-2367	-10.7	403
bta-miR-2367	-10.3	502
bta-miR-2367	-10.1	386
bta-miR-2367*	-20.4	399
bta-miR-2367*	-19.9	59
bta-miR-2367*	-19.8	512
bta-miR-2367*	-18.1	368
bta-miR-2367*	-17.5	111
bta-miR-2367*	-17.2	626
bta-miR-2367*	-17.2	356
bta-miR-2367*	-16.9	474
bta-miR-2367*	-16.5	261
bta-miR-2367*	-16.0	552
bta-miR-2367*	-15.9	580
bta-miR-2367*	-15.6	657
bta-miR-2367*	-15.5	90
bta-miR-2367*	-15.3	5
bta-miR-2367*	-15.2	33
bta-miR-2367*	-14.5	289
bta-miR-2367*	-14.5	421
bta-miR-2367*	-14.0	236
bta-miR-2367*	-14.0	680
bta-miR-2367*	-13.1	146
bta-miR-2367*	-13.0	81
bta-miR-2367*	-13.0	537
bta-miR-2367*	-12.4	740
bta-miR-2367*	-12.0	15
bta-miR-2367*	-11.7	431
bta-miR-2367*	-11.6	461
bta-miR-2367*	-11.6	191
bta-miR-2367*	-11.4	386
bta-miR-2367*	-11.4	714
bta-miR-2367*	-11.2	167
bta-miR-2367*	-10.7	25
bta-miR-2367*	-10.6	491
bta-miR-2367*	-10.5	51
bta-miR-2367*	-10.5	126
bta-miR-2367*	-10.2	249
bta-miR-2367*	-10.2	566
bta-miR-2368	-28.0	410
bta-miR-2368	-25.5	228
bta-miR-2368	-23.4	100
bta-miR-2368	-22.2	454
bta-miR-2368	-21.4	592
bta-miR-2368	-21.3	571
bta-miR-2368	-21.2	67

bta-miR-2368	-20.8	149
bta-miR-2368	-20.8	499
bta-miR-2368	-20.6	9
bta-miR-2368	-20.4	306
bta-miR-2368	-20.2	126
bta-miR-2368	-19.9	198
bta-miR-2368	-19.7	41
bta-miR-2368	-19.4	351
bta-miR-2368	-18.9	620
bta-miR-2368	-18.1	553
bta-miR-2368	-16.9	264
bta-miR-2368	-16.7	481
bta-miR-2368	-16.4	165
bta-miR-2368	-16.4	323
bta-miR-2368	-14.9	653
bta-miR-2368	-14.8	376
bta-miR-2368	-14.7	739
bta-miR-2368	-14.6	278
bta-miR-2368	-14.5	705
bta-miR-2368	-13.7	515
bta-miR-2368	-10.8	607
bta-miR-2368	-10.7	442
bta-miR-2368	-10.4	139
bta-miR-2368	-10.4	338
bta-miR-2368*	-24.8	605
bta-miR-2368*	-22.0	66
bta-miR-2368*	-21.5	12
bta-miR-2368*	-20.4	494
bta-miR-2368*	-19.8	414
bta-miR-2368*	-19.8	448
bta-miR-2368*	-19.1	172
bta-miR-2368*	-18.5	654
bta-miR-2368*	-18.1	199
bta-miR-2368*	-17.5	555
bta-miR-2368*	-16.0	349
bta-miR-2368*	-15.3	264
bta-miR-2368*	-14.3	107
bta-miR-2368*	-14.3	630
bta-miR-2368*	-13.1	228
bta-miR-2368*	-12.6	376
bta-miR-2368*	-12.2	37
bta-miR-2368*	-11.4	737
bta-miR-2368*	-11.2	584
bta-miR-2368*	-10.3	4
bta-miR-2368*	-10.1	155
bta-miR-2369	-21.8	104
bta-miR-2369	-21.7	351
bta-miR-2369	-19.8	405
bta-miR-2369	-19.2	22
bta-miR-2369	-17.5	46
bta-miR-2369	-17.4	154
bta-miR-2369	-17.3	371
bta-miR-2369	-17.1	269
bta-miR-2369	-16.8	124
bta-miR-2369	-16.8	731
bta-miR-2369	-16.6	494
bta-miR-2369	-16.2	197
bta-miR-2369	-16.1	470
bta-miR-2369	-16.0	240

bta-miR-2369	-15.6	324
bta-miR-2369	-15.5	558
bta-miR-2369	-14.2	589
bta-miR-2369	-14.2	178
bta-miR-2369	-13.3	448
bta-miR-2369	-13.3	514
bta-miR-2369	-13.3	616
bta-miR-2369	-13.2	301
bta-miR-2369	-13.0	652
bta-miR-2369	-12.5	69
bta-miR-2369	-11.7	430
bta-miR-2369	-11.7	695
bta-miR-2369	-11.3	6
bta-miR-2369	-11.0	145
bta-miR-2369	-10.6	338
bta-miR-2369	-10.4	260
bta-miR-2369	-10.0	574
bta-miR-2370	-18.6	155
bta-miR-2370	-18.5	669
bta-miR-2370	-17.4	698
bta-miR-2370	-17.0	243
bta-miR-2370	-16.7	18
bta-miR-2370	-16.6	569
bta-miR-2370	-16.5	125
bta-miR-2370	-16.4	63
bta-miR-2370	-16.2	407
bta-miR-2370	-15.8	633
bta-miR-2370	-14.9	183
bta-miR-2370	-14.3	340
bta-miR-2370	-14.3	377
bta-miR-2370	-14.3	482
bta-miR-2370	-14.2	95
bta-miR-2370	-13.9	456
bta-miR-2370	-13.4	304
bta-miR-2370	-12.3	282
bta-miR-2370	-12.3	50
bta-miR-2370	-11.6	509
bta-miR-2370	-11.1	365
bta-miR-2370	-10.9	330
bta-miR-2370	-10.4	732
bta-miR-2370	-10.4	524
bta-miR-2370*	-27.6	441
bta-miR-2370*	-22.7	605
bta-miR-2370*	-18.4	498
bta-miR-2370*	-18.1	231
bta-miR-2370*	-18.0	69
bta-miR-2370*	-17.5	202
bta-miR-2370*	-16.7	267
bta-miR-2370*	-16.0	735
bta-miR-2370*	-15.8	171
bta-miR-2370*	-15.2	348
bta-miR-2370*	-15.0	478
bta-miR-2370*	-14.5	413
bta-miR-2370*	-14.0	366
bta-miR-2370*	-13.7	11
bta-miR-2370*	-13.7	570
bta-miR-2370*	-13.2	629
bta-miR-2370*	-13.0	25
bta-miR-2370*	-12.0	548

bta-miR-2370*	-10.2	696
bta-miR-2370*	-10.1	101
bta-miR-2371	-20.1	147
bta-miR-2371	-19.6	47
bta-miR-2371	-16.8	366
bta-miR-2371	-16.4	405
bta-miR-2371	-16.3	470
bta-miR-2371	-16.1	119
bta-miR-2371	-14.4	338
bta-miR-2371	-13.7	725
bta-miR-2371	-13.5	506
bta-miR-2371	-13.2	188
bta-miR-2371	-13.0	557
bta-miR-2371	-12.8	598
bta-miR-2371	-12.8	381
bta-miR-2371	-12.5	570
bta-miR-2371	-12.4	66
bta-miR-2371	-12.3	94
bta-miR-2371	-12.2	166
bta-miR-2371	-12.0	453
bta-miR-2371	-11.5	28
bta-miR-2371	-11.4	484
bta-miR-2371	-11.3	648
bta-miR-2371	-11.2	325
bta-miR-2371	-11.2	620
bta-miR-2371	-10.9	273
bta-miR-2371	-10.9	232
bta-miR-2371	-10.8	313
bta-miR-2371	-10.6	108
bta-miR-2371	-10.4	16
bta-miR-2371	-10.1	429
bta-miR-2371	-10.0	215
bta-miR-2372	-18.8	130
bta-miR-2372	-18.8	514
bta-miR-2372	-18.1	365
bta-miR-2372	-17.0	275
bta-miR-2372	-16.9	27
bta-miR-2372	-16.8	85
bta-miR-2372	-16.7	623
bta-miR-2372	-16.4	670
bta-miR-2372	-16.0	424
bta-miR-2372	-16.0	162
bta-miR-2372	-16.0	569
bta-miR-2372	-14.8	242
bta-miR-2372	-14.5	185
bta-miR-2372	-14.4	465
bta-miR-2372	-14.0	711
bta-miR-2372	-13.9	229
bta-miR-2372	-13.5	307
bta-miR-2372	-13.5	12
bta-miR-2372	-13.3	392
bta-miR-2372	-12.9	109
bta-miR-2372	-12.7	583
bta-miR-2372	-12.2	324
bta-miR-2372	-11.5	404
bta-miR-2372	-11.4	455
bta-miR-2372	-11.4	738
bta-miR-2372	-10.2	413
bta-miR-2372	-10.2	650

bta-miR-2372	-10.1	66
bta-miR-2372	-10.1	1
bta-miR-2372	-10.0	493
bta-miR-2373	-33.4	425
bta-miR-2373	-29.4	359
bta-miR-2373	-27.0	596
bta-miR-2373	-26.9	403
bta-miR-2373	-25.9	163
bta-miR-2373	-24.9	462
bta-miR-2373	-23.3	242
bta-miR-2373	-23.2	622
bta-miR-2373	-22.5	81
bta-miR-2373	-22.4	27
bta-miR-2373	-22.2	199
bta-miR-2373	-22.0	483
bta-miR-2373	-22.0	563
bta-miR-2373	-20.7	4
bta-miR-2373	-18.8	119
bta-miR-2373	-17.9	650
bta-miR-2373	-16.7	515
bta-miR-2373	-15.7	577
bta-miR-2373	-15.5	231
bta-miR-2373	-15.1	185
bta-miR-2373	-14.2	325
bta-miR-2373	-14.2	738
bta-miR-2373	-13.9	614
bta-miR-2373	-13.6	539
bta-miR-2373	-13.4	308
bta-miR-2373	-12.8	455
bta-miR-2373	-12.6	281
bta-miR-2373	-12.3	664
bta-miR-2373	-11.6	378
bta-miR-2373	-11.1	698
bta-miR-2373	-10.5	136
bta-miR-2373	-10.4	587
bta-miR-2373	-10.2	634
bta-miR-2373*	-33.1	395
bta-miR-2373*	-26.6	85
bta-miR-2373*	-23.7	457
bta-miR-2373*	-22.8	58
bta-miR-2373*	-22.8	507
bta-miR-2373*	-22.7	5
bta-miR-2373*	-22.7	576
bta-miR-2373*	-20.9	252
bta-miR-2373*	-20.8	155
bta-miR-2373*	-20.2	120
bta-miR-2373*	-20.2	616
bta-miR-2373*	-17.5	632
bta-miR-2373*	-16.7	344
bta-miR-2373*	-16.6	424
bta-miR-2373*	-16.3	211
bta-miR-2373*	-16.3	551
bta-miR-2373*	-16.2	310
bta-miR-2373*	-15.5	39
bta-miR-2373*	-14.9	185
bta-miR-2373*	-14.1	374
bta-miR-2373*	-14.0	489
bta-miR-2373*	-13.7	691
bta-miR-2373*	-13.5	283

bta-miR-2373*	-13.4	23
bta-miR-2373*	-13.2	717
bta-miR-2373*	-12.4	136
bta-miR-2373*	-11.5	537
bta-miR-2373*	-11.5	677
bta-miR-2373*	-11.5	668
bta-miR-2374	-34.1	307
bta-miR-2374	-34.0	120
bta-miR-2374	-28.4	395
bta-miR-2374	-28.0	561
bta-miR-2374	-27.8	156
bta-miR-2374	-27.6	91
bta-miR-2374	-26.0	245
bta-miR-2374	-25.7	461
bta-miR-2374	-23.8	2
bta-miR-2374	-23.5	413
bta-miR-2374	-21.0	491
bta-miR-2374	-20.4	185
bta-miR-2374	-20.4	342
bta-miR-2374	-19.2	51
bta-miR-2374	-19.1	283
bta-miR-2374	-16.9	211
bta-miR-2374	-16.4	684
bta-miR-2374	-15.1	633
bta-miR-2374	-14.8	707
bta-miR-2374	-14.7	73
bta-miR-2374	-13.4	654
bta-miR-2374	-13.1	380
bta-miR-2374	-12.6	623
bta-miR-2374	-12.4	530
bta-miR-2374	-12.2	477
bta-miR-2374	-12.1	34
bta-miR-2374	-10.9	740
bta-miR-2374	-10.6	170
bta-miR-2375	-25.2	238
bta-miR-2375	-25.1	566
bta-miR-2375	-24.3	38
bta-miR-2375	-24.3	393
bta-miR-2375	-22.3	1
bta-miR-2375	-22.1	128
bta-miR-2375	-21.9	454
bta-miR-2375	-21.8	81
bta-miR-2375	-19.9	277
bta-miR-2375	-19.9	617
bta-miR-2375	-19.5	500
bta-miR-2375	-18.8	200
bta-miR-2375	-17.8	154
bta-miR-2375	-17.7	423
bta-miR-2375	-17.6	635
bta-miR-2375	-17.4	711
bta-miR-2375	-16.8	589
bta-miR-2375	-16.7	543
bta-miR-2375	-16.5	355
bta-miR-2375	-16.3	305
bta-miR-2375	-15.2	376
bta-miR-2375	-13.9	182
bta-miR-2375	-13.3	659
bta-miR-2375	-12.5	24
bta-miR-2375	-12.5	71

bta-miR-2375	-12.2	481
bta-miR-2375	-11.3	691
bta-miR-2375	-10.8	116
bta-miR-2375	-10.7	676
bta-miR-2375	-10.0	530
bta-miR-2376	-28.7	480
bta-miR-2376	-24.2	559
bta-miR-2376	-23.7	423
bta-miR-2376	-23.6	167
bta-miR-2376	-23.3	51
bta-miR-2376	-22.7	608
bta-miR-2376	-21.9	258
bta-miR-2376	-20.8	115
bta-miR-2376	-18.7	358
bta-miR-2376	-18.7	205
bta-miR-2376	-18.2	7
bta-miR-2376	-17.3	517
bta-miR-2376	-16.3	30
bta-miR-2376	-16.1	584
bta-miR-2376	-15.6	408
bta-miR-2376	-15.5	86
bta-miR-2376	-14.3	698
bta-miR-2376	-13.8	633
bta-miR-2376	-13.5	307
bta-miR-2376	-12.2	388
bta-miR-2376	-12.1	344
bta-miR-2376	-11.7	451
bta-miR-2376	-11.7	456
bta-miR-2376	-11.3	668
bta-miR-2376	-11.2	737
bta-miR-2376	-11.0	282
bta-miR-2376	-10.9	108
bta-miR-2376	-10.5	243
bta-miR-2376	-10.3	546
bta-miR-2377	-23.1	11
bta-miR-2377	-23.0	480
bta-miR-2377	-22.7	570
bta-miR-2377	-21.9	50
bta-miR-2377	-21.1	615
bta-miR-2377	-20.9	411
bta-miR-2377	-20.4	70
bta-miR-2377	-20.0	499
bta-miR-2377	-19.5	241
bta-miR-2377	-19.4	108
bta-miR-2377	-19.4	366
bta-miR-2377	-19.0	28
bta-miR-2377	-18.0	203
bta-miR-2377	-16.3	454
bta-miR-2377	-16.2	653
bta-miR-2377	-15.7	280
bta-miR-2377	-15.3	166
bta-miR-2377	-14.4	592
bta-miR-2377	-13.9	532
bta-miR-2377	-13.2	349
bta-miR-2377	-12.5	442
bta-miR-2377	-12.3	223
bta-miR-2377	-12.1	4
bta-miR-2377	-12.0	737
bta-miR-2377	-11.6	472

bta-miR-2377	-11.3	386
bta-miR-2377	-11.0	268
bta-miR-2377	-10.9	548
bta-miR-2377	-10.6	429
bta-miR-2377	-10.3	308
bta-miR-2377	-10.2	669
bta-miR-2377	-10.1	96
bta-miR-2378	-28.8	110
bta-miR-2378	-24.9	247
bta-miR-2378	-23.8	416
bta-miR-2378	-22.9	484
bta-miR-2378	-21.7	14
bta-miR-2378	-21.5	342
bta-miR-2378	-20.3	142
bta-miR-2378	-19.7	580
bta-miR-2378	-18.3	73
bta-miR-2378	-18.2	191
bta-miR-2378	-18.1	693
bta-miR-2378	-17.7	368
bta-miR-2378	-16.5	290
bta-miR-2378	-16.5	386
bta-miR-2378	-15.2	625
bta-miR-2378	-15.1	457
bta-miR-2378	-14.4	211
bta-miR-2378	-14.3	96
bta-miR-2378	-14.2	511
bta-miR-2378	-14.1	552
bta-miR-2378	-13.7	321
bta-miR-2378	-13.0	172
bta-miR-2378	-12.7	43
bta-miR-2378	-12.4	657
bta-miR-2378	-11.9	436
bta-miR-2378	-11.7	730
bta-miR-2378	-10.8	606
bta-miR-2378	-10.6	58
bta-miR-2378	-10.6	330
bta-miR-2378	-10.2	530
bta-miR-2378	-10.2	642
bta-miR-2378	-10.1	2
bta-miR-2379	-24.2	398
bta-miR-2379	-21.7	58
bta-miR-2379	-21.5	475
bta-miR-2379	-20.7	88
bta-miR-2379	-20.1	227
bta-miR-2379	-19.8	501
bta-miR-2379	-19.3	566
bta-miR-2379	-18.6	630
bta-miR-2379	-17.6	255
bta-miR-2379	-17.6	3
bta-miR-2379	-17.6	177
bta-miR-2379	-17.5	112
bta-miR-2379	-16.7	164
bta-miR-2379	-15.8	385
bta-miR-2379	-15.7	451
bta-miR-2379	-15.5	417
bta-miR-2379	-15.2	38
bta-miR-2379	-14.6	203
bta-miR-2379	-14.4	678
bta-miR-2379	-14.3	535

bta-miR-2379	-13.6	286
bta-miR-2379	-13.4	740
bta-miR-2379	-13.0	596
bta-miR-2379	-12.8	139
bta-miR-2379	-12.4	494
bta-miR-2379	-12.3	311
bta-miR-2379	-12.2	74
bta-miR-2379	-12.0	620
bta-miR-2379	-11.7	21
bta-miR-2379	-11.4	356
bta-miR-2379	-10.7	364
bta-miR-2379	-10.7	705
bta-miR-2379	-10.2	132
bta-miR-2284c	-17.4	405
bta-miR-2284c	-16.6	48
bta-miR-2284c	-16.1	331
bta-miR-2284c	-14.7	480
bta-miR-2284c	-14.4	78
bta-miR-2284c	-14.2	507
bta-miR-2284c	-13.7	444
bta-miR-2284c	-13.6	169
bta-miR-2284c	-13.2	99
bta-miR-2284c	-13.1	570
bta-miR-2284c	-13.0	273
bta-miR-2284c	-12.9	308
bta-miR-2284c	-12.7	361
bta-miR-2284c	-12.6	621
bta-miR-2284c	-12.4	124
bta-miR-2284c	-12.3	232
bta-miR-2284c	-12.2	726
bta-miR-2284c	-12.1	669
bta-miR-2284c	-12.1	464
bta-miR-2284c	-12.1	592
bta-miR-2284c	-11.9	34
bta-miR-2284c	-11.7	196
bta-miR-2284c	-11.2	340
bta-miR-2284c	-10.7	701
bta-miR-2284c	-10.6	20
bta-miR-2284c	-10.5	152
bta-miR-2284c	-10.1	736
bta-miR-2325b	-21.7	149
bta-miR-2325b	-20.8	121
bta-miR-2325b	-20.3	477
bta-miR-2325b	-18.9	227
bta-miR-2325b	-18.5	352
bta-miR-2325b	-18.1	405
bta-miR-2325b	-17.7	188
bta-miR-2325b	-17.5	92
bta-miR-2325b	-16.2	326
bta-miR-2325b	-16.1	586
bta-miR-2325b	-15.8	560
bta-miR-2325b	-15.3	65
bta-miR-2325b	-15.2	633
bta-miR-2325b	-15.1	504
bta-miR-2325b	-15.1	12
bta-miR-2325b	-14.2	48
bta-miR-2325b	-14.0	373
bta-miR-2325b	-13.9	300
bta-miR-2325b	-13.9	268

bta-miR-2325b	-13.7	456
bta-miR-2325b	-12.7	738
bta-miR-2325b	-11.0	698
bta-miR-2325b	-10.4	573
bta-miR-2325b	-10.1	171
bta-miR-2380	-19.0	424
bta-miR-2380	-17.6	29
bta-miR-2380	-17.4	365
bta-miR-2380	-16.8	472
bta-miR-2380	-16.7	509
bta-miR-2380	-16.0	71
bta-miR-2380	-15.6	398
bta-miR-2380	-15.3	716
bta-miR-2380	-14.8	187
bta-miR-2380	-14.4	545
bta-miR-2380	-14.3	340
bta-miR-2380	-13.8	243
bta-miR-2380	-13.1	622
bta-miR-2380	-13.0	583
bta-miR-2380	-13.0	212
bta-miR-2380	-12.9	108
bta-miR-2380	-12.3	12
bta-miR-2380	-11.5	130
bta-miR-2380	-11.3	286
bta-miR-2380	-11.1	50
bta-miR-2380	-10.7	648
bta-miR-2380	-10.7	569
bta-miR-2380	-10.2	166
bta-miR-2380	-10.0	91
bta-miR-2381	-31.0	482
bta-miR-2381	-30.6	407
bta-miR-2381	-28.7	184
bta-miR-2381	-27.2	50
bta-miR-2381	-27.0	8
bta-miR-2381	-23.4	228
bta-miR-2381	-23.3	429
bta-miR-2381	-23.1	265
bta-miR-2381	-23.1	97
bta-miR-2381	-22.1	596
bta-miR-2381	-21.8	155
bta-miR-2381	-21.8	650
bta-miR-2381	-20.1	344
bta-miR-2381	-19.1	34
bta-miR-2381	-18.8	555
bta-miR-2381	-18.7	616
bta-miR-2381	-18.6	123
bta-miR-2381	-18.4	376
bta-miR-2381	-16.8	572
bta-miR-2381	-14.3	462
bta-miR-2381	-14.0	515
bta-miR-2381	-13.6	669
bta-miR-2381	-13.2	294
bta-miR-2381	-12.5	736
bta-miR-2381	-12.3	143
bta-miR-2381	-11.7	216
bta-miR-2382	-34.5	106
bta-miR-2382	-29.4	414
bta-miR-2382	-29.1	72
bta-miR-2382	-25.8	494

bta-miR-2382	-25.2	183
bta-miR-2382	-25.2	270
bta-miR-2382	-24.5	230
bta-miR-2382	-24.2	456
bta-miR-2382	-23.6	559
bta-miR-2382	-23.4	623
bta-miR-2382	-23.3	4
bta-miR-2382	-21.7	321
bta-miR-2382	-21.4	140
bta-miR-2382	-21.1	383
bta-miR-2382	-19.2	653
bta-miR-2382	-18.1	698
bta-miR-2382	-17.4	41
bta-miR-2382	-17.4	586
bta-miR-2382	-15.9	341
bta-miR-2382	-15.6	517
bta-miR-2382	-15.3	603
bta-miR-2382	-14.6	678
bta-miR-2382	-14.5	165
bta-miR-2382	-14.1	539
bta-miR-2382	-13.7	294
bta-miR-2382	-13.7	484
bta-miR-2382	-13.3	214
bta-miR-2382	-12.5	259
bta-miR-2382	-12.5	737
bta-miR-2382	-11.6	368
bta-miR-2382	-11.4	98
bta-miR-2382	-10.6	435
bta-miR-2382*	-30.5	598
bta-miR-2382*	-28.7	412
bta-miR-2382*	-27.9	266
bta-miR-2382*	-25.7	480
bta-miR-2382*	-25.3	70
bta-miR-2382*	-22.8	621
bta-miR-2382*	-21.1	15
bta-miR-2382*	-20.7	356
bta-miR-2382*	-20.0	155
bta-miR-2382*	-20.0	199
bta-miR-2382*	-19.3	114
bta-miR-2382*	-16.3	229
bta-miR-2382*	-15.6	505
bta-miR-2382*	-13.2	560
bta-miR-2382*	-12.7	464
bta-miR-2382*	-12.0	738
bta-miR-2382*	-10.8	307
bta-miR-2382*	-10.6	337
bta-miR-2382*	-10.4	544
bta-miR-2382*	-10.2	381
bta-miR-2383	-22.7	263
bta-miR-2383	-22.3	44
bta-miR-2383	-19.9	198
bta-miR-2383	-18.8	377
bta-miR-2383	-18.1	609
bta-miR-2383	-17.0	2
bta-miR-2383	-15.9	566
bta-miR-2383	-15.8	482
bta-miR-2383	-15.6	85
bta-miR-2383	-15.2	17
bta-miR-2383	-15.0	408

bta-miR-2383	-14.9	125
bta-miR-2383	-14.8	170
bta-miR-2383	-14.6	64
bta-miR-2383	-13.5	658
bta-miR-2383	-12.9	543
bta-miR-2383	-12.8	449
bta-miR-2383	-12.8	629
bta-miR-2383	-12.4	150
bta-miR-2383	-11.8	282
bta-miR-2383	-11.8	393
bta-miR-2383	-11.0	692
bta-miR-2383	-10.8	107
bta-miR-2383	-10.8	463
bta-miR-2383	-10.4	516
bta-miR-2383	-10.2	220
bta-miR-2384	-27.4	501
bta-miR-2384	-23.6	45
bta-miR-2384	-22.9	87
bta-miR-2384	-22.5	384
bta-miR-2384	-22.1	458
bta-miR-2384	-21.9	564
bta-miR-2384	-19.7	235
bta-miR-2384	-19.2	4
bta-miR-2384	-18.9	207
bta-miR-2384	-17.4	143
bta-miR-2384	-16.8	421
bta-miR-2384	-15.5	532
bta-miR-2384	-15.1	596
bta-miR-2384	-15.0	111
bta-miR-2384	-14.8	317
bta-miR-2384	-14.5	354
bta-miR-2384	-14.4	191
bta-miR-2384	-13.8	670
bta-miR-2384	-12.6	705
bta-miR-2384	-12.0	650
bta-miR-2384	-11.6	331
bta-miR-2384	-11.4	31
bta-miR-2384	-11.4	277
bta-miR-2384	-10.8	74
bta-miR-2384	-10.7	617
bta-miR-2384	-10.7	690
bta-miR-2384	-10.6	626
bta-miR-2325c	-22.3	443
bta-miR-2325c	-19.7	218
bta-miR-2325c	-16.5	101
bta-miR-2325c	-14.2	338
bta-miR-2325c	-14.0	609
bta-miR-2325c	-13.8	174
bta-miR-2325c	-13.6	196
bta-miR-2325c	-13.1	725
bta-miR-2325c	-11.6	557
bta-miR-2325c	-11.2	499
bta-miR-2325c	-10.4	239
bta-miR-2325c	-10.4	479
bta-miR-2325c	-10.2	114
bta-miR-2325c	-10.2	165
bta-miR-2385-5p	-24.3	312
bta-miR-2385-5p	-23.1	125
bta-miR-2385-5p	-22.4	229

bta-miR-2385-5p	-21.6	574
bta-miR-2385-5p	-21.2	412
bta-miR-2385-5p	-20.5	378
bta-miR-2385-5p	-19.8	343
bta-miR-2385-5p	-19.7	94
bta-miR-2385-5p	-19.6	463
bta-miR-2385-5p	-18.9	19
bta-miR-2385-5p	-17.1	249
bta-miR-2385-5p	-17.1	156
bta-miR-2385-5p	-16.6	513
bta-miR-2385-5p	-16.5	57
bta-miR-2385-5p	-16.4	547
bta-miR-2385-5p	-16.3	618
bta-miR-2385-5p	-16.1	686
bta-miR-2385-5p	-15.4	270
bta-miR-2385-5p	-15.3	660
bta-miR-2385-5p	-14.7	184
bta-miR-2385-5p	-14.6	526
bta-miR-2385-5p	-14.5	489
bta-miR-2385-5p	-13.9	395
bta-miR-2385-5p	-13.6	329
bta-miR-2385-5p	-13.4	740
bta-miR-2385-5p	-13.0	73
bta-miR-2385-5p	-12.9	2
bta-miR-2385-5p	-12.8	293
bta-miR-2385-5p	-12.5	34
bta-miR-2385-5p	-12.4	702
bta-miR-2385-5p	-12.4	116
bta-miR-2385-5p	-11.3	365
bta-miR-2385-5p	-10.6	640
bta-miR-2385-5p	-10.3	51
bta-miR-2385-5p	-10.1	146
bta-miR-2385-5p	-10.1	483
bta-miR-2385-3p	-27.0	606
bta-miR-2385-3p	-25.9	437
bta-miR-2385-3p	-23.1	415
bta-miR-2385-3p	-22.5	350
bta-miR-2385-3p	-22.4	6
bta-miR-2385-3p	-20.6	61
bta-miR-2385-3p	-19.4	585
bta-miR-2385-3p	-18.2	493
bta-miR-2385-3p	-17.3	369
bta-miR-2385-3p	-17.0	630
bta-miR-2385-3p	-16.5	265
bta-miR-2385-3p	-16.0	216
bta-miR-2385-3p	-15.7	116
bta-miR-2385-3p	-15.5	156
bta-miR-2385-3p	-15.4	185
bta-miR-2385-3p	-14.6	737
bta-miR-2385-3p	-14.5	477
bta-miR-2385-3p	-13.5	535
bta-miR-2385-3p	-13.4	560
bta-miR-2385-3p	-12.2	649
bta-miR-2385-3p	-11.7	27
bta-miR-2385-3p	-10.9	403
bta-miR-2385-3p	-10.2	92
bta-miR-2284v	-20.4	261
bta-miR-2284v	-18.8	166
bta-miR-2284v	-18.6	327

bta-miR-2284v	-17.4	108
bta-miR-2284v	-17.4	28
bta-miR-2284v	-17.2	358
bta-miR-2284v	-16.5	49
bta-miR-2284v	-16.0	508
bta-miR-2284v	-16.0	232
bta-miR-2284v	-16.0	406
bta-miR-2284v	-15.7	471
bta-miR-2284v	-15.7	188
bta-miR-2284v	-15.6	622
bta-miR-2284v	-15.1	273
bta-miR-2284v	-15.1	308
bta-miR-2284v	-14.8	141
bta-miR-2284v	-14.8	119
bta-miR-2284v	-14.8	429
bta-miR-2284v	-13.8	94
bta-miR-2284v	-13.8	486
bta-miR-2284v	-13.4	134
bta-miR-2284v	-13.3	320
bta-miR-2284v	-13.3	715
bta-miR-2284v	-13.1	689
bta-miR-2284v	-12.9	595
bta-miR-2284v	-12.7	246
bta-miR-2284v	-12.7	80
bta-miR-2284v	-12.5	451
bta-miR-2284v	-11.9	210
bta-miR-2284v	-11.7	558
bta-miR-2284v	-11.4	7
bta-miR-2284v	-11.3	579
bta-miR-2284v	-11.1	342
bta-miR-2284v	-10.9	650
bta-miR-2284v	-10.2	669
bta-miR-2386	-18.5	470
bta-miR-2386	-17.9	114
bta-miR-2386	-16.5	220
bta-miR-2386	-15.8	350
bta-miR-2386	-15.7	614
bta-miR-2386	-15.3	443
bta-miR-2386	-14.6	589
bta-miR-2386	-14.3	177
bta-miR-2386	-14.2	405
bta-miR-2386	-14.1	333
bta-miR-2386	-13.4	499
bta-miR-2386	-13.4	565
bta-miR-2386	-13.3	726
bta-miR-2386	-12.8	41
bta-miR-2386	-12.5	265
bta-miR-2386	-11.9	102
bta-miR-2386	-11.8	661
bta-miR-2386	-11.2	16
bta-miR-2386	-11.2	155
bta-miR-2386	-11.1	312
bta-miR-2386	-11.0	415
bta-miR-2386	-10.4	79
bta-miR-2387	-30.6	415
bta-miR-2387	-29.4	116
bta-miR-2387	-26.6	66
bta-miR-2387	-26.5	560
bta-miR-2387	-26.4	465

bta-miR-2387	-25.2	266
bta-miR-2387	-24.7	654
bta-miR-2387	-24.0	488
bta-miR-2387	-23.6	4
bta-miR-2387	-22.1	41
bta-miR-2387	-21.9	185
bta-miR-2387	-20.4	617
bta-miR-2387	-19.8	387
bta-miR-2387	-19.6	586
bta-miR-2387	-19.0	231
bta-miR-2387	-18.1	436
bta-miR-2387	-17.5	156
bta-miR-2387	-17.2	308
bta-miR-2387	-16.8	685
bta-miR-2387	-16.6	352
bta-miR-2387	-15.2	96
bta-miR-2387	-14.8	606
bta-miR-2387	-14.6	25
bta-miR-2387	-13.9	734
bta-miR-2387	-13.3	520
bta-miR-2387	-11.6	548
bta-miR-2387	-10.5	325
bta-miR-2387	-10.5	212
bta-miR-2388*	-36.1	605
bta-miR-2388*	-29.9	435
bta-miR-2388*	-26.7	60
bta-miR-2388*	-26.4	205
bta-miR-2388*	-25.5	407
bta-miR-2388*	-24.5	487
bta-miR-2388*	-24.1	263
bta-miR-2388*	-23.6	165
bta-miR-2388*	-21.0	9
bta-miR-2388*	-19.9	582
bta-miR-2388*	-19.1	463
bta-miR-2388*	-18.9	344
bta-miR-2388*	-18.0	100
bta-miR-2388*	-15.5	653
bta-miR-2388*	-15.4	737
bta-miR-2388*	-14.9	630
bta-miR-2388*	-13.5	361
bta-miR-2388*	-11.7	238
bta-miR-2388*	-11.6	147
bta-miR-2388*	-11.5	516
bta-miR-2388*	-11.2	559
bta-miR-2388*	-10.3	194
bta-miR-2388*	-10.0	37
bta-miR-2388	-24.1	384
bta-miR-2388	-22.9	489
bta-miR-2388	-22.8	95
bta-miR-2388	-22.5	461
bta-miR-2388	-22.3	181
bta-miR-2388	-21.7	577
bta-miR-2388	-20.7	513
bta-miR-2388	-20.4	161
bta-miR-2388	-19.8	53
bta-miR-2388	-19.6	276
bta-miR-2388	-19.3	2
bta-miR-2388	-17.8	207
bta-miR-2388	-17.8	623

bta-miR-2388	-17.6	296
bta-miR-2388	-16.5	422
bta-miR-2388	-16.0	692
bta-miR-2388	-15.9	258
bta-miR-2388	-15.8	137
bta-miR-2388	-15.6	639
bta-miR-2388	-15.5	537
bta-miR-2388	-14.6	342
bta-miR-2388	-14.6	123
bta-miR-2388	-13.2	363
bta-miR-2388	-12.5	664
bta-miR-2388	-12.2	565
bta-miR-2388	-12.1	316
bta-miR-2388	-11.1	34
bta-miR-2388	-10.8	455
bta-miR-2388	-10.6	740
bta-miR-2388	-10.2	248
bta-miR-2389	-30.4	127
bta-miR-2389	-29.4	231
bta-miR-2389	-29.0	186
bta-miR-2389	-26.8	567
bta-miR-2389	-26.4	461
bta-miR-2389	-24.2	386
bta-miR-2389	-23.9	623
bta-miR-2389	-23.5	352
bta-miR-2389	-22.8	317
bta-miR-2389	-22.8	9
bta-miR-2389	-22.4	85
bta-miR-2389	-21.4	426
bta-miR-2389	-21.1	498
bta-miR-2389	-19.4	152
bta-miR-2389	-18.8	275
bta-miR-2389	-18.4	587
bta-miR-2389	-18.2	107
bta-miR-2389	-16.6	699
bta-miR-2389	-16.3	39
bta-miR-2389	-15.3	259
bta-miR-2389	-14.7	738
bta-miR-2389	-14.1	365
bta-miR-2389	-13.8	72
bta-miR-2389	-13.7	521
bta-miR-2389	-13.7	677
bta-miR-2389	-13.5	403
bta-miR-2389	-12.6	342
bta-miR-2389	-12.1	215
bta-miR-2389	-11.9	289
bta-miR-2389	-11.8	657
bta-miR-2389	-10.9	478
bta-miR-2389	-10.2	173
bta-miR-2390	-17.6	102
bta-miR-2390	-16.7	592
bta-miR-2390	-16.6	371
bta-miR-2390	-16.2	153
bta-miR-2390	-15.6	177
bta-miR-2390	-15.6	731
bta-miR-2390	-15.1	405
bta-miR-2390	-14.8	41
bta-miR-2390	-14.7	479
bta-miR-2390	-13.7	338

bta-miR-2390	-13.1	220
bta-miR-2390	-12.8	69
bta-miR-2390	-12.6	16
bta-miR-2390	-12.5	499
bta-miR-2390	-12.3	268
bta-miR-2390	-11.8	443
bta-miR-2390	-11.8	609
bta-miR-2390	-10.0	631
bta-miR-2391	-21.6	448
bta-miR-2391	-21.6	614
bta-miR-2391	-17.4	268
bta-miR-2391	-16.4	69
bta-miR-2391	-14.7	479
bta-miR-2391	-14.3	418
bta-miR-2391	-13.9	630
bta-miR-2391	-13.0	12
bta-miR-2391	-12.1	598
bta-miR-2391	-11.2	232
bta-miR-2391	-11.1	176
bta-miR-2391	-10.8	499
bta-miR-2391	-10.4	220
bta-miR-2392	-35.2	319
bta-miR-2392	-33.4	126
bta-miR-2392	-27.0	244
bta-miR-2392	-26.3	86
bta-miR-2392	-26.1	21
bta-miR-2392	-25.0	464
bta-miR-2392	-24.8	185
bta-miR-2392	-24.6	560
bta-miR-2392	-24.0	359
bta-miR-2392	-21.8	161
bta-miR-2392	-21.2	51
bta-miR-2392	-21.0	422
bta-miR-2392	-20.3	498
bta-miR-2392	-18.0	396
bta-miR-2392	-17.9	686
bta-miR-2392	-17.6	271
bta-miR-2392	-16.4	622
bta-miR-2392	-15.8	654
bta-miR-2392	-14.6	526
bta-miR-2392	-14.4	305
bta-miR-2392	-11.8	5
bta-miR-2392	-11.3	235
bta-miR-2392	-11.1	740
bta-miR-2392	-10.9	488
bta-miR-2392	-10.6	342
bta-miR-2392	-10.6	594
bta-miR-2392	-10.6	709
bta-miR-2392	-10.2	70
bta-miR-2392	-10.0	518
bta-miR-2393	-17.7	439
bta-miR-2393	-16.0	221
bta-miR-2393	-14.0	610
bta-miR-2393	-13.3	196
bta-miR-2393	-13.2	101
bta-miR-2393	-11.9	479
bta-miR-2393	-11.8	598
bta-miR-2393	-11.4	153
bta-miR-2393	-10.8	68

bta-miR-2393	-10.7	239
bta-miR-2393	-10.6	366
bta-miR-2393	-10.4	725
bta-miR-2393	-10.2	557
bta-miR-2393	-10.2	348
bta-miR-2394	-31.6	435
bta-miR-2394	-22.8	191
bta-miR-2394	-22.7	609
bta-miR-2394	-21.9	234
bta-miR-2394	-19.9	580
bta-miR-2394	-19.0	59
bta-miR-2394	-18.4	401
bta-miR-2394	-17.9	89
bta-miR-2394	-16.9	464
bta-miR-2394	-15.7	345
bta-miR-2394	-15.6	166
bta-miR-2394	-15.6	626
bta-miR-2394	-15.1	502
bta-miR-2394	-14.5	8
bta-miR-2394	-14.4	108
bta-miR-2394	-13.0	81
bta-miR-2394	-11.5	645
bta-miR-2394	-11.4	539
bta-miR-2394	-10.1	566
bta-miR-2395	-24.7	448
bta-miR-2395	-23.7	344
bta-miR-2395	-23.1	60
bta-miR-2395	-22.6	114
bta-miR-2395	-22.3	416
bta-miR-2395	-22.3	551
bta-miR-2395	-21.8	174
bta-miR-2395	-21.6	235
bta-miR-2395	-21.3	476
bta-miR-2395	-21.1	14
bta-miR-2395	-19.9	83
bta-miR-2395	-18.9	215
bta-miR-2395	-18.8	566
bta-miR-2395	-18.5	437
bta-miR-2395	-18.4	657
bta-miR-2395	-18.0	613
bta-miR-2395	-16.1	202
bta-miR-2395	-16.1	737
bta-miR-2395	-15.3	267
bta-miR-2395	-14.9	395
bta-miR-2395	-14.7	684
bta-miR-2395	-14.6	502
bta-miR-2395	-14.3	631
bta-miR-2395	-14.0	37
bta-miR-2395	-14.0	156
bta-miR-2395	-13.7	1
bta-miR-2395	-12.3	126
bta-miR-2395	-11.1	291
bta-miR-2395	-10.2	465
bta-miR-2395	-10.0	164
bta-miR-2396	-26.2	564
bta-miR-2396	-23.8	342
bta-miR-2396	-23.4	6
bta-miR-2396	-22.3	504
bta-miR-2396	-21.9	237

bta-miR-2396	-21.6	618
bta-miR-2396	-21.3	143
bta-miR-2396	-20.9	52
bta-miR-2396	-19.4	28
bta-miR-2396	-19.1	99
bta-miR-2396	-19.1	401
bta-miR-2396	-18.8	474
bta-miR-2396	-18.6	368
bta-miR-2396	-18.4	261
bta-miR-2396	-18.3	120
bta-miR-2396	-18.2	597
bta-miR-2396	-17.5	188
bta-miR-2396	-17.0	322
bta-miR-2396	-15.1	422
bta-miR-2396	-15.1	297
bta-miR-2396	-15.1	736
bta-miR-2396	-14.8	458
bta-miR-2396	-14.7	384
bta-miR-2396	-14.5	645
bta-miR-2396	-14.5	277
bta-miR-2396	-13.7	705
bta-miR-2396	-13.0	204
bta-miR-2396	-12.4	521
bta-miR-2396	-12.1	535
bta-miR-2396	-10.8	670
bta-miR-2396	-10.7	690
bta-miR-2396	-10.5	87
bta-miR-2285c	-17.2	51
bta-miR-2285c	-17.0	408
bta-miR-2285c	-16.2	237
bta-miR-2285c	-15.4	429
bta-miR-2285c	-15.4	606
bta-miR-2285c	-14.7	8
bta-miR-2285c	-14.1	483
bta-miR-2285c	-13.9	30
bta-miR-2285c	-13.3	645
bta-miR-2285c	-12.8	200
bta-miR-2285c	-12.5	169
bta-miR-2285c	-12.4	331
bta-miR-2285c	-12.4	277
bta-miR-2285c	-12.4	563
bta-miR-2285c	-12.4	515
bta-miR-2285c	-12.1	283
bta-miR-2285c	-12.0	452
bta-miR-2285c	-11.9	73
bta-miR-2285c	-11.6	126
bta-miR-2285c	-11.5	99
bta-miR-2285c	-11.4	669
bta-miR-2285c	-10.8	468
bta-miR-2285c	-10.7	544
bta-miR-2285c	-10.5	354
bta-miR-2285c	-10.5	628
bta-miR-2285c	-10.1	374
bta-miR-2397*	-29.2	602
bta-miR-2397*	-25.0	478
bta-miR-2397*	-24.2	408
bta-miR-2397*	-22.3	10
bta-miR-2397*	-21.4	69
bta-miR-2397*	-19.1	263

bta-miR-2397*	-18.3	165
bta-miR-2397*	-18.0	358
bta-miR-2397*	-17.4	437
bta-miR-2397*	-16.9	575
bta-miR-2397*	-16.7	122
bta-miR-2397*	-16.4	654
bta-miR-2397*	-16.0	219
bta-miR-2397*	-15.6	30
bta-miR-2397*	-14.8	106
bta-miR-2397*	-13.8	515
bta-miR-2397*	-13.8	154
bta-miR-2397*	-12.4	189
bta-miR-2397*	-12.4	544
bta-miR-2397*	-11.6	628
bta-miR-2397*	-10.9	115
bta-miR-2397*	-10.8	739
bta-miR-2397*	-10.5	304
bta-miR-2397*	-10.3	342
bta-miR-2397	-27.8	544
bta-miR-2397	-24.7	493
bta-miR-2397	-22.0	659
bta-miR-2397	-20.9	359
bta-miR-2397	-20.6	412
bta-miR-2397	-18.9	42
bta-miR-2397	-18.5	694
bta-miR-2397	-18.2	578
bta-miR-2397	-18.0	73
bta-miR-2397	-17.9	473
bta-miR-2397	-17.8	109
bta-miR-2397	-16.9	618
bta-miR-2397	-16.7	525
bta-miR-2397	-16.4	244
bta-miR-2397	-16.2	172
bta-miR-2397	-15.9	15
bta-miR-2397	-15.2	119
bta-miR-2397	-14.9	449
bta-miR-2397	-14.7	283
bta-miR-2397	-13.5	199
bta-miR-2397	-13.3	266
bta-miR-2397	-13.0	306
bta-miR-2397	-12.3	744
bta-miR-2397	-12.3	132
bta-miR-2397	-12.1	2
bta-miR-2397	-11.6	395
bta-miR-2397	-11.0	337
bta-miR-2397	-10.8	643
bta-miR-2398	-22.7	585
bta-miR-2398	-22.1	411
bta-miR-2398	-22.0	80
bta-miR-2398	-21.0	614
bta-miR-2398	-20.8	185
bta-miR-2398	-20.7	244
bta-miR-2398	-19.6	492
bta-miR-2398	-19.4	360
bta-miR-2398	-19.0	560
bta-miR-2398	-18.6	126
bta-miR-2398	-18.5	464
bta-miR-2398	-18.3	13
bta-miR-2398	-18.2	307

bta-miR-2398	-17.9	376
bta-miR-2398	-17.4	199
bta-miR-2398	-17.1	225
bta-miR-2398	-16.3	167
bta-miR-2398	-15.7	37
bta-miR-2398	-15.0	696
bta-miR-2398	-14.4	519
bta-miR-2398	-14.1	657
bta-miR-2398	-14.0	742
bta-miR-2398	-13.7	537
bta-miR-2398	-13.5	435
bta-miR-2398	-12.9	337
bta-miR-2398	-12.8	483
bta-miR-2398	-12.2	118
bta-miR-2398	-11.9	2
bta-miR-2398	-11.8	271
bta-miR-2398	-11.8	156
bta-miR-2398	-11.7	66
bta-miR-2398	-11.0	136
bta-miR-2398	-10.9	324
bta-miR-2398	-10.8	30
bta-miR-2399	-21.5	464
bta-miR-2399	-21.1	169
bta-miR-2399	-19.1	40
bta-miR-2399	-17.6	596
bta-miR-2399	-17.4	435
bta-miR-2399	-17.1	259
bta-miR-2399	-16.9	98
bta-miR-2399	-16.6	551
bta-miR-2399	-16.5	331
bta-miR-2399	-16.1	209
bta-miR-2399	-16.0	409
bta-miR-2399	-16.0	484
bta-miR-2399	-15.1	229
bta-miR-2399	-14.7	120
bta-miR-2399	-14.0	146
bta-miR-2399	-13.4	617
bta-miR-2399	-13.4	513
bta-miR-2399	-13.2	5
bta-miR-2399	-12.9	576
bta-miR-2399	-12.7	740
bta-miR-2399	-12.3	354
bta-miR-2399	-12.2	691
bta-miR-2399	-11.7	389
bta-miR-2399	-11.7	456
bta-miR-2399	-11.6	75
bta-miR-2399	-11.3	500
bta-miR-2399	-11.2	662
bta-miR-2399	-10.4	20
bta-miR-2399	-10.2	316
bta-miR-2399	-10.2	646
bta-miR-2399*	-18.9	56
bta-miR-2399*	-17.9	425
bta-miR-2399*	-17.0	520
bta-miR-2399*	-16.5	488
bta-miR-2399*	-16.0	540
bta-miR-2399*	-15.2	206
bta-miR-2399*	-15.2	654
bta-miR-2399*	-14.1	381

bta-miR-2399*	-14.0	73
bta-miR-2399*	-13.6	560
bta-miR-2399*	-13.0	1
bta-miR-2399*	-12.3	618
bta-miR-2399*	-11.2	636
bta-miR-2399*	-10.5	447
bta-miR-2399*	-10.3	476
bta-miR-2399*	-10.2	263
bta-miR-2399*	-10.2	683
bta-miR-2399*	-10.1	37
bta-miR-2399*	-10.0	412
bta-miR-2400	-29.8	410
bta-miR-2400	-29.3	566
bta-miR-2400	-27.9	107
bta-miR-2400	-26.4	4
bta-miR-2400	-24.5	80
bta-miR-2400	-23.9	344
bta-miR-2400	-22.8	234
bta-miR-2400	-21.9	461
bta-miR-2400	-21.8	539
bta-miR-2400	-20.9	617
bta-miR-2400	-20.2	495
bta-miR-2400	-19.8	165
bta-miR-2400	-19.1	37
bta-miR-2400	-18.5	200
bta-miR-2400	-17.6	393
bta-miR-2400	-17.4	737
bta-miR-2400	-17.2	596
bta-miR-2400	-17.0	277
bta-miR-2400	-17.0	650
bta-miR-2400	-15.8	434
bta-miR-2400	-15.3	263
bta-miR-2400	-13.5	692
bta-miR-2400	-13.1	299
bta-miR-2400	-13.0	664
bta-miR-2400	-12.8	128
bta-miR-2400	-12.7	378
bta-miR-2400	-12.6	25
bta-miR-2400	-12.4	482
bta-miR-2400	-12.0	455
bta-miR-2400	-11.5	522
bta-miR-2400	-11.2	71
bta-miR-2400	-10.7	635
bta-miR-2400	-10.1	218
bta-miR-2284q	-21.4	403
bta-miR-2284q	-20.1	60
bta-miR-2284q	-19.2	461
bta-miR-2284q	-18.1	14
bta-miR-2284q	-16.9	173
bta-miR-2284q	-16.7	596
bta-miR-2284q	-16.6	98
bta-miR-2284q	-16.3	611
bta-miR-2284q	-15.3	342
bta-miR-2284q	-15.0	692
bta-miR-2284q	-14.9	215
bta-miR-2284q	-14.6	364
bta-miR-2284q	-14.0	564
bta-miR-2284q	-13.8	730
bta-miR-2284q	-13.6	267

bta-miR-2284q	-13.4	451
bta-miR-2284q	-13.2	628
bta-miR-2284q	-13.0	491
bta-miR-2284q	-12.8	140
bta-miR-2284q	-12.0	657
bta-miR-2284q	-11.9	309
bta-miR-2284q	-11.6	514
bta-miR-2284q	-10.9	39
bta-miR-2284q	-10.7	242
bta-miR-2401	-32.4	107
bta-miR-2401	-25.5	472
bta-miR-2401	-23.5	361
bta-miR-2401	-22.7	165
bta-miR-2401	-22.4	429
bta-miR-2401	-21.9	407
bta-miR-2401	-21.2	563
bta-miR-2401	-19.9	623
bta-miR-2401	-19.9	258
bta-miR-2401	-19.3	29
bta-miR-2401	-18.8	64
bta-miR-2401	-17.9	596
bta-miR-2401	-17.9	234
bta-miR-2401	-17.4	141
bta-miR-2401	-17.0	344
bta-miR-2401	-16.7	198
bta-miR-2401	-16.5	4
bta-miR-2401	-16.2	495
bta-miR-2401	-15.4	737
bta-miR-2401	-14.5	389
bta-miR-2401	-13.6	89
bta-miR-2401	-13.1	580
bta-miR-2401	-12.3	97
bta-miR-2401	-10.9	308
bta-miR-2401	-10.8	698
bta-miR-2401	-10.4	610
bta-miR-2401	-10.3	663
bta-miR-2402	-27.3	605
bta-miR-2402	-25.9	13
bta-miR-2402	-23.2	420
bta-miR-2402	-21.8	61
bta-miR-2402	-20.7	185
bta-miR-2402	-19.9	477
bta-miR-2402	-19.0	369
bta-miR-2402	-17.3	266
bta-miR-2402	-17.1	403
bta-miR-2402	-16.5	623
bta-miR-2402	-15.6	582
bta-miR-2402	-15.5	560
bta-miR-2402	-15.2	216
bta-miR-2402	-14.8	644
bta-miR-2402	-13.9	86
bta-miR-2402	-13.8	156
bta-miR-2402	-12.9	116
bta-miR-2402	-12.4	730
bta-miR-2402	-12.0	352
bta-miR-2402	-11.2	519
bta-miR-2403	-27.9	70
bta-miR-2403	-27.4	408
bta-miR-2403	-25.6	480

bta-miR-2403	-24.1	574
bta-miR-2403	-23.7	615
bta-miR-2403	-22.8	16
bta-miR-2403	-22.6	166
bta-miR-2403	-22.5	114
bta-miR-2403	-22.0	268
bta-miR-2403	-21.0	447
bta-miR-2403	-20.7	233
bta-miR-2403	-20.1	392
bta-miR-2403	-17.9	515
bta-miR-2403	-17.6	544
bta-miR-2403	-16.0	348
bta-miR-2403	-15.9	205
bta-miR-2403	-15.0	51
bta-miR-2403	-14.8	698
bta-miR-2403	-14.8	736
bta-miR-2403	-14.8	654
bta-miR-2403	-14.2	37
bta-miR-2403	-13.9	153
bta-miR-2403	-13.6	308
bta-miR-2403	-12.9	249
bta-miR-2403	-12.8	97
bta-miR-2403	-12.8	371
bta-miR-2403	-12.0	632
bta-miR-2403	-11.7	604
bta-miR-2403	-11.5	468
bta-miR-2403	-11.0	140
bta-miR-2403	-10.5	8
bta-miR-2403	-10.0	324
bta-miR-2403	-10.0	330
bta-miR-2404	-22.8	479
bta-miR-2404	-22.2	586
bta-miR-2404	-20.1	506
bta-miR-2404	-19.5	349
bta-miR-2404	-19.2	10
bta-miR-2404	-19.0	66
bta-miR-2404	-17.0	618
bta-miR-2404	-16.9	405
bta-miR-2404	-16.8	185
bta-miR-2404	-15.6	649
bta-miR-2404	-15.5	238
bta-miR-2404	-15.1	100
bta-miR-2404	-15.1	451
bta-miR-2404	-14.5	367
bta-miR-2404	-14.4	28
bta-miR-2404	-13.4	542
bta-miR-2404	-13.2	559
bta-miR-2404	-12.8	423
bta-miR-2404	-12.1	165
bta-miR-2404	-11.9	663
bta-miR-2404	-11.4	51
bta-miR-2404	-11.2	523
bta-miR-2404	-10.9	278
bta-miR-2404	-10.7	224
bta-miR-2404	-10.6	338
bta-miR-2404	-10.2	128
bta-miR-449d	-25.0	111
bta-miR-449d	-24.4	60
bta-miR-449d	-24.1	401

bta-miR-449d	-23.3	190
bta-miR-449d	-21.0	484
bta-miR-449d	-20.8	146
bta-miR-449d	-19.0	14
bta-miR-449d	-18.9	236
bta-miR-449d	-18.7	431
bta-miR-449d	-18.5	368
bta-miR-449d	-18.4	34
bta-miR-449d	-18.2	461
bta-miR-449d	-18.1	259
bta-miR-449d	-17.2	579
bta-miR-449d	-17.1	317
bta-miR-449d	-16.0	625
bta-miR-449d	-15.6	170
bta-miR-449d	-15.3	346
bta-miR-449d	-14.9	299
bta-miR-449d	-14.8	650
bta-miR-449d	-14.5	515
bta-miR-449d	-13.9	559
bta-miR-449d	-13.4	90
bta-miR-449d	-12.3	132
bta-miR-449d	-12.2	737
bta-miR-449d	-11.9	681
bta-miR-449d	-11.3	607
bta-miR-449d	-11.1	447
bta-miR-449d	-11.1	80
bta-miR-449d	-10.2	703
bta-miR-2405	-25.2	114
bta-miR-2405	-24.9	480
bta-miR-2405	-23.1	418
bta-miR-2405	-20.7	357
bta-miR-2405	-19.6	154
bta-miR-2405	-19.4	41
bta-miR-2405	-19.3	70
bta-miR-2405	-17.6	586
bta-miR-2405	-17.5	11
bta-miR-2405	-16.9	621
bta-miR-2405	-16.7	268
bta-miR-2405	-16.6	233
bta-miR-2405	-16.4	449
bta-miR-2405	-16.3	563
bta-miR-2405	-16.1	174
bta-miR-2405	-16.1	308
bta-miR-2405	-14.9	331
bta-miR-2405	-14.3	499
bta-miR-2405	-13.5	405
bta-miR-2405	-12.9	663
bta-miR-2405	-12.7	464
bta-miR-2405	-12.5	136
bta-miR-2405	-12.3	97
bta-miR-2405	-11.9	698
bta-miR-2405	-11.7	218
bta-miR-2405	-11.6	575
bta-miR-2405	-11.6	604
bta-miR-2405	-10.4	727
bta-miR-2406	-23.9	234
bta-miR-2406	-22.0	409
bta-miR-2406	-21.0	277
bta-miR-2406	-21.0	127

bta-miR-2406	-20.8	191
bta-miR-2406	-20.5	391
bta-miR-2406	-19.9	460
bta-miR-2406	-19.5	89
bta-miR-2406	-19.1	706
bta-miR-2406	-18.3	555
bta-miR-2406	-18.2	626
bta-miR-2406	-18.1	493
bta-miR-2406	-16.8	663
bta-miR-2406	-16.2	44
bta-miR-2406	-15.2	151
bta-miR-2406	-15.2	309
bta-miR-2406	-15.0	356
bta-miR-2406	-14.7	8
bta-miR-2406	-13.8	179
bta-miR-2406	-12.9	113
bta-miR-2406	-12.4	72
bta-miR-2406	-11.6	583
bta-miR-2406	-11.5	516
bta-miR-2406	-10.9	691
bta-miR-2406	-10.8	170
bta-miR-2406	-10.6	740
bta-miR-2406	-10.3	484
bta-miR-2406	-10.0	263
bta-miR-2407	-29.0	107
bta-miR-2407	-27.2	318
bta-miR-2407	-26.6	155
bta-miR-2407	-26.2	243
bta-miR-2407	-26.1	392
bta-miR-2407	-25.8	127
bta-miR-2407	-25.8	85
bta-miR-2407	-23.6	559
bta-miR-2407	-22.9	456
bta-miR-2407	-21.9	576
bta-miR-2407	-21.8	50
bta-miR-2407	-21.5	515
bta-miR-2407	-21.2	207
bta-miR-2407	-20.9	361
bta-miR-2407	-20.2	9
bta-miR-2407	-19.9	482
bta-miR-2407	-19.3	282
bta-miR-2407	-17.0	623
bta-miR-2407	-16.6	183
bta-miR-2407	-15.8	653
bta-miR-2407	-15.7	687
bta-miR-2407	-13.3	342
bta-miR-2407	-13.0	33
bta-miR-2407	-12.2	703
bta-miR-2407	-11.9	72
bta-miR-2407	-11.7	740
bta-miR-2407	-11.2	539
bta-miR-2407	-11.0	495
bta-miR-2408	-28.8	12
bta-miR-2408	-25.0	617
bta-miR-2408	-23.9	413
bta-miR-2408	-22.2	462
bta-miR-2408	-20.6	72
bta-miR-2408	-20.4	342
bta-miR-2408	-19.5	110

bta-miR-2408	-19.4	369
bta-miR-2408	-19.2	582
bta-miR-2408	-17.4	169
bta-miR-2408	-16.9	28
bta-miR-2408	-16.8	270
bta-miR-2408	-15.4	598
bta-miR-2408	-15.1	560
bta-miR-2408	-15.1	450
bta-miR-2408	-14.8	155
bta-miR-2408	-14.2	740
bta-miR-2408	-14.1	199
bta-miR-2408	-13.7	662
bta-miR-2408	-13.6	216
bta-miR-2408	-12.9	237
bta-miR-2408	-12.9	515
bta-miR-2408	-12.8	146
bta-miR-2408	-12.5	50
bta-miR-2408	-12.5	126
bta-miR-2408	-11.5	698
bta-miR-2408	-10.8	403
bta-miR-2408	-10.6	499
bta-miR-2408	-10.3	313
bta-miR-2409	-28.7	12
bta-miR-2409	-23.8	418
bta-miR-2409	-23.5	70
bta-miR-2409	-23.0	615
bta-miR-2409	-21.1	480
bta-miR-2409	-19.7	219
bta-miR-2409	-19.0	114
bta-miR-2409	-18.8	175
bta-miR-2409	-18.4	268
bta-miR-2409	-17.9	449
bta-miR-2409	-15.9	516
bta-miR-2409	-15.8	560
bta-miR-2409	-15.8	342
bta-miR-2409	-15.7	371
bta-miR-2409	-15.6	605
bta-miR-2409	-13.8	653
bta-miR-2409	-13.6	38
bta-miR-2409	-12.8	358
bta-miR-2409	-12.5	154
bta-miR-2409	-12.4	527
bta-miR-2409	-12.0	205
bta-miR-2409	-11.9	464
bta-miR-2409	-11.8	63
bta-miR-2409	-11.7	243
bta-miR-2409	-11.7	393
bta-miR-2409	-11.6	408
bta-miR-2409	-11.4	308
bta-miR-2409	-10.9	257
bta-miR-2409	-10.4	742
bta-miR-2409	-10.3	3
bta-miR-2409	-10.1	472
bta-miR-2410	-25.9	575
bta-miR-2410	-24.0	420
bta-miR-2410	-22.0	147
bta-miR-2410	-21.8	276
bta-miR-2410	-21.8	199
bta-miR-2410	-21.4	391

bta-miR-2410	-21.0	502
bta-miR-2410	-20.9	66
bta-miR-2410	-20.9	461
bta-miR-2410	-20.4	628
bta-miR-2410	-20.0	238
bta-miR-2410	-18.8	114
bta-miR-2410	-17.1	172
bta-miR-2410	-16.6	4
bta-miR-2410	-15.3	354
bta-miR-2410	-15.1	606
bta-miR-2410	-15.0	132
bta-miR-2410	-14.9	190
bta-miR-2410	-14.8	704
bta-miR-2410	-14.8	41
bta-miR-2410	-14.8	299
bta-miR-2410	-14.0	655
bta-miR-2410	-13.6	691
bta-miR-2410	-13.4	548
bta-miR-2410	-12.4	739
bta-miR-2410	-12.4	22
bta-miR-2410	-11.9	678
bta-miR-2410	-11.2	319
bta-miR-2410	-11.1	488
bta-miR-2410	-10.2	107
bta-miR-2410	-10.2	451
bta-miR-1584	-33.7	156
bta-miR-1584	-32.1	109
bta-miR-1584	-31.1	244
bta-miR-1584	-30.6	30
bta-miR-1584	-30.5	307
bta-miR-1584	-28.9	415
bta-miR-1584	-27.3	457
bta-miR-1584	-24.1	360
bta-miR-1584	-23.1	88
bta-miR-1584	-22.8	562
bta-miR-1584	-22.2	395
bta-miR-1584	-20.7	187
bta-miR-1584	-20.6	271
bta-miR-1584	-20.5	483
bta-miR-1584	-20.1	3
bta-miR-1584	-19.2	581
bta-miR-1584	-17.9	60
bta-miR-1584	-17.2	519
bta-miR-1584	-16.4	140
bta-miR-1584	-16.3	654
bta-miR-1584	-16.2	624
bta-miR-1584	-16.0	234
bta-miR-1584	-15.0	538
bta-miR-1584	-14.5	377
bta-miR-1584	-13.9	697
bta-miR-1584	-13.5	730
bta-miR-1584	-12.7	262
bta-miR-1584	-10.9	599
bta-miR-1584	-10.6	211
bta-miR-1584	-10.6	337
bta-miR-1584*	-33.5	419
bta-miR-1584*	-27.9	12
bta-miR-1584*	-26.2	616
bta-miR-1584*	-25.1	199

bta-miR-1584*	-21.4	463
bta-miR-1584*	-20.4	560
bta-miR-1584*	-20.2	72
bta-miR-1584*	-19.0	155
bta-miR-1584*	-18.6	584
bta-miR-1584*	-16.6	337
bta-miR-1584*	-16.5	448
bta-miR-1584*	-16.3	231
bta-miR-1584*	-16.0	112
bta-miR-1584*	-14.0	660
bta-miR-1584*	-13.9	403
bta-miR-1584*	-13.5	371
bta-miR-1584*	-13.5	547
bta-miR-1584*	-13.1	38
bta-miR-1584*	-12.8	1
bta-miR-1584*	-11.7	738
bta-miR-1584*	-11.6	185
bta-miR-1584*	-11.4	60
bta-miR-1584*	-10.6	518
bta-miR-1584*	-10.3	386
bta-miR-1940	-25.6	481
bta-miR-1940	-24.9	418
bta-miR-1940	-23.9	232
bta-miR-1940	-23.4	71
bta-miR-1940	-23.0	371
bta-miR-1940	-22.8	11
bta-miR-1940	-22.1	275
bta-miR-1940	-22.1	622
bta-miR-1940	-22.1	188
bta-miR-1940	-21.8	102
bta-miR-1940	-21.0	41
bta-miR-1940	-20.3	560
bta-miR-1940	-19.1	154
bta-miR-1940	-18.4	130
bta-miR-1940	-17.9	450
bta-miR-1940	-17.7	653
bta-miR-1940	-15.8	698
bta-miR-1940	-15.5	582
bta-miR-1940	-13.2	737
bta-miR-1940	-12.7	351
bta-miR-1940	-11.7	315
bta-miR-1940	-11.7	407
bta-miR-1940	-11.6	520
bta-miR-1940	-11.6	678
bta-miR-1940	-10.9	542
bta-miR-2411	-30.2	560
bta-miR-2411	-27.4	115
bta-miR-2411	-27.2	482
bta-miR-2411	-25.0	12
bta-miR-2411	-24.4	619
bta-miR-2411	-24.1	85
bta-miR-2411	-24.0	370
bta-miR-2411	-21.0	155
bta-miR-2411	-21.0	413
bta-miR-2411	-19.2	40
bta-miR-2411	-18.6	267
bta-miR-2411	-18.1	654
bta-miR-2411	-17.9	343
bta-miR-2411	-17.9	450

bta-miR-2411	-16.1	583
bta-miR-2411	-16.1	741
bta-miR-2411	-15.6	206
bta-miR-2411	-15.4	307
bta-miR-2411	-14.9	1
bta-miR-2411	-14.2	698
bta-miR-2411	-13.9	539
bta-miR-2411	-13.9	519
bta-miR-2411	-13.6	599
bta-miR-2411	-13.2	432
bta-miR-2411	-12.6	244
bta-miR-2411	-12.5	473
bta-miR-2411	-10.3	403
bta-miR-2411	-10.2	326
bta-miR-2411	-10.2	364
bta-miR-2411	-10.1	144
bta-miR-2411*	-26.4	614
bta-miR-2411*	-23.5	448
bta-miR-2411*	-22.5	16
bta-miR-2411*	-20.9	217
bta-miR-2411*	-20.8	589
bta-miR-2411*	-20.6	266
bta-miR-2411*	-20.4	478
bta-miR-2411*	-20.4	69
bta-miR-2411*	-19.3	499
bta-miR-2411*	-18.7	412
bta-miR-2411*	-18.3	350
bta-miR-2411*	-16.6	176
bta-miR-2411*	-15.5	94
bta-miR-2411*	-14.7	152
bta-miR-2411*	-13.3	544
bta-miR-2411*	-13.2	736
bta-miR-2411*	-12.3	659
bta-miR-2411*	-11.0	1
bta-miR-2411*	-10.8	609
bta-miR-2411*	-10.7	170
bta-miR-2411*	-10.6	566
bta-miR-2411*	-10.0	383
bta-miR-2412	-35.1	309
bta-miR-2412	-34.6	135
bta-miR-2412	-29.0	396
bta-miR-2412	-26.7	90
bta-miR-2412	-26.4	209
bta-miR-2412	-25.9	458
bta-miR-2412	-23.0	564
bta-miR-2412	-22.9	248
bta-miR-2412	-21.8	3
bta-miR-2412	-21.8	502
bta-miR-2412	-21.6	362
bta-miR-2412	-19.6	474
bta-miR-2412	-19.4	186
bta-miR-2412	-19.4	581
bta-miR-2412	-18.8	117
bta-miR-2412	-18.5	637
bta-miR-2412	-16.7	58
bta-miR-2412	-16.5	692
bta-miR-2412	-15.1	429
bta-miR-2412	-14.2	43
bta-miR-2412	-13.5	677

bta-miR-2412	-12.6	623
bta-miR-2412	-12.3	537
bta-miR-2412	-11.7	287
bta-miR-2412	-11.1	740
bta-miR-2412	-10.9	550
bta-miR-2412	-10.6	342
bta-miR-2412	-10.6	170
bta-miR-2412	-10.3	489
bta-miR-2413	-29.8	86
bta-miR-2413	-27.8	460
bta-miR-2413	-27.4	560
bta-miR-2413	-26.8	387
bta-miR-2413	-26.7	492
bta-miR-2413	-26.0	21
bta-miR-2413	-25.6	250
bta-miR-2413	-24.6	162
bta-miR-2413	-24.1	341
bta-miR-2413	-22.9	199
bta-miR-2413	-22.7	618
bta-miR-2413	-19.5	126
bta-miR-2413	-19.3	6
bta-miR-2413	-18.7	425
bta-miR-2413	-18.4	320
bta-miR-2413	-17.4	61
bta-miR-2413	-15.5	580
bta-miR-2413	-15.4	685
bta-miR-2413	-14.9	275
bta-miR-2413	-14.6	654
bta-miR-2413	-14.5	595
bta-miR-2413	-14.0	241
bta-miR-2413	-13.3	516
bta-miR-2413	-12.2	190
bta-miR-2413	-12.1	536
bta-miR-2413	-11.7	739
bta-miR-2413	-11.1	637
bta-miR-2413	-10.7	449
bta-miR-2413	-10.6	307
bta-miR-2413	-10.1	142
bta-miR-2284m	-20.0	366
bta-miR-2284m	-16.8	592
bta-miR-2284m	-16.7	196
bta-miR-2284m	-16.4	166
bta-miR-2284m	-16.2	273
bta-miR-2284m	-15.7	470
bta-miR-2284m	-15.4	60
bta-miR-2284m	-15.1	108
bta-miR-2284m	-14.7	507
bta-miR-2284m	-14.3	405
bta-miR-2284m	-14.2	621
bta-miR-2284m	-14.2	28
bta-miR-2284m	-13.7	241
bta-miR-2284m	-13.6	706
bta-miR-2284m	-13.6	724
bta-miR-2284m	-13.5	338
bta-miR-2284m	-13.4	5
bta-miR-2284m	-13.1	308
bta-miR-2284m	-12.4	451
bta-miR-2284m	-12.2	91
bta-miR-2284m	-11.9	423

bta-miR-2284m	-11.8	327
bta-miR-2284m	-11.7	567
bta-miR-2284m	-11.4	142
bta-miR-2284m	-11.2	460
bta-miR-2284m	-11.1	216
bta-miR-2284m	-10.6	581
bta-miR-2284m	-10.5	48
bta-miR-2284m	-10.4	669
bta-miR-2284m	-10.4	552
bta-miR-2414	-22.1	51
bta-miR-2414	-21.3	105
bta-miR-2414	-20.9	559
bta-miR-2414	-20.8	130
bta-miR-2414	-20.4	158
bta-miR-2414	-19.4	480
bta-miR-2414	-19.3	408
bta-miR-2414	-19.0	341
bta-miR-2414	-19.0	372
bta-miR-2414	-18.4	457
bta-miR-2414	-17.8	1
bta-miR-2414	-17.3	243
bta-miR-2414	-17.1	633
bta-miR-2414	-16.9	591
bta-miR-2414	-16.8	202
bta-miR-2414	-16.8	510
bta-miR-2414	-15.2	669
bta-miR-2414	-15.0	82
bta-miR-2414	-14.1	319
bta-miR-2414	-13.6	425
bta-miR-2414	-12.9	282
bta-miR-2414	-12.8	360
bta-miR-2414	-12.4	736
bta-miR-2414	-11.7	229
bta-miR-2414	-11.0	29
bta-miR-2414	-10.6	548
bta-miR-2414	-10.1	704
bta-miR-2415*	-33.3	413
bta-miR-2415*	-30.9	115
bta-miR-2415*	-28.6	167
bta-miR-2415*	-28.2	12
bta-miR-2415*	-28.2	617
bta-miR-2415*	-27.4	72
bta-miR-2415*	-24.7	482
bta-miR-2415*	-24.4	448
bta-miR-2415*	-23.8	563
bta-miR-2415*	-23.1	599
bta-miR-2415*	-22.9	270
bta-miR-2415*	-18.9	38
bta-miR-2415*	-18.4	220
bta-miR-2415*	-18.4	342
bta-miR-2415*	-18.1	369
bta-miR-2415*	-17.5	199
bta-miR-2415*	-16.3	136
bta-miR-2415*	-16.1	308
bta-miR-2415*	-15.1	698
bta-miR-2415*	-14.6	662
bta-miR-2415*	-14.1	242
bta-miR-2415*	-14.1	154
bta-miR-2415*	-13.7	738

bta-miR-2415*	-12.9	63
bta-miR-2415*	-12.3	97
bta-miR-2415*	-12.3	325
bta-miR-2415*	-12.3	586
bta-miR-2415*	-11.2	393
bta-miR-2415*	-10.8	1
bta-miR-2415*	-10.3	516
bta-miR-2415	-27.7	88
bta-miR-2415	-26.0	408
bta-miR-2415	-24.4	3
bta-miR-2415	-24.0	463
bta-miR-2415	-22.1	60
bta-miR-2415	-21.9	597
bta-miR-2415	-21.8	565
bta-miR-2415	-21.3	231
bta-miR-2415	-20.9	257
bta-miR-2415	-20.0	616
bta-miR-2415	-19.9	164
bta-miR-2415	-19.0	38
bta-miR-2415	-18.3	504
bta-miR-2415	-17.2	115
bta-miR-2415	-17.1	659
bta-miR-2415	-16.5	147
bta-miR-2415	-15.8	356
bta-miR-2415	-15.5	436
bta-miR-2415	-14.3	177
bta-miR-2415	-14.0	546
bta-miR-2415	-13.9	377
bta-miR-2415	-13.9	308
bta-miR-2415	-13.9	487
bta-miR-2415	-12.7	580
bta-miR-2415	-12.6	198
bta-miR-2415	-12.4	277
bta-miR-2415	-12.2	686
bta-miR-2415	-12.0	740
bta-miR-2415	-10.7	23
bta-miR-2415	-10.6	525
bta-miR-2415	-10.4	644
bta-miR-2415	-10.3	209
bta-miR-2416	-28.3	398
bta-miR-2416	-26.7	58
bta-miR-2416	-24.1	4
bta-miR-2416	-23.6	259
bta-miR-2416	-23.2	512
bta-miR-2416	-22.9	459
bta-miR-2416	-21.5	98
bta-miR-2416	-20.8	637
bta-miR-2416	-20.7	565
bta-miR-2416	-20.5	615
bta-miR-2416	-18.6	491
bta-miR-2416	-18.4	168
bta-miR-2416	-18.1	368
bta-miR-2416	-15.9	193
bta-miR-2416	-15.8	212
bta-miR-2416	-15.8	431
bta-miR-2416	-15.5	591
bta-miR-2416	-14.2	316
bta-miR-2416	-13.9	146
bta-miR-2416	-13.8	34

bta-miR-2416	-13.3	626
bta-miR-2416	-13.2	535
bta-miR-2416	-12.6	343
bta-miR-2416	-12.3	692
bta-miR-2416	-12.3	120
bta-miR-2416	-12.2	713
bta-miR-2416	-11.8	284
bta-miR-2416	-11.5	384
bta-miR-2416	-10.3	90
bta-miR-2416	-10.2	136
bta-miR-2417	-21.1	39
bta-miR-2417	-20.8	539
bta-miR-2417	-18.5	516
bta-miR-2417	-17.2	379
bta-miR-2417	-17.1	277
bta-miR-2417	-17.1	356
bta-miR-2417	-16.9	463
bta-miR-2417	-16.4	575
bta-miR-2417	-16.2	307
bta-miR-2417	-16.0	181
bta-miR-2417	-15.4	67
bta-miR-2417	-15.3	25
bta-miR-2417	-15.0	113
bta-miR-2417	-14.9	632
bta-miR-2417	-14.8	490
bta-miR-2417	-14.8	200
bta-miR-2417	-14.8	410
bta-miR-2417	-14.3	390
bta-miR-2417	-14.1	238
bta-miR-2417	-14.0	423
bta-miR-2417	-13.7	657
bta-miR-2417	-13.6	1
bta-miR-2417	-13.3	263
bta-miR-2417	-13.1	80
bta-miR-2417	-12.0	678
bta-miR-2417	-11.7	137
bta-miR-2417	-11.7	617
bta-miR-2417	-11.5	478
bta-miR-2417	-11.1	316
bta-miR-2417	-11.1	17
bta-miR-2417	-10.7	93
bta-miR-2417	-10.7	154
bta-miR-2417	-10.7	337
bta-miR-2417	-10.4	743
bta-miR-2417	-10.4	603
bta-miR-2417	-10.3	707
bta-miR-2417	-10.3	58
bta-miR-2417	-10.3	328
bta-miR-2417	-10.2	504
bta-miR-2417	-10.1	167
bta-miR-2418	-29.9	119
bta-miR-2418	-24.1	366
bta-miR-2418	-23.0	414
bta-miR-2418	-22.9	312
bta-miR-2418	-22.2	143
bta-miR-2418	-21.7	235
bta-miR-2418	-21.4	560
bta-miR-2418	-21.0	72
bta-miR-2418	-20.4	20

bta-miR-2418	-20.4	482
bta-miR-2418	-20.0	45
bta-miR-2418	-19.1	188
bta-miR-2418	-18.0	451
bta-miR-2418	-16.9	1
bta-miR-2418	-16.5	697
bta-miR-2418	-16.4	95
bta-miR-2418	-15.6	659
bta-miR-2418	-15.5	267
bta-miR-2418	-15.2	738
bta-miR-2418	-14.7	341
bta-miR-2418	-14.0	595
bta-miR-2418	-13.8	635
bta-miR-2418	-13.4	394
bta-miR-2418	-12.7	405
bta-miR-2418	-12.7	518
bta-miR-2418	-11.6	305
bta-miR-2418	-11.1	611
bta-miR-2418	-10.9	473
bta-miR-2284b	-18.0	47
bta-miR-2284b	-18.0	405
bta-miR-2284b	-16.2	479
bta-miR-2284b	-15.4	124
bta-miR-2284b	-15.4	225
bta-miR-2284b	-15.2	166
bta-miR-2284b	-15.2	441
bta-miR-2284b	-14.9	338
bta-miR-2284b	-14.8	102
bta-miR-2284b	-14.8	263
bta-miR-2284b	-14.4	506
bta-miR-2284b	-13.3	308
bta-miR-2284b	-12.8	589
bta-miR-2284b	-12.8	196
bta-miR-2284b	-12.7	272
bta-miR-2284b	-12.7	20
bta-miR-2284b	-12.7	80
bta-miR-2284b	-12.5	648
bta-miR-2284b	-12.2	366
bta-miR-2284b	-11.8	620
bta-miR-2284b	-11.7	570
bta-miR-2284b	-11.6	669
bta-miR-2284b	-11.5	176
bta-miR-2284b	-11.5	735
bta-miR-2284b	-11.4	495
bta-miR-2284b	-10.5	701
bta-miR-1814c	-20.1	350
bta-miR-1814c	-19.0	268
bta-miR-1814c	-18.8	103
bta-miR-1814c	-18.2	159
bta-miR-1814c	-16.8	454
bta-miR-1814c	-16.7	480
bta-miR-1814c	-16.1	228
bta-miR-1814c	-15.4	184
bta-miR-1814c	-15.1	326
bta-miR-1814c	-14.6	599
bta-miR-1814c	-14.1	405
bta-miR-1814c	-14.1	423
bta-miR-1814c	-14.0	28
bta-miR-1814c	-13.5	508

bta-miR-1814c	-13.1	204
bta-miR-1814c	-12.9	138
bta-miR-1814c	-12.8	726
bta-miR-1814c	-12.5	303
bta-miR-1814c	-12.1	48
bta-miR-1814c	-11.8	650
bta-miR-1814c	-11.6	10
bta-miR-1814c	-11.2	557
bta-miR-1814c	-10.5	622
bta-miR-1814c	-10.5	79
bta-miR-1814c	-10.0	320
bta-miR-2419	-26.2	10
bta-miR-2419	-23.1	601
bta-miR-2419	-22.1	80
bta-miR-2419	-18.5	348
bta-miR-2419	-17.6	494
bta-miR-2419	-16.5	198
bta-miR-2419	-16.0	543
bta-miR-2419	-15.8	264
bta-miR-2419	-15.5	238
bta-miR-2419	-15.0	38
bta-miR-2419	-14.8	170
bta-miR-2419	-14.7	410
bta-miR-2419	-14.5	440
bta-miR-2419	-14.5	653
bta-miR-2419	-13.9	737
bta-miR-2419	-13.7	576
bta-miR-2419	-13.4	623
bta-miR-2419	-12.6	516
bta-miR-2419	-12.1	65
bta-miR-2419	-11.8	105
bta-miR-2419	-11.2	128
bta-miR-2419*	-29.0	550
bta-miR-2419*	-23.6	347
bta-miR-2419*	-23.4	1
bta-miR-2419*	-23.2	413
bta-miR-2419*	-23.1	229
bta-miR-2419*	-20.4	654
bta-miR-2419*	-20.2	452
bta-miR-2419*	-19.8	311
bta-miR-2419*	-19.3	478
bta-miR-2419*	-19.1	113
bta-miR-2419*	-18.7	205
bta-miR-2419*	-18.4	500
bta-miR-2419*	-18.1	372
bta-miR-2419*	-18.1	161
bta-miR-2419*	-17.6	81
bta-miR-2419*	-17.5	38
bta-miR-2419*	-16.5	614
bta-miR-2419*	-16.3	174
bta-miR-2419*	-16.1	570
bta-miR-2419*	-16.0	326
bta-miR-2419*	-14.7	266
bta-miR-2419*	-14.6	685
bta-miR-2419*	-14.5	94
bta-miR-2419*	-14.5	128
bta-miR-2419*	-14.1	516
bta-miR-2419*	-13.1	292
bta-miR-2419*	-13.1	137

bta-miR-2419*	-12.9	147
bta-miR-2419*	-12.8	63
bta-miR-2419*	-12.6	589
bta-miR-2419*	-12.1	742
bta-miR-2419*	-11.2	529
bta-miR-2419*	-10.9	251
bta-miR-2419*	-10.5	408
bta-miR-2419*	-10.4	342
bta-miR-2419*	-10.2	705
bta-miR-2420	-18.5	153
bta-miR-2420	-18.0	175
bta-miR-2420	-17.5	405
bta-miR-2420	-17.2	731
bta-miR-2420	-15.6	349
bta-miR-2420	-14.9	101
bta-miR-2420	-14.6	222
bta-miR-2420	-14.4	479
bta-miR-2420	-14.2	600
bta-miR-2420	-14.0	613
bta-miR-2420	-13.9	16
bta-miR-2420	-13.7	442
bta-miR-2420	-13.6	371
bta-miR-2420	-13.3	268
bta-miR-2420	-13.1	197
bta-miR-2420	-12.4	574
bta-miR-2420	-12.1	65
bta-miR-2420	-12.0	80
bta-miR-2420	-11.9	651
bta-miR-2420	-11.4	313
bta-miR-2420	-11.3	49
bta-miR-2420	-11.2	129
bta-miR-2420	-10.9	300
bta-miR-2420	-10.7	334
bta-miR-2420	-10.7	555
bta-miR-2420	-10.1	469
bta-miR-2421	-15.2	65
bta-miR-2421	-14.7	600
bta-miR-2421	-14.5	351
bta-miR-2421	-14.1	159
bta-miR-2421	-13.4	434
bta-miR-2421	-13.2	204
bta-miR-2421	-12.9	11
bta-miR-2421	-12.5	737
bta-miR-2421	-12.4	267
bta-miR-2421	-11.8	477
bta-miR-2421	-11.3	504
bta-miR-2421	-11.0	336
bta-miR-2421	-10.3	418
bta-miR-2421	-10.2	39
bta-miR-2421	-10.2	621
bta-miR-2422	-27.6	564
bta-miR-2422	-25.4	19
bta-miR-2422	-24.1	378
bta-miR-2422	-23.6	461
bta-miR-2422	-22.9	132
bta-miR-2422	-22.6	257
bta-miR-2422	-22.1	491
bta-miR-2422	-21.8	342
bta-miR-2422	-21.8	316

bta-miR-2422	-21.1	421
bta-miR-2422	-20.9	60
bta-miR-2422	-20.9	161
bta-miR-2422	-20.6	190
bta-miR-2422	-19.6	235
bta-miR-2422	-18.9	87
bta-miR-2422	-18.2	695
bta-miR-2422	-17.6	650
bta-miR-2422	-16.9	291
bta-miR-2422	-16.1	624
bta-miR-2422	-14.6	117
bta-miR-2422	-14.0	515
bta-miR-2422	-13.7	596
bta-miR-2422	-13.6	2
bta-miR-2422	-13.0	39
bta-miR-2422	-12.7	403
bta-miR-2422	-12.3	739
bta-miR-2422	-11.5	537
bta-miR-2422	-11.1	673
bta-miR-2422	-10.5	282
bta-miR-2422	-10.4	481
bta-miR-2422	-10.0	367
bta-miR-2422	-10.0	583
bta-miR-1843	-27.5	605
bta-miR-1843	-26.4	220
bta-miR-1843	-24.8	419
bta-miR-1843	-24.7	350
bta-miR-1843	-22.7	66
bta-miR-1843	-22.5	8
bta-miR-1843	-22.2	266
bta-miR-1843	-21.7	482
bta-miR-1843	-21.3	109
bta-miR-1843	-20.3	155
bta-miR-1843	-19.6	197
bta-miR-1843	-16.9	586
bta-miR-1843	-16.5	35
bta-miR-1843	-16.1	555
bta-miR-1843	-15.9	736
bta-miR-1843	-15.3	406
bta-miR-1843	-15.1	632
bta-miR-1843	-14.0	129
bta-miR-1843	-13.7	463
bta-miR-1843	-12.1	308
bta-miR-1843	-11.9	379
bta-miR-1843	-10.7	698
bta-miR-1843	-10.5	177
bta-miR-1843	-10.1	92
bta-miR-320b	-28.3	161
bta-miR-320b	-23.8	468
bta-miR-320b	-22.9	392
bta-miR-320b	-22.9	244
bta-miR-320b	-22.7	318
bta-miR-320b	-22.4	560
bta-miR-320b	-22.2	116
bta-miR-320b	-21.1	51
bta-miR-320b	-21.1	361
bta-miR-320b	-21.0	207
bta-miR-320b	-20.4	138
bta-miR-320b	-19.9	2

bta-miR-320b	-19.3	425
bta-miR-320b	-18.3	30
bta-miR-320b	-18.2	510
bta-miR-320b	-18.1	636
bta-miR-320b	-17.7	185
bta-miR-320b	-17.3	86
bta-miR-320b	-17.2	669
bta-miR-320b	-15.6	578
bta-miR-320b	-14.7	414
bta-miR-320b	-14.5	712
bta-miR-320b	-14.0	457
bta-miR-320b	-13.9	271
bta-miR-320b	-13.3	594
bta-miR-320b	-12.6	341
bta-miR-320b	-12.2	305
bta-miR-320b	-12.0	374
bta-miR-320b	-11.6	624
bta-miR-320b	-11.1	490
bta-miR-320b	-11.1	236
bta-miR-320b	-11.1	534
bta-miR-320b	-10.4	110
bta-miR-320b	-10.1	286
bta-miR-320b	-10.0	382
bta-miR-2423	-22.2	102
bta-miR-2423	-19.6	154
bta-miR-2423	-18.3	448
bta-miR-2423	-17.9	176
bta-miR-2423	-17.1	41
bta-miR-2423	-16.8	350
bta-miR-2423	-16.2	219
bta-miR-2423	-16.0	371
bta-miR-2423	-15.9	630
bta-miR-2423	-15.4	68
bta-miR-2423	-15.4	16
bta-miR-2423	-14.6	598
bta-miR-2423	-14.1	710
bta-miR-2423	-13.9	557
bta-miR-2423	-13.9	499
bta-miR-2423	-13.7	418
bta-miR-2423	-13.5	613
bta-miR-2423	-13.3	125
bta-miR-2423	-13.2	482
bta-miR-2423	-13.1	269
bta-miR-2423	-12.8	202
bta-miR-2423	-12.4	313
bta-miR-2423	-11.4	575
bta-miR-2423	-11.0	138
bta-miR-2423	-10.5	407
bta-miR-2423	-10.0	301
bta-miR-2423	-10.0	334
bta-miR-2424	-25.9	456
bta-miR-2424	-25.4	563
bta-miR-2424	-24.6	50
bta-miR-2424	-24.3	482
bta-miR-2424	-19.5	85
bta-miR-2424	-18.3	653
bta-miR-2424	-17.8	108
bta-miR-2424	-17.8	222
bta-miR-2424	-17.7	407

bta-miR-2424	-16.2	155
bta-miR-2424	-16.1	17
bta-miR-2424	-15.8	342
bta-miR-2424	-15.0	308
bta-miR-2424	-14.7	381
bta-miR-2424	-14.6	605
bta-miR-2424	-14.6	258
bta-miR-2424	-14.5	184
bta-miR-2424	-14.5	581
bta-miR-2424	-14.4	687
bta-miR-2424	-14.2	125
bta-miR-2424	-13.1	4
bta-miR-2424	-13.1	740
bta-miR-2424	-12.8	539
bta-miR-2424	-11.2	243
bta-miR-2424	-10.1	511
bta-miR-2425	-26.0	418
bta-miR-2425	-24.7	354
bta-miR-2425	-24.5	497
bta-miR-2425	-24.4	81
bta-miR-2425	-23.7	454
bta-miR-2425	-20.9	230
bta-miR-2425	-20.0	618
bta-miR-2425	-19.9	9
bta-miR-2425	-19.8	532
bta-miR-2425	-18.9	573
bta-miR-2425	-17.7	389
bta-miR-2425	-17.1	657
bta-miR-2425	-16.8	55
bta-miR-2425	-16.3	146
bta-miR-2425	-16.1	596
bta-miR-2425	-15.9	263
bta-miR-2425	-15.3	370
bta-miR-2425	-14.7	294
bta-miR-2425	-14.7	709
bta-miR-2425	-14.4	480
bta-miR-2425	-13.6	551
bta-miR-2425	-13.1	311
bta-miR-2425	-13.1	115
bta-miR-2425	-13.1	177
bta-miR-2425	-12.5	206
bta-miR-2425	-11.7	42
bta-miR-2425	-11.6	741
bta-miR-2425	-11.4	680
bta-miR-2425	-10.9	252
bta-miR-2425	-10.8	336
bta-miR-2425	-10.2	328
bta-miR-2425*	-30.9	613
bta-miR-2425*	-27.3	479
bta-miR-2425*	-26.8	414
bta-miR-2425*	-23.3	68
bta-miR-2425*	-23.1	598
bta-miR-2425*	-22.7	12
bta-miR-2425*	-21.3	450
bta-miR-2425*	-18.4	219
bta-miR-2425*	-17.3	265
bta-miR-2425*	-16.2	171
bta-miR-2425*	-15.7	109
bta-miR-2425*	-12.9	573

bta-miR-2425*	-12.3	357
bta-miR-2425*	-11.9	28
bta-miR-2425*	-11.3	202
bta-miR-2425*	-10.9	560
bta-miR-2425*	-10.8	654
bta-miR-2426	-25.1	400
bta-miR-2426	-24.5	32
bta-miR-2426	-23.1	110
bta-miR-2426	-23.1	626
bta-miR-2426	-22.2	140
bta-miR-2426	-21.9	259
bta-miR-2426	-21.3	466
bta-miR-2426	-20.7	191
bta-miR-2426	-20.5	236
bta-miR-2426	-19.7	316
bta-miR-2426	-18.4	691
bta-miR-2426	-17.5	74
bta-miR-2426	-17.5	502
bta-miR-2426	-17.2	567
bta-miR-2426	-17.0	430
bta-miR-2426	-16.9	368
bta-miR-2426	-16.3	665
bta-miR-2426	-15.8	455
bta-miR-2426	-15.5	133
bta-miR-2426	-14.9	5
bta-miR-2426	-13.9	168
bta-miR-2426	-13.9	485
bta-miR-2426	-13.6	552
bta-miR-2426	-13.6	646
bta-miR-2426	-13.2	342
bta-miR-2426	-12.7	596
bta-miR-2426	-11.5	740
bta-miR-2426	-11.0	309
bta-miR-2426	-10.5	390
bta-miR-2426	-10.2	96
bta-miR-2426	-10.2	214
bta-miR-2427	-25.2	623
bta-miR-2427	-22.9	243
bta-miR-2427	-22.2	165
bta-miR-2427	-20.9	361
bta-miR-2427	-20.5	393
bta-miR-2427	-20.4	107
bta-miR-2427	-20.1	429
bta-miR-2427	-20.0	72
bta-miR-2427	-19.6	472
bta-miR-2427	-19.6	205
bta-miR-2427	-18.6	563
bta-miR-2427	-18.5	29
bta-miR-2427	-17.5	576
bta-miR-2427	-16.7	516
bta-miR-2427	-16.4	495
bta-miR-2427	-16.3	342
bta-miR-2427	-16.2	715
bta-miR-2427	-15.8	282
bta-miR-2427	-15.0	8
bta-miR-2427	-14.5	261
bta-miR-2427	-14.1	653
bta-miR-2427	-12.6	125
bta-miR-2427	-12.4	326

bta-miR-2427	-12.4	234
bta-miR-2427	-11.6	698
bta-miR-2427	-11.2	596
bta-miR-2427	-10.9	419
bta-miR-2427	-10.4	97
bta-miR-2427	-10.0	58
bta-miR-2428	-27.4	473
bta-miR-2428	-26.0	563
bta-miR-2428	-25.8	111
bta-miR-2428	-25.7	408
bta-miR-2428	-25.6	139
bta-miR-2428	-23.0	361
bta-miR-2428	-22.7	57
bta-miR-2428	-21.7	261
bta-miR-2428	-21.7	181
bta-miR-2428	-21.3	3
bta-miR-2428	-20.7	308
bta-miR-2428	-20.1	682
bta-miR-2428	-20.0	510
bta-miR-2428	-19.8	636
bta-miR-2428	-19.5	213
bta-miR-2428	-18.0	34
bta-miR-2428	-17.0	457
bta-miR-2428	-16.3	657
bta-miR-2428	-15.8	596
bta-miR-2428	-15.6	88
bta-miR-2428	-15.6	327
bta-miR-2428	-14.8	540
bta-miR-2428	-13.8	169
bta-miR-2428	-13.6	622
bta-miR-2428	-13.0	431
bta-miR-2428	-12.7	132
bta-miR-2428	-12.5	703
bta-miR-2428	-11.9	739
bta-miR-2428	-11.8	286
bta-miR-2428	-11.6	246
bta-miR-2428	-11.6	397
bta-miR-2428	-10.5	202
bta-miR-2429	-22.6	441
bta-miR-2429	-17.7	608
bta-miR-2429	-14.5	174
bta-miR-2429	-13.6	598
bta-miR-2429	-13.5	68
bta-miR-2429	-13.0	222
bta-miR-2429	-11.8	506
bta-miR-2429	-11.8	479
bta-miR-2429	-11.0	371
bta-miR-2429	-11.0	423
bta-miR-2429	-10.9	622
bta-miR-2429	-10.3	268
bta-miR-2429	-10.0	83
bta-miR-2430	-28.7	576
bta-miR-2430	-28.4	462
bta-miR-2430	-28.3	99
bta-miR-2430	-28.1	402
bta-miR-2430	-27.4	342
bta-miR-2430	-25.8	176
bta-miR-2430	-25.6	619
bta-miR-2430	-25.1	237

bta-miR-2430	-23.7	505
bta-miR-2430	-23.6	69
bta-miR-2430	-23.3	14
bta-miR-2430	-22.2	134
bta-miR-2430	-21.5	309
bta-miR-2430	-19.9	558
bta-miR-2430	-19.9	425
bta-miR-2430	-17.8	709
bta-miR-2430	-17.6	204
bta-miR-2430	-17.5	262
bta-miR-2430	-16.8	693
bta-miR-2430	-14.5	57
bta-miR-2430	-14.5	662
bta-miR-2430	-14.4	381
bta-miR-2430	-13.9	167
bta-miR-2430	-13.6	486
bta-miR-2430	-13.2	640
bta-miR-2430	-12.9	3
bta-miR-2430	-12.6	281
bta-miR-2430	-12.2	451
bta-miR-2430	-11.6	600
bta-miR-2430	-11.2	741
bta-miR-2430	-10.6	51
bta-miR-2430	-10.4	436
bta-miR-2430	-10.2	330
bta-miR-2430	-10.1	540
bta-miR-2430	-10.1	682
bta-miR-2431*	-20.2	623
bta-miR-2431*	-19.8	353
bta-miR-2431*	-19.3	130
bta-miR-2431*	-18.9	533
bta-miR-2431*	-18.3	416
bta-miR-2431*	-18.3	648
bta-miR-2431*	-18.1	9
bta-miR-2431*	-17.6	579
bta-miR-2431*	-17.3	52
bta-miR-2431*	-17.0	95
bta-miR-2431*	-16.7	274
bta-miR-2431*	-16.7	466
bta-miR-2431*	-16.6	377
bta-miR-2431*	-16.1	233
bta-miR-2431*	-16.0	689
bta-miR-2431*	-15.7	513
bta-miR-2431*	-15.6	166
bta-miR-2431*	-15.3	314
bta-miR-2431*	-15.0	115
bta-miR-2431*	-15.0	559
bta-miR-2431*	-14.8	488
bta-miR-2431*	-12.4	715
bta-miR-2431*	-11.9	429
bta-miR-2431*	-11.7	24
bta-miR-2431*	-11.4	205
bta-miR-2431	-33.7	597
bta-miR-2431	-30.2	67
bta-miR-2431	-26.6	480
bta-miR-2431	-24.7	421
bta-miR-2431	-24.0	620
bta-miR-2431	-22.8	14
bta-miR-2431	-19.6	252

bta-miR-2431	-16.3	165
bta-miR-2431	-15.8	393
bta-miR-2431	-14.8	573
bta-miR-2431	-13.9	654
bta-miR-2431	-13.5	100
bta-miR-2431	-13.1	215
bta-miR-2431	-12.4	518
bta-miR-2431	-12.1	368
bta-miR-2431	-12.0	349
bta-miR-2431	-11.6	532
bta-miR-2431	-11.5	277
bta-miR-2431	-10.4	379
bta-miR-2431	-10.2	742
bta-miR-677	-24.6	277
bta-miR-677	-23.8	238
bta-miR-677	-23.7	67
bta-miR-677	-23.7	614
bta-miR-677	-23.7	8
bta-miR-677	-21.4	558
bta-miR-677	-21.3	177
bta-miR-677	-21.3	479
bta-miR-677	-20.7	450
bta-miR-677	-19.6	100
bta-miR-677	-19.1	410
bta-miR-677	-17.3	44
bta-miR-677	-16.9	371
bta-miR-677	-16.6	737
bta-miR-677	-15.3	516
bta-miR-677	-14.6	577
bta-miR-677	-13.5	692
bta-miR-677	-13.4	206
bta-miR-677	-13.1	659
bta-miR-677	-13.0	338
bta-miR-677	-12.7	85
bta-miR-677	-12.3	548
bta-miR-677	-12.0	147
bta-miR-677	-11.8	391
bta-miR-677	-10.1	36
bta-miR-677	-10.0	303
bta-miR-2432	-27.9	473
bta-miR-2432	-27.2	73
bta-miR-2432	-27.0	415
bta-miR-2432	-23.7	210
bta-miR-2432	-23.1	563
bta-miR-2432	-22.0	361
bta-miR-2432	-20.7	109
bta-miR-2432	-19.3	162
bta-miR-2432	-19.0	283
bta-miR-2432	-17.4	595
bta-miR-2432	-16.7	192
bta-miR-2432	-16.6	691
bta-miR-2432	-16.3	3
bta-miR-2432	-15.7	57
bta-miR-2432	-15.3	650
bta-miR-2432	-15.1	455
bta-miR-2432	-14.4	30
bta-miR-2432	-14.3	242
bta-miR-2432	-14.2	520
bta-miR-2432	-14.1	341

bta-miR-2432	-13.8	261
bta-miR-2432	-12.6	49
bta-miR-2432	-12.5	719
bta-miR-2432	-12.4	636
bta-miR-2432	-12.3	406
bta-miR-2432	-12.0	149
bta-miR-2432	-12.0	437
bta-miR-2432	-10.8	510
bta-miR-2432	-10.6	97
bta-miR-2433	-33.4	4
bta-miR-2433	-25.8	207
bta-miR-2433	-25.6	384
bta-miR-2433	-25.5	544
bta-miR-2433	-25.1	459
bta-miR-2433	-25.0	107
bta-miR-2433	-24.3	53
bta-miR-2433	-24.2	342
bta-miR-2433	-23.2	258
bta-miR-2433	-23.0	566
bta-miR-2433	-21.9	297
bta-miR-2433	-21.7	484
bta-miR-2433	-19.6	81
bta-miR-2433	-19.1	596
bta-miR-2433	-18.3	657
bta-miR-2433	-17.0	328
bta-miR-2433	-16.7	144
bta-miR-2433	-16.0	629
bta-miR-2433	-15.5	38
bta-miR-2433	-15.4	522
bta-miR-2433	-15.0	170
bta-miR-2433	-14.1	692
bta-miR-2433	-14.0	582
bta-miR-2433	-12.9	369
bta-miR-2433	-12.6	280
bta-miR-2433	-11.6	740
bta-miR-2433	-10.8	199
bta-miR-2433	-10.5	681
bta-miR-2434	-20.5	20
bta-miR-2434	-19.1	353
bta-miR-2434	-18.0	239
bta-miR-2434	-17.6	149
bta-miR-2434	-17.2	100
bta-miR-2434	-16.7	122
bta-miR-2434	-16.6	168
bta-miR-2434	-16.2	199
bta-miR-2434	-15.0	493
bta-miR-2434	-14.9	301
bta-miR-2434	-14.8	329
bta-miR-2434	-14.4	420
bta-miR-2434	-14.1	271
bta-miR-2434	-14.1	449
bta-miR-2434	-13.7	368
bta-miR-2434	-13.5	141
bta-miR-2434	-13.5	473
bta-miR-2434	-13.3	320
bta-miR-2434	-13.1	38
bta-miR-2434	-13.1	593
bta-miR-2434	-12.6	185
bta-miR-2434	-12.4	573

bta-miR-2434	-11.4	405
bta-miR-2434	-11.3	255
bta-miR-2434	-11.0	90
bta-miR-2434	-10.9	436
bta-miR-2434	-10.9	705
bta-miR-2434	-10.8	620
bta-miR-2434	-10.6	338
bta-miR-2434	-10.5	557
bta-miR-2434	-10.2	628
bta-miR-2434	-10.2	324
bta-miR-2434	-10.1	379
bta-miR-2434	-10.0	654
bta-miR-2435	-20.6	473
bta-miR-2435	-18.8	429
bta-miR-2435	-17.7	8
bta-miR-2435	-17.3	331
bta-miR-2435	-17.1	200
bta-miR-2435	-15.9	67
bta-miR-2435	-15.7	606
bta-miR-2435	-15.6	237
bta-miR-2435	-14.9	276
bta-miR-2435	-14.6	172
bta-miR-2435	-13.7	449
bta-miR-2435	-13.5	126
bta-miR-2435	-13.3	572
bta-miR-2435	-12.4	669
bta-miR-2435	-12.2	30
bta-miR-2435	-12.2	408
bta-miR-2435	-12.2	698
bta-miR-2435	-11.9	520
bta-miR-2435	-11.6	350
bta-miR-2435	-11.6	381
bta-miR-2435	-11.4	51
bta-miR-2435	-10.7	220
bta-miR-2435	-10.1	299
bta-miR-2435	-10.1	109
bta-miR-2436-5p	-32.4	395
bta-miR-2436-5p	-30.5	86
bta-miR-2436-5p	-29.8	361
bta-miR-2436-5p	-28.2	563
bta-miR-2436-5p	-27.4	244
bta-miR-2436-5p	-26.9	30
bta-miR-2436-5p	-25.4	51
bta-miR-2436-5p	-24.7	167
bta-miR-2436-5p	-24.4	457
bta-miR-2436-5p	-23.9	511
bta-miR-2436-5p	-23.0	4
bta-miR-2436-5p	-22.7	425
bta-miR-2436-5p	-21.5	624
bta-miR-2436-5p	-20.9	126
bta-miR-2436-5p	-18.7	211
bta-miR-2436-5p	-17.9	324
bta-miR-2436-5p	-17.9	716
bta-miR-2436-5p	-16.6	596
bta-miR-2436-5p	-15.9	283
bta-miR-2436-5p	-15.0	669
bta-miR-2436-5p	-14.7	488
bta-miR-2436-5p	-14.4	537
bta-miR-2436-5p	-14.0	199

bta-miR-2436-5p	-12.0	345
bta-miR-2436-5p	-11.6	383
bta-miR-2436-5p	-10.4	119
bta-miR-2436-5p	-10.0	313
bta-miR-2436-3p	-34.0	598
bta-miR-2436-3p	-28.9	448
bta-miR-2436-3p	-25.4	408
bta-miR-2436-3p	-23.7	71
bta-miR-2436-3p	-20.8	15
bta-miR-2436-3p	-20.6	266
bta-miR-2436-3p	-20.4	622
bta-miR-2436-3p	-16.6	172
bta-miR-2436-3p	-16.5	494
bta-miR-2436-3p	-15.7	113
bta-miR-2436-3p	-14.0	217
bta-miR-2436-3p	-12.7	566
bta-miR-2436-3p	-12.4	199
bta-miR-2436-3p	-12.1	356
bta-miR-2436-3p	-12.0	542
bta-miR-2436-3p	-10.9	741
bta-miR-2436-3p	-10.7	154
bta-miR-2436-3p	-10.4	240
bta-miR-2284r	-26.0	460
bta-miR-2284r	-23.5	60
bta-miR-2284r	-22.9	401
bta-miR-2284r	-22.2	97
bta-miR-2284r	-20.3	616
bta-miR-2284r	-19.7	235
bta-miR-2284r	-19.6	24
bta-miR-2284r	-17.7	173
bta-miR-2284r	-17.3	4
bta-miR-2284r	-17.1	490
bta-miR-2284r	-17.0	342
bta-miR-2284r	-16.8	566
bta-miR-2284r	-16.7	596
bta-miR-2284r	-15.9	364
bta-miR-2284r	-15.3	692
bta-miR-2284r	-15.1	140
bta-miR-2284r	-15.1	650
bta-miR-2284r	-14.4	437
bta-miR-2284r	-14.1	730
bta-miR-2284r	-13.0	309
bta-miR-2284r	-12.9	204
bta-miR-2284r	-12.6	277
bta-miR-2284r	-11.8	541
bta-miR-2284r	-11.2	126
bta-miR-2284r	-10.6	51
bta-miR-2284r	-10.3	119
bta-miR-2437	-19.9	218
bta-miR-2437	-19.2	611
bta-miR-2437	-18.8	102
bta-miR-2437	-18.8	445
bta-miR-2437	-16.7	174
bta-miR-2437	-16.3	371
bta-miR-2437	-15.7	479
bta-miR-2437	-15.4	16
bta-miR-2437	-14.9	268
bta-miR-2437	-14.6	41
bta-miR-2437	-13.5	153

bta-miR-2437	-13.0	65
bta-miR-2437	-12.7	350
bta-miR-2437	-12.5	333
bta-miR-2437	-12.5	202
bta-miR-2437	-12.4	598
bta-miR-2437	-12.0	405
bta-miR-2437	-11.9	557
bta-miR-2437	-11.9	414
bta-miR-2437	-11.8	124
bta-miR-2437	-11.5	631
bta-miR-2437	-11.5	736
bta-miR-2437	-11.3	312
bta-miR-2437	-10.3	710
bta-miR-2437	-10.3	574
bta-miR-2437	-10.1	79
bta-miR-2437	-10.0	464
bta-miR-2438	-30.9	8
bta-miR-2438	-30.7	108
bta-miR-2438	-28.8	154
bta-miR-2438	-27.0	407
bta-miR-2438	-26.8	450
bta-miR-2438	-26.5	575
bta-miR-2438	-26.4	68
bta-miR-2438	-25.4	232
bta-miR-2438	-23.1	37
bta-miR-2438	-23.1	605
bta-miR-2438	-22.4	316
bta-miR-2438	-22.1	494
bta-miR-2438	-21.8	270
bta-miR-2438	-21.6	358
bta-miR-2438	-20.5	198
bta-miR-2438	-18.7	389
bta-miR-2438	-18.7	479
bta-miR-2438	-18.7	631
bta-miR-2438	-16.7	555
bta-miR-2438	-15.2	139
bta-miR-2438	-13.1	344
bta-miR-2438	-12.8	687
bta-miR-2438	-12.2	98
bta-miR-2438	-11.7	249
bta-miR-2438	-11.2	737
bta-miR-2438	-10.0	263
bta-miR-2439*	-30.7	1
bta-miR-2439*	-24.0	404
bta-miR-2439*	-23.9	240
bta-miR-2439*	-23.8	450
bta-miR-2439*	-22.7	93
bta-miR-2439*	-22.1	69
bta-miR-2439*	-21.9	598
bta-miR-2439*	-21.9	202
bta-miR-2439*	-20.8	119
bta-miR-2439*	-20.3	350
bta-miR-2439*	-19.3	615
bta-miR-2439*	-19.1	165
bta-miR-2439*	-18.8	40
bta-miR-2439*	-18.7	565
bta-miR-2439*	-18.1	499
bta-miR-2439*	-16.2	147
bta-miR-2439*	-15.9	655

bta-miR-2439*	-15.5	26
bta-miR-2439*	-14.7	298
bta-miR-2439*	-14.5	425
bta-miR-2439*	-13.2	384
bta-miR-2439*	-13.1	177
bta-miR-2439*	-11.7	524
bta-miR-2439*	-11.2	730
bta-miR-2439*	-10.6	580
bta-miR-2439*	-10.5	228
bta-miR-2439*	-10.3	438
bta-miR-2439*	-10.2	548
bta-miR-2439	-27.7	561
bta-miR-2439	-23.3	425
bta-miR-2439	-21.0	206
bta-miR-2439	-19.7	81
bta-miR-2439	-19.5	360
bta-miR-2439	-18.4	398
bta-miR-2439	-18.3	666
bta-miR-2439	-18.3	5
bta-miR-2439	-17.8	51
bta-miR-2439	-17.4	519
bta-miR-2439	-16.5	117
bta-miR-2439	-16.2	244
bta-miR-2439	-16.1	695
bta-miR-2439	-15.6	457
bta-miR-2439	-15.3	307
bta-miR-2439	-15.1	167
bta-miR-2439	-14.9	491
bta-miR-2439	-14.9	624
bta-miR-2439	-14.0	740
bta-miR-2439	-13.9	291
bta-miR-2439	-13.7	30
bta-miR-2439	-13.4	271
bta-miR-2439	-12.3	341
bta-miR-2439	-11.4	109
bta-miR-2439	-11.3	132
bta-miR-2439	-10.4	654
bta-miR-2439	-10.3	257
bta-miR-2439	-10.3	483
bta-miR-2439	-10.0	547
bta-miR-2439	-10.0	156
bta-miR-2440	-25.7	572
bta-miR-2440	-22.0	476
bta-miR-2440	-20.8	34
bta-miR-2440	-19.4	409
bta-miR-2440	-18.4	514
bta-miR-2440	-18.3	391
bta-miR-2440	-18.0	619
bta-miR-2440	-17.9	173
bta-miR-2440	-17.8	259
bta-miR-2440	-17.3	83
bta-miR-2440	-16.7	111
bta-miR-2440	-16.4	127
bta-miR-2440	-15.8	542
bta-miR-2440	-15.8	596
bta-miR-2440	-14.8	286
bta-miR-2440	-14.6	657
bta-miR-2440	-14.5	14
bta-miR-2440	-14.2	236

bta-miR-2440	-13.7	447
bta-miR-2440	-13.5	190
bta-miR-2440	-13.5	70
bta-miR-2440	-12.9	143
bta-miR-2440	-12.2	461
bta-miR-2440	-12.2	368
bta-miR-2440	-12.1	740
bta-miR-2440	-12.1	342
bta-miR-2440	-11.9	502
bta-miR-2440	-11.6	4
bta-miR-2440	-11.5	98
bta-miR-2440	-11.5	613
bta-miR-2440	-10.2	211
bta-miR-2440	-10.2	692
bta-miR-2441	-26.8	401
bta-miR-2441	-25.0	561
bta-miR-2441	-24.4	86
bta-miR-2441	-21.2	512
bta-miR-2441	-21.0	354
bta-miR-2441	-20.9	246
bta-miR-2441	-19.6	190
bta-miR-2441	-19.0	384
bta-miR-2441	-18.0	135
bta-miR-2441	-17.9	466
bta-miR-2441	-17.6	1
bta-miR-2441	-17.5	33
bta-miR-2441	-17.5	110
bta-miR-2441	-16.8	655
bta-miR-2441	-15.7	330
bta-miR-2441	-15.2	55
bta-miR-2441	-14.6	276
bta-miR-2441	-14.5	316
bta-miR-2441	-14.4	625
bta-miR-2441	-14.2	492
bta-miR-2441	-13.9	163
bta-miR-2441	-13.5	545
bta-miR-2441	-13.0	693
bta-miR-2441	-12.0	670
bta-miR-2441	-11.8	232
bta-miR-2441	-11.5	457
bta-miR-2441	-11.4	262
bta-miR-2441	-11.3	739
bta-miR-2441	-10.5	17
bta-miR-2441	-10.2	715
bta-miR-2441	-10.0	299
bta-miR-2442	-31.4	86
bta-miR-2442	-30.1	231
bta-miR-2442	-26.9	17
bta-miR-2442	-26.7	404
bta-miR-2442	-26.6	356
bta-miR-2442	-26.2	463
bta-miR-2442	-26.1	560
bta-miR-2442	-25.5	156
bta-miR-2442	-25.4	493
bta-miR-2442	-23.9	199
bta-miR-2442	-23.3	40
bta-miR-2442	-23.1	126
bta-miR-2442	-22.2	614
bta-miR-2442	-20.8	267

bta-miR-2442	-20.7	319
bta-miR-2442	-17.9	480
bta-miR-2442	-17.6	293
bta-miR-2442	-17.6	183
bta-miR-2442	-17.3	64
bta-miR-2442	-17.2	660
bta-miR-2442	-16.3	631
bta-miR-2442	-15.5	697
bta-miR-2442	-15.0	526
bta-miR-2442	-14.4	258
bta-miR-2442	-14.3	1
bta-miR-2442	-13.8	393
bta-miR-2442	-13.1	449
bta-miR-2442	-12.6	737
bta-miR-2442	-12.3	591
bta-miR-2442	-11.3	120
bta-miR-2442	-10.4	435
bta-miR-2443	-28.7	415
bta-miR-2443	-24.9	654
bta-miR-2443	-24.2	73
bta-miR-2443	-22.8	563
bta-miR-2443	-22.6	261
bta-miR-2443	-22.6	30
bta-miR-2443	-22.4	306
bta-miR-2443	-21.9	473
bta-miR-2443	-21.5	206
bta-miR-2443	-20.8	160
bta-miR-2443	-20.5	593
bta-miR-2443	-20.0	383
bta-miR-2443	-19.6	353
bta-miR-2443	-18.6	494
bta-miR-2443	-17.9	698
bta-miR-2443	-16.8	119
bta-miR-2443	-16.8	529
bta-miR-2443	-15.2	1
bta-miR-2443	-15.0	283
bta-miR-2443	-14.6	457
bta-miR-2443	-13.5	741
bta-miR-2443	-12.9	629
bta-miR-2443	-12.4	59
bta-miR-2443	-11.6	139
bta-miR-2443	-11.3	247
bta-miR-2443	-10.6	328
bta-miR-2443	-10.3	150
bta-miR-2443	-10.3	408
bta-miR-2443	-10.2	673
bta-miR-2284h	-19.6	166
bta-miR-2284h	-19.1	60
bta-miR-2284h	-19.1	616
bta-miR-2284h	-18.6	429
bta-miR-2284h	-18.5	242
bta-miR-2284h	-18.1	406
bta-miR-2284h	-17.6	366
bta-miR-2284h	-16.8	460
bta-miR-2284h	-16.4	490
bta-miR-2284h	-16.3	327
bta-miR-2284h	-16.1	595
bta-miR-2284h	-15.9	108
bta-miR-2284h	-15.0	273

bta-miR-2284h	-15.0	197
bta-miR-2284h	-14.4	689
bta-miR-2284h	-14.4	28
bta-miR-2284h	-14.2	508
bta-miR-2284h	-13.7	11
bta-miR-2284h	-13.2	306
bta-miR-2284h	-11.8	91
bta-miR-2284h	-11.6	229
bta-miR-2284h	-11.5	581
bta-miR-2284h	-11.4	725
bta-miR-2284h	-11.4	669
bta-miR-2284h	-11.2	124
bta-miR-2284h	-11.2	451
bta-miR-2284h	-10.7	558
bta-miR-2284h	-10.7	648
bta-miR-2284h	-10.3	320
bta-miR-2284h	-10.2	635
bta-miR-2284h*	-28.5	614
bta-miR-2284h*	-23.7	44
bta-miR-2284h*	-22.4	464
bta-miR-2284h*	-21.1	252
bta-miR-2284h*	-20.3	436
bta-miR-2284h*	-18.5	576
bta-miR-2284h*	-18.2	73
bta-miR-2284h*	-17.8	8
bta-miR-2284h*	-17.5	395
bta-miR-2284h*	-16.7	277
bta-miR-2284h*	-15.8	482
bta-miR-2284h*	-15.7	645
bta-miR-2284h*	-15.3	147
bta-miR-2284h*	-13.7	97
bta-miR-2284h*	-13.6	525
bta-miR-2284h*	-13.1	20
bta-miR-2284h*	-13.0	202
bta-miR-2284h*	-12.5	551
bta-miR-2284h*	-12.5	235
bta-miR-2284h*	-12.2	453
bta-miR-2284h*	-11.9	692
bta-miR-2284h*	-11.4	419
bta-miR-2284h*	-11.4	735
bta-miR-2284h*	-11.3	604
bta-miR-2284h*	-10.9	121
bta-miR-2284h*	-10.8	371
bta-miR-2444	-17.8	196
bta-miR-2444	-16.3	100
bta-miR-2444	-14.7	371
bta-miR-2444	-14.5	149
bta-miR-2444	-14.0	338
bta-miR-2444	-13.8	171
bta-miR-2444	-13.3	592
bta-miR-2444	-12.7	221
bta-miR-2444	-11.9	65
bta-miR-2444	-11.8	726
bta-miR-2444	-11.7	444
bta-miR-2444	-11.5	46
bta-miR-2444	-11.1	470
bta-miR-2444	-10.9	405
bta-miR-2444	-10.3	268
bta-miR-2445	-29.5	440

bta-miR-2445	-23.1	222
bta-miR-2445	-20.4	608
bta-miR-2445	-16.7	348
bta-miR-2445	-16.7	174
bta-miR-2445	-15.6	101
bta-miR-2445	-14.7	405
bta-miR-2445	-14.5	36
bta-miR-2445	-14.2	470
bta-miR-2445	-13.1	731
bta-miR-2445	-13.0	68
bta-miR-2445	-12.6	196
bta-miR-2445	-12.2	11
bta-miR-2445	-11.8	124
bta-miR-2445	-11.5	268
bta-miR-2445	-11.5	499
bta-miR-2445	-10.4	557
bta-miR-2445	-10.3	598
bta-miR-2445	-10.2	570
bta-miR-2445	-10.1	153
bta-miR-2445	-10.1	652
bta-miR-2445	-10.0	166
bta-miR-2446	-17.0	361
bta-miR-2446	-16.2	12
bta-miR-2446	-15.7	461
bta-miR-2446	-15.0	30
bta-miR-2446	-14.4	429
bta-miR-2446	-14.2	308
bta-miR-2446	-14.2	91
bta-miR-2446	-13.9	167
bta-miR-2446	-13.4	72
bta-miR-2446	-12.9	623
bta-miR-2446	-12.9	244
bta-miR-2446	-12.8	482
bta-miR-2446	-12.7	566
bta-miR-2446	-12.6	669
bta-miR-2446	-12.5	109
bta-miR-2446	-12.1	414
bta-miR-2446	-11.8	514
bta-miR-2446	-11.6	715
bta-miR-2446	-11.5	142
bta-miR-2446	-11.4	596
bta-miR-2446	-11.1	342
bta-miR-2446	-10.8	402
bta-miR-2446	-10.7	119
bta-miR-2446	-10.7	270
bta-miR-2446	-10.5	51
bta-miR-2446	-10.4	532
bta-miR-2446	-10.4	235
bta-miR-2446	-10.2	659
bta-miR-2446	-10.2	473
bta-miR-2446	-10.1	740
bta-miR-2446	-10.1	155
bta-miR-2446	-10.0	424
bta-miR-2447	-31.9	411
bta-miR-2447	-29.6	453
bta-miR-2447	-26.3	559
bta-miR-2447	-26.2	479
bta-miR-2447	-25.9	115
bta-miR-2447	-23.0	155

bta-miR-2447	-22.6	83
bta-miR-2447	-22.3	4
bta-miR-2447	-21.5	205
bta-miR-2447	-21.3	584
bta-miR-2447	-20.6	653
bta-miR-2447	-19.8	41
bta-miR-2447	-19.8	737
bta-miR-2447	-19.4	264
bta-miR-2447	-18.3	606
bta-miR-2447	-17.4	358
bta-miR-2447	-16.5	698
bta-miR-2447	-16.2	508
bta-miR-2447	-16.2	376
bta-miR-2447	-16.0	243
bta-miR-2447	-15.9	308
bta-miR-2447	-15.6	337
bta-miR-2447	-14.3	393
bta-miR-2447	-13.7	172
bta-miR-2447	-12.6	107
bta-miR-2447	-11.6	327
bta-miR-2447	-11.3	198
bta-miR-2447	-11.2	136
bta-miR-2447	-11.0	435
bta-miR-2447	-11.0	472
bta-miR-2447	-10.9	282
bta-miR-2447	-10.2	632
bta-miR-2447	-10.1	520
bta-miR-2447	-10.1	70
bta-miR-2448*	-20.2	543
bta-miR-2448*	-19.2	462
bta-miR-2448*	-18.2	409
bta-miR-2448*	-17.3	81
bta-miR-2448*	-16.2	25
bta-miR-2448*	-15.9	497
bta-miR-2448*	-15.8	658
bta-miR-2448*	-14.8	518
bta-miR-2448*	-14.8	362
bta-miR-2448*	-14.7	741
bta-miR-2448*	-14.1	3
bta-miR-2448*	-14.0	564
bta-miR-2448*	-13.8	248
bta-miR-2448*	-13.8	614
bta-miR-2448*	-12.5	113
bta-miR-2448*	-12.5	266
bta-miR-2448*	-12.3	707
bta-miR-2448*	-12.2	231
bta-miR-2448*	-12.0	12
bta-miR-2448*	-11.4	187
bta-miR-2448*	-11.3	685
bta-miR-2448*	-11.1	598
bta-miR-2448*	-10.6	66
bta-miR-2448*	-10.1	342
bta-miR-2448	-28.6	109
bta-miR-2448	-24.9	473
bta-miR-2448	-23.3	501
bta-miR-2448	-22.3	624
bta-miR-2448	-22.2	230
bta-miR-2448	-21.2	33
bta-miR-2448	-20.7	324

bta-miR-2448	-20.5	361
bta-miR-2448	-19.9	135
bta-miR-2448	-19.4	167
bta-miR-2448	-19.0	429
bta-miR-2448	-18.4	275
bta-miR-2448	-17.6	563
bta-miR-2448	-16.4	586
bta-miR-2448	-16.2	384
bta-miR-2448	-15.6	702
bta-miR-2448	-15.3	305
bta-miR-2448	-15.0	91
bta-miR-2448	-14.7	247
bta-miR-2448	-13.4	67
bta-miR-2448	-13.2	662
bta-miR-2448	-13.1	14
bta-miR-2448	-12.5	192
bta-miR-2448	-11.5	416
bta-miR-2448	-11.0	1
bta-miR-2448	-10.9	734
bta-miR-2449	-31.3	408
bta-miR-2449	-28.1	563
bta-miR-2449	-25.2	73
bta-miR-2449	-25.1	109
bta-miR-2449	-23.4	386
bta-miR-2449	-22.9	167
bta-miR-2449	-22.0	244
bta-miR-2449	-22.0	361
bta-miR-2449	-21.7	30
bta-miR-2449	-19.8	520
bta-miR-2449	-19.0	4
bta-miR-2449	-19.0	461
bta-miR-2449	-18.9	683
bta-miR-2449	-18.9	624
bta-miR-2449	-18.7	654
bta-miR-2449	-18.4	308
bta-miR-2449	-18.0	488
bta-miR-2449	-15.3	283
bta-miR-2449	-15.2	132
bta-miR-2449	-14.8	206
bta-miR-2449	-14.5	58
bta-miR-2449	-14.1	547
bta-miR-2449	-12.7	738
bta-miR-2449	-12.1	235
bta-miR-2449	-12.0	330
bta-miR-2449	-11.6	707
bta-miR-2449	-10.8	587
bta-miR-2449	-10.2	432
bta-miR-2450c	-23.6	420
bta-miR-2450c	-23.4	353
bta-miR-2450c	-21.6	382
bta-miR-2450c	-21.1	199
bta-miR-2450c	-21.0	469
bta-miR-2450c	-20.7	563
bta-miR-2450c	-20.5	106
bta-miR-2450c	-20.2	160
bta-miR-2450c	-19.0	22
bta-miR-2450c	-18.8	66
bta-miR-2450c	-18.6	256
bta-miR-2450c	-18.5	602

bta-miR-2450c	-16.8	278
bta-miR-2450c	-16.1	518
bta-miR-2450c	-15.7	654
bta-miR-2450c	-15.3	307
bta-miR-2450c	-14.9	488
bta-miR-2450c	-14.6	3
bta-miR-2450c	-14.3	739
bta-miR-2450c	-13.9	122
bta-miR-2450c	-13.2	686
bta-miR-2450c	-13.1	244
bta-miR-2450c	-12.3	51
bta-miR-2450c	-12.1	137
bta-miR-2450c	-11.9	545
bta-miR-2450c	-11.7	708
bta-miR-2450c	-11.1	635
bta-miR-2450c	-10.9	326
bta-miR-2450b	-24.4	473
bta-miR-2450b	-23.6	199
bta-miR-2450b	-23.4	106
bta-miR-2450b	-23.2	560
bta-miR-2450b	-22.7	353
bta-miR-2450b	-21.9	379
bta-miR-2450b	-21.5	577
bta-miR-2450b	-20.0	22
bta-miR-2450b	-19.8	66
bta-miR-2450b	-17.9	256
bta-miR-2450b	-17.8	420
bta-miR-2450b	-17.7	172
bta-miR-2450b	-17.5	488
bta-miR-2450b	-17.4	631
bta-miR-2450b	-17.1	304
bta-miR-2450b	-16.7	686
bta-miR-2450b	-16.6	122
bta-miR-2450b	-15.0	654
bta-miR-2450b	-14.9	278
bta-miR-2450b	-14.2	3
bta-miR-2450b	-14.2	531
bta-miR-2450b	-13.8	56
bta-miR-2450b	-13.6	326
bta-miR-2450b	-13.3	729
bta-miR-2450b	-13.3	148
bta-miR-2450b	-13.1	244
bta-miR-2450b	-12.4	594
bta-miR-2450b	-12.1	91
bta-miR-2450b	-11.5	457
bta-miR-2450b	-10.6	621
bta-miR-2450b	-10.2	411
bta-miR-2450b	-10.0	231
bta-miR-2450a	-26.7	106
bta-miR-2450a	-23.3	546
bta-miR-2450a	-23.1	66
bta-miR-2450a	-23.1	415
bta-miR-2450a	-21.8	473
bta-miR-2450a	-21.5	199
bta-miR-2450a	-21.0	20
bta-miR-2450a	-20.7	379
bta-miR-2450a	-19.7	148
bta-miR-2450a	-19.5	353
bta-miR-2450a	-19.3	573

bta-miR-2450a	-18.5	304
bta-miR-2450a	-18.3	631
bta-miR-2450a	-17.9	244
bta-miR-2450a	-17.9	518
bta-miR-2450a	-17.8	126
bta-miR-2450a	-17.6	686
bta-miR-2450a	-16.9	654
bta-miR-2450a	-16.8	278
bta-miR-2450a	-16.4	39
bta-miR-2450a	-14.8	92
bta-miR-2450a	-14.7	172
bta-miR-2450a	-14.5	494
bta-miR-2450a	-13.4	231
bta-miR-2450a	-12.9	3
bta-miR-2450a	-12.8	602
bta-miR-2450a	-12.6	457
bta-miR-2450a	-11.9	739
bta-miR-2450a	-11.2	261
bta-miR-2450a	-11.1	327
bta-miR-2450a	-10.0	702
bta-miR-2451	-26.0	259
bta-miR-2451	-25.3	185
bta-miR-2451	-23.4	467
bta-miR-2451	-23.4	90
bta-miR-2451	-22.3	136
bta-miR-2451	-22.0	624
bta-miR-2451	-21.9	55
bta-miR-2451	-21.8	572
bta-miR-2451	-21.3	236
bta-miR-2451	-20.9	391
bta-miR-2451	-19.2	537
bta-miR-2451	-18.9	317
bta-miR-2451	-18.8	342
bta-miR-2451	-18.6	510
bta-miR-2451	-18.2	5
bta-miR-2451	-17.0	283
bta-miR-2451	-16.8	491
bta-miR-2451	-16.4	690
bta-miR-2451	-15.3	454
bta-miR-2451	-14.5	363
bta-miR-2451	-14.4	655
bta-miR-2451	-14.3	116
bta-miR-2451	-14.2	715
bta-miR-2451	-13.0	169
bta-miR-2451	-12.8	213
bta-miR-2451	-12.7	30
bta-miR-2451	-12.5	559
bta-miR-2451	-12.3	431
bta-miR-2451	-12.0	603
bta-miR-2451	-11.2	83
bta-miR-2451	-10.4	437
bta-miR-2451	-10.2	615
bta-miR-1777a	-36.5	317
bta-miR-1777a	-33.4	237
bta-miR-1777a	-32.7	117
bta-miR-1777a	-28.7	564
bta-miR-1777a	-27.0	2
bta-miR-1777a	-26.8	461
bta-miR-1777a	-26.0	163

bta-miR-1777a	-25.5	87
bta-miR-1777a	-23.4	386
bta-miR-1777a	-23.1	511
bta-miR-1777a	-22.8	342
bta-miR-1777a	-20.9	291
bta-miR-1777a	-19.4	43
bta-miR-1777a	-19.1	271
bta-miR-1777a	-17.5	582
bta-miR-1777a	-15.7	199
bta-miR-1777a	-15.5	624
bta-miR-1777a	-15.4	695
bta-miR-1777a	-15.0	642
bta-miR-1777a	-14.6	483
bta-miR-1777a	-14.1	740
bta-miR-1777a	-13.5	537
bta-miR-1777a	-13.5	368
bta-miR-1777a	-11.9	146
bta-miR-1777a	-11.6	66
bta-miR-2452	-20.9	442
bta-miR-2452	-20.6	608
bta-miR-2452	-19.6	225
bta-miR-2452	-18.9	174
bta-miR-2452	-18.8	479
bta-miR-2452	-18.1	153
bta-miR-2452	-15.9	506
bta-miR-2452	-15.2	371
bta-miR-2452	-15.0	102
bta-miR-2452	-14.7	268
bta-miR-2452	-14.2	16
bta-miR-2452	-13.9	630
bta-miR-2452	-13.8	350
bta-miR-2452	-13.7	65
bta-miR-2452	-12.6	724
bta-miR-2452	-11.8	36
bta-miR-2452	-11.7	598
bta-miR-2452	-11.0	405
bta-miR-2452	-10.7	128
bta-miR-2452	-10.6	334
bta-miR-2452	-10.5	79
bta-miR-2452	-10.4	218
bta-miR-2452	-10.2	574
bta-miR-2453	-36.5	416
bta-miR-2453	-28.0	457
bta-miR-2453	-27.9	206
bta-miR-2453	-27.9	84
bta-miR-2453	-27.3	156
bta-miR-2453	-26.5	561
bta-miR-2453	-25.5	378
bta-miR-2453	-25.0	347
bta-miR-2453	-24.0	51
bta-miR-2453	-22.6	19
bta-miR-2453	-22.5	505
bta-miR-2453	-20.0	658
bta-miR-2453	-19.9	244
bta-miR-2453	-18.9	117
bta-miR-2453	-18.8	619
bta-miR-2453	-17.5	483
bta-miR-2453	-17.3	633
bta-miR-2453	-17.2	686

bta-miR-2453	-16.7	283
bta-miR-2453	-16.2	545
bta-miR-2453	-15.0	185
bta-miR-2453	-13.8	1
bta-miR-2453	-13.6	316
bta-miR-2453	-13.6	739
bta-miR-2453	-13.6	235
bta-miR-2453	-12.9	598
bta-miR-2453	-12.8	436
bta-miR-2453	-12.8	712
bta-miR-2453	-12.0	527
bta-miR-2453	-11.8	449
bta-miR-2453	-11.4	137
bta-miR-2453	-10.9	271
bta-miR-2453	-10.6	328
bta-miR-2453	-10.3	408
bta-miR-2454	-33.4	5
bta-miR-2454	-31.3	399
bta-miR-2454	-30.5	86
bta-miR-2454	-29.6	473
bta-miR-2454	-27.6	212
bta-miR-2454	-27.5	577
bta-miR-2454	-27.2	363
bta-miR-2454	-26.6	58
bta-miR-2454	-25.7	261
bta-miR-2454	-25.1	308
bta-miR-2454	-24.0	544
bta-miR-2454	-23.1	654
bta-miR-2454	-22.7	132
bta-miR-2454	-21.2	167
bta-miR-2454	-21.1	692
bta-miR-2454	-17.5	738
bta-miR-2454	-17.0	454
bta-miR-2454	-15.6	280
bta-miR-2454	-15.6	39
bta-miR-2454	-15.2	631
bta-miR-2454	-13.5	520
bta-miR-2454	-13.2	386
bta-miR-2454	-13.2	566
bta-miR-2454	-12.4	681
bta-miR-2454	-11.7	342
bta-miR-2454	-11.1	193
bta-miR-2454	-10.7	288
bta-miR-2454	-10.7	465
bta-miR-2454*	-28.2	614
bta-miR-2454*	-28.2	202
bta-miR-2454*	-25.5	439
bta-miR-2454*	-21.8	65
bta-miR-2454*	-21.3	479
bta-miR-2454*	-21.1	411
bta-miR-2454*	-20.0	232
bta-miR-2454*	-19.5	265
bta-miR-2454*	-18.8	11
bta-miR-2454*	-18.5	114
bta-miR-2454*	-18.3	348
bta-miR-2454*	-18.1	598
bta-miR-2454*	-16.9	155
bta-miR-2454*	-14.7	559
bta-miR-2454*	-14.5	455

bta-miR-2454*	-13.2	94
bta-miR-2454*	-13.1	425
bta-miR-2454*	-12.4	659
bta-miR-2454*	-12.3	41
bta-miR-2454*	-11.9	242
bta-miR-2454*	-11.8	737
bta-miR-2454*	-11.6	376
bta-miR-2454*	-11.4	575
bta-miR-2454*	-10.5	669
bta-miR-2455	-35.0	421
bta-miR-2455	-28.2	572
bta-miR-2455	-28.0	248
bta-miR-2455	-27.6	133
bta-miR-2455	-26.7	467
bta-miR-2455	-26.4	110
bta-miR-2455	-26.4	362
bta-miR-2455	-25.7	186
bta-miR-2455	-25.0	390
bta-miR-2455	-23.3	86
bta-miR-2455	-23.0	163
bta-miR-2455	-22.9	209
bta-miR-2455	-22.3	33
bta-miR-2455	-21.8	2
bta-miR-2455	-20.5	502
bta-miR-2455	-20.5	321
bta-miR-2455	-18.5	623
bta-miR-2455	-16.4	289
bta-miR-2455	-16.3	561
bta-miR-2455	-15.9	641
bta-miR-2455	-15.8	19
bta-miR-2455	-15.5	665
bta-miR-2455	-14.0	694
bta-miR-2455	-13.1	457
bta-miR-2455	-12.7	73
bta-miR-2455	-12.6	521
bta-miR-2455	-12.4	606
bta-miR-2455	-12.0	541
bta-miR-2455	-11.8	740
bta-miR-2455	-10.8	281
bta-miR-2455	-10.6	242
bta-miR-2455	-10.2	443
bta-miR-339b	-28.5	622
bta-miR-339b	-28.3	472
bta-miR-339b	-27.4	576
bta-miR-339b	-25.1	204
bta-miR-339b	-24.4	418
bta-miR-339b	-22.4	58
bta-miR-339b	-21.1	357
bta-miR-339b	-19.7	242
bta-miR-339b	-18.8	108
bta-miR-339b	-18.1	166
bta-miR-339b	-17.5	394
bta-miR-339b	-16.3	5
bta-miR-339b	-15.4	86
bta-miR-339b	-15.3	281
bta-miR-339b	-14.7	28
bta-miR-339b	-14.2	342
bta-miR-339b	-14.0	502
bta-miR-339b	-13.4	455

bta-miR-339b	-12.8	614
bta-miR-339b	-12.1	190
bta-miR-339b	-11.9	465
bta-miR-339b	-11.9	698
bta-miR-339b	-11.8	559
bta-miR-339b	-11.5	737
bta-miR-339b	-11.1	139
bta-miR-339b	-10.9	129
bta-miR-339b	-10.7	653
bta-miR-1434*	-16.3	576
bta-miR-1434*	-15.8	1
bta-miR-1434*	-14.6	653
bta-miR-1434*	-14.2	50
bta-miR-1434*	-13.9	449
bta-miR-1434*	-13.5	370
bta-miR-1434*	-13.4	279
bta-miR-1434*	-13.2	506
bta-miR-1434*	-13.1	338
bta-miR-1434*	-12.3	224
bta-miR-1434*	-12.3	389
bta-miR-1434*	-12.2	69
bta-miR-1434*	-12.1	614
bta-miR-1434*	-11.5	239
bta-miR-1434*	-11.5	539
bta-miR-1434*	-11.4	39
bta-miR-1434*	-10.6	482
bta-miR-1434*	-10.5	201
bta-miR-1434*	-10.2	175
bta-miR-1434	-19.9	261
bta-miR-1434	-19.4	499
bta-miR-1434	-18.8	622
bta-miR-1434	-17.2	9
bta-miR-1434	-16.3	703
bta-miR-1434	-16.1	474
bta-miR-1434	-16.0	192
bta-miR-1434	-15.9	564
bta-miR-1434	-15.8	362
bta-miR-1434	-14.9	596
bta-miR-1434	-13.9	423
bta-miR-1434	-13.7	163
bta-miR-1434	-13.6	516
bta-miR-1434	-13.1	111
bta-miR-1434	-13.0	386
bta-miR-1434	-13.0	82
bta-miR-1434	-12.8	128
bta-miR-1434	-12.5	237
bta-miR-1434	-12.4	737
bta-miR-1434	-12.2	58
bta-miR-1434	-12.0	309
bta-miR-1434	-11.9	581
bta-miR-1434	-11.9	665
bta-miR-1434	-11.8	402
bta-miR-1434	-11.2	216
bta-miR-1434	-10.7	444
bta-miR-1434	-10.5	351
bta-miR-1434	-10.3	25
bta-miR-1434	-10.2	154
bta-miR-2456	-25.4	354
bta-miR-2456	-24.5	55

bta-miR-2456	-23.3	581
bta-miR-2456	-22.8	210
bta-miR-2456	-22.6	502
bta-miR-2456	-21.4	161
bta-miR-2456	-21.4	249
bta-miR-2456	-21.1	136
bta-miR-2456	-20.9	106
bta-miR-2456	-20.4	468
bta-miR-2456	-19.3	396
bta-miR-2456	-19.1	2
bta-miR-2456	-18.6	33
bta-miR-2456	-17.7	636
bta-miR-2456	-17.1	607
bta-miR-2456	-17.1	420
bta-miR-2456	-16.7	272
bta-miR-2456	-15.5	318
bta-miR-2456	-15.3	189
bta-miR-2456	-14.8	530
bta-miR-2456	-14.5	120
bta-miR-2456	-14.3	624
bta-miR-2456	-14.2	86
bta-miR-2456	-12.9	236
bta-miR-2456	-12.8	712
bta-miR-2456	-12.0	560
bta-miR-2456	-11.8	386
bta-miR-2456	-11.0	330
bta-miR-2456	-10.5	292
bta-miR-2456	-10.3	461
bta-miR-2457	-27.0	65
bta-miR-2457	-25.1	407
bta-miR-2457	-24.6	480
bta-miR-2457	-24.5	12
bta-miR-2457	-23.6	659
bta-miR-2457	-22.6	617
bta-miR-2457	-21.3	265
bta-miR-2457	-18.7	171
bta-miR-2457	-18.6	376
bta-miR-2457	-18.3	559
bta-miR-2457	-15.9	456
bta-miR-2457	-15.8	108
bta-miR-2457	-15.3	429
bta-miR-2457	-15.3	525
bta-miR-2457	-14.6	358
bta-miR-2457	-12.1	214
bta-miR-2457	-11.9	694
bta-miR-2457	-11.8	155
bta-miR-2457	-11.7	741
bta-miR-2457	-11.2	517
bta-miR-2457	-10.4	5
bta-miR-2458	-20.5	492
bta-miR-2458	-19.7	383
bta-miR-2458	-19.6	457
bta-miR-2458	-19.2	126
bta-miR-2458	-18.5	698
bta-miR-2458	-17.3	61
bta-miR-2458	-17.0	275
bta-miR-2458	-17.0	559
bta-miR-2458	-16.6	167
bta-miR-2458	-16.0	353

bta-miR-2458	-15.8	654
bta-miR-2458	-15.1	107
bta-miR-2458	-14.9	205
bta-miR-2458	-14.9	617
bta-miR-2458	-14.6	415
bta-miR-2458	-14.6	297
bta-miR-2458	-14.4	528
bta-miR-2458	-13.8	148
bta-miR-2458	-13.6	22
bta-miR-2458	-13.4	244
bta-miR-2458	-13.3	593
bta-miR-2458	-13.2	739
bta-miR-2458	-12.7	632
bta-miR-2458	-12.0	263
bta-miR-2458	-11.1	341
bta-miR-2458	-11.0	38
bta-miR-2458	-10.9	2
bta-miR-2458	-10.9	520
bta-miR-2458	-10.8	92
bta-miR-2458	-10.7	224
bta-miR-2458	-10.3	680
bta-miR-2458	-10.1	326
bta-miR-2459	-23.7	463
bta-miR-2459	-22.2	26
bta-miR-2459	-21.4	128
bta-miR-2459	-21.2	559
bta-miR-2459	-20.6	243
bta-miR-2459	-20.3	44
bta-miR-2459	-19.8	385
bta-miR-2459	-18.8	355
bta-miR-2459	-18.3	107
bta-miR-2459	-18.0	5
bta-miR-2459	-17.9	205
bta-miR-2459	-17.7	623
bta-miR-2459	-17.7	692
bta-miR-2459	-17.5	72
bta-miR-2459	-17.3	645
bta-miR-2459	-17.2	282
bta-miR-2459	-16.2	165
bta-miR-2459	-16.1	500
bta-miR-2459	-15.5	583
bta-miR-2459	-14.8	422
bta-miR-2459	-14.7	182
bta-miR-2459	-13.7	742
bta-miR-2459	-13.6	484
bta-miR-2459	-13.2	326
bta-miR-2459	-12.4	311
bta-miR-2459	-11.3	90
bta-miR-2459	-11.3	539
bta-miR-2459	-10.5	117
bta-miR-2459	-10.2	714
bta-miR-2459	-10.1	416
bta-miR-2460	-33.7	266
bta-miR-2460	-30.1	7
bta-miR-2460	-27.0	420
bta-miR-2460	-27.0	109
bta-miR-2460	-26.2	66
bta-miR-2460	-25.7	562
bta-miR-2460	-25.2	172

bta-miR-2460	-25.2	603
bta-miR-2460	-24.2	465
bta-miR-2460	-23.4	493
bta-miR-2460	-22.8	359
bta-miR-2460	-20.1	305
bta-miR-2460	-19.5	232
bta-miR-2460	-19.2	38
bta-miR-2460	-19.1	697
bta-miR-2460	-18.3	658
bta-miR-2460	-18.3	445
bta-miR-2460	-17.7	408
bta-miR-2460	-15.6	154
bta-miR-2460	-15.0	388
bta-miR-2460	-14.8	518
bta-miR-2460	-14.5	342
bta-miR-2460	-13.2	215
bta-miR-2460	-12.3	596
bta-miR-2460	-12.1	95
bta-miR-2460	-12.1	256
bta-miR-2460	-12.1	629
bta-miR-2460	-10.9	136
bta-miR-2460	-10.9	325
bta-miR-2460	-10.8	738
bta-miR-1777b	-37.3	317
bta-miR-1777b	-34.8	236
bta-miR-1777b	-33.2	127
bta-miR-1777b	-28.7	564
bta-miR-1777b	-27.8	98
bta-miR-1777b	-27.6	461
bta-miR-1777b	-27.1	163
bta-miR-1777b	-26.5	14
bta-miR-1777b	-24.5	502
bta-miR-1777b	-24.3	396
bta-miR-1777b	-22.8	342
bta-miR-1777b	-21.6	291
bta-miR-1777b	-20.9	73
bta-miR-1777b	-19.2	43
bta-miR-1777b	-18.9	271
bta-miR-1777b	-18.5	374
bta-miR-1777b	-17.3	582
bta-miR-1777b	-16.1	695
bta-miR-1777b	-15.6	642
bta-miR-1777b	-15.6	206
bta-miR-1777b	-14.5	484
bta-miR-1777b	-14.1	2
bta-miR-1777b	-14.1	740
bta-miR-1777b	-13.6	624
bta-miR-1777b	-13.2	524
bta-miR-1777b	-11.9	146
bta-miR-1777b	-11.9	545
bta-miR-1777b	-10.1	368
bta-miR-1777b	-10.1	674
bta-miR-2461-5p	-29.6	596
bta-miR-2461-5p	-26.3	428
bta-miR-2461-5p	-25.3	360
bta-miR-2461-5p	-24.6	473
bta-miR-2461-5p	-24.5	51
bta-miR-2461-5p	-24.3	408
bta-miR-2461-5p	-23.0	7

bta-miR-2461-5p	-23.0	172
bta-miR-2461-5p	-21.6	206
bta-miR-2461-5p	-20.1	81
bta-miR-2461-5p	-19.8	109
bta-miR-2461-5p	-18.4	235
bta-miR-2461-5p	-17.5	562
bta-miR-2461-5p	-17.4	622
bta-miR-2461-5p	-16.8	697
bta-miR-2461-5p	-16.2	307
bta-miR-2461-5p	-16.0	506
bta-miR-2461-5p	-15.6	453
bta-miR-2461-5p	-15.4	30
bta-miR-2461-5p	-15.3	342
bta-miR-2461-5p	-14.3	658
bta-miR-2461-5p	-13.7	277
bta-miR-2461-5p	-12.3	380
bta-miR-2461-5p	-11.3	581
bta-miR-2461-5p	-10.1	143
bta-miR-2461-3p	-27.5	381
bta-miR-2461-3p	-25.7	418
bta-miR-2461-3p	-24.2	616
bta-miR-2461-3p	-21.5	11
bta-miR-2461-3p	-21.1	175
bta-miR-2461-3p	-20.5	269
bta-miR-2461-3p	-20.3	506
bta-miR-2461-3p	-19.7	555
bta-miR-2461-3p	-19.3	66
bta-miR-2461-3p	-19.0	481
bta-miR-2461-3p	-18.4	654
bta-miR-2461-3p	-18.0	154
bta-miR-2461-3p	-17.4	575
bta-miR-2461-3p	-17.3	448
bta-miR-2461-3p	-17.2	532
bta-miR-2461-3p	-16.1	114
bta-miR-2461-3p	-15.6	41
bta-miR-2461-3p	-15.3	300
bta-miR-2461-3p	-14.8	241
bta-miR-2461-3p	-14.7	353
bta-miR-2461-3p	-14.6	687
bta-miR-2461-3p	-13.2	737
bta-miR-2461-3p	-12.7	598
bta-miR-2461-3p	-12.2	403
bta-miR-2461-3p	-11.5	469
bta-miR-2461-3p	-11.3	636
bta-miR-2461-3p	-10.9	344
bta-miR-2461-3p	-10.7	4
bta-miR-2461-3p	-10.0	219
bta-miR-2462	-22.3	177
bta-miR-2462	-19.8	488
bta-miR-2462	-19.1	380
bta-miR-2462	-19.0	663
bta-miR-2462	-18.9	117
bta-miR-2462	-17.9	561
bta-miR-2462	-17.8	688
bta-miR-2462	-16.7	21
bta-miR-2462	-16.0	410
bta-miR-2462	-15.8	463
bta-miR-2462	-15.8	80
bta-miR-2462	-14.9	243

bta-miR-2462	-14.9	149
bta-miR-2462	-14.8	301
bta-miR-2462	-14.7	199
bta-miR-2462	-14.6	43
bta-miR-2462	-14.2	271
bta-miR-2462	-13.9	512
bta-miR-2462	-13.6	353
bta-miR-2462	-13.3	107
bta-miR-2462	-13.0	5
bta-miR-2462	-12.8	535
bta-miR-2462	-12.8	165
bta-miR-2462	-12.6	624
bta-miR-2462	-12.2	482
bta-miR-2462	-12.2	583
bta-miR-2462	-11.7	66
bta-miR-2462	-11.3	229
bta-miR-2462	-11.3	603
bta-miR-2462	-11.1	263
bta-miR-2462	-11.1	136
bta-miR-2462	-10.7	170
bta-miR-2462	-10.5	456
bta-miR-2463	-21.7	351
bta-miR-2463	-21.0	100
bta-miR-2463	-20.5	41
bta-miR-2463	-19.1	507
bta-miR-2463	-18.8	80
bta-miR-2463	-18.2	479
bta-miR-2463	-18.1	154
bta-miR-2463	-17.2	592
bta-miR-2463	-16.6	130
bta-miR-2463	-16.3	238
bta-miR-2463	-16.3	313
bta-miR-2463	-16.0	200
bta-miR-2463	-15.9	10
bta-miR-2463	-15.8	393
bta-miR-2463	-15.7	632
bta-miR-2463	-15.6	269
bta-miR-2463	-15.6	418
bta-miR-2463	-15.1	570
bta-miR-2463	-13.8	737
bta-miR-2463	-13.7	143
bta-miR-2463	-13.7	325
bta-miR-2463	-13.6	454
bta-miR-2463	-12.9	548
bta-miR-2463	-12.7	710
bta-miR-2463	-12.3	665
bta-miR-2463	-10.6	31
bta-miR-2463	-10.6	66
bta-miR-2463	-10.6	673
bta-miR-2463	-10.3	177
bta-miR-2463	-10.0	259
bta-miR-2464-5p	-29.6	44
bta-miR-2464-5p	-24.8	476
bta-miR-2464-5p	-24.7	596
bta-miR-2464-5p	-24.2	4
bta-miR-2464-5p	-23.7	416
bta-miR-2464-5p	-23.2	566
bta-miR-2464-5p	-21.2	112
bta-miR-2464-5p	-20.5	512

bta-miR-2464-5p	-20.1	258
bta-miR-2464-5p	-19.8	173
bta-miR-2464-5p	-19.7	212
bta-miR-2464-5p	-17.2	74
bta-miR-2464-5p	-16.8	386
bta-miR-2464-5p	-16.7	622
bta-miR-2464-5p	-16.4	637
bta-miR-2464-5p	-15.1	342
bta-miR-2464-5p	-15.0	540
bta-miR-2464-5p	-14.7	369
bta-miR-2464-5p	-13.8	146
bta-miR-2464-5p	-13.7	448
bta-miR-2464-5p	-13.2	678
bta-miR-2464-5p	-12.9	660
bta-miR-2464-5p	-12.9	286
bta-miR-2464-5p	-12.6	461
bta-miR-2464-5p	-11.9	691
bta-miR-2464-5p	-11.4	740
bta-miR-2464-5p	-10.4	316
bta-miR-2464-5p	-10.4	502
bta-miR-2464-5p	-10.2	34
bta-miR-2464-3p	-25.4	463
bta-miR-2464-3p	-22.0	410
bta-miR-2464-3p	-21.5	5
bta-miR-2464-3p	-20.8	613
bta-miR-2464-3p	-20.2	386
bta-miR-2464-3p	-20.1	83
bta-miR-2464-3p	-19.6	59
bta-miR-2464-3p	-19.3	167
bta-miR-2464-3p	-19.2	264
bta-miR-2464-3p	-19.1	694
bta-miR-2464-3p	-17.8	427
bta-miR-2464-3p	-17.2	575
bta-miR-2464-3p	-17.0	203
bta-miR-2464-3p	-16.3	663
bta-miR-2464-3p	-16.0	360
bta-miR-2464-3p	-15.9	491
bta-miR-2464-3p	-15.3	552
bta-miR-2464-3p	-15.0	527
bta-miR-2464-3p	-14.2	641
bta-miR-2464-3p	-14.1	117
bta-miR-2464-3p	-13.5	20
bta-miR-2464-3p	-13.4	290
bta-miR-2464-3p	-13.1	225
bta-miR-2464-3p	-13.0	186
bta-miR-2464-3p	-12.3	740
bta-miR-2464-3p	-11.8	516
bta-miR-2464-3p	-10.8	451
bta-miR-2464-3p	-10.4	376
bta-miR-2464-3p	-10.3	146
bta-miR-2464-3p	-10.1	235
bta-miR-2464-3p	-10.1	345
bta-miR-2465	-27.3	409
bta-miR-2465	-26.4	572
bta-miR-2465	-24.9	467
bta-miR-2465	-23.6	619
bta-miR-2465	-23.6	70
bta-miR-2465	-22.8	650
bta-miR-2465	-21.8	514

bta-miR-2465	-21.4	4
bta-miR-2465	-20.6	235
bta-miR-2465	-19.9	97
bta-miR-2465	-18.3	277
bta-miR-2465	-17.8	488
bta-miR-2465	-17.2	152
bta-miR-2465	-16.5	34
bta-miR-2465	-16.5	173
bta-miR-2465	-16.1	532
bta-miR-2465	-15.2	374
bta-miR-2465	-15.2	204
bta-miR-2465	-14.1	596
bta-miR-2465	-13.3	692
bta-miR-2465	-13.1	357
bta-miR-2465	-13.1	739
bta-miR-2465	-12.2	452
bta-miR-2465	-12.2	670
bta-miR-2465	-11.9	120
bta-miR-2465	-10.9	607
bta-miR-2465	-10.7	427
bta-miR-2466-5p	-30.4	22
bta-miR-2466-5p	-29.7	114
bta-miR-2466-5p	-29.0	353
bta-miR-2466-5p	-28.0	381
bta-miR-2466-5p	-26.7	582
bta-miR-2466-5p	-24.3	257
bta-miR-2466-5p	-24.0	160
bta-miR-2466-5p	-23.4	84
bta-miR-2466-5p	-22.2	425
bta-miR-2466-5p	-21.8	56
bta-miR-2466-5p	-21.7	469
bta-miR-2466-5p	-21.6	319
bta-miR-2466-5p	-21.5	137
bta-miR-2466-5p	-21.0	560
bta-miR-2466-5p	-20.6	1
bta-miR-2466-5p	-20.3	488
bta-miR-2466-5p	-19.9	654
bta-miR-2466-5p	-19.8	238
bta-miR-2466-5p	-18.5	300
bta-miR-2466-5p	-18.5	185
bta-miR-2466-5p	-17.2	696
bta-miR-2466-5p	-16.5	206
bta-miR-2466-5p	-16.3	454
bta-miR-2466-5p	-16.1	624
bta-miR-2466-5p	-15.6	329
bta-miR-2466-5p	-14.0	516
bta-miR-2466-5p	-13.7	739
bta-miR-2466-5p	-13.5	535
bta-miR-2466-5p	-11.7	109
bta-miR-2466-5p	-10.6	283
bta-miR-2466-5p	-10.5	606
bta-miR-2466-5p	-10.3	51
bta-miR-2466-3p	-30.5	61
bta-miR-2466-3p	-29.6	432
bta-miR-2466-3p	-28.7	403
bta-miR-2466-3p	-27.9	598
bta-miR-2466-3p	-27.0	92
bta-miR-2466-3p	-25.4	3
bta-miR-2466-3p	-22.4	216

bta-miR-2466-3p	-21.6	257
bta-miR-2466-3p	-19.9	464
bta-miR-2466-3p	-19.9	561
bta-miR-2466-3p	-19.4	170
bta-miR-2466-3p	-19.4	730
bta-miR-2466-3p	-18.3	356
bta-miR-2466-3p	-16.3	495
bta-miR-2466-3p	-14.9	423
bta-miR-2466-3p	-14.6	630
bta-miR-2466-3p	-13.2	125
bta-miR-2466-3p	-13.1	650
bta-miR-2466-3p	-12.5	27
bta-miR-2466-3p	-11.8	347
bta-miR-2466-3p	-11.6	188
bta-miR-2466-3p	-11.0	235
bta-miR-2466-3p	-10.9	147
bta-miR-2467*	-34.4	616
bta-miR-2467*	-30.4	63
bta-miR-2467*	-29.1	411
bta-miR-2467*	-28.7	447
bta-miR-2467*	-25.5	4
bta-miR-2467*	-22.9	494
bta-miR-2467*	-22.1	154
bta-miR-2467*	-20.9	107
bta-miR-2467*	-20.6	575
bta-miR-2467*	-19.9	232
bta-miR-2467*	-18.2	653
bta-miR-2467*	-16.2	119
bta-miR-2467*	-16.0	29
bta-miR-2467*	-15.8	482
bta-miR-2467*	-15.6	559
bta-miR-2467*	-15.4	358
bta-miR-2467*	-15.3	515
bta-miR-2467*	-15.2	198
bta-miR-2467*	-13.3	279
bta-miR-2467*	-12.6	50
bta-miR-2467*	-12.5	425
bta-miR-2467*	-12.3	741
bta-miR-2467*	-11.5	698
bta-miR-2467*	-11.0	539
bta-miR-2467*	-10.9	669
bta-miR-2467*	-10.5	183
bta-miR-2467*	-10.3	86
bta-miR-2467	-36.4	107
bta-miR-2467	-35.2	407
bta-miR-2467	-30.4	64
bta-miR-2467	-30.1	234
bta-miR-2467	-28.8	361
bta-miR-2467	-26.9	165
bta-miR-2467	-26.6	563
bta-miR-2467	-26.2	472
bta-miR-2467	-26.1	29
bta-miR-2467	-25.9	270
bta-miR-2467	-23.7	499
bta-miR-2467	-21.6	623
bta-miR-2467	-20.3	198
bta-miR-2467	-19.8	653
bta-miR-2467	-19.1	4
bta-miR-2467	-17.3	135

bta-miR-2467	-17.3	321
bta-miR-2467	-17.3	451
bta-miR-2467	-16.2	394
bta-miR-2467	-14.7	524
bta-miR-2467	-14.3	698
bta-miR-2467	-13.6	737
bta-miR-2467	-12.9	146
bta-miR-2467	-12.9	435
bta-miR-2467	-12.8	214
bta-miR-2467	-12.6	596
bta-miR-2467	-12.4	96
bta-miR-2467	-11.6	543
bta-miR-2467	-10.6	345
bta-miR-2468	-20.0	452
bta-miR-2468	-19.3	372
bta-miR-2468	-19.0	563
bta-miR-2468	-18.8	497
bta-miR-2468	-17.6	66
bta-miR-2468	-16.8	206
bta-miR-2468	-16.8	270
bta-miR-2468	-16.5	1
bta-miR-2468	-16.0	634
bta-miR-2468	-15.8	413
bta-miR-2468	-14.2	122
bta-miR-2468	-14.1	668
bta-miR-2468	-13.8	43
bta-miR-2468	-13.5	176
bta-miR-2468	-13.4	94
bta-miR-2468	-13.2	301
bta-miR-2468	-12.9	337
bta-miR-2468	-12.9	539
bta-miR-2468	-12.7	605
bta-miR-2468	-12.5	476
bta-miR-2468	-12.5	240
bta-miR-2468	-12.4	155
bta-miR-2468	-12.0	588
bta-miR-2468	-11.9	711
bta-miR-2468	-11.9	738
bta-miR-2469	-28.4	471
bta-miR-2469	-27.3	615
bta-miR-2469	-24.2	418
bta-miR-2469	-23.0	65
bta-miR-2469	-22.4	242
bta-miR-2469	-22.0	558
bta-miR-2469	-21.5	4
bta-miR-2469	-20.7	342
bta-miR-2469	-20.6	108
bta-miR-2469	-19.9	166
bta-miR-2469	-17.9	502
bta-miR-2469	-17.6	652
bta-miR-2469	-17.0	84
bta-miR-2469	-16.6	443
bta-miR-2469	-16.6	36
bta-miR-2469	-16.4	394
bta-miR-2469	-16.1	269
bta-miR-2469	-16.1	596
bta-miR-2469	-16.1	124
bta-miR-2469	-14.3	154
bta-miR-2469	-13.3	202

bta-miR-2469	-13.0	223
bta-miR-2469	-12.3	323
bta-miR-2469	-12.1	308
bta-miR-2469	-12.0	30
bta-miR-2469	-11.6	696
bta-miR-2469	-11.5	736
bta-miR-2469	-10.9	380
bta-miR-2469	-10.2	281
bta-miR-2469	-10.0	548
bta-miR-2470	-23.7	94
bta-miR-2470	-22.2	463
bta-miR-2470	-21.7	351
bta-miR-2470	-20.4	128
bta-miR-2470	-18.8	592
bta-miR-2470	-18.6	228
bta-miR-2470	-18.3	41
bta-miR-2470	-17.5	418
bta-miR-2470	-17.4	495
bta-miR-2470	-17.1	203
bta-miR-2470	-16.9	393
bta-miR-2470	-16.4	154
bta-miR-2470	-16.2	10
bta-miR-2470	-15.6	563
bta-miR-2470	-15.6	313
bta-miR-2470	-15.6	635
bta-miR-2470	-15.2	269
bta-miR-2470	-14.5	661
bta-miR-2470	-13.6	188
bta-miR-2470	-13.3	447
bta-miR-2470	-12.7	481
bta-miR-2470	-12.7	575
bta-miR-2470	-12.6	376
bta-miR-2470	-12.4	737
bta-miR-2470	-12.3	71
bta-miR-2470	-12.0	710
bta-miR-2470	-11.9	613
bta-miR-2470	-11.7	338
bta-miR-2471	-25.6	474
bta-miR-2471	-25.5	213
bta-miR-2471	-22.5	430
bta-miR-2471	-21.4	342
bta-miR-2471	-19.8	261
bta-miR-2471	-19.7	58
bta-miR-2471	-19.6	625
bta-miR-2471	-19.0	110
bta-miR-2471	-18.8	176
bta-miR-2471	-18.6	396
bta-miR-2471	-18.3	598
bta-miR-2471	-17.0	693
bta-miR-2471	-16.3	564
bta-miR-2471	-15.9	14
bta-miR-2471	-14.2	730
bta-miR-2471	-13.6	201
bta-miR-2471	-13.4	369
bta-miR-2471	-13.3	239
bta-miR-2471	-13.0	290
bta-miR-2471	-12.4	581
bta-miR-2471	-12.4	657
bta-miR-2471	-12.0	614

bta-miR-2471	-11.2	94
bta-miR-2471	-10.7	521
bta-miR-2471	-10.6	463
bta-miR-2471	-10.0	1
bta-miR-2471*	-30.1	80
bta-miR-2471*	-23.2	414
bta-miR-2471*	-23.0	108
bta-miR-2471*	-22.1	12
bta-miR-2471*	-21.3	198
bta-miR-2471*	-21.0	265
bta-miR-2471*	-20.8	605
bta-miR-2471*	-20.1	623
bta-miR-2471*	-19.9	65
bta-miR-2471*	-17.1	472
bta-miR-2471*	-16.6	448
bta-miR-2471*	-16.5	171
bta-miR-2471*	-16.5	559
bta-miR-2471*	-15.8	37
bta-miR-2471*	-14.4	381
bta-miR-2471*	-14.1	653
bta-miR-2471*	-13.9	506
bta-miR-2471*	-13.1	282
bta-miR-2471*	-12.7	737
bta-miR-2471*	-11.7	228
bta-miR-2471*	-11.5	698
bta-miR-2471*	-11.4	539
bta-miR-2471*	-11.3	586
bta-miR-2471*	-11.2	2
bta-miR-2471*	-10.8	155
bta-miR-2471*	-10.6	243
bta-miR-2471*	-10.2	520
bta-miR-2472	-29.1	86
bta-miR-2472	-26.1	419
bta-miR-2472	-25.5	308
bta-miR-2472	-25.1	384
bta-miR-2472	-23.5	353
bta-miR-2472	-23.2	457
bta-miR-2472	-23.0	233
bta-miR-2472	-22.6	488
bta-miR-2472	-22.1	22
bta-miR-2472	-21.9	126
bta-miR-2472	-20.2	183
bta-miR-2472	-20.0	560
bta-miR-2472	-18.8	156
bta-miR-2472	-18.7	249
bta-miR-2472	-18.6	326
bta-miR-2472	-17.4	594
bta-miR-2472	-17.2	705
bta-miR-2472	-14.5	514
bta-miR-2472	-14.0	51
bta-miR-2472	-13.8	654
bta-miR-2472	-13.6	624
bta-miR-2472	-13.5	2
bta-miR-2472	-13.1	684
bta-miR-2472	-11.1	339
bta-miR-2472	-10.5	406
bta-miR-2473	-23.4	450
bta-miR-2473	-22.2	663
bta-miR-2473	-21.9	616

bta-miR-2473	-20.4	65
bta-miR-2473	-20.1	418
bta-miR-2473	-19.4	506
bta-miR-2473	-18.5	174
bta-miR-2473	-17.6	101
bta-miR-2473	-17.2	575
bta-miR-2473	-17.1	348
bta-miR-2473	-16.8	227
bta-miR-2473	-15.8	394
bta-miR-2473	-15.6	202
bta-miR-2473	-15.4	5
bta-miR-2473	-15.0	558
bta-miR-2473	-14.7	630
bta-miR-2473	-14.5	268
bta-miR-2473	-14.4	28
bta-miR-2473	-14.3	371
bta-miR-2473	-14.3	539
bta-miR-2473	-14.1	736
bta-miR-2473	-13.4	694
bta-miR-2473	-13.2	439
bta-miR-2473	-13.0	487
bta-miR-2473	-12.7	12
bta-miR-2473	-12.4	126
bta-miR-2473	-12.4	598
bta-miR-2473	-12.2	154
bta-miR-2473	-11.9	652
bta-miR-2473	-11.5	51
bta-miR-2473	-11.4	609
bta-miR-2473	-11.1	307
bta-miR-2473	-11.0	386
bta-miR-2473	-10.6	710
bta-miR-2473	-10.5	496
bta-miR-2473	-10.5	567
bta-miR-2474	-31.5	400
bta-miR-2474	-27.0	578
bta-miR-2474	-26.6	90
bta-miR-2474	-26.4	511
bta-miR-2474	-22.5	55
bta-miR-2474	-22.5	5
bta-miR-2474	-22.0	461
bta-miR-2474	-21.7	139
bta-miR-2474	-21.1	236
bta-miR-2474	-20.7	713
bta-miR-2474	-20.5	259
bta-miR-2474	-20.1	637
bta-miR-2474	-20.0	190
bta-miR-2474	-18.3	342
bta-miR-2474	-18.1	617
bta-miR-2474	-18.0	491
bta-miR-2474	-17.9	310
bta-miR-2474	-17.7	549
bta-miR-2474	-17.4	117
bta-miR-2474	-16.5	426
bta-miR-2474	-16.0	212
bta-miR-2474	-15.1	692
bta-miR-2474	-13.9	181
bta-miR-2474	-13.8	386
bta-miR-2474	-13.5	605
bta-miR-2474	-13.4	170

bta-miR-2474	-13.2	284
bta-miR-2474	-11.8	675
bta-miR-2474	-10.9	85
bta-miR-2474	-10.8	450
bta-miR-2475	-22.0	38
bta-miR-2475	-20.3	362
bta-miR-2475	-19.8	560
bta-miR-2475	-19.3	488
bta-miR-2475	-18.2	654
bta-miR-2475	-18.2	185
bta-miR-2475	-18.0	266
bta-miR-2475	-17.6	116
bta-miR-2475	-16.9	585
bta-miR-2475	-16.7	61
bta-miR-2475	-15.8	13
bta-miR-2475	-15.7	403
bta-miR-2475	-15.7	457
bta-miR-2475	-15.1	136
bta-miR-2475	-15.1	617
bta-miR-2475	-14.2	515
bta-miR-2475	-13.4	81
bta-miR-2475	-12.9	342
bta-miR-2475	-12.6	292
bta-miR-2475	-12.6	682
bta-miR-2475	-12.0	430
bta-miR-2475	-11.5	743
bta-miR-2475	-11.3	547
bta-miR-2475	-10.6	231
bta-miR-2476	-22.5	413
bta-miR-2476	-22.0	108
bta-miR-2476	-21.8	54
bta-miR-2476	-21.6	472
bta-miR-2476	-21.1	568
bta-miR-2476	-19.0	136
bta-miR-2476	-19.0	364
bta-miR-2476	-17.7	263
bta-miR-2476	-16.4	186
bta-miR-2476	-16.0	622
bta-miR-2476	-15.5	5
bta-miR-2476	-15.3	71
bta-miR-2476	-15.1	19
bta-miR-2476	-14.8	699
bta-miR-2476	-14.4	734
bta-miR-2476	-14.2	318
bta-miR-2476	-14.1	650
bta-miR-2476	-13.7	500
bta-miR-2476	-13.4	340
bta-miR-2476	-13.3	125
bta-miR-2476	-13.1	231
bta-miR-2476	-12.5	385
bta-miR-2476	-12.0	456
bta-miR-2476	-11.7	86
bta-miR-2476	-11.7	611
bta-miR-2476	-11.2	298
bta-miR-2476	-10.6	556
bta-miR-2476	-10.1	520
bta-miR-2284o	-20.9	166
bta-miR-2284o	-20.9	357
bta-miR-2284o	-19.8	102

bta-miR-2284o	-19.0	240
bta-miR-2284o	-18.1	620
bta-miR-2284o	-17.6	429
bta-miR-2284o	-17.5	327
bta-miR-2284o	-17.5	405
bta-miR-2284o	-17.5	28
bta-miR-2284o	-17.2	308
bta-miR-2284o	-16.8	69
bta-miR-2284o	-16.6	470
bta-miR-2284o	-16.2	506
bta-miR-2284o	-15.3	273
bta-miR-2284o	-14.6	188
bta-miR-2284o	-14.4	443
bta-miR-2284o	-14.4	592
bta-miR-2284o	-14.2	557
bta-miR-2284o	-13.9	223
bta-miR-2284o	-13.6	320
bta-miR-2284o	-13.3	129
bta-miR-2284o	-13.2	14
bta-miR-2284o	-12.6	342
bta-miR-2284o	-12.6	141
bta-miR-2284o	-12.4	689
bta-miR-2284o	-12.3	90
bta-miR-2284o	-12.1	580
bta-miR-2284o	-12.0	710
bta-miR-2284o	-10.6	461
bta-miR-2284o	-10.6	486
bta-miR-2284o	-10.4	48
bta-miR-2284o	-10.2	214
bta-miR-2477	-20.8	277
bta-miR-2477	-20.7	8
bta-miR-2477	-20.0	81
bta-miR-2477	-19.7	126
bta-miR-2477	-18.1	38
bta-miR-2477	-17.4	376
bta-miR-2477	-16.7	565
bta-miR-2477	-16.7	450
bta-miR-2477	-16.1	235
bta-miR-2477	-14.9	497
bta-miR-2477	-14.6	645
bta-miR-2477	-14.6	114
bta-miR-2477	-14.5	199
bta-miR-2477	-14.4	702
bta-miR-2477	-13.4	154
bta-miR-2477	-12.7	618
bta-miR-2477	-12.5	266
bta-miR-2477	-12.2	737
bta-miR-2477	-12.1	395
bta-miR-2477	-12.0	518
bta-miR-2477	-12.0	539
bta-miR-2477	-11.7	603
bta-miR-2477	-11.2	173
bta-miR-2477	-11.2	343
bta-miR-2477	-10.7	418
bta-miR-2477	-10.6	481
bta-miR-2477	-10.5	681
bta-miR-2477	-10.5	71
bta-miR-2478	-24.3	609
bta-miR-2478	-22.6	7

bta-miR-2478	-22.1	40
bta-miR-2478	-19.6	257
bta-miR-2478	-19.6	441
bta-miR-2478	-17.8	93
bta-miR-2478	-16.2	493
bta-miR-2478	-15.7	464
bta-miR-2478	-15.7	408
bta-miR-2478	-15.1	576
bta-miR-2478	-14.8	623
bta-miR-2478	-13.8	231
bta-miR-2478	-13.2	170
bta-miR-2478	-13.0	277
bta-miR-2478	-12.4	356
bta-miR-2478	-12.3	644
bta-miR-2478	-12.1	598
bta-miR-2478	-10.8	215
bta-miR-2478	-10.5	543
bta-miR-2478	-10.1	737
bta-miR-2479	-17.3	441
bta-miR-2479	-16.2	348
bta-miR-2479	-14.7	610
bta-miR-2479	-13.9	174
bta-miR-2479	-13.2	225
bta-miR-2479	-13.1	122
bta-miR-2479	-12.7	268
bta-miR-2479	-12.6	598
bta-miR-2479	-12.6	405
bta-miR-2479	-12.5	723
bta-miR-2479	-12.4	479
bta-miR-2479	-11.9	68
bta-miR-2479	-11.5	506
bta-miR-2479	-11.2	100
bta-miR-2479	-10.9	371
bta-miR-2479	-10.8	197
bta-miR-2479	-10.6	332
bta-miR-2480	-16.3	347
bta-miR-2480	-14.5	591
bta-miR-2480	-14.4	263
bta-miR-2480	-13.9	502
bta-miR-2480	-13.7	150
bta-miR-2480	-13.6	476
bta-miR-2480	-13.2	8
bta-miR-2480	-12.7	215
bta-miR-2480	-12.7	452
bta-miR-2480	-11.9	438
bta-miR-2480	-11.4	395
bta-miR-2480	-10.9	371
bta-miR-2480	-10.9	620
bta-miR-2480	-10.8	170
bta-miR-2480	-10.6	68
bta-miR-2480	-10.3	299
bta-miR-2480	-10.0	97
bta-miR-2481	-23.7	162
bta-miR-2481	-21.0	188
bta-miR-2481	-20.7	48
bta-miR-2481	-19.8	361
bta-miR-2481	-19.6	592
bta-miR-2481	-19.4	480
bta-miR-2481	-17.9	507

bta-miR-2481	-17.5	621
bta-miR-2481	-17.4	405
bta-miR-2481	-17.3	241
bta-miR-2481	-17.0	119
bta-miR-2481	-16.7	11
bta-miR-2481	-16.2	429
bta-miR-2481	-15.6	274
bta-miR-2481	-15.5	94
bta-miR-2481	-15.2	338
bta-miR-2481	-14.8	28
bta-miR-2481	-14.4	460
bta-miR-2481	-13.7	216
bta-miR-2481	-13.6	648
bta-miR-2481	-13.5	557
bta-miR-2481	-12.3	142
bta-miR-2481	-12.0	177
bta-miR-2481	-11.5	582
bta-miR-2481	-11.0	110
bta-miR-2481	-10.5	320
bta-miR-2481	-10.3	391
bta-miR-2481	-10.2	135
bta-miR-2481	-10.2	701
bta-miR-2481	-10.1	80
bta-miR-2482	-18.6	599
bta-miR-2482	-17.1	444
bta-miR-2482	-16.3	726
bta-miR-2482	-15.7	365
bta-miR-2482	-15.4	171
bta-miR-2482	-14.7	227
bta-miR-2482	-14.2	93
bta-miR-2482	-13.7	496
bta-miR-2482	-13.4	630
bta-miR-2482	-13.1	333
bta-miR-2482	-13.1	68
bta-miR-2482	-13.0	199
bta-miR-2482	-12.8	270
bta-miR-2482	-12.4	569
bta-miR-2482	-11.9	12
bta-miR-2482	-11.8	119
bta-miR-2482	-11.6	557
bta-miR-2482	-11.3	413
bta-miR-2482	-11.1	149
bta-miR-2482	-10.3	470
bta-miR-2482	-10.2	218
bta-miR-2482	-10.1	615
bta-miR-2483*	-24.7	603
bta-miR-2483*	-22.2	9
bta-miR-2483*	-21.2	419
bta-miR-2483*	-18.8	485
bta-miR-2483*	-18.6	67
bta-miR-2483*	-18.3	623
bta-miR-2483*	-17.6	104
bta-miR-2483*	-17.6	165
bta-miR-2483*	-17.5	651
bta-miR-2483*	-16.5	446
bta-miR-2483*	-15.5	471
bta-miR-2483*	-15.2	559
bta-miR-2483*	-15.1	200
bta-miR-2483*	-14.0	351

bta-miR-2483*	-13.6	238
bta-miR-2483*	-13.6	731
bta-miR-2483*	-13.0	37
bta-miR-2483*	-12.5	369
bta-miR-2483*	-12.2	272
bta-miR-2483*	-12.0	586
bta-miR-2483*	-11.4	407
bta-miR-2483*	-10.7	539
bta-miR-2483*	-10.7	701
bta-miR-2483*	-10.5	516
bta-miR-2483*	-10.2	147
bta-miR-2483*	-10.1	45
bta-miR-2483	-21.6	5
bta-miR-2483	-21.3	396
bta-miR-2483	-20.8	502
bta-miR-2483	-19.1	475
bta-miR-2483	-18.7	107
bta-miR-2483	-18.3	639
bta-miR-2483	-18.2	74
bta-miR-2483	-17.9	421
bta-miR-2483	-17.6	691
bta-miR-2483	-17.5	578
bta-miR-2483	-17.0	211
bta-miR-2483	-17.0	165
bta-miR-2483	-16.3	259
bta-miR-2483	-15.5	25
bta-miR-2483	-15.3	181
bta-miR-2483	-14.1	366
bta-miR-2483	-14.0	45
bta-miR-2483	-13.8	310
bta-miR-2483	-13.7	623
bta-miR-2483	-13.7	456
bta-miR-2483	-13.5	567
bta-miR-2483	-13.2	132
bta-miR-2483	-12.3	603
bta-miR-2483	-12.2	146
bta-miR-2483	-12.2	332
bta-miR-2483	-12.2	665
bta-miR-2483	-12.2	592
bta-miR-2483	-11.9	717
bta-miR-2483	-11.7	99
bta-miR-2483	-10.0	616
bta-miR-2484	-23.4	40
bta-miR-2484	-21.4	606
bta-miR-2484	-20.8	379
bta-miR-2484	-18.6	562
bta-miR-2484	-17.9	266
bta-miR-2484	-17.5	167
bta-miR-2484	-16.5	199
bta-miR-2484	-16.2	644
bta-miR-2484	-15.9	493
bta-miR-2484	-15.4	1
bta-miR-2484	-14.8	457
bta-miR-2484	-14.2	86
bta-miR-2484	-13.8	232
bta-miR-2484	-13.2	293
bta-miR-2484	-13.0	525
bta-miR-2484	-12.7	147
bta-miR-2484	-12.5	20

bta-miR-2484	-12.0	344
bta-miR-2484	-12.0	686
bta-miR-2484	-11.4	729
bta-miR-2484	-11.3	428
bta-miR-2484	-10.8	67
bta-miR-2484	-10.5	478
bta-miR-2484	-10.2	118
bta-miR-664	-32.4	144
bta-miR-664	-27.7	97
bta-miR-664	-25.8	317
bta-miR-664	-25.5	409
bta-miR-664	-23.5	18
bta-miR-664	-23.3	377
bta-miR-664	-23.2	235
bta-miR-664	-23.0	459
bta-miR-664	-22.5	353
bta-miR-664	-21.8	120
bta-miR-664	-21.8	191
bta-miR-664	-21.4	561
bta-miR-664	-21.1	501
bta-miR-664	-19.2	258
bta-miR-664	-18.6	584
bta-miR-664	-18.5	299
bta-miR-664	-18.1	650
bta-miR-664	-16.3	53
bta-miR-664	-15.2	169
bta-miR-664	-15.2	429
bta-miR-664	-14.4	485
bta-miR-664	-14.0	1
bta-miR-664	-13.9	84
bta-miR-664	-13.5	525
bta-miR-664	-13.4	681
bta-miR-664	-13.0	626
bta-miR-664	-11.8	137
bta-miR-664	-11.8	739
bta-miR-664	-11.7	181
bta-miR-664	-10.6	401
bta-miR-664	-10.5	705
bta-miR-2485	-22.1	420
bta-miR-2485	-20.0	189
bta-miR-2485	-19.4	490
bta-miR-2485	-19.2	86
bta-miR-2485	-18.4	457
bta-miR-2485	-18.1	594
bta-miR-2485	-17.9	1
bta-miR-2485	-17.0	116
bta-miR-2485	-16.5	367
bta-miR-2485	-15.7	57
bta-miR-2485	-15.5	560
bta-miR-2485	-14.5	624
bta-miR-2485	-14.5	649
bta-miR-2485	-13.7	307
bta-miR-2485	-13.6	579
bta-miR-2485	-13.4	739
bta-miR-2485	-13.4	167
bta-miR-2485	-12.4	261
bta-miR-2485	-12.2	232
bta-miR-2485	-12.1	695
bta-miR-2485	-11.7	341

bta-miR-2485	-11.6	510
bta-miR-2485	-11.2	408
bta-miR-2485	-10.5	399
bta-miR-2485	-10.1	549
bta-miR-2284e	-17.7	220
bta-miR-2284e	-17.5	443
bta-miR-2284e	-15.5	83
bta-miR-2284e	-15.0	610
bta-miR-2284e	-14.8	173
bta-miR-2284e	-14.3	240
bta-miR-2284e	-13.7	102
bta-miR-2284e	-13.5	405
bta-miR-2284e	-13.4	596
bta-miR-2284e	-13.0	14
bta-miR-2284e	-12.8	506
bta-miR-2284e	-12.8	348
bta-miR-2284e	-12.8	60
bta-miR-2284e	-12.3	460
bta-miR-2284e	-12.2	710
bta-miR-2284e	-11.7	202
bta-miR-2284e	-11.1	309
bta-miR-2284e	-11.0	566
bta-miR-2284e	-10.7	574
bta-miR-2284e	-10.7	689
bta-miR-2284e	-10.6	629
bta-miR-2284e	-10.5	276
bta-miR-2284e	-10.4	735
bta-miR-2486	-25.6	163
bta-miR-2486	-25.3	109
bta-miR-2486	-25.2	362
bta-miR-2486	-23.8	324
bta-miR-2486	-22.2	247
bta-miR-2486	-22.1	649
bta-miR-2486	-22.1	624
bta-miR-2486	-21.5	135
bta-miR-2486	-21.5	474
bta-miR-2486	-21.5	561
bta-miR-2486	-21.3	402
bta-miR-2486	-20.9	510
bta-miR-2486	-20.3	52
bta-miR-2486	-18.3	427
bta-miR-2486	-18.2	31
bta-miR-2486	-17.9	299
bta-miR-2486	-17.3	699
bta-miR-2486	-16.8	5
bta-miR-2486	-16.3	586
bta-miR-2486	-16.1	534
bta-miR-2486	-15.8	207
bta-miR-2486	-12.9	737
bta-miR-2486	-12.7	680
bta-miR-2486	-10.0	463
bta-miR-2486	-10.0	524
bta-miR-2486*	-22.3	238
bta-miR-2486*	-21.0	352
bta-miR-2486*	-20.7	419
bta-miR-2486*	-20.6	653
bta-miR-2486*	-20.4	12
bta-miR-2486*	-20.1	199
bta-miR-2486*	-17.7	274

bta-miR-2486*	-17.5	125
bta-miR-2486*	-17.2	100
bta-miR-2486*	-17.0	171
bta-miR-2486*	-16.9	605
bta-miR-2486*	-16.8	487
bta-miR-2486*	-16.5	694
bta-miR-2486*	-16.3	66
bta-miR-2486*	-16.3	381
bta-miR-2486*	-16.3	456
bta-miR-2486*	-15.4	307
bta-miR-2486*	-14.7	539
bta-miR-2486*	-14.6	83
bta-miR-2486*	-14.3	319
bta-miR-2486*	-13.6	710
bta-miR-2486*	-13.5	137
bta-miR-2486*	-13.2	732
bta-miR-2486*	-13.2	570
bta-miR-2486*	-13.0	2
bta-miR-2486*	-13.0	516
bta-miR-2486*	-12.7	51
bta-miR-2486*	-12.3	149
bta-miR-2486*	-12.1	631
bta-miR-2486*	-11.7	407
bta-miR-2486*	-11.4	118
bta-miR-2486*	-11.4	562
bta-miR-2486*	-10.3	586
bta-miR-2487	-34.7	419
bta-miR-2487	-30.1	155
bta-miR-2487	-28.2	462
bta-miR-2487	-25.4	596
bta-miR-2487	-24.6	229
bta-miR-2487	-24.4	85
bta-miR-2487	-23.6	352
bta-miR-2487	-23.5	568
bta-miR-2487	-23.3	12
bta-miR-2487	-22.1	658
bta-miR-2487	-21.5	482
bta-miR-2487	-21.2	186
bta-miR-2487	-20.1	738
bta-miR-2487	-19.9	318
bta-miR-2487	-19.7	127
bta-miR-2487	-18.7	510
bta-miR-2487	-17.6	387
bta-miR-2487	-16.9	33
bta-miR-2487	-16.1	257
bta-miR-2487	-14.7	206
bta-miR-2487	-14.3	408
bta-miR-2487	-14.0	543
bta-miR-2487	-13.5	72
bta-miR-2487	-12.6	450
bta-miR-2487	-12.5	634
bta-miR-2487	-11.9	116
bta-miR-2487	-11.9	698
bta-miR-2487	-11.5	304
bta-miR-2487	-11.3	1
bta-miR-2487	-11.3	341
bta-miR-2487	-11.2	271
bta-miR-2488	-22.5	610
bta-miR-2488	-20.0	174

bta-miR-2488	-19.3	440
bta-miR-2488	-18.9	68
bta-miR-2488	-17.0	479
bta-miR-2488	-16.2	11
bta-miR-2488	-15.6	598
bta-miR-2488	-15.5	218
bta-miR-2488	-14.8	265
bta-miR-2488	-13.0	349
bta-miR-2488	-12.9	630
bta-miR-2488	-12.9	411
bta-miR-2488	-11.9	506
bta-miR-2488	-11.2	28
bta-miR-2488	-10.4	113
bta-miR-2488	-10.3	241
bta-miR-2488	-10.1	154
bta-miR-1388-5p	-24.6	59
bta-miR-1388-5p	-21.1	214
bta-miR-1388-5p	-21.0	396
bta-miR-1388-5p	-19.4	262
bta-miR-1388-5p	-19.2	7
bta-miR-1388-5p	-19.1	476
bta-miR-1388-5p	-19.0	607
bta-miR-1388-5p	-17.8	449
bta-miR-1388-5p	-17.5	107
bta-miR-1388-5p	-17.1	703
bta-miR-1388-5p	-16.3	35
bta-miR-1388-5p	-15.6	370
bta-miR-1388-5p	-15.4	276
bta-miR-1388-5p	-14.6	578
bta-miR-1388-5p	-14.6	539
bta-miR-1388-5p	-14.5	170
bta-miR-1388-5p	-13.4	432
bta-miR-1388-5p	-13.4	345
bta-miR-1388-5p	-13.3	629
bta-miR-1388-5p	-13.2	656
bta-miR-1388-5p	-12.0	94
bta-miR-1388-5p	-11.8	200
bta-miR-1388-5p	-11.1	740
bta-miR-1388-5p	-10.7	513
bta-miR-1388-5p	-10.6	240
bta-miR-1388-5p	-10.3	26
bta-miR-1388-5p	-10.0	128
bta-miR-1388-3p	-28.6	415
bta-miR-1388-3p	-27.7	109
bta-miR-1388-3p	-27.4	614
bta-miR-1388-3p	-25.3	69
bta-miR-1388-3p	-24.1	478
bta-miR-1388-3p	-23.0	266
bta-miR-1388-3p	-22.6	172
bta-miR-1388-3p	-22.6	583
bta-miR-1388-3p	-22.4	7
bta-miR-1388-3p	-18.0	448
bta-miR-1388-3p	-17.7	359
bta-miR-1388-3p	-16.9	554
bta-miR-1388-3p	-16.6	155
bta-miR-1388-3p	-16.5	38
bta-miR-1388-3p	-16.1	337
bta-miR-1388-3p	-16.1	220
bta-miR-1388-3p	-16.0	658

bta-miR-1388-3p	-14.5	380
bta-miR-1388-3p	-14.4	698
bta-miR-1388-3p	-13.8	602
bta-miR-1388-3p	-12.5	464
bta-miR-1388-3p	-11.7	543
bta-miR-1388-3p	-10.9	730
bta-miR-1388-3p	-10.6	304
bta-miR-1388-3p	-10.2	634
bta-miR-1468	-26.9	597
bta-miR-1468	-25.2	263
bta-miR-1468	-22.2	64
bta-miR-1468	-21.5	198
bta-miR-1468	-19.6	482
bta-miR-1468	-19.5	414
bta-miR-1468	-18.9	617
bta-miR-1468	-18.3	165
bta-miR-1468	-18.2	440
bta-miR-1468	-17.0	5
bta-miR-1468	-16.9	100
bta-miR-1468	-15.8	147
bta-miR-1468	-13.7	653
bta-miR-1468	-13.4	736
bta-miR-1468	-13.3	555
bta-miR-1468	-13.3	351
bta-miR-1468	-12.6	116
bta-miR-1468	-11.5	692
bta-miR-1468	-11.1	238
bta-miR-1468	-10.7	182
bta-miR-1468	-10.6	463
bta-miR-1468	-10.3	296
bta-miR-1468	-10.3	516
bta-miR-424	-21.3	350
bta-miR-424	-18.7	722
bta-miR-424	-18.5	103
bta-miR-424	-15.7	165
bta-miR-424	-15.7	405
bta-miR-424	-15.3	599
bta-miR-424	-15.0	9
bta-miR-424	-14.7	573
bta-miR-424	-14.6	81
bta-miR-424	-14.4	544
bta-miR-424	-14.1	469
bta-miR-424	-13.8	437
bta-miR-424	-12.8	194
bta-miR-424	-12.8	516
bta-miR-424	-12.7	225
bta-miR-424	-12.4	267
bta-miR-424	-11.9	45
bta-miR-424	-11.9	650
bta-miR-424	-11.8	371
bta-miR-424	-11.8	626
bta-miR-424	-10.6	299
bta-miR-424	-10.3	696
bta-miR-424	-10.1	332
bta-miR-424*	-21.8	453
bta-miR-424*	-20.1	516
bta-miR-424*	-19.9	63
bta-miR-424*	-19.5	617
bta-miR-424*	-18.9	265

bta-miR-424*	-18.9	419
bta-miR-424*	-18.7	12
bta-miR-424*	-18.6	559
bta-miR-424*	-18.3	85
bta-miR-424*	-17.2	494
bta-miR-424*	-17.0	41
bta-miR-424*	-16.9	115
bta-miR-424*	-16.9	198
bta-miR-424*	-16.6	358
bta-miR-424*	-15.8	307
bta-miR-424*	-15.7	653
bta-miR-424*	-15.3	376
bta-miR-424*	-15.2	165
bta-miR-424*	-15.2	230
bta-miR-424*	-15.1	575
bta-miR-424*	-15.1	480
bta-miR-424*	-15.0	407
bta-miR-424*	-15.0	183
bta-miR-424*	-14.5	282
bta-miR-424*	-14.1	605
bta-miR-424*	-13.8	107
bta-miR-424*	-12.8	539
bta-miR-424*	-12.5	29
bta-miR-424*	-12.3	429
bta-miR-424*	-11.6	395
bta-miR-424*	-11.4	741
bta-miR-424*	-10.5	698
bta-miR-424*	-10.4	146
bta-miR-424*	-10.3	669
bta-miR-424*	-10.3	635
bta-miR-542-5p	-26.2	352
bta-miR-542-5p	-24.5	412
bta-miR-542-5p	-24.4	19
bta-miR-542-5p	-22.5	583
bta-miR-542-5p	-19.7	81
bta-miR-542-5p	-17.9	130
bta-miR-542-5p	-17.0	378
bta-miR-542-5p	-16.8	292
bta-miR-542-5p	-16.8	605
bta-miR-542-5p	-16.7	657
bta-miR-542-5p	-16.0	249
bta-miR-542-5p	-16.0	225
bta-miR-542-5p	-15.7	494
bta-miR-542-5p	-15.5	190
bta-miR-542-5p	-15.0	437
bta-miR-542-5p	-13.8	542
bta-miR-542-5p	-13.7	629
bta-miR-542-5p	-13.4	155
bta-miR-542-5p	-13.3	323
bta-miR-542-5p	-13.3	329
bta-miR-542-5p	-13.3	711
bta-miR-542-5p	-13.3	738
bta-miR-542-5p	-13.2	567
bta-miR-542-5p	-11.9	267
bta-miR-542-5p	-11.9	40
bta-miR-542-5p	-11.4	62
bta-miR-542-5p	-10.9	368
bta-miR-542-5p	-10.8	475
bta-miR-542-5p	-10.7	336

bta-miR-542-5p	-10.5	1
bta-miR-542-5p	-10.5	240
bta-miR-2917	-17.4	37
bta-miR-2917	-16.7	234
bta-miR-2917	-16.1	89
bta-miR-2917	-15.7	4
bta-miR-2917	-15.6	20
bta-miR-2917	-13.6	379
bta-miR-2917	-13.2	539
bta-miR-2917	-12.9	566
bta-miR-2917	-12.8	479
bta-miR-2917	-12.5	401
bta-miR-2917	-12.4	286
bta-miR-2917	-11.8	261
bta-miR-2917	-11.7	342
bta-miR-2917	-11.5	164
bta-miR-2917	-11.2	596
bta-miR-2917	-11.0	517
bta-miR-2917	-10.9	453
bta-miR-2917	-10.9	191
bta-miR-2917	-10.6	143
bta-miR-2917	-10.4	50
bta-miR-2917	-10.4	114
bta-miR-2917	-10.1	657
bta-miR-2917	-10.0	580
bta-miR-2917	-10.0	639
bta-miR-193a	-25.1	419
bta-miR-193a	-23.8	559
bta-miR-193a	-23.5	12
bta-miR-193a	-21.6	85
bta-miR-193a	-21.1	112
bta-miR-193a	-19.9	184
bta-miR-193a	-19.6	155
bta-miR-193a	-19.2	450
bta-miR-193a	-17.8	243
bta-miR-193a	-17.5	308
bta-miR-193a	-17.3	372
bta-miR-193a	-17.2	220
bta-miR-193a	-17.1	498
bta-miR-193a	-16.2	39
bta-miR-193a	-16.1	738
bta-miR-193a	-16.0	473
bta-miR-193a	-15.5	346
bta-miR-193a	-15.3	72
bta-miR-193a	-15.1	623
bta-miR-193a	-14.5	601
bta-miR-193a	-14.4	698
bta-miR-193a	-14.3	582
bta-miR-193a	-13.9	270
bta-miR-193a	-13.9	659
bta-miR-193a	-12.2	413
bta-miR-193a	-12.0	402
bta-miR-193a	-11.0	520
bta-miR-193a	-10.7	172
bta-miR-193a	-10.4	136
bta-miR-193a	-10.2	324
bta-miR-193a	-10.1	364
bta-miR-669	-25.6	10
bta-miR-669	-25.2	369

bta-miR-669	-24.6	144
bta-miR-669	-23.3	119
bta-miR-669	-22.6	415
bta-miR-669	-20.7	488
bta-miR-669	-19.8	95
bta-miR-669	-19.7	38
bta-miR-669	-19.4	585
bta-miR-669	-19.0	172
bta-miR-669	-18.9	304
bta-miR-669	-18.5	343
bta-miR-669	-18.1	654
bta-miR-669	-17.9	256
bta-miR-669	-17.0	554
bta-miR-669	-15.9	614
bta-miR-669	-15.3	457
bta-miR-669	-13.9	66
bta-miR-669	-13.7	686
bta-miR-669	-13.7	518
bta-miR-669	-13.4	202
bta-miR-669	-12.6	230
bta-miR-669	-11.9	729
bta-miR-669	-10.6	330
bta-miR-669	-10.3	388
bta-miR-669	-10.1	167
bta-miR-2881	-33.5	308
bta-miR-2881	-30.5	237
bta-miR-2881	-30.2	126
bta-miR-2881	-27.6	459
bta-miR-2881	-27.1	415
bta-miR-2881	-26.2	161
bta-miR-2881	-25.9	561
bta-miR-2881	-24.8	85
bta-miR-2881	-24.4	22
bta-miR-2881	-23.6	384
bta-miR-2881	-22.0	342
bta-miR-2881	-20.3	489
bta-miR-2881	-17.9	51
bta-miR-2881	-17.8	684
bta-miR-2881	-17.0	271
bta-miR-2881	-16.9	199
bta-miR-2881	-16.8	2
bta-miR-2881	-16.4	399
bta-miR-2881	-16.2	654
bta-miR-2881	-14.0	115
bta-miR-2881	-13.9	636
bta-miR-2881	-13.6	739
bta-miR-2881	-13.4	705
bta-miR-2881	-13.3	73
bta-miR-2881	-12.4	624
bta-miR-2881	-12.2	287
bta-miR-2881	-12.2	594
bta-miR-2881	-11.6	522
bta-miR-2881	-11.6	541
bta-miR-2881	-10.1	144
bta-miR-2881	-10.1	483
bta-miR-2882	-36.6	614
bta-miR-2882	-31.2	448
bta-miR-2882	-30.2	67
bta-miR-2882	-27.9	410

bta-miR-2882	-22.3	264
bta-miR-2882	-21.5	598
bta-miR-2882	-20.5	10
bta-miR-2882	-19.2	495
bta-miR-2882	-18.3	219
bta-miR-2882	-17.6	170
bta-miR-2882	-16.3	575
bta-miR-2882	-14.6	357
bta-miR-2882	-14.1	429
bta-miR-2882	-12.6	368
bta-miR-2882	-12.0	740
bta-miR-2882	-11.5	142
bta-miR-2882	-11.0	198
bta-miR-2882	-11.0	546
bta-miR-2882	-10.9	663
bta-miR-2882	-10.8	308
bta-miR-2882	-10.5	119
bta-miR-2882	-10.0	566
bta-miR-2883	-22.5	586
bta-miR-2883	-20.6	155
bta-miR-2883	-20.5	464
bta-miR-2883	-19.2	602
bta-miR-2883	-18.8	623
bta-miR-2883	-18.3	419
bta-miR-2883	-17.4	12
bta-miR-2883	-16.7	72
bta-miR-2883	-14.8	483
bta-miR-2883	-14.8	107
bta-miR-2883	-14.5	262
bta-miR-2883	-13.7	211
bta-miR-2883	-13.6	181
bta-miR-2883	-12.9	344
bta-miR-2883	-12.5	377
bta-miR-2883	-11.8	116
bta-miR-2883	-11.8	698
bta-miR-2883	-11.4	139
bta-miR-2883	-11.3	271
bta-miR-2883	-11.3	361
bta-miR-2883	-11.1	310
bta-miR-2883	-11.1	663
bta-miR-2883	-11.0	41
bta-miR-2883	-11.0	86
bta-miR-2883	-11.0	560
bta-miR-2883	-11.0	614
bta-miR-2883	-10.6	294
bta-miR-2883	-10.5	193
bta-miR-2885	-37.6	109
bta-miR-2885	-27.9	30
bta-miR-2885	-27.7	408
bta-miR-2885	-27.3	539
bta-miR-2885	-26.2	73
bta-miR-2885	-24.8	577
bta-miR-2885	-24.4	244
bta-miR-2885	-23.9	167
bta-miR-2885	-23.4	457
bta-miR-2885	-21.7	361
bta-miR-2885	-21.1	12
bta-miR-2885	-19.9	654
bta-miR-2885	-19.1	307

bta-miR-2885	-18.8	624
bta-miR-2885	-18.7	513
bta-miR-2885	-17.8	51
bta-miR-2885	-17.8	483
bta-miR-2885	-16.9	329
bta-miR-2885	-16.9	740
bta-miR-2885	-16.5	134
bta-miR-2885	-16.3	262
bta-miR-2885	-14.6	188
bta-miR-2885	-14.0	431
bta-miR-2885	-13.1	395
bta-miR-2885	-12.7	209
bta-miR-2885	-12.3	605
bta-miR-2885	-11.6	707
bta-miR-2885	-11.2	448
bta-miR-2885	-11.2	502
bta-miR-2885	-11.1	682
bta-miR-2885	-10.9	2
bta-miR-2885	-10.7	96
bta-miR-2885	-10.3	156
bta-miR-2886	-25.6	412
bta-miR-2886	-24.2	70
bta-miR-2886	-24.1	7
bta-miR-2886	-24.0	609
bta-miR-2886	-21.5	473
bta-miR-2886	-21.3	266
bta-miR-2886	-20.2	359
bta-miR-2886	-20.0	25
bta-miR-2886	-19.1	560
bta-miR-2886	-18.5	438
bta-miR-2886	-16.8	659
bta-miR-2886	-16.7	497
bta-miR-2886	-16.3	107
bta-miR-2886	-15.7	738
bta-miR-2886	-14.9	167
bta-miR-2886	-14.9	588
bta-miR-2886	-14.2	628
bta-miR-2886	-12.4	462
bta-miR-2886	-12.3	543
bta-miR-2886	-12.0	185
bta-miR-2886	-11.7	155
bta-miR-2886	-11.3	51
bta-miR-2886	-10.5	88
bta-miR-2886	-10.5	404
bta-miR-2886	-10.1	231
bta-miR-2886	-10.1	524
bta-miR-2887	-30.0	560
bta-miR-2887	-29.9	494
bta-miR-2887	-27.2	418
bta-miR-2887	-26.8	449
bta-miR-2887	-25.6	119
bta-miR-2887	-23.8	66
bta-miR-2887	-23.0	22
bta-miR-2887	-22.8	235
bta-miR-2887	-22.3	341
bta-miR-2887	-21.3	148
bta-miR-2887	-21.0	584
bta-miR-2887	-20.6	319
bta-miR-2887	-20.0	654

bta-miR-2887	-19.8	381
bta-miR-2887	-19.1	102
bta-miR-2887	-18.1	622
bta-miR-2887	-17.8	199
bta-miR-2887	-17.5	172
bta-miR-2887	-17.4	473
bta-miR-2887	-17.0	271
bta-miR-2887	-15.5	698
bta-miR-2887	-14.6	740
bta-miR-2887	-14.2	515
bta-miR-2887	-13.8	606
bta-miR-2887	-13.5	308
bta-miR-2887	-13.1	4
bta-miR-2887	-13.1	401
bta-miR-2887	-12.9	51
bta-miR-2887	-12.1	635
bta-miR-2887	-11.4	371
bta-miR-2887	-10.9	539
bta-miR-2888	-35.8	318
bta-miR-2888	-34.3	127
bta-miR-2888	-27.4	157
bta-miR-2888	-25.3	237
bta-miR-2888	-24.9	29
bta-miR-2888	-23.8	107
bta-miR-2888	-23.1	462
bta-miR-2888	-22.0	572
bta-miR-2888	-20.9	186
bta-miR-2888	-20.6	419
bta-miR-2888	-19.9	360
bta-miR-2888	-19.9	509
bta-miR-2888	-19.0	635
bta-miR-2888	-18.5	85
bta-miR-2888	-18.4	392
bta-miR-2888	-17.6	282
bta-miR-2888	-17.5	688
bta-miR-2888	-14.3	374
bta-miR-2888	-13.8	305
bta-miR-2888	-13.6	556
bta-miR-2888	-13.4	670
bta-miR-2888	-12.9	711
bta-miR-2888	-12.2	1
bta-miR-2888	-11.4	623
bta-miR-2888	-11.3	738
bta-miR-2888	-10.9	144
bta-miR-2888	-10.8	485
bta-miR-2888	-10.0	272
bta-miR-2889	-37.3	617
bta-miR-2889	-34.2	462
bta-miR-2889	-33.9	415
bta-miR-2889	-28.4	61
bta-miR-2889	-26.1	8
bta-miR-2889	-25.5	231
bta-miR-2889	-25.0	155
bta-miR-2889	-24.8	99
bta-miR-2889	-22.9	356
bta-miR-2889	-22.5	586
bta-miR-2889	-21.1	494
bta-miR-2889	-20.1	185
bta-miR-2889	-18.7	38

bta-miR-2889	-17.8	280
bta-miR-2889	-17.7	119
bta-miR-2889	-16.6	560
bta-miR-2889	-16.4	654
bta-miR-2889	-15.4	454
bta-miR-2889	-15.3	308
bta-miR-2889	-14.1	740
bta-miR-2889	-13.4	89
bta-miR-2889	-13.0	515
bta-miR-2889	-12.6	321
bta-miR-2889	-12.6	386
bta-miR-2889	-12.4	141
bta-miR-2889	-12.0	402
bta-miR-2889	-11.8	710
bta-miR-2889	-10.4	342
bta-miR-2890	-38.3	604
bta-miR-2890	-29.1	66
bta-miR-2890	-28.8	11
bta-miR-2890	-28.6	418
bta-miR-2890	-25.8	451
bta-miR-2890	-23.1	234
bta-miR-2890	-22.0	109
bta-miR-2890	-20.8	575
bta-miR-2890	-19.9	154
bta-miR-2890	-18.2	488
bta-miR-2890	-17.2	408
bta-miR-2890	-16.2	548
bta-miR-2890	-15.8	654
bta-miR-2890	-15.6	628
bta-miR-2890	-15.6	358
bta-miR-2890	-15.2	277
bta-miR-2890	-14.9	199
bta-miR-2890	-13.5	737
bta-miR-2890	-10.8	308
bta-miR-2890	-10.7	126
bta-miR-2890	-10.6	517
bta-miR-2890	-10.3	342
bta-miR-2890	-10.0	44
bta-miR-2891	-25.7	155
bta-miR-2891	-23.3	419
bta-miR-2891	-21.7	229
bta-miR-2891	-21.2	85
bta-miR-2891	-19.9	364
bta-miR-2891	-19.2	456
bta-miR-2891	-17.9	179
bta-miR-2891	-17.5	559
bta-miR-2891	-17.2	112
bta-miR-2891	-16.2	12
bta-miR-2891	-16.0	205
bta-miR-2891	-15.6	387
bta-miR-2891	-15.1	509
bta-miR-2891	-15.0	698
bta-miR-2891	-14.8	623
bta-miR-2891	-14.8	482
bta-miR-2891	-14.8	586
bta-miR-2891	-14.2	57
bta-miR-2891	-14.2	302
bta-miR-2891	-14.1	327
bta-miR-2891	-13.8	340

bta-miR-2891	-13.6	243
bta-miR-2891	-13.5	646
bta-miR-2891	-12.9	270
bta-miR-2891	-12.9	131
bta-miR-2891	-12.1	408
bta-miR-2891	-11.8	734
bta-miR-2891	-11.5	36
bta-miR-2891	-11.5	50
bta-miR-2891	-11.1	253
bta-miR-2892	-32.9	448
bta-miR-2892	-29.2	614
bta-miR-2892	-26.3	69
bta-miR-2892	-24.7	12
bta-miR-2892	-24.4	418
bta-miR-2892	-21.5	548
bta-miR-2892	-21.1	371
bta-miR-2892	-20.8	268
bta-miR-2892	-20.7	232
bta-miR-2892	-20.3	114
bta-miR-2892	-19.7	176
bta-miR-2892	-19.5	41
bta-miR-2892	-19.1	577
bta-miR-2892	-18.5	663
bta-miR-2892	-18.2	506
bta-miR-2892	-17.1	359
bta-miR-2892	-15.8	399
bta-miR-2892	-13.9	742
bta-miR-2892	-13.6	156
bta-miR-2892	-13.6	311
bta-miR-2892	-13.5	634
bta-miR-2892	-13.5	209
bta-miR-2892	-13.4	257
bta-miR-2892	-13.4	691
bta-miR-2892	-13.4	130
bta-miR-2892	-13.3	523
bta-miR-2892	-12.6	1
bta-miR-2892	-11.2	342
bta-miR-2892	-10.8	598
bta-miR-2892	-10.4	488
bta-miR-2893	-31.0	110
bta-miR-2893	-23.1	368
bta-miR-2893	-22.6	168
bta-miR-2893	-22.4	260
bta-miR-2893	-22.2	416
bta-miR-2893	-22.0	502
bta-miR-2893	-21.5	14
bta-miR-2893	-21.3	572
bta-miR-2893	-21.0	309
bta-miR-2893	-20.4	461
bta-miR-2893	-20.1	82
bta-miR-2893	-19.6	204
bta-miR-2893	-19.1	627
bta-miR-2893	-16.3	707
bta-miR-2893	-15.5	596
bta-miR-2893	-15.3	139
bta-miR-2893	-15.2	682
bta-miR-2893	-14.9	39
bta-miR-2893	-14.7	481
bta-miR-2893	-14.3	650

bta-miR-2893	-14.1	521
bta-miR-2893	-13.9	71
bta-miR-2893	-13.7	541
bta-miR-2893	-13.1	329
bta-miR-2893	-12.9	236
bta-miR-2893	-11.9	614
bta-miR-2893	-11.7	622
bta-miR-2893	-10.9	1
bta-miR-2893	-10.7	449
bta-miR-2893	-10.6	362
bta-miR-2893	-10.5	58
bta-miR-2893	-10.5	431
bta-miR-2893	-10.4	163
bta-miR-2893	-10.4	740
bta-miR-2894	-31.7	408
bta-miR-2894	-30.9	590
bta-miR-2894	-26.4	56
bta-miR-2894	-24.3	619
bta-miR-2894	-23.9	266
bta-miR-2894	-23.8	93
bta-miR-2894	-22.9	464
bta-miR-2894	-22.7	494
bta-miR-2894	-21.5	441
bta-miR-2894	-21.4	12
bta-miR-2894	-20.2	229
bta-miR-2894	-18.1	161
bta-miR-2894	-17.3	661
bta-miR-2894	-15.3	354
bta-miR-2894	-14.9	40
bta-miR-2894	-14.4	738
bta-miR-2894	-13.8	118
bta-miR-2894	-13.6	569
bta-miR-2894	-12.7	199
bta-miR-2894	-11.6	560
bta-miR-2894	-10.9	429
bta-miR-2894	-10.9	155
bta-miR-2894	-10.2	377
bta-miR-2895	-21.8	433
bta-miR-2895	-21.8	252
bta-miR-2895	-21.1	588
bta-miR-2895	-19.3	15
bta-miR-2895	-17.6	611
bta-miR-2895	-16.7	404
bta-miR-2895	-16.7	93
bta-miR-2895	-16.3	64
bta-miR-2895	-16.0	497
bta-miR-2895	-15.4	178
bta-miR-2895	-14.7	370
bta-miR-2895	-14.3	658
bta-miR-2895	-14.3	478
bta-miR-2895	-14.3	544
bta-miR-2895	-14.2	223
bta-miR-2895	-14.0	70
bta-miR-2895	-13.3	337
bta-miR-2895	-11.8	452
bta-miR-2895	-11.6	1
bta-miR-2895	-11.6	202
bta-miR-2895	-11.3	170
bta-miR-2895	-11.2	633

bta-miR-2895	-11.1	277
bta-miR-2895	-10.9	42
bta-miR-2895	-10.8	730
bta-miR-2895	-10.6	518
bta-miR-2895	-10.0	562
bta-miR-2896	-22.3	417
bta-miR-2896	-19.8	481
bta-miR-2896	-19.2	370
bta-miR-2896	-19.0	71
bta-miR-2896	-18.6	229
bta-miR-2896	-18.3	630
bta-miR-2896	-18.2	113
bta-miR-2896	-17.2	40
bta-miR-2896	-17.2	280
bta-miR-2896	-16.9	560
bta-miR-2896	-16.8	661
bta-miR-2896	-16.4	17
bta-miR-2896	-16.4	1
bta-miR-2896	-15.6	576
bta-miR-2896	-15.4	497
bta-miR-2896	-15.1	299
bta-miR-2896	-15.1	738
bta-miR-2896	-15.1	709
bta-miR-2896	-14.1	176
bta-miR-2896	-14.0	686
bta-miR-2896	-13.4	543
bta-miR-2896	-12.9	350
bta-miR-2896	-12.8	454
bta-miR-2896	-11.9	599
bta-miR-2896	-11.6	617
bta-miR-2896	-11.6	518
bta-miR-2896	-11.5	204
bta-miR-2896	-10.9	155
bta-miR-2896	-10.9	269
bta-miR-2896	-10.9	399
bta-miR-2896	-10.6	130
bta-miR-2897	-28.1	262
bta-miR-2897	-28.0	81
bta-miR-2897	-26.9	310
bta-miR-2897	-26.8	498
bta-miR-2897	-25.4	416
bta-miR-2897	-24.3	617
bta-miR-2897	-23.1	9
bta-miR-2897	-22.7	112
bta-miR-2897	-22.6	448
bta-miR-2897	-22.0	475
bta-miR-2897	-21.0	155
bta-miR-2897	-20.1	39
bta-miR-2897	-19.7	542
bta-miR-2897	-18.9	356
bta-miR-2897	-18.6	231
bta-miR-2897	-18.4	386
bta-miR-2897	-17.9	70
bta-miR-2897	-17.8	176
bta-miR-2897	-17.2	657
bta-miR-2897	-16.2	576
bta-miR-2897	-15.8	686
bta-miR-2897	-14.4	142
bta-miR-2897	-14.2	208

bta-miR-2897	-13.6	708
bta-miR-2897	-13.0	597
bta-miR-2897	-12.5	738
bta-miR-2897	-11.6	329
bta-miR-2897	-10.4	1
bta-miR-2897	-10.4	371
bta-miR-2897	-10.3	286
bta-miR-2898	-25.4	32
bta-miR-2898	-23.5	110
bta-miR-2898	-22.5	133
bta-miR-2898	-22.0	309
bta-miR-2898	-21.7	572
bta-miR-2898	-21.5	461
bta-miR-2898	-20.7	259
bta-miR-2898	-19.6	362
bta-miR-2898	-19.1	416
bta-miR-2898	-17.3	650
bta-miR-2898	-17.2	163
bta-miR-2898	-17.2	322
bta-miR-2898	-17.2	502
bta-miR-2898	-16.9	191
bta-miR-2898	-16.8	90
bta-miR-2898	-16.5	681
bta-miR-2898	-16.4	707
bta-miR-2898	-16.3	626
bta-miR-2898	-15.6	740
bta-miR-2898	-15.2	540
bta-miR-2898	-14.0	521
bta-miR-2898	-13.9	488
bta-miR-2898	-13.5	430
bta-miR-2898	-13.4	14
bta-miR-2898	-13.1	236
bta-miR-2898	-12.9	596
bta-miR-2898	-12.0	60
bta-miR-2898	-11.8	605
bta-miR-2898	-11.2	286
bta-miR-2898	-10.5	177
bta-miR-2898	-10.0	146
bta-miR-2899	-34.2	107
bta-miR-2899	-28.9	416
bta-miR-2899	-27.7	157
bta-miR-2899	-26.7	317
bta-miR-2899	-26.0	567
bta-miR-2899	-24.4	467
bta-miR-2899	-22.1	236
bta-miR-2899	-22.0	9
bta-miR-2899	-21.6	27
bta-miR-2899	-21.1	127
bta-miR-2899	-20.9	361
bta-miR-2899	-19.5	391
bta-miR-2899	-19.3	498
bta-miR-2899	-18.8	650
bta-miR-2899	-17.9	186
bta-miR-2899	-17.0	72
bta-miR-2899	-16.7	274
bta-miR-2899	-16.3	734
bta-miR-2899	-15.9	699
bta-miR-2899	-15.6	587
bta-miR-2899	-13.7	623

bta-miR-2899	-13.6	55
bta-miR-2899	-13.6	516
bta-miR-2899	-13.5	299
bta-miR-2899	-13.4	332
bta-miR-2899	-13.1	541
bta-miR-2899	-12.7	456
bta-miR-2899	-12.5	372
bta-miR-2899	-12.5	207
bta-miR-2899	-11.9	561
bta-miR-2899	-11.8	261
bta-miR-2899	-11.7	431
bta-miR-2899	-10.5	93
bta-miR-2900	-35.9	409
bta-miR-2900	-26.5	98
bta-miR-2900	-26.4	236
bta-miR-2900	-25.4	461
bta-miR-2900	-25.3	559
bta-miR-2900	-24.3	34
bta-miR-2900	-22.3	316
bta-miR-2900	-22.1	143
bta-miR-2900	-22.0	390
bta-miR-2900	-20.7	67
bta-miR-2900	-20.6	513
bta-miR-2900	-20.2	342
bta-miR-2900	-20.0	580
bta-miR-2900	-19.4	657
bta-miR-2900	-19.2	541
bta-miR-2900	-18.6	14
bta-miR-2900	-16.9	740
bta-miR-2900	-16.6	191
bta-miR-2900	-15.9	2
bta-miR-2900	-15.5	263
bta-miR-2900	-15.0	285
bta-miR-2900	-14.4	691
bta-miR-2900	-14.4	128
bta-miR-2900	-14.3	368
bta-miR-2900	-14.1	492
bta-miR-2900	-14.1	617
bta-miR-2900	-11.6	170
bta-miR-2900	-10.6	628
bta-miR-2900	-10.4	433
bta-miR-2900	-10.2	55
bta-miR-2901	-28.8	608
bta-miR-2901	-28.2	70
bta-miR-2901	-26.9	472
bta-miR-2901	-24.1	262
bta-miR-2901	-21.8	440
bta-miR-2901	-20.7	407
bta-miR-2901	-20.6	171
bta-miR-2901	-20.4	496
bta-miR-2901	-19.4	20
bta-miR-2901	-19.0	584
bta-miR-2901	-18.4	199
bta-miR-2901	-18.2	358
bta-miR-2901	-17.4	114
bta-miR-2901	-17.1	559
bta-miR-2901	-16.5	654
bta-miR-2901	-15.6	343
bta-miR-2901	-15.2	220

bta-miR-2901	-14.5	525
bta-miR-2901	-14.5	308
bta-miR-2901	-14.3	695
bta-miR-2901	-14.0	425
bta-miR-2901	-13.6	631
bta-miR-2901	-13.5	42
bta-miR-2901	-12.8	240
bta-miR-2901	-12.1	388
bta-miR-2901	-11.9	2
bta-miR-2901	-11.5	281
bta-miR-2901	-10.6	137
bta-miR-2901	-10.6	741
bta-miR-2902	-32.0	468
bta-miR-2902	-29.8	92
bta-miR-2902	-28.1	163
bta-miR-2902	-26.0	55
bta-miR-2902	-25.2	426
bta-miR-2902	-24.4	572
bta-miR-2902	-23.4	324
bta-miR-2902	-23.2	259
bta-miR-2902	-21.6	386
bta-miR-2902	-21.4	135
bta-miR-2902	-20.2	209
bta-miR-2902	-19.4	5
bta-miR-2902	-18.7	364
bta-miR-2902	-18.7	624
bta-miR-2902	-18.4	236
bta-miR-2902	-18.1	650
bta-miR-2902	-18.0	510
bta-miR-2902	-16.6	33
bta-miR-2902	-16.3	308
bta-miR-2902	-15.6	615
bta-miR-2902	-15.2	699
bta-miR-2902	-14.2	190
bta-miR-2902	-13.6	549
bta-miR-2902	-12.9	125
bta-miR-2902	-12.8	608
bta-miR-2902	-12.5	495
bta-miR-2902	-12.1	411
bta-miR-2902	-12.0	739
bta-miR-2902	-11.9	246
bta-miR-2902	-10.9	461
bta-miR-2903	-23.1	605
bta-miR-2903	-20.8	420
bta-miR-2903	-19.8	38
bta-miR-2903	-19.8	477
bta-miR-2903	-19.2	13
bta-miR-2903	-18.7	369
bta-miR-2903	-18.2	266
bta-miR-2903	-17.5	560
bta-miR-2903	-17.3	623
bta-miR-2903	-16.9	155
bta-miR-2903	-16.8	73
bta-miR-2903	-16.6	185
bta-miR-2903	-16.2	231
bta-miR-2903	-14.7	454
bta-miR-2903	-14.5	654
bta-miR-2903	-14.5	346
bta-miR-2903	-13.2	109

bta-miR-2903	-12.8	740
bta-miR-2903	-12.8	542
bta-miR-2903	-12.7	504
bta-miR-2903	-11.3	413
bta-miR-2903	-10.3	525
bta-miR-2903	-10.0	92
bta-miR-2903	-10.0	597
bta-miR-2904	-27.9	70
bta-miR-2904	-25.5	154
bta-miR-2904	-25.5	414
bta-miR-2904	-25.3	563
bta-miR-2904	-25.2	114
bta-miR-2904	-23.8	480
bta-miR-2904	-23.6	604
bta-miR-2904	-23.0	16
bta-miR-2904	-22.8	268
bta-miR-2904	-21.7	454
bta-miR-2904	-21.0	233
bta-miR-2904	-19.3	698
bta-miR-2904	-18.8	348
bta-miR-2904	-17.9	202
bta-miR-2904	-16.3	630
bta-miR-2904	-16.2	308
bta-miR-2904	-14.9	586
bta-miR-2904	-14.4	41
bta-miR-2904	-14.2	371
bta-miR-2904	-14.0	738
bta-miR-2904	-13.4	4
bta-miR-2904	-13.1	136
bta-miR-2904	-12.3	324
bta-miR-2904	-12.2	252
bta-miR-2904	-12.0	395
bta-miR-2904	-11.8	439
bta-miR-2904	-11.4	107
bta-miR-2904	-11.2	517
bta-miR-2904	-11.1	539
bta-miR-2904	-10.5	340
bta-miR-2284w	-21.9	350
bta-miR-2284w	-20.9	240
bta-miR-2284w	-20.8	452
bta-miR-2284w	-19.0	101
bta-miR-2284w	-18.9	592
bta-miR-2284w	-18.7	197
bta-miR-2284w	-18.2	620
bta-miR-2284w	-17.8	506
bta-miR-2284w	-17.2	479
bta-miR-2284w	-16.8	166
bta-miR-2284w	-16.5	405
bta-miR-2284w	-16.0	567
bta-miR-2284w	-15.6	221
bta-miR-2284w	-15.4	69
bta-miR-2284w	-15.0	28
bta-miR-2284w	-14.5	710
bta-miR-2284w	-14.1	306
bta-miR-2284w	-13.5	1
bta-miR-2284w	-13.0	423
bta-miR-2284w	-12.8	327
bta-miR-2284w	-11.5	281
bta-miR-2284w	-11.5	142

bta-miR-2284w	-11.4	49
bta-miR-2284w	-11.3	184
bta-miR-2284w	-10.9	689
bta-miR-2284w	-10.7	91
bta-miR-2284w	-10.7	441
bta-miR-2284w	-10.7	581
bta-miR-2284w	-10.5	552
bta-miR-2284w	-10.5	663
bta-miR-2284w	-10.3	320
bta-miR-3431	-25.7	401
bta-miR-3431	-25.2	144
bta-miR-3431	-24.4	602
bta-miR-3431	-22.9	4
bta-miR-3431	-22.6	459
bta-miR-3431	-22.1	565
bta-miR-3431	-21.8	206
bta-miR-3431	-21.6	73
bta-miR-3431	-21.2	256
bta-miR-3431	-20.7	95
bta-miR-3431	-20.4	488
bta-miR-3431	-19.6	431
bta-miR-3431	-19.0	624
bta-miR-3431	-18.2	354
bta-miR-3431	-16.9	121
bta-miR-3431	-16.0	180
bta-miR-3431	-14.5	235
bta-miR-3431	-14.4	325
bta-miR-3431	-14.1	514
bta-miR-3431	-13.9	446
bta-miR-3431	-13.9	53
bta-miR-3431	-13.7	645
bta-miR-3431	-13.5	532
bta-miR-3431	-13.5	379
bta-miR-3431	-13.3	739
bta-miR-3431	-13.1	32
bta-miR-3431	-12.5	191
bta-miR-3431	-12.3	690
bta-miR-3431	-11.6	317
bta-miR-3431	-11.4	299
bta-miR-3431	-10.8	551
bta-miR-3431	-10.2	136
bta-miR-2284x	-18.1	166
bta-miR-2284x	-17.6	366
bta-miR-2284x	-16.4	405
bta-miR-2284x	-15.9	108
bta-miR-2284x	-15.7	273
bta-miR-2284x	-15.7	621
bta-miR-2284x	-15.5	48
bta-miR-2284x	-15.4	470
bta-miR-2284x	-15.1	507
bta-miR-2284x	-14.2	327
bta-miR-2284x	-14.1	451
bta-miR-2284x	-13.7	11
bta-miR-2284x	-13.6	596
bta-miR-2284x	-13.6	196
bta-miR-2284x	-13.2	306
bta-miR-2284x	-13.1	241
bta-miR-2284x	-13.1	706
bta-miR-2284x	-13.0	423

bta-miR-2284x	-12.6	223
bta-miR-2284x	-12.4	28
bta-miR-2284x	-11.9	724
bta-miR-2284x	-11.8	91
bta-miR-2284x	-11.6	124
bta-miR-2284x	-11.5	81
bta-miR-2284x	-11.5	581
bta-miR-2284x	-11.5	557
bta-miR-2284x	-11.2	460
bta-miR-2284x	-10.4	669
bta-miR-2284x	-10.3	320
bta-miR-2284x	-10.3	490
bta-miR-2284x	-10.3	604
bta-miR-2284x	-10.3	260
bta-miR-2284x	-10.1	350
bta-miR-3432	-23.1	408
bta-miR-3432	-23.0	119
bta-miR-3432	-21.5	167
bta-miR-3432	-21.5	563
bta-miR-3432	-19.9	483
bta-miR-3432	-18.5	73
bta-miR-3432	-18.4	367
bta-miR-3432	-17.8	51
bta-miR-3432	-17.5	13
bta-miR-3432	-17.4	649
bta-miR-3432	-17.2	210
bta-miR-3432	-17.0	326
bta-miR-3432	-16.8	454
bta-miR-3432	-16.1	586
bta-miR-3432	-15.5	147
bta-miR-3432	-15.1	696
bta-miR-3432	-14.7	247
bta-miR-3432	-14.6	297
bta-miR-3432	-14.6	602
bta-miR-3432	-14.4	383
bta-miR-3432	-14.3	271
bta-miR-3432	-14.0	515
bta-miR-3432	-13.6	729
bta-miR-3432	-13.0	431
bta-miR-3432	-12.3	352
bta-miR-3432	-12.1	106
bta-miR-3432	-11.5	670
bta-miR-3432	-11.3	140
bta-miR-3432	-10.3	635
bta-miR-3432	-10.1	473
bta-miR-3600	-26.3	558
bta-miR-3600	-25.3	423
bta-miR-3600	-20.9	114
bta-miR-3600	-20.9	4
bta-miR-3600	-20.3	61
bta-miR-3600	-20.0	408
bta-miR-3600	-19.1	154
bta-miR-3600	-17.6	616
bta-miR-3600	-17.5	486
bta-miR-3600	-16.9	357
bta-miR-3600	-16.7	204
bta-miR-3600	-16.4	28
bta-miR-3600	-16.0	265
bta-miR-3600	-16.0	233

bta-miR-3600	-16.0	586
bta-miR-3600	-15.7	542
bta-miR-3600	-15.3	473
bta-miR-3600	-14.8	742
bta-miR-3600	-14.6	512
bta-miR-3600	-13.9	663
bta-miR-3600	-13.6	342
bta-miR-3600	-12.5	375
bta-miR-3600	-12.5	303
bta-miR-3600	-11.9	96
bta-miR-3600	-11.6	696
bta-miR-3600	-11.6	605
bta-miR-3600	-11.4	51
bta-miR-3600	-10.8	462
bta-miR-3600	-10.4	643
bta-miR-3600	-10.1	43
bta-miR-2957	-24.0	20
bta-miR-2957	-23.5	603
bta-miR-2957	-20.2	263
bta-miR-2957	-19.8	358
bta-miR-2957	-19.8	408
bta-miR-2957	-19.5	488
bta-miR-2957	-17.2	582
bta-miR-2957	-17.1	200
bta-miR-2957	-17.1	60
bta-miR-2957	-16.3	435
bta-miR-2957	-15.9	165
bta-miR-2957	-15.4	3
bta-miR-2957	-15.4	516
bta-miR-2957	-14.3	697
bta-miR-2957	-13.7	631
bta-miR-2957	-13.1	88
bta-miR-2957	-13.0	234
bta-miR-2957	-13.0	379
bta-miR-2957	-12.9	344
bta-miR-2957	-12.8	543
bta-miR-2957	-12.4	185
bta-miR-2957	-12.2	451
bta-miR-2957	-11.9	729
bta-miR-2957	-11.8	655
bta-miR-2957	-11.6	566
bta-miR-2957	-11.1	115
bta-miR-2957	-10.1	463
bta-miR-3578	-17.9	611
bta-miR-3578	-16.9	405
bta-miR-3578	-16.1	2
bta-miR-3578	-15.5	434
bta-miR-3578	-15.4	548
bta-miR-3578	-15.3	69
bta-miR-3578	-14.7	492
bta-miR-3578	-14.1	257
bta-miR-3578	-13.9	630
bta-miR-3578	-12.7	454
bta-miR-3578	-12.2	36
bta-miR-3578	-11.9	517
bta-miR-3578	-11.9	709
bta-miR-3578	-11.1	357
bta-miR-3578	-10.4	537
bta-miR-3578	-10.2	218

bta-miR-3578	-10.0	388
bta-miR-3578	-10.0	575
bta-miR-409b	-27.9	16
bta-miR-409b	-24.3	412
bta-miR-409b	-23.7	616
bta-miR-409b	-22.7	496
bta-miR-409b	-22.6	229
bta-miR-409b	-21.5	80
bta-miR-409b	-20.0	546
bta-miR-409b	-19.3	265
bta-miR-409b	-19.2	454
bta-miR-409b	-18.8	307
bta-miR-409b	-16.9	344
bta-miR-409b	-16.7	62
bta-miR-409b	-16.5	583
bta-miR-409b	-16.2	657
bta-miR-409b	-15.6	130
bta-miR-409b	-15.4	39
bta-miR-409b	-15.1	519
bta-miR-409b	-15.0	156
bta-miR-409b	-15.0	185
bta-miR-409b	-14.6	478
bta-miR-409b	-14.4	696
bta-miR-409b	-13.9	250
bta-miR-409b	-13.9	376
bta-miR-409b	-13.5	109
bta-miR-409b	-13.5	743
bta-miR-409b	-13.3	327
bta-miR-409b	-13.1	172
bta-miR-409b	-12.2	199
bta-miR-409b	-12.2	429
bta-miR-409b	-11.1	606
bta-miR-409b	-10.9	293
bta-miR-409b	-10.3	366
bta-miR-409b	-10.2	404
bta-miR-409b	-10.0	436
bta-miR-3601	-24.4	449
bta-miR-3601	-23.5	82
bta-miR-3601	-21.5	614
bta-miR-3601	-18.7	1
bta-miR-3601	-18.0	267
bta-miR-3601	-17.3	173
bta-miR-3601	-16.5	39
bta-miR-3601	-15.9	598
bta-miR-3601	-15.5	422
bta-miR-3601	-15.3	215
bta-miR-3601	-14.7	497
bta-miR-3601	-14.5	371
bta-miR-3601	-14.1	298
bta-miR-3601	-13.3	576
bta-miR-3601	-12.8	20
bta-miR-3601	-12.5	113
bta-miR-3601	-12.4	655
bta-miR-3601	-12.1	343
bta-miR-3601	-10.9	140
bta-miR-3601	-10.9	384
bta-miR-3601	-10.8	523
bta-miR-3601	-10.6	710
bta-miR-3601	-10.5	250

bta-miR-3601	-10.1	412
bta-miR-3602	-22.2	657
bta-miR-3602	-21.0	408
bta-miR-3602	-20.0	13
bta-miR-3602	-19.8	86
bta-miR-3602	-19.5	562
bta-miR-3602	-18.7	459
bta-miR-3602	-18.5	492
bta-miR-3602	-18.1	341
bta-miR-3602	-17.8	162
bta-miR-3602	-17.7	628
bta-miR-3602	-17.4	307
bta-miR-3602	-17.4	116
bta-miR-3602	-17.0	360
bta-miR-3602	-16.7	477
bta-miR-3602	-16.5	40
bta-miR-3602	-15.3	180
bta-miR-3602	-14.9	244
bta-miR-3602	-14.8	266
bta-miR-3602	-14.6	615
bta-miR-3602	-14.1	142
bta-miR-3602	-13.8	541
bta-miR-3602	-12.5	226
bta-miR-3602	-12.2	596
bta-miR-3602	-12.1	66
bta-miR-3602	-12.0	330
bta-miR-3602	-12.0	702
bta-miR-3602	-11.6	387
bta-miR-3602	-11.5	428
bta-miR-3602	-10.9	206
bta-miR-3602	-10.9	445
bta-miR-3602	-10.8	377
bta-miR-3602	-10.3	734
bta-miR-26c	-22.8	573
bta-miR-26c	-22.5	76
bta-miR-26c	-21.8	411
bta-miR-26c	-20.1	607
bta-miR-26c	-18.9	4
bta-miR-26c	-17.4	55
bta-miR-26c	-17.3	461
bta-miR-26c	-16.8	263
bta-miR-26c	-16.7	227
bta-miR-26c	-16.0	437
bta-miR-26c	-15.0	23
bta-miR-26c	-14.6	485
bta-miR-26c	-14.3	145
bta-miR-26c	-14.3	169
bta-miR-26c	-14.3	357
bta-miR-26c	-14.0	680
bta-miR-26c	-13.1	514
bta-miR-26c	-12.7	202
bta-miR-26c	-12.0	123
bta-miR-26c	-11.3	626
bta-miR-26c	-11.2	651
bta-miR-26c	-11.1	311
bta-miR-26c	-10.9	390
bta-miR-26c	-10.0	732
bta-miR-3604	-23.2	10
bta-miR-3604	-22.1	410

bta-miR-3604	-21.0	373
bta-miR-3604	-19.2	494
bta-miR-3604	-18.0	617
bta-miR-3604	-17.9	80
bta-miR-3604	-17.8	264
bta-miR-3604	-16.9	60
bta-miR-3604	-15.7	576
bta-miR-3604	-15.6	632
bta-miR-3604	-14.1	424
bta-miR-3604	-13.7	115
bta-miR-3604	-13.5	555
bta-miR-3604	-13.4	695
bta-miR-3604	-12.6	199
bta-miR-3604	-12.0	344
bta-miR-3604	-11.8	463
bta-miR-3604	-11.5	393
bta-miR-3604	-11.1	230
bta-miR-3604	-10.9	741
bta-miR-3604	-10.7	165
bta-miR-3604	-10.6	530
bta-miR-3604	-10.6	541
bta-miR-3604	-10.6	666
bta-miR-3604	-10.4	602
bta-miR-3604	-10.2	4
bta-miR-3596	-23.2	615
bta-miR-3596	-18.9	657
bta-miR-3596	-18.8	69
bta-miR-3596	-17.4	11
bta-miR-3596	-16.3	478
bta-miR-3596	-16.0	448
bta-miR-3596	-14.8	217
bta-miR-3596	-14.6	517
bta-miR-3596	-14.4	412
bta-miR-3596	-14.2	350
bta-miR-3596	-13.9	265
bta-miR-3596	-13.3	575
bta-miR-3596	-13.2	542
bta-miR-3596	-11.6	174
bta-miR-3596	-11.1	737
bta-miR-3596	-10.6	598
